# Effect of Vitamin D_3_ and Omega-3 Fatty Acid Supplementation on Risk of Frailty: An Ancillary Study of a Randomized Clinical Trial

**DOI:** 10.1001/jamanetworkopen.2022.31206

**Published:** 2022-09-13

**Authors:** Ariela R. Orkaby, Rimma Dushkes, Rachel Ward, Luc Djousse, Julie E. Buring, I-Min Lee, Nancy R. Cook, Meryl S. LeBoff, Olivia I. Okereke, Trisha Copeland, JoAnn E. Manson

**Affiliations:** 1 New England Geriatric Research, Education, and Clinical Center, Veterans Affairs Boston Healthcare System, Boston, Massachusetts; 2 Division of Aging, Brigham and Women’s Hospital, Boston, Massachusetts; 3 Harvard Medical School, Boston, Massachusetts; 4 Division of Preventive Medicine, Department of Medicine, Brigham and Women’s Hospital, Boston, Massachusetts; 5 Massachusetts Veterans Epidemiology Research and Information Center, Veterans Affairs Boston Healthcare System, Boston, Massachusetts; 6 Department of Epidemiology, Harvard T. H. Chan School of Public Health, Boston, Massachusetts; 7 Endocrinology, Diabetes, and Hypertension Division, Brigham and Women’s Hospital, Boston, Massachusetts; 8 Department of Medicine, Brigham and Women’s Hospital, Boston, Massachusetts; 9 Department of Psychiatry, Massachusetts General Hospital, Boston, Massachusetts

## Abstract

**Importance:**

Preventive strategies for frailty are needed. Whether supplements with anti-inflammatory properties, such as vitamin D_3_ or marine omega-3 fatty acids, are useful for frailty prevention is unknown.

**Objective:**

To test the effects of vitamin D_3_ and omega-3 supplements on change in frailty in older individuals.

**Design, Setting, and Participants:**

This study was conducted in 2021, as a prespecified ancillary to the Vitamin D and Omega-3 (VITAL) trial, a 2 × 2 factorial randomized clinical trial. A total of 25 871 individuals (men aged ≥50 years and women aged ≥55 years), without cancer or cardiovascular disease and with data on frailty, were recruited across all 50 US states from November 2011 to March 2014 and followed up through December 31, 2017. Data analysis for the ancillary study was conducted from December 1, 2019, to March 30, 2022.

**Interventions:**

Vitamin D_3_, 2000 IU/d, and marine omega-3 fatty acids, 1 g/d.

**Main Outcomes and Measures:**

Frailty was measured using a validated 36-item frailty index that includes measures of function, cognition, mood, and comorbidities from annual questionnaires. Change in frailty score from baseline to year 5, according to randomization, using an intention-to-treat protocol, was assessed using repeated measures. Cox proportional hazards regression models assessed incident frailty. In subgroup analysis, an alternative frailty definition, the physical phenotype, was used as a sensitivity analysis.

**Results:**

Of 25 871 VITAL trial participants randomized, 25 057 had sufficient data to calculate a frailty index. Baseline mean (SD) age was 67.2 (7.0) years, and 12 698 (50.7.%) were women. Mean (SD) frailty score was 0.109 (0.090) (range, 0.00-0.685), and 3174 individuals (12.7%) were frail. During a median 5-year follow-up, mean (SD) frailty scores increased to 0.121 (0.099) (range, 0.00-0.792). Neither vitamin D_3_ nor omega-3 fatty acid supplementation affected mean frailty scores over time (mean difference at year 5: vitamin D_3_, −0.0002; *P* = .85; omega-3 fatty acid, −0.0001; *P* = .90) or rate of change in mean frailty score (interaction with time: vitamin D_3_; *P* = .98; omega-3 fatty acid; *P* = .13) Incident frailty remained similar over time (interaction with time: vitamin D_3_, *P* = .90; omega-3 fatty acid; *P* = .32). Results were unchanged using the frailty physical phenotype.

**Conclusions and Relevance:**

In this ancillary study of the VITAL randomized clinical trial, treatment with vitamin D_3_ or omega-3 fatty acid supplementation, compared with placebo, did not affect the rate of frailty change or incidence over time. These results do not support routine use of either vitamin D_3_ or omega-3 fatty acid supplementation for frailty prevention in generally healthy community-dwelling older adults not selected for vitamin D_3_ deficiency.

**Trial Registration:**

ClinicalTrials.gov Identifier: NCT01169259

## Key Points

**Question:**

Does supplementation with vitamin D_3_ and/or omega-3 fatty acids reduce the risk of frailty?

**Findings:**

In this ancillary study of a randomized clinical trial including 25 871 individuals aged 50 years or older, neither vitamin D_3_, 2000 IU/d, nor omega-3 fatty acid, 1 g/d, supplementation, compared with placebo, significantly affected change in frailty score during 5 years of treatment. Results were unchanged using the frailty physical phenotype.

**Meaning:**

The results of this ancillary study do not support the routine use of vitamin D_3_ or omega-3 fatty acid supplementation in community-dwelling older adults for the prevention of frailty.

## Teaser

This ancillary study of a randomized clinical trial investigates the effects of vitamin D3 with omega-3 fatty acid supplementation on frailty in older adults.

## Introduction

The rapid increase of the worldwide population of aging individuals has led to increased urgency to identify potential preventive strategies for frailty. Frailty is a syndrome of decreased physiologic reserve in the face of external stressors that is associated with an increased risk of morbidity and mortality.^1,2,3^ Although frailty is independent of age, it increases in prevalence with age, with estimates that up to half of adults aged 85 years and older are living with frailty.^1^ Chronic inflammation is a primary hypothesized mechanism leading to frailty.^4^ Whether medications and supplements with anti-inflammatory properties can lower the risk of frailty remains uncertain.^1,5^

The Vitamin D and Omega-3 (VITAL) trial was conducted to assess the efficacy of vitamin D_3_ and omega-3 fatty acid supplementation on the primary outcomes of cancer or cardiovascular disease (CVD).^6,7^ Both of these supplements have anti-inflammatory properties and may have a role in lowering the risk of frailty over time or modifying the trajectory of frailty.^1,8^ Specifically, low serum 25-hydroxyvitamin D (25[OH]D) levels are associated with frailty, potentially as a marker of poor nutrition or through a direct effect on muscle and bone health, both of which are closely linked with frailty.^9^ Observational studies, however, are limited by reverse causation. Studies in mice have suggested that supplementation with vitamin D_3_ may slow the development of frailty,^10^ but observational data in humans have been mixed and study data are limited.^1,10^ Omega-3 fatty acids may have a role in prevention of CVD,^11^ which shares a bidirectional association with frailty.^12^ Moreover, some evidence suggests that omega-3 fatty acid supplementation may improve sarcopenia, or age-related muscle loss, which is closely interrelated with frailty.^13^ Therefore, we sought to test the hypothesis that vitamin D_3_ and omega-3 fatty acid supplementation would lower the risk of frailty, defined according to 2 leading definitions of frailty, over time in community-dwelling older adults who participated in the VITAL trial.

## Methods

### Ethics

The institutional review board at Partners HealthCare–Brigham and Women’s Hospital approved the trial. The trial protocol is available in Supplement 1. The ancillary study protocol is available in Supplement 2. All participants provided written informed consent before enrollment. This study follows the Consolidated Standards of Reporting Trials (CONSORT) reporting guideline for trials.^14^

### Trial Design

Details of the VITAL trial have been described in detail.^6,7^ Briefly, the VITAL trial was a double-blind, placebo-controlled randomized trial that used a 2 × 2 factorial design to test the efficacy of daily supplementation with vitamin D_3_, 2000 IU, and/or marine omega-3 fatty acids, 840 mg (including eicosapentaenoic acid, 460 mg, and docosahexaenoic acid, 380 mg,) for preventing cancer and CVD. The trial enrolled 25 871 men aged ≥50 years and women aged ≥55 years free of CVD and cancer at baseline. Patients were recruited across all 50 US states from November 2011 to March 2014 and followed up through December 31, 2017. Data analysis for the ancillary study was conducted from December 1, 2019, to March 30, 2022.

To capture updated information on health status, lifestyle, and other variables, participants completed questionnaires at baseline (prerandomization), 6 months after the trial started, and then annually throughout the trial. A subgroup of participants who lived within driving distance of Boston, Massachusetts, were invited to participate in in-person assessments at the Clinical and Translational Science Center at Brigham and Women’s Hospital. These visits included a clinical examination that incorporated markers of frailty at baseline, year 2, and year 4.

### Outcome Assessment

The primary outcome of this ancillary study was change in frailty score over time. Although several tools can be used to measure frailty,^1,15^ in the VITAL trial, frailty was primarily defined according to the well-validated, cumulative deficit model developed by Rockwood and colleagues.^16,17^ The Rockwood frailty index (FI) has been used in prospective and retrospective studies of diverse populations around the world^18,19,20,21,22^ and is a particularly useful tool to characterize older adult populations in clinical trials.^23,24,25^ This theory of frailty posits that deficits in health accumulate over the life span. These deficits can be counted to generate an FI and determine an individual’s frailty status.

To be included in the FI, variables must (1) be related to health status, (2) increase in prevalence with age, (3) not saturate in the population (eg, presbyopia), and (4) include a range of systems, such as cognition, function, and morbidity.^17^ For repeated measures, such as in this study, the identical items should be assessed at each frailty measurement. A minimum of 30 variables are typically included, and scores range from 0 to 1, with the 99th percentile for most populations 0.7.^1,17^ For example, an individual with 6 of 30 possible deficits will have a score of 0.2. Population studies suggest that community-dwelling older adults accumulate deficits at a rate of 3% per year,^26^ and clinically meaningful annual changes in an FI score are 0.019 (small change) and 0.057 (large change).^27^ However, in clinical trials, meaningful annual increases as small as 0.005 have been described.^28^ Based on prior literature, frailty scores of 0 to 0.1 are considered nonfrail, 0.1 to 0.2 are prefrail, and higher than 0.2 are frail.^23^

A total of 36 variables were included (eTable 1 in Supplement 3). Variables included in the FI covered domains related to functional status, mood, and comorbidities. The Short-Form 36 was used to extract self-reported information on health status, function, and mood. Annual questionnaires captured information on comorbidities and cognition. Variables are not weighted, rather, within each person, deficits will autoweight. For example, if an individual has hearing loss and arthritis, but no other deficits, they are not frail. Another individual with these same conditions who also has other deficits, such as function or mood, would be prefrail or frail.^16,29^ Participants missing more than 10% of the items required for the FI were excluded. When possible, logical imputation was used to fill in missing data. For example, if an individual reported no trouble climbing several flights of stairs but did not indicate whether they could climb 1 flight of stairs, they were assumed to have no difficulty. Data for self-reported items, such as function, were carried forward from the previous questionnaire if missing in the following year (maximum carry forward 1 year). For validated diagnoses, such as heart failure, once a diagnosis was confirmed, it was carried forward. The only exceptions were for anemia or depression, which could change based on reported diagnoses. Data were available to calculate an FI at baseline and years 3, 4, and 5. In year 5, questionnaires were sent to only 16 639 VITAL trial participants, and 14 287 had sufficient data to calculate frailty in year 5.

In a subgroup of 1054 participants, the Fried physical phenotype was measured at baseline and at follow-up years 2 and 4. The physical phenotype of frailty includes 5 interrelated variables: greater than or equal to 2.3 kg of unintentional weight loss in the previous year, self-reported exhaustion, low energy expenditure according to kilocalories or energy, slow walking speed, and weak grip strength^2^ (eTable 2 in Supplement 3).

### Other Covariates

Several demographic variables and risk factors were assessed at baseline, including age; sex; race and ethnicity, as required by the funder; smoking status; body mass index (BMI) (categorized as <25, 25 to <30, and ≥30 [calculated as weight in kilograms divided by height in meters squared]); alcohol use; baseline 25(OH)D level (dichotomized at <20 vs ≥20 ng/mL); and baseline dietary fish consumption (dichotomized at <1.5 vs ≥1.5 servings/week).^6,7^

### Statistical Analysis

For this ancillary study, we expected a minimum increase in the FI of approximately 0.01 during 5 years for participants randomized to placebo, 5% loss to follow-up per year, and n = 25 000. Given these factors, we would have greater than 90% power provided that randomization to vitamin D_3_ or omega-3 fatty acids slows the rate of deficit accumulation by at least 40% (ie, reduces the mean FI increase during 5 years from 0.01 to 0.006). Baseline characteristics were compared for each treatment group to confirm balance by randomization was maintained.

The association between the FI and risk of mortality over the duration of follow-up was assessed using Cox proportional hazards regression to show validity of the FI, according to standard procedures.^17^ Because there was an a priori assumption of no interaction between vitamin D_3_ and omega-3 fatty acids, all analyses examined the pooled main effects of each supplement on change in frailty over time. The primary outcome was a continuous FI score over the duration of the trial according to randomization to either vitamin D_3_ or omega-3 fatty acids, using intention-to-treat analysis. Repeated-measures models with an unstructured covariance matrix were fit using the SAS PROC MIXED procedure. The associations between vitamin D_3_ and omega-3 fatty acid treatment and change in FI score over time (baseline and years 3, 4, and 5) were assessed using interaction terms between treatment and time. We assessed adjusted means within each treatment group comparing the vitamin D_3_ and omega-3 fatty acid groups with placebo groups and adjusted mean differences in FI level change for each follow-up time point. This analysis was repeated for the subgroup that had the Fried definition of frailty available.

In examining incident frailty, those with an FI score greater than 0.21 were excluded. Participants were followed up until the estimated frailty level was greater than 0.21 at the reported follow-up questionnaire date, death, or the end of the trial, whichever came first. Cox proportional hazards regression models were used to estimate hazard ratios and 95% CIs for vitamin D_3_ or omega-3 fatty acids vs placebo, adjusting for age and sex. Cumulative incidence curves were used to compare the vitamin D_3_, omega-3, and placebo groups at each time point, with linear interpolation between points.

Secondarily, the following prespecified interactions were tested for both vitamin D_3_ and omega-3 fatty acids: median age (66.8 years), race and ethnicity, and sex. In addition, for vitamin D_3_, interactions with baseline 25(OH)D and BMI were assessed. For omega-3 fatty acid, interaction with baseline fish consumption was calculated.

Analyses were conducted using SAS software, version 9.4 (SAS Institute Inc). Statistical significance was set at 2-sided *P* < .05.

## Results

**Figure 1.**
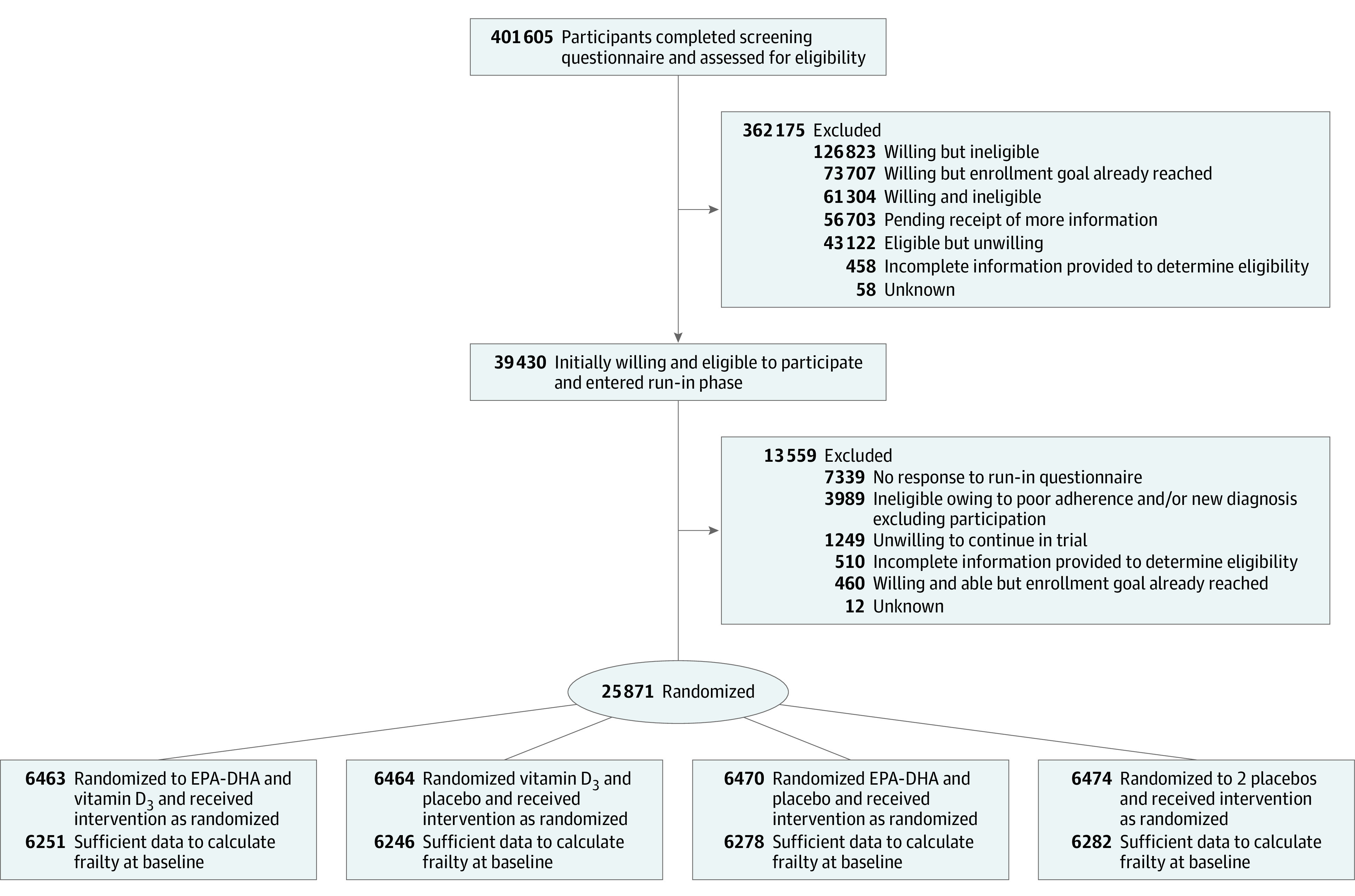
Participant Flow Diagram EPA-DHA indicates eicosapentaenoic acid and docosahexaenoic acid.

At baseline, data were available to calculate frailty for approximately 97% of VITAL trial participants (n = 25 057) (Figure 1) At years 3, 4, and 5, data were available for 22 761 (88%), 21 449 (83%), and 14 287 (55%) participants. Mean (SD) age at baseline was 67.2 (7.0) years, 12 698 were women (50.7.%). Self-reported racial and ethnic groups comprised 357 (1.4%) Asian/Pacific Islander, 4842 (19.3%) Black or African American, 357 (1.4%) Native American/Alaskan Native, 962 (3.8%) non-Black Hispanic, 17 632 (70.4%) non-Hispanic White, and 497 (2.0%) other or unknown individuals. Baseline demographic and other characteristics did not differ substantially by treatment group (Table). Mean (SD) baseline FI was 0.109 (0.090) (range, 0.00-0.685), and 3174 individuals (12.7%) in the cohort were frail, as defined by an FI score greater than 0.21, 7285 (29%) were prefrail (FI score, 0.1-0.21), and 14 598 (58%) were not frail (FI score, <0.1). Baseline distribution of the FI scores for the overall trial and according to vitamin D_3_ or omega-3 fatty acid randomization status are shown in eFigure 1 and eFigure 2 in Supplement 3. Baseline characteristics according to frailty category are reported in eTable 3 in Supplement 3. Over a median of 5.3 years of follow-up, mean (SD) FI scores increased to 0.121 (0.099) (range, 0.00-0.792) and a total of 2487 (11.3%) became frail according to the FI score during follow-up. To validate the FI, the association between FI level and mortality was assessed, and a higher level of frailty was associated with an increased risk of mortality over time^17^ (eFigure 3 in Supplement 3).

**Table.** Baseline Characteristics of 25 057 Participants in the VITAL Trial With Sufficient Frailty Data Available^a^

Characteristic	Vitamin D_3_ and omega-3 fatty acids (n = 6251)	Vitamin D_3_ only (n = 6246)	Omega-3 only (n = 6278)	Placebo only (n = 6282)
Age, mean (SD), y	67.2 (7.0)	67.1 (7.0)	67.2 (7.0)	67.2 (7.1)
Sex, No. (%)				
Female	3170 (50.7)	3165 (50.7)	3182 (50.7)	3181 (50.6)
Male	3081 (49.3)	3081 (49.3)	3096 (49.3)	3101 (49.4)
Racial and ethnic group, No. (%)^b^				
Asian/Pacific Islander	92 (1.5)	82 (1.3)	91 (1.5)	92 (1.5)
Black or African American	1207 (19.7)	1214 (19.9)	1206 (19.7)	1215 (19.8)
Native American/Alaskan Native	92 (1.5)	82 (1.3)	91 (1.5)	92 (1.5)
Non-Black Hispanic	233 (3.8)	257 (4.2)	236 (3.8)	236 (3.8)
Non-Hispanic White	4407 (72.0)	4375 (71.6)	4430 (72.2)	4420 (71.9)
Other or unknown^c^	122 (2.0)	125 (2.1)	118 (1.9)	132 (2.2)
BMI, mean (SD)^d^	28.1 (5.7)	28.1 (5.7)	28.1 (5.7)	28.0 (5.8)
Current smoker, No. (%)	439 (7.1)	456 (7.4)	449 (7.2)	436 (7.0)
Daily alcohol use, No. (%)^e^	1588 (25.6)	1626 (26.3)	1664 (26.7)	1636 (26.3)
Current regular aspirin use, No. (%)^f^	2808 (45.3)	2801 (45.2)	2832 (45.5)	2862 (46.0)
Current use of statins, No. (%)	2212 (35.7)	2197 (35.6)	2157 (34.8)	2122 (34.1)
Baseline 25(OH)D levels, mean (SD), ng/mL	NA	31.0 (10.1)	NA	30.8 (9.9)
Baseline dietary fish consumption ≥1.5 servings/wk, No. (%)	2906 (46.9)	2878 (46.4)	2906 (46.7)	2947 (47.3)
Leisure-time physical activity and stair climbing, total metabolic equivalent of task -hours per week, median (IQR)	15.5 (4.6-31.5)	15.2 (4.5-31.3)	15.5 (4.7-31.7)	15.7 (4.7-32.4)
Frailty Index, median (IQR)	0.08 (0.04-0.15)	0.08 (0.05-0.14)	0.08 (0.05-0.14)	0.08 (0.05-0.15)

Abbreviations: BMI, body mass index (calculated as weight in kilograms divided by height in meters squared); NA, not applicable; 25(OH)D, 25-hydroxyvitamin D; VITAL, Vitamin D and Omega-3.

SI conversion: to convert 25(OH)D to nanomoles per liter, multiply by 2.496.

^a^ There were no significant differences between groups regarding the baseline characteristics.

^b^ Race and ethnic group were reported by participants.

^c^ Patients self-reported other or unknown race or ethnicity (n = 497).

^d^ Data were missing for 2.3% of the participants. For vitamin D_3_ and omega-3 fatty acids, n=6104; vitamin D_3_ only, n=6105; omega-3 only, n=6121; and placebo only, n=6139.

^e^ Alcohol use greater than or equal to 1 drink per day of wine (150-mL glass), liquor (shot), or beer (bottle, can, or glass).

^f^ At least monthly.

### Main Findings

**Figure 2.**
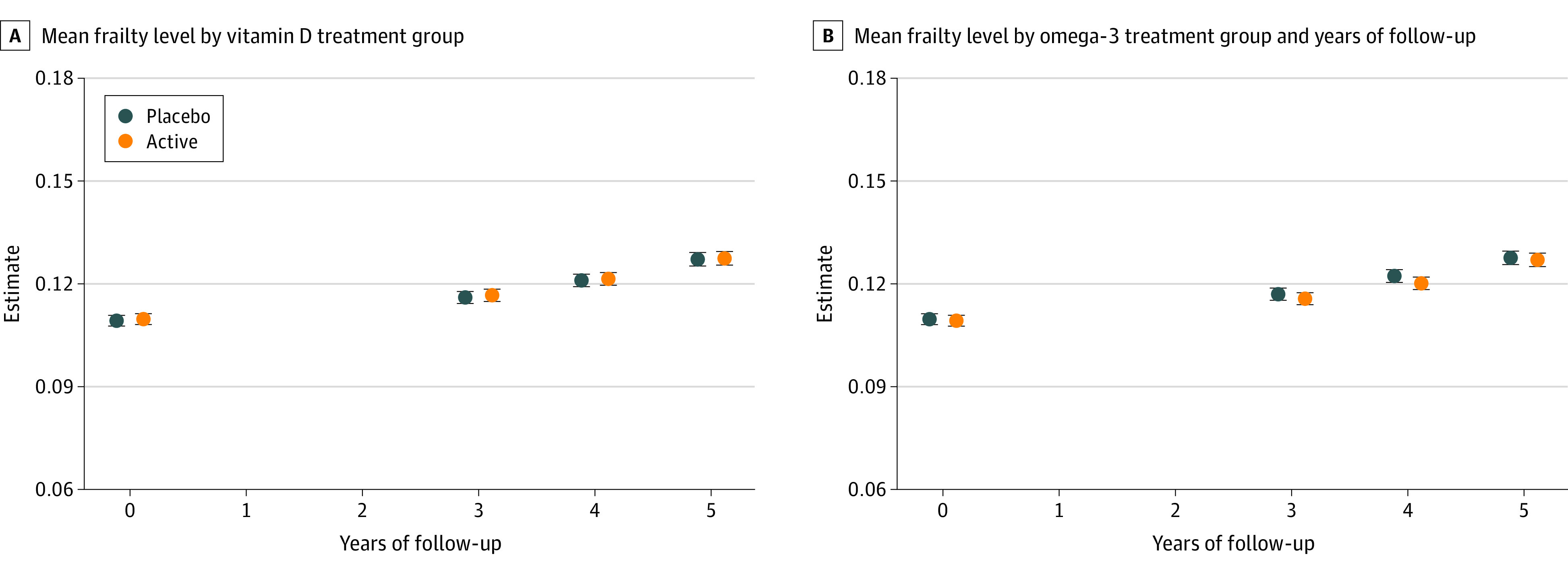
Change in Mean Frailty Levels During the Study A, Change in mean frailty levels in vitamin D group compared with placebo. B, Change in mean frailty levels in omega-3 fatty acids group compared with placebo. Error bars indicate 95% CI.

Neither vitamin D_3_ nor omega-3 fatty acid supplementation affected mean frailty scores over time (vitamin D_3_ mean difference, −0.0002; *P* = .85; and omega-3 fatty acid mean difference, −0.0001; *P* = .90) (eTable 4 in Supplement 3) or the rate of change in FI score over time (interaction with time: vitamin D_3_; *P* = .98; omega-3 fatty acid; *P* = .13) (Figure 2). Incident frailty remained similar between randomization groups over time (interaction with time: *P* = .90 for vitamin D_3_ and *P* = .32 for omega-3 fatty acid) (eFigure 4A and eFigure 4B in Supplement 3) Results were unchanged after adjusting models for baseline age, sex, and the other randomized intervention.

### Interaction With Vitamin D_3_ Supplementation

**Figure 3.**
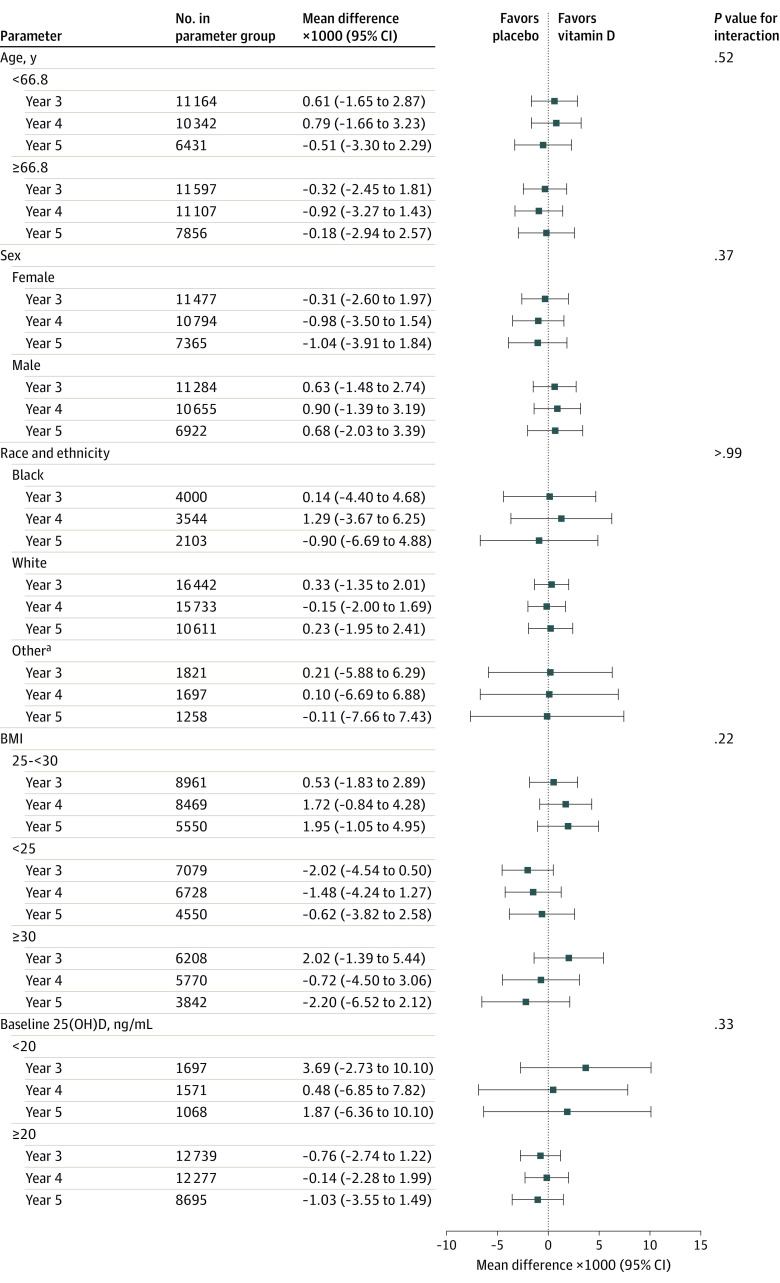
Mean Change in Frailty Score at Each Year Since Randomization According to Vitamin D_3_ and Placebo Groups by Baseline Subgroups BMI indicates body mass index (calculated as weight in kilograms divided by height in meters squared; 25(OH)D, 25-hydroxyvitamin D. ^a^Self-reported Asian/Pacific Islander, Native American/Alaskan Native, non-Black Hispanic, and unknown race and ethnicity.

Older participants in the trial had a greater increase in the FI score over the follow-up period; however, there was no interaction between vitamin D_3_ and age (*P* = .52 for interaction). At baseline, women had a higher mean FI score (0.13 vs 0.09 in men); however, there was no significant interaction between vitamin D_3_ and sex (*P* = .37 for interaction). Black individuals had higher frailty scores at baseline compared with White individuals and those identifying as other race, but there was no significant interaction between vitamin D_3_ and race (*P* > .99 for interaction). Participants with baseline 25(OH)D levels less than 20 ng/mL had a higher baseline frailty score (0.13 vs 0.10), yet there was no significant interaction with vitamin D_3_ (*P* = .33 for interaction). Participants with a BMI less than 25 and 25 to 30 had mean frailty scores at baseline in the nonfrail range, compared with those with a BMI greater than or equal to 30 with FI scores in the high prefrail range; however, there was no interaction between vitamin D_3_ and BMI (*P* = .22 for interaction) (Figure 3; eFigure 5 in Supplement 3).

### Interaction With Omega-3 Fatty Acid Supplementation

**Figure 4.**
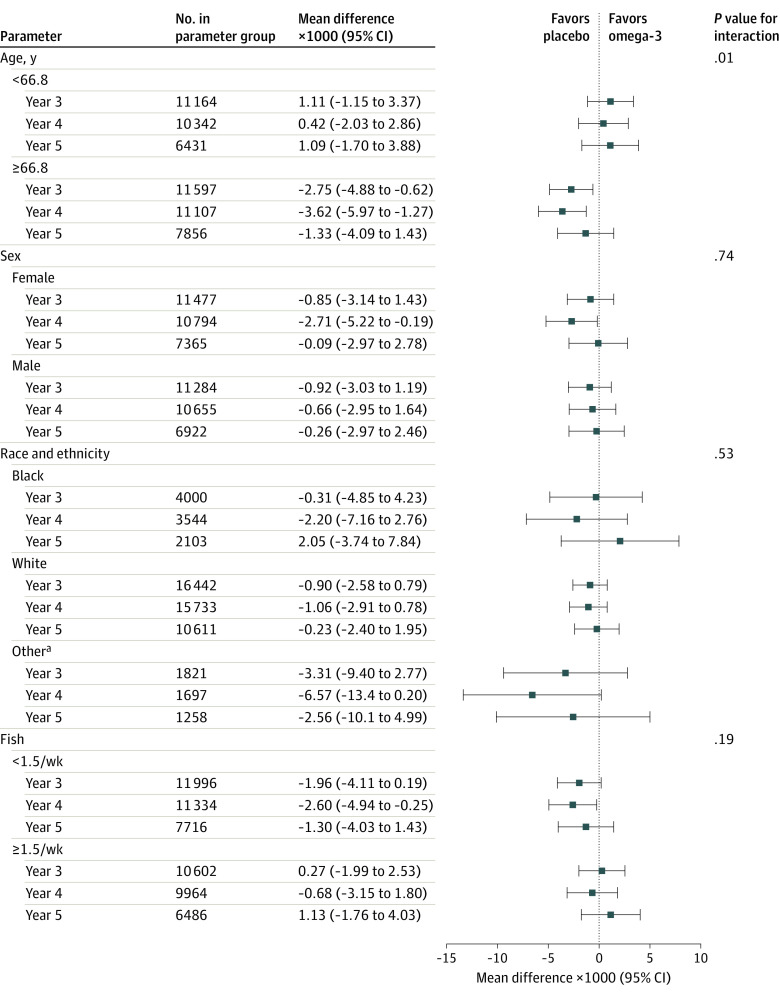
Mean Change in Frailty Score at Each Year Since Randomization According to Omega-3 Fatty Acid and Placebo Groups by Baseline Subgroups ^a^Self-reported Asian/Pacific Islander, Native American/Alaskan Native, non-Black Hispanic, and unknown race and ethnicity.

There were no significant interactions between omega-3 fatty acids and frailty scores by sex (*P* = .74) or race and ethnicity (*P* = .53 for interaction). There was a significant interaction by age (*P* = .01). Frailty scores at baseline were similar between participants who reported eating 1.5 or more servings of fish per week or less than 1.5 servings per week. There was no interaction between baseline fish consumption and omega-3 fatty acid supplementation on frailty scores (*P* = .15 for interaction) (Figure 4; eFigure 6 in Supplement 3).

### Sensitivity Analysis

Data were available to define frailty according to the Fried physical phenotype for 1054 participants. Baseline characteristics for this subgroup are reported in eTable 5 in Supplement 3 and are similar to those of the population in the main trial. Results remained largely unchanged when the Fried definition was used (eTable 6A and B and eFigure 7 and eFigure 8 in Supplement 3).

## Discussion

In this ancillary study of the VITAL randomized clinical trial of community-dwelling participants aged 50 years and older, neither vitamin D_3_, 2000 IU/d, nor omega-3 fatty acid, 1 g/d, supplementation, compared with placebo, affected frailty levels or incidence change in frailty scores over 5 years of treatment. Results did not change significantly after considering interactions by sex, race and ethnicity, BMI, baseline serum 25(OH)D levels, and weekly fish consumption, or when using an alternative definition of frailty, except for a significant interaction by age for omega-3 fatty acid supplementation. These results do not support the routine supplementation of healthy community-dwelling adults with vitamin D_3_ or omega-3 fatty acids for the prevention of frailty.

Frailty, a syndrome of diminished physiologic reserve, is caused by inflammation and worsened by poor nutrition.^1,30,31^ Observational data have shown an association between malnutrition and increased risk of frailty, and supplementation with micronutrients and macronutrients has been hypothesized as a potential approach for both prevention and treatment of frailty.^1,9^ Moreover, sarcopenia and decline in function are closely related, and perhaps on the pathway to frailty.^32^ Frailty is differentiated from sarcopenia as a multisystem impairment leading to an increased vulnerability. Low 25(OH)D concentrations have been implicated in sarcopenia, in part through the presence of 1,25(OH)_2_D receptors in skeletal muscle that lead to alterations in contractility and reduced muscle synthesis.^9,13^ Low concentrations of omega-3 fatty acids have similarly been associated with an increased risk of frailty and sarcopenia.^13^ It is therefore tempting to consider supplementation with vitamin D_3_ and omega-3 fatty acids, both of which have anti-inflammatory properties, as strategies to lower the risk of frailty and sarcopenia.^9,13^ However, the reported findings in this trial on participants largely replete in vitamin D_3_ do not support this practice. It is possible that in a less-nourished population, vitamin D_3_ and omega-3 fatty acids may have a role in prevention of frailty. Regular exercise and the Mediterranean diet are proven strategies for the prevention of frailty and should be encouraged for older adults.^1,28,33,34^

Similar to the VITAL trial, the DO-HEALTH trial randomized community-dwelling older adults in Europe aged 70 years and older in a 2 × 2 × 2 factorial design to vitamin D_3_, 2000 IU/d, omega-3 fatty acid, 1 g/d, and a strength-training exercise program.^35^ Among several primary outcomes, there was no statistically significant difference in function, measured using the short physical performance battery in participants randomized to vitamin D_3_ or omega-3 fatty acid compared with placebo. The short physical performance battery, which includes measurement of gait speed, balance, and chair stands, has also been used as a measure of frailty.^36^ Compared with the VITAL trial, DO-HEALTH focused on an older population and follow-up was shorter at 3 years. A possible lack of efficacy for vitamin D_3_ and omega-3 fatty acids for functional outcomes could have been attributed to the need to begin supplementation earlier in life and continue treatment for longer durations. Results from the VITAL trial, with a younger population and 5.3 years of follow-up, suggest that, even at younger ages and longer duration, there is no benefit of vitamin D_3_ or omega-3 fatty acid supplementation for frailty prevention.

A meta-analysis of 29 randomized clinical trials of vitamin D supplementation that enrolled a total of 5533 participants reported a small increase in overall muscle strength (standard difference in means of 0.17; 95% CI, 0.03-0.31; *P* = .02), although heterogeneity was high (*I*^2^ = 77.7%).^37^ Changes were most notable among participants whose baseline serum 25(OH)D levels were less than 12 ng/mL (to convert to nanomoles per liter, multiply by 2.496). In addition, low serum 25(OHD levels are associated with an increased risk of falls and osteoporotic fractures, which can be an antecedent to or a marker of frailty. However, supplementation with vitamin D_3_ for primary prevention of falls has not reduced the risk of falls and is not routinely recommended.^38,39^ In the VITAL trial cohort, supplementation with vitamin D_3_ vs placebo did not reduce falls.^40^ The data in the present study are in line with results to date that do not suggest a role of vitamin D_3_ for physical performance or frailty prevention.

Omega-3 polyunsaturated fatty acids are postulated to have anabolic effects through activation of the mTOR signaling pathway and lowering insulin resistance, both of which are implicated in sarcopenia, and CVD.^13,41^ In the VITAL trial, omega-3 fatty acid supplementation did not lower the overall incidence of a major CVD event (hazard ratio, 0.92; 95% CI, 0.80-1.06; *P* = .24), although there was a significant effect on lower risk of myocardial infarction, fatal myocardial infarction, and total coronary heart disease. In a meta-analysis of 13 trials that included a total of 127 477 participants followed up for an average of 5 years, there was a significantly lower risk of CVD events in those randomized to omega-3 fatty acids vs placebo.^11^ Furthermore, the Prevention with Mediterranean Diet (PREDIMED) trial demonstrated that high adherence to a Mediterranean diet, which is high in fish consumption, was associated with a lower risk of frailty in post-hoc analysis.^42^ These data have led to the hypothesis that omega-3 fatty acid supplementation may also prevent frailty.^43^ The data in the present study do not support the use of omega-3 fatty acids for prevention of frailty; in fact, in subgroup analyses, there was a significant interaction for age favoring placebo for individuals aged 67 years and older. These subgroup data should be interpreted with caution.

The lack of benefit of vitamin D_3_ or omega-3 fatty acid supplementation on frailty in the VITAL trial may be due to the overall healthy population, as evidenced by only 13% with frailty at baseline. In the general population of older adults of similar ages, prevalence rates of frailty approach 25%, and the rate in those with CVD can be as high as 60%.^12,44^ It is possible that persons who will benefit the most from supplementation with vitamin D_3_ and omega-3 fatty acids are more vulnerable individuals, such as those no longer able to live in the community.

### Strengths and Limitations

This study has strengths. This was a large ancillary study of a randomized clinical trial of community-dwelling older adults that included diverse participants with high follow-up rates and adherence to the study protocol. The use of a well-validated frailty definition that has been applied in other trials is a strength, as is the sensitivity analysis that used an alternative leading definition of frailty. We specifically used these 2 leading definitions of frailty because each identifies somewhat different populations of older adults with frailty and are predicated on different theories of frailty.^45^ Moreover, it is possible that an intervention may affect cumulative deficit frailty differently than phenotypic frailty.^28,46^ The consistent results in this study suggest that there was no effect of either intervention on frailty, regardless of the definition used. Mean follow-up was more than 5 years, with sufficient time expected to see changes in frailty scores.

The study has limitations. The doses used in the trial may not have been optimal for prevention of frailty, and only a single dose was studied. Rates of frailty were lower than in the general population, suggesting an overall healthier cohort and possible healthy volunteer bias. As a result, the study does not generalize to noncommunity dwelling older adults who are the most vulnerable and at risk of poor outcomes from frailty. In addition, direct measures of function and frailty status, such as gait speed, were available only in a subgroup of participants, although the FI score included multiple self-reported items related to functional status.

## Conclusions

In this ancillary study to the VITAL trial, treatment with vitamin D_3_ and/or omega-3 fatty acids, compared with placebo did not affect the frailty scores or the incidence of frailty over time. These results do not support the routine use of either vitamin D_3_ or omega-3 fatty acid supplementation for prevention of frailty in healthy, community-dwelling older adults.

## Notes

Supplement 1.
Trial Protocol

Supplement 2.
Ancillary Trial Protocol

Supplement 3. **eTable 1.** Cumulative Deficit Frailty Index Components for the VITAL Trial **eTable 2.** Fried Physical Phenotype of Frailty in VITAL Subcohort (n=1054) **eTable 3.** Baseline Characteristics of the VITAL Trial (n=25 057) According to Frailty Status **eTable 4. **Adjusted Means at Baseline and Mean Change (95% CI) in Frailty level at Each Year Since Randomization Compared to Baseline, According to Vitamin D vs placebo and Omega-3 vs Placebo Groups **eTable 5. **Baseline Characteristics of 1054 Participants in the VITAL Trial in Person Subgroup **eTable 6A. **Adjusted Means at Baseline and Mean Change (95% CI) in Fried Frailty Level at Each Year Since Randomization Compared to Baseline, According to Vitamin D and Placebo Groups **eTable 6B. **Adjusted Means at Baseline and Mean Change (95% CI) in Fried Frailty Level at Each Year Since Randomization Compared to Baseline, According to n-3 and Placebo Groups **eFigure 1. **Distribution of Frailty Index at Baseline **eFigure 2. **Distribution of Frailty Index at Baseline According to Randomization Group **eFigure 3.** Validation of the FI: Association Between FI Level and Mortality **eFigure 4A.** Effect of Vitamin D_3_ on Incident Frailty Over Time **eFigure 4B.** Effect of Omega-3 on Incident Frailty Over Time **eFigure 5.** Vitamin D_3_ Supplementation and Frailty Subgroups **eFigure 6.** Omega-3 Supplementation and Frailty Subgroups **eFigure 7.** Effect of Vitamin D_3_ on Incident Fried Frailty Over Time **eFigure 8.** Effect of Omega-3 on Incident Fried Frailty Over Time
eReferences

Supplement 4.
Data Sharing Statement
