# Supplementary material for: Effect of Vitamin D3 and Omega-3 Fatty Acid Supplementation on Risk of Frailty: An Ancillary Study of a Randomized Clinical Trial
Source: JAMA Netw Open. Author manuscript; Available in PMC 2022 Nov 3. (PMC9471979; doi:10.1001/jamanetworkopen.2022.31206)
Supplement: NIHMS1841134 — SUPPLEMENT 1. Trial Protocol SUPPLEMENT 2. Ancillary Trial Protocol SUPPLEMENT 3. eTable 1. Cumulative Deficit Frailty Index Components for the VITAL Trial eTable 2. Fried Physical Phenotype of Frailty in VITAL Subcohort (n=1054) eTable 3. Baseline Characteristics of the VITAL Trial (n=25 057) According to Frailty Status eTable 4. Adjusted Means at Baseline and Mean Change (95% CI) in Frailty level at Each Year Since Randomization Compared to Baseline, According to Vitamin D vs placebo and Omega-3 vs Placebo Groups eTable 5. Baseline Characteristics of 1054 Participants in the VITAL Trial in Person Subgroup eTable 6A. Adjusted Means at Baseline and Mean Change (95% CI) in Fried Frailty Level at Each Year Since Randomization Compared to Baseline, According to Vitamin D and Placebo Groups eTable 6B. Adjusted Means at Baseline and Mean Change (95% CI) in Fried Frailty Level at Each Year Since Randomization Compared to Baseline, According to n-3 and Placebo Groups eFigure 1. Distribution of Frailty Index at Baseline eFigure 2. Distribution of Frailty Index at Baseline According to Randomization Group eFigure 3. Validation of the FI: Association Between FI Level and Mortality eFigure 4A. Effect of Vitamin D3 on Incident Frailty Over Time eFigure 4B. Effect of Omega-3 on Incident Frailty Over Time eFigure 5. Vitamin D3 Supplementation and Frailty Subgroups eFigure 6. Omega-3 Supplementation and Frailty Subgroups eFigure 7. Effect of Vitamin D3 on Incident Fried Frailty Over Time eFigure 8. Effect of Omega-3 on Incident Fried Frailty Over Time eReferences SUPPLEMENT 4. Data Sharing Statement [file NIHMS1841134-supplement-NIHMS1841134.pdf]

PROTOCOL FOR THE VITAL TRIAL January 28, 2010

SUMMARY

The VITAL trial (***VIT**amin D and Omeg**A**-3 Tria**L***) is a randomized, double-blind, placebo-controlled, 2x2 factorial trial of vitamin D (in the form of vitamin D_3 [cholecalciferol]) and marine omega-3 fatty acid (eicosapentaenoic acid [EPA] + docosahexaenoic acid [DHA]) supplements in the primary prevention of cancer and cardiovascular disease (CVD). The trial will be conducted among 20,000 men aged ≥60 and women aged ≥65, to be recruited from mailings to 2.5 million persons, including health and other professionals, members of AARP (formerly known as the American Association of Retired Persons), and others. Willing and eligible respondents to the mailings will be enrolled in a 3-month run-in, during which they will receive placebos. Upon successful completion of the run-in, participants who remain willing and eligible will be randomly assigned to one of four treatment groups for 5 years: vitamin D_3 (2000 IU/d) and fish oil (1000 mg capsule containing 840 mg EPA+DHA); vitamin D_3 and fish oil placebo; placebo vitamin D_3 and fish oil; and placebo vitamin D_3 and placebo fish oil. Blood samples will be collected and stored to allow assessment of effect modification by baseline 25-hydroxyvitamin D and omega-3 fatty acid levels, as well as future ancillary studies of genetic/biochemical hypotheses. At 1-year intervals, participants will receive a new supply of pills and a follow-up questionnaire on compliance, possible side effects, and incidence of endpoints. Endpoints will be confirmed by medical record review.

I. BACKGROUND AND SIGNIFICANCE

The VITAL Trial will evaluate the role of vitamin D and long-chain marine omega-3 polyunsaturated fatty acid (eicosapentaenoic acid [EPA] plus docosahexaenoic acid [DHA]) supplements in the primary prevention of cancer and cardiovascular disease (CVD). Although there have been marked advances in our understanding of potentially beneficial effects of vitamin D and omega-3 fatty acids on cancer and cardiovascular risk in recent years, clear gaps in knowledge remain.

Data from laboratory studies, epidemiologic investigations, and small clinical trials strongly suggest a protective effect for vitamin D against cancer, but there are no completed or ongoing large randomized trials of high-dose vitamin D supplements for the primary prevention of cancer in a general population.[1] At a 2007 conference on the role of vitamin D in cancer prevention sponsored by the National Cancer Institute (NCI) and the National Institutes of Health Office of Dietary Supplements (ODS), most speakers "expressed a need for intervention studies with comprehensive assessment of vitamin D status.[1] To quote one such investigator, Dr. Edward Giovannucci, "Further observational studies—and randomized trials if feasible—would be useful in testing the hypothesis that vitamin D lowers cancer risk. … Confirming that vitamin D reduces the risk of cancer incidence or mortality is critical, because current health recommendations typically do not encourage high intakes of vitamin D and tend to discourage sun exposure. Current dietary recommendations are geared only to prevent quite low vitamin D levels. If the association between better vitamin D status and reduced cancer risk is a causal one, the levels of intake currently recommended are probably inadequate. [*Note*: Institute of Medicine guidelines call for 400 IU/d of vitamin D for adults aged 51-70 and 600 IU/d for adults aged >70 to maintain bone health and normal calcium metabolism.[2]] Defining … the optimal levels of

vitamin D for cancer prevention remains a challenge, but further study should be a high priority because the potential for benefit is substantial."[3]

Available data on the connection between vitamin D and CVD are less extensive but also support a possible protective effect. It is thus concerning that >1/2 of U.S. middle-aged and older women and >1/3 of similarly aged men have vitamin D insufficiency.[4, 5] African-American (black) individuals are particularly vulnerable, because darkly pigmented skin is less able to synthesize vitamin D in response to solar radiation and because they tend to have lower intakes of dietary and supplemental vitamin D than whites.[6, 7] Obese individuals are also at above-average risk, presumably due to decreased bioavailability of this fat-soluble vitamin.[8, 9] Given the aging population and soaring obesity prevalence,[10, 11] low vitamin D status is an increasingly important public health issue.

Marine omega-3 fatty acids have shown considerable promise for the primary prevention of CVD in laboratory and observational studies; large randomized trials in secondary prevention or high-risk settings also find benefit. However, there are no completed or ongoing trials of such supplements for the primary prevention of CVD in a general population that has been selected only on the basis of age and *not* on vascular risk factors such as diabetes. The Nutrition Committee of the American Heart Association (AHA) has stated that "Additional studies are needed to confirm and further define the health benefits of omega-3 fatty acid supplements for … primary … prevention."[12] (p. 2775) The conclusion from a 2004 workshop of the ODS and the National Heart, Lung and Blood Institute (NHLBI) Working Group on Future Clinical Research Directions on Omega-3 Fatty Acids and CVD[13] is that "… the body of evidence is consistent with the hypothesis that intake of omega-3 fatty acids reduces CVD but … a definitive trial is needed" and that "A primary prevention trial [would] fill a major gap in knowledge because virtually none of the trials to date have been conducted in a primary prevention population and thus … it is unknown whether omega-3 fatty acids can prevent first events." In terms of methodology, the ODS/NHLBI Working Group recommended a large simple trial, with fish oil given in supplement rather than in food form. It is critically important that such a trial be undertaken in a timely fashion, to avoid a possible dilution of effect from fortification of the food supply with omega-3 fats, an increasingly common practice among U.S. manufacturers.[14]

It is important to clarify the roles of vitamin D and omega-3 fatty acids in the primary prevention of cancer and CVD because their purported health benefits are receiving increasing attention in both the medical literature and the popular press.[15-31] The Canadian Cancer Society recently raised its recommended dose of vitamin D to 1000 IU/d,[32] and many experts are urging a similar move in the U.S.[33] Sales of individual vitamin D supplements at U.S. grocery, drug, and "big box/club" stores jumped 91% to nearly $30 million during 2007 (C. Reider of Pharmavite LLC, personal communication). In 2007, sales of omega-3 fatty acid supplements in the U.S. reached $600 million, an increase of 20% from the prior year; omega-3 supplements are now the fifth-best-selling dietary supplement.[14] In addition, an increasing number of foods are omega-3 fortified; >1200 such products were launched in 2006 alone.[14] The growing enthusiasm for vitamin D and fish oil underscores the urgent need for a timely initiation of a large randomized trial to test these agents rigorously, before their use becomes so prevalent (through supplements and fortification of food) as to render participant recruitment and hypothesis testing impossible. As Dr. Cindy Davis of the NCI has noted, "While vitamin D may well have multiple benefits beyond bone, health professionals and the public should not in a rush to judgment assume that vitamin D is a magic bullet and consume high amounts of vitamin D. More definitive data on both benefits and potential adverse effects of high doses are urgently needed."[34] The same can be said of marine omega-3 fatty acids; the conclusion from a 2004 workshop on

omega-3 fatty acids and CVD sponsored by ODS and the NHLBI was that "a definitive trial is needed."[13]

Indeed, skeptics will note that most single-agent nutritional interventions have not withstood the test of rigorous trials for the primary prevention of cancer and CVD—witness the disappointing findings for antioxidant vitamins.[35-40] However, unlike the antioxidant scenario, the two agents in this proposal appear promising not only in observational studies but also in randomized settings. Omega-3 fatty acids have shown cardiovascular benefit in two large trials in high-risk individuals[41, 42] (one of which also tested vitamin E and found no benefit for it[42]), while vitamin D was suggestive of cancer benefit in a modestly sized trial[43] and of reductions in total and cancer mortality even at a low dose in another.[44] The proposed large-scale randomized controlled trial of vitamin D and omega-3 fatty acids will provide definitive data on their roles in the primary prevention of cancer and CVD.

II. SPECIFIC AIMS

The overall aim of the VITAL trial is to evaluate the role of vitamin D and long-chain marine omega-3 polyunsaturated fatty acid (eicosapentaenoic acid [EPA] plus docosahexaenoic acid [DHA]) supplements in the primary prevention of cancer and cardiovascular disease (CVD).

Primary aims:

1. To test whether vitamin D_3 supplementation reduces the risk of (a) total cancer (excluding non-melanoma skin cancer) and (b) major CVD events (a composite endpoint of myocardial infarction [MI], stroke, and cardiovascular mortality).
2. To test whether EPA+DHA supplementation reduces the risk of (a) total cancer (excluding non-melanoma skin cancer) and (b) major CVD events.

Secondary aims:

1. To test whether vitamin D_3 or EPA+DHA supplementation reduces the risk of (a) incident colorectal cancer, (b) incident breast cancer (in women), (c) incident prostate cancer (in men), and (d) total cancer mortality.
2. To test whether vitamin D_3 or EPA+DHA supplementation reduces the risk of (a) an expanded composite cardiovascular endpoint of MI, stroke, cardiovascular mortality, and coronary revascularization (i.e., coronary artery bypass grafting [CABG] or percutaneous coronary intervention [PCI]) and (b) the individual components of the primary cardiovascular endpoint, particularly total CVD mortality.

Tertiary aims:

1. To explore whether vitamin D_3 and EPA+DHA supplementation exhibit synergistic or additive effects on the risk of (a) total cancer, (b) major CVD events, and (c) the secondary endpoints specified above.
2. To explore whether the effect of vitamin D_3 or EPA+DHA supplementation on cancer and CVD risk varies by (a) baseline blood levels of these nutrients, (b) race/skin pigmentation (for vitamin D_3), and (c) body mass index (BMI) (for vitamin D_3).

III. SUBJECT SELECTION

Study population: VITAL will be conducted among 20,000 apparently healthy participants. Included in the trial will be men aged ≥60 and women aged ≥65, ages at which rates of chronic disease increase substantially. All participants will need to have minimally a high school

education (to complete mail-based questionnaires). Excluded from the study will be individuals who have (1) a history of cancer (except non-melanoma skin cancer), MI, stroke, TIA, angina, CABG, or PCI; (2) have any of the following safety exclusions: use of anticoagulants at baseline, history of kidney stones, renal failure or dialysis, hypercalcemia, hypo- or hyperparathyroidism, severe liver disease (cirrhosis), or sarcoidosis or other granulomatous diseases such as active chronic tuberculosis or Wegener's granulomatosis; (3) have an allergy to fish (for EPA+DHA); (4) have another serious illness that would preclude participation; (5) are consuming greater than 800 IU of vitamin D from all supplemental sources combined (individual vitamin D supplements, calcium+vitamin D supplements, medications with vitamin D [e.g., Fosamax Plus D], and multivitamins), or, and are not willing to decrease or forego such use during the trial (to ensure total supplemental vitamin D intake does not exceed the current safety limit [i.e., NOAEL] of 4000 IU/d set by the European Commission Scientific Committee on Food (EC SCF)); (6) are consuming greater than 1200 mg/d of calcium (the RDA for individuals aged >50[2]) from all supplemental sources combined; (7) are taking fish oil supplements and are not willing to forego their use during the trial; and (8) are not willing to participate, as evidenced by not signing the informed consent form. Although thiazide diuretics and supplemental vitamin D may interact to increase hypercalcemia risk, it is unlikely that such an interaction would occur at the vitamin D dose we propose.[45] Thus, thiazide use will not be an exclusion criterion.

Cohort assembly: A master mailing tape containing 2.5 million names and addresses will be assembled by Listmart, a list broker with access to all commercially available U.S. mailing lists. The listings will include licensed health professionals (e.g., physicians, nurses, pharmacists, veterinarians, physical therapists), other professionals (e.g., executives, managers, financial service professionals, computer professionals, architects, engineers, accountants), AARP members, and subscribers to magazines that have minority readership or appeal to professionals and college-educated individuals (e.g., *Essence, National Geographic* and *Time*). Additionally, it will included individuals who have indicated interest in participating through the study website (www.vitaltrial.org; see paragraph below), publicized via advertisements and feature articles in "healthy-lifestyle" publications, as well as individuals who are currently participating in our other observational epidemiologic studies (e.g., Women's Health Study). Of the 2.5 million names on the mailing tape, an estimated 500,000 will be black or Hispanic. Each invited individual will be assigned a study identification number and mailed an invitation letter and screening questionnaire.

In addition, we will consider a strategy of placing advertisements and feature articles in magazines that have minority readership and in "healthy-lifestyle" publications. The advertisement would explain the objectives of the trial and the general eligibility criteria. Interested individuals would be encouraged to visit a study web site at www.vitaltrial.org where they could review in greater detail the purpose of the trial, what participation involves, and the eligibility criteria. By submitting their name, address and age at the web site, individuals would be assigned a study identification number and mailed the VITAL invitation letter and a screening questionnaire. If necessary, we will also consider inviting eligible participants from groups with large minority representation, such as church groups.

IV. SUBJECT ENROLLMENT

Screening : The W.A. Wilde Company, a direct marketer in Holliston, MA that we have used in our previous trials, will mail screening materials by non-profit bulk rate to each individual in the assembled cohort. The mailings will occur in groupings of approximately 500,000 each, spaced at monthly intervals. The mailing package will include:

- A letter explaining the rationale for VITAL. The letter will outline what participation would entail and provide sources for further information on relevant scientific issues. We will also consider sending a targeted letter to individuals identified as African-American with a high degree of certainty (e.g., *Essence* subscribers). The special invitation letter would emphasize that African-Americans are at higher risk for vitamin D deficiency, as well as heart disease, stroke, and certain cancers.
- A very brief screening questionnaire with items on demographics (age, gender, race/ethnicity, education); medical history (cancer, MI, stroke, CABG and PCI); and current use of supplements containing vitamin D or fish oil.
- A self-addressed, pre-paid envelope for returning study forms.

If the initial response rates are lower than anticipated, a second enrollment request will be mailed to selected non-respondents, who will be identified from mailing groups that had high response and eligibility rates.

Eligible at screening : A computer program will evaluate the screening questionnaire data to classify respondents' eligibility to receive the baseline questionnaire and informed consent form as ineligible, eligibility pending, or eligible. Respondents with eligibility pending will be sent a follow-up form to clarify incomplete or inconsistent responses so that they can be classified as ineligible or eligible. Respondents will be eligible to receive the baseline questionnaire and informed consent form if they meet these criteria: (1) are age ≥60 (men) or ≥65 (women) years; (2) have at least a high school education (to be able to complete mail-based questionnaires); (3) have no history of cancer (except non-melanoma skin cancer), MI, stroke, CABG, or PCI; (4) are consuming no more than 800 IU of vitamin D from all supplemental sources combined (individual vitamin D supplements, calcium+vitamin D supplements, medications with vitamin D [e.g., Fosamax Plus D], and multivitamins), or, if taking, willing to decrease or forego such use during the trial (to ensure total supplemental vitamin D intake does not exceed the current safety limit [i.e., NOAEL] of 4000 IU/d set by the EC SCF); (5) are not taking fish oil supplements, or, if taking, willing to forego their use during the trial.

We estimate that approximately 125,000 respondents will be potentially eligible for participation in the VITAL trial based upon their responses on the screening questionnaire. Respondents who are not eligible will be mailed a letter thanking them for their interest and informing them of the general criteria that determined eligibility.

Enrollment: Every two weeks, the names/addresses of recent respondents, who are eligible to receive the baseline questionnaire, will be electronically compiled in preparation for the mailing of the baseline questionnaire and informed consent form. It is anticipated that there will be 5-10,000 entries per bi-weekly list. W.A. Wilde Company will mail these materials by non-profit bulk rate to each:

- A letter of appreciation for the initial response which also reiterates the purpose of the study and what participation involves.
- An informed consent form.
- A baseline questionnaire with items on demographics (date of birth); medical history (kidney stones, hypercalcemia, kidney failure, sarcoidosis, liver disease, thyroid disease, chronic tuberculosis, other major illnesses); allergy to fish; current use of calcium supplements and other supplements containing vitamin D or fish oil; height and weight; use of anticoagulant medications; and potential effect modifiers such as skin pigmentation and sunlight exposure. Respondents will also be asked for telephone

number(s) and an e-mail address in the event that follow-up needs to be conducted in order to clarify questionnaire responses and to provide their social security number (optional) which will be used for identification purposes only. In addition, the form will contain a question asking whether individuals would be willing to return a blood sample if mailed a convenient blood collection kit.

- A self-addressed, pre-paid envelope for returning study forms.

Eligibility: A computer program will evaluate the questionnaire data to classify respondents' eligibility as ineligible (to enter the run-in), eligibility pending, or eligible. The "eligibility pending" respondents will be sent a follow-up form to clarify incomplete or inconsistent responses. Respondents will be eligible if they meet these criteria: (1) have none of the following safety exclusions: use of anticoagulants, history of kidney stones, renal failure or dialysis, hypercalcemia, hypo- or hyperparathyroidism, severe liver disease (cirrhosis), or sarcoidosis or other granulomatous diseases such as active chronic tuberculosis or Wegener's granulomatosis; (2) have no allergy to fish (for EPA+DHA) or fish oil supplements; (3) have no other serious illness that would preclude participation; (4) are consuming no more than 800 IU of vitamin D from all supplemental sources combined (individual vitamin D supplements, calcium+vitamin D supplements, medications with vitamin D [e.g., Fosamax Plus D], and multivitamins), or, if taking, willing to decrease or forego such use during the trial (to ensure total supplemental vitamin D intake does not exceed the current safety limit [i.e., NOAEL] of 4000 IU/d set by the EC SCF); (5) are consuming no more than 1200 mg/d of calcium (the RDA for individuals aged >50[2]) from all supplemental sources combined; (6) are willing to participate, as evidenced by signing the informed consent form.

We estimate that approximately 40,000 individuals will be both willing and fully eligible to participate in the trial and enter the run-in. Respondents who are not eligible will be mailed a letter thanking them for their interest and informing them of the general criteria that determined eligibility.

Run-in: The experience of other studies conducted by our research group (PHS, WHS, and WAFACS) has demonstrated the success of a run-in period in identifying a group of excellent compliers for long-term follow-up. Because the health experience of participants will be analyzed according to randomized treatment assignment, the use of a run-in phase to eliminate poor compliers prior to randomization will increase the power of the study.[46] During a 3-month run-in period, all participants will take placebo vitamin D and placebo fish oil. As the effects of both agents on cancer prevention are likely to be chronic, it would not be scientifically appropriate to use active agent in the run-in and then randomize to placebo. Moreover, the use of placebos will allow us to most clearly analyze the side effects of the study agents during the randomized treatment period.

At trial enrollment, willing and eligible participants will be mailed a 6-month supply of run-in calendar packs (i.e., 3 months for the actual run-in and 3 months to allow pill-taking to continue uninterrupted while we check participants' responses for continued eligibility and then ship randomized pills to participants who remain eligible). Participants will take two pills per day (one vitamin D placebo pill and one fish oil placebo capsule). At 3 months, participants will receive a follow-up run-in questionnaire asking about continued eligibility, willingness to continue in the trial, pill compliance, and potential side effects. Non-responders will be mailed second and third requests at 6-week intervals.

Participants will be randomized only if they: (1) demonstrate good compliance in pill taking, defined as taking at least two-thirds of the study pills during the run-in; (2) express willingness to continue in the trial; (3) report no new history of cancer (except non-melanoma skin cancer), MI, stroke, TIA, angina, CABG, PCI, use of anticoagulants, kidney stones, hypercalcemia, sarcoidosis, or other serious illness during the run-in; (4) are consuming no more than 1200 mg/d of calcium (the RDA for individuals aged >50[2]) from all supplemental sources combined; and (5) demonstrate continued willingness to limit supplemental vitamin D intake to ≤800 IU/d and to forego the use of fish oil supplements.

We estimate that of the 40,000 initially willing and eligible individuals enrolled in the run-in, 50% (n=20,000) will be compliant and remain willing and eligible for randomization.

Randomization: Willing and eligible participants will be randomly assigned to one of four treatment groups: vitamin D_3 (2000 IU/d) and fish oil (EPA+DHA, 840 mg/d); vitamin D_3 and fish oil placebo; placebo vitamin D_3 and fish oil; and placebo vitamin D_3 and placebo fish oil. Individuals will be randomized to treatment within 5-year age groups using a computer-generated table of random numbers. Within each age group, treatment assignments will be generated in blocks of eight individuals, with two individuals in each of the four treatment combinations. The date of randomized treatment assignment will be the formal "in-study" date. (The use of age stratification during randomization will increase statistical efficiency and power.) Participants will be mailed an annual supply of pills (in calendar packs) and instructed to take two pills – one of vitamin D or placebo and one of fish oil or placebo – each day.

V. STUDY PROCEDURES

A. BLOOD COLLECTION

Baseline: Participants who indicate on their baseline questionnaire a willingness to provide an optional venous blood sample will be sent a blood collection kit during the run-in. Participants who indicate on the questionnaire that they are "not sure" about participating in the blood collection, or who do not answer the question regarding participation, will be called to clarify their degree of interest. The kit will include a consent form, supplies and instructions for having the blood drawn into two EDTA tubes, one heparin tube, and one citrate tube, a gel-filled freezer pack, and an overnight courier air bill. We anticipate that most participants will have their blood drawn by their own healthcare providers or in another healthcare or blood-drawing facility. Participants will be instructed to provide a fasting sample, if possible, and to record the time of venipuncture and the time of their last meal. They will be instructed to send their sample to our blood laboratory in the freezer packs within 24 hours of the blood draw. Upon receipt, the samples will be centrifuged to separate plasma, red blood cells, and buffy coat; aliquoted into 2-ml Nunc vials (including multiple vials of EDTA, heparin, and citrate plasma, and vials for EDTA red blood cells, and buffy coat and stored in individual vial locations in nitrogen freezers(-170 ºC). The process will be completed within several hours of specimen receipt to ensure that samples are frozen within 30-36 hours after venipuncture. Each freezer has an electronic alarm which is monitored 24 hours a day. If problems arise, staff members are paged by the alarm company and can adjust the levels of liquid nitrogen as needed. This system has protected stored samples from inadvertent thawing or warming for nearly 20 years.

The main reason for collecting pre-randomization blood specimens is to provide the opportunity to assess whether treatment effects are modified by baseline blood levels of 25(OH)D and EPA+DHA. When stratifying by 25(OH)D, season (assessed by date of return) and geographic

location (assessed by zip code) will be considered. This resource will also be extremely valuable for future applications for funding of ancillary studies to explore other genetic and biochemical hypotheses in this well-characterized cohort. A nested case-control or case-cohort approach can be used, enabling the study of many promising biochemical and genetic markers (e.g., VDR polymorphisms) as potential predictors of disease risk and modifiers of intervention effects at very low incremental cost.

Follow-up: A follow-up fasting blood sample will be collected in year 2 and year 4 from a randomly selected subset of 2000 participants who provided a baseline fasting blood sample. These follow-up samples, to be collected using the same methods as for the baseline samples, will allow assessment of (a) pill-taking compliance, (b) changes in biomarkers with treatment, and (c) in the placebo group, the effect of changing trends in background fortification with vitamin D and omega-3 fats. The samples will be particularly important for an exploratory analysis of treatment-induced changes in 25(OH)D levels among black participants, as there are few data on this topic.[47] In addition, changes in blood calcium and parathyroid hormone (PTH) levels will be examined as markers of possible hypercalcemia, a potential side effect of high vitamin D intake. The bloods will also be archived for future ancillary studies to evaluate whether treatment with one or both of the study agents leads to changes in biochemical factors, including those in the lipid, glucose tolerance, inflammation, endothelial dysfunction, thrombosis, insulin, and insulin-like growth factor pathways.

Biochemical assays: Blood levels of 25(OH)D and EPA+DHA will be assayed in the final 2 years of the study in a case-cohort design on a subset of participants who provide an analyzable sample at baseline and all 2000 participants who provide a blood sample at follow-up. In addition, baseline and follow-up blood levels of calcium and PTH will be assayed in the latter group. The 25(OH)D assay will be performed by the laboratory of Dr. Bruce Hollis at the Medical University of South Carolina in Charleston (a consultant), and the other assays will be performed by the laboratory of Dr. Nader Rifai at Children's Hospital in Boston, MA.

The sample for analysis of baseline blood levels will be selected to maximize efficiency within budgetary constraints. A case-cohort design,[48] which has efficiency advantages over case-control studies, will be employed to minimize cost while allowing the unbiased estimation of hazard ratios as well as absolute risk for individuals. The common subcohort sample can be used as a reference risk set for more than one outcome—total cancer and total CVD in this proposal. Over 5 years of follow-up, we would expect to accrue approximately 1660 cancer cases and 990 CVD cases, with somewhat smaller numbers if the agents are highly effective. If 60% of participants provide blood specimens, this would include about 996 cancer cases and 594 CVD cases with bloods. The size of the subcohort sample will be initially set at 2410, to reach a total of 4000 blood assays and provide more than two non-cases per case for each outcome. However, 12-13% of the subcohort will likely be in one of the case groups. Thus, we will resample from the main cohort to increase the subcohort size by the number of duplicates to maintain the total number of assays at 4000. Control selection will be stratified by gender and baseline age (within 5-year groups) to frequency-match the distribution in the total case group.

B. ASSESSMENT OF DIETARY AND SUPPLEMENTAL INTAKE

A validated, self-administered semi-quantitative food frequency questionnaire (FFQ) will be mailed to participants during the run-in. This questionnaire, developed by Walter Willett, MD, DrPH, and colleagues, is an efficient, reliable, and accurate instrument for categorizing individuals according to their intake of 32 nutrients, including vitamin D and marine omega-3

fats.[49-54] Participants will be asked to estimate their average intake over the past year of various foods, beverages, and supplements that contain vitamin D, marine omega-3 fats, and other nutrients. Dietary vitamin D intake will be estimated from participants' reported intakes of certain foods, including dairy products, fortified breakfast cereals, fortified juices, dark-meat fish, and cod liver oil. Dietary marine omega-3 fat intake will be estimated from intake of dark-meat fish, canned tuna, other fish, and seafood. Classifying participants by baseline intake of various nutrients will allow us to evaluate whether the study agents' effects vary by such intake. Participants will also be asked to complete the FFQ at 2 years and at trial's end, enabling an examination of dietary changes over time.

Questions on use of non-study supplements or drugs containing vitamin D or EPA+DHA will be asked at baseline, 6 months, and on yearly follow-up questionnaires. We will also assess flaxseed and alpha-linolenic acid intakes. We will ascertain and analyze intake from food and supplements separately, and from both sources combined.

C. TREATMENT

In our research application, we proposed testing 1600 IU (40 μg) per day of vitamin D_3 (cholecalciferol) in VITAL. Since the application was submitted, based on new research and with approval from NCI, we now will test 2000 IU (50 μg) per day instead. We seek to obtain a large-enough difference in vitamin D status between the treatment and placebo groups to detect health benefits, and there appear to be few safety issues associated with this dose. Recent studies have found little toxicity risk with doses up to 10,000 IU/d.[45] (Nevertheless, to further ensure participants' safety, we will exclude from the trial persons with a history of kidney stones, hypercalcemia, or sarcoidosis [Section 5.2]; follow-up calcium and parathyroid hormone [PTH] levels will be obtained in a random subsample of participants [Section 5.4].) Accumulating evidence suggests that vitamin D intakes well above currently recommended amounts of 400 IU/d for adults aged 50-70 and 600 IU/d for adults aged >70[2] are necessary for health benefits. In a review of studies of serum 25(OH)D in relation to bone mineral density, lower-extremity function, dental health, and risk of falls, fractures, and colorectal cancer, Bischoff-Ferrari et al.[56] found that, for all endpoints, advantageous 25(OH)D levels began at 75 nmol/L, and optimal levels were between 90-100 nmol/L. The average older individual requires an oral vitamin D_3 intake of at least 800-1000 IU/d (20-25 μg) to achieve a serum 25(OH)D of 75 nmol/L.[57] Among postmenopausal women in the WHI, 400 IU/d of vitamin D_3 raised median plasma 25(OH)D from 42.3 to only 54.1 nmol/L.[44, 58]

The primary reason for the dose change is a recent study by Aloia et al[59] which indicates that the dose-response for serum 25(OH)D with vitamin D intake is not linear, but that the rate of increase in serum levels decreases at higher levels of intake. At a dose of 800 IU (20 μg/day), the rate of change is 1.2 nmol/L. However, at doses ≥1,400 IU (35 μg/day), the rate of change decreases to 0.7 nmol/L. Thus, extrapolation of these data suggests that with the originally proposed 1600 IU/d (40 μg/day), the expected change in mean serum 25(OH)D levels would be only 28 nmol/L, or a change from an estimated 42 nmol/L at baseline to a final mean level of 70 nmol/L. With 2000 IU/d (50 μg/day), the increase would be 35 nmol/L from baseline to a final mean level 77 nmol/L, which would be above the 75 nmol/L threshold level at which health benefits are believed to occur [56] , as well as a level believed to be free of any risks.

Potential side effects of vitamin D are rare. They include gastrointestinal (GI) upset (presence or absence of symptoms of peptic ulcer, nausea, constipation, diarrhea), and physician diagnosis of hypercalcemia or kidney stones. To minimize the risks of hypercalcemia and

kidney stones, we will require that participants limit their total intake of calcium from all sources, including multivitamins, single supplements of calcium, and other drugs that contain calcium to 1200 mg or less a day,

Active vitamin D_3 (2000 IU [50 μg] per pill) and matching inert placebo will be provided by Pharmavite LLC.

In our research application, we proposed testing a total EPA+DHA dose of 1 mg/d (EPA [500 mg/d] + DHA [500 mg/d]). Since the application was submitted, we have worked with various drug companies to develop soft-gel capsules containing these doses. We now propose to test a total of EPA+DHA dose of 840 mg/d instead, since this is the highest dose of EPA + DHA currently available in a 1000 mg capsule.

We believe that this dose provides the best balance of feasibility, efficacy and safety. We seek to obtain a large-enough difference in omega-3 fatty acid status between the treatment and placebo groups to detect health benefits, and there appear to be few safety issues associated with this dose. Health authorities recommend 400 mg to 1 g/d for cardioprotection.[60] A total dose of 850 mg/d was used in the GISSI-Prevenzione Trial (EPA to DHA ratio, 1:2) and AREDS 2 (EPA to DHA ratio, 2:1), while a dose of 1.8 g/d of EPA was used in JELIS. For this trial, we have selected a total dose of omega-3 fatty acids recommended by the American Heart Association for cardioprotection and demonstrated to be beneficial in one secondary prevention population with minimal side effects.[42] Because the optimal ratio of EPA to DHA is unknown,[61, 62] we have selected a 40:30 ratio of EPA to DHA. The ratio of omega-3 to omega-6 fatty acid intake is between 1:10 and 1:20 in most Western countries, including the U.S., whereas the optimal ratio for disease prevention has been hypothesized to be closer to 1:1 or 1:2,[63, 64] although this is controversial.[65] Indeed, there is growing consensus that the absolute intake of omega-3 is a more important predictor of health than is the ratio of omega-3 to omega-6 intake, at least for cardiovascular outcomes.[65-68] However, given that the average intake of EPA+DHA is 100-200 mg/d among U.S. adults,[60] the proposed intervention of 840 mg/d would be expected to increase the average participant's omega-3 intake by a factor of 4 to 8. Assuming no concurrent change in omega-6 intake, the proposed omega-3 dose would thus have the effect of achieving the purported optimal omega-3 to omega-6 ratio and providing intakes associated with benefits in previous studies.

As with vitamin D, potential side effects of omega-3 fatty acids are rare. They include gastrointestinal (GI) symptoms (stomach upset or pain, nausea, constipation, diarrhea), bleeding (any GI bleed, GI bleeding requiring transfusion, hematuria, easy bruising, epistaxis), skin rash, colds or upper respiratory tract infection, flu-like symptoms, bad taste in mouth, and physician diagnosis of atrial fibrillation or other irregular rhythms. The FDA has concluded that marine omega-3 fatty acid doses of up to 3 g/d are "Generally Recognized as Safe."[69] The AHA also has concluded that these risks are very low or low at doses of up to 1 g/d and low to moderate at doses of 1-3 g/d.[12] Because they undergo an extensive purification process, high-quality fish oil supplements are free of environmental toxins (e.g., methylmercury, polychlorinated biphenyls [PCBs], and dioxins) found in some fish.[12, 71]

Active fish oil (1000 mg capsule containing 840 mg EPA + DHA) and matching inert placebo will be provided by Pronova BioPharma ASA of Norway.

D. FOLLOW-UP

With regard to follow-up, at 6 months, participants will be mailed a very brief follow-up questionnaire and at each anniversary date a more detailed questionnaire and a re-supply of calendar packs. The questionnaire will include items on compliance with randomized treatments, use of non-trial supplements of vitamin D and fish oil, use of calcium supplements, development of major illnesses, dietary intakes of vitamin D and fish, and updated risk factors related to cancer and CVD. Information on potential side effects of the study agents will be elicited for monitoring by the Data and Safety Monitoring Board. For fish oil, these side effects include GI symptoms (stomach upset or pain, nausea, constipation, diarrhea), bleeding (any GI bleed, GI bleeding, hematuria, easy bruising, epistaxis), skin rash, colds or upper respiratory tract infection, flu-like symptoms, bad taste in mouth, and physician diagnosis of atrial fibrillation or other irregular rhythms. For vitamin D, these side effects include GI symptoms as listed above, and physician diagnosis of hypercalcemia or kidney stones. Non-responders will be mailed a second and third request and will then be telephoned to collect study data. At the very least, vital status will be ascertained. Participants will be instructed to discontinue their study pills if, during follow-up, they receive a diagnosis of kidney stones, hypercalcemia, sarcoidosis, or other safety-exclusion conditions. Likewise, participants will be advised to discontinue the vitamin D component if, in the course of follow-up, they report taking supplementary vitamin D (not from diet) that amounts to greater than 800 IU per day or daily calcium supplements that amount to greater than 1200 mg per day.

At 6-month intervals between annual follow-ups, participants will be mailed an interim form with questions on the development of primary endpoints (cancer, MI, and stroke), difficulties with pill compliance, and address changes. Participants will be asked to return the form only if one or more questions apply. A newsletter will be included with each interim mailing to keep participants abreast of the trial's progress and provide information on related health topics. The interim follow-up will help the staff maintain accurate contact information and provide a head start in addressing compliance issues and timely collection of medical records to confirm endpoints.

E. COMPLIANCE

Our primary measure of compliance will be the self-reported information provided on the yearly follow-up questionnaires, which will ask about adherence to the pill-taking regimen. Our experience in PHS, WHS and WAFACS indicates that although most participants try diligently to adhere to the regimen, those who do not comply have no embarrassment about describing what they are actually doing. Thus, blood levels have shown near-perfect correlations with self-reported questionnaire data on adherence.[72] Because VITAL participants will reside throughout the U.S., it will not be possible to obtain blood samples for a validity study on all, or even a random sample of, participants. Thus, at 6-months and thereafter every year we will visit unannounced 100 New England-area participants, to request an on-the-spot blood sample to be analyzed for 25(OH)D and EPA+DHA levels. The distribution of these values will be compared between the active and placebo groups, and compared with the questionnaire data on compliance, as a test for validity. Individuals contacted in this manner will be asked to sign an informed consent for the blood draw. Additionally, we will be able to assess 25 (OH)D and EPA + DHA levels in the 2000 randomly selected participants who will be asked to provide a blood sample at year 2 and year 4, according to active and placebo groups.

F. ENDPOINT ASCERTAINMENT AND VALIDATION

The primary cancer endpoint of VITAL will be total cancer incidence (excluding non-melanoma skin cancer), with cancer mortality and the individual sites of colorectal, breast, and prostate cancer as secondary endpoints. For CVD, the primary endpoint will be a composite endpoint of MI, stroke, and CVD mortality. Secondary endpoints will include a second composite endpoint adding revascularization procedures of CABG and PCI, as well as the individual endpoints of MI, stroke, revascularization, and CVD mortality.

Participants who self-report one of the above study endpoints will be asked to sign a medical release form authorizing VITAL staff to obtain hospital/physician records. The request will be accompanied by a cover letter expressing sympathy for the diagnosis and explaining HIPAA guidelines and the scientific importance of record validation. Non-responders will be sent two additional requests for the medical release form. After the release is obtained, a copy will be sent to the treating hospital/physician. If there is no response within 1 month, a second request will be mailed, followed by a phone call.

After all records are obtained, an Endpoints Committee of physicians, blinded to the randomized treatment assignment, will review the file and, using a defined protocol, will confirm or disconfirm the case. A cancer diagnosis will be confirmed with histologic or cytologic evidence. In the absence of these diagnostic tests, strong clinical evidence accompanied by radiologic evidence or laboratory markers (e.g., PSA levels) will be used to confirm cancer occurrence. The histologic type, grade, and stage of cancer will be recorded.[73] MI will be confirmed using Joint European Society of Cardiology/American College of Cardiology Foundation/ American Heart Association/World Heart Federation (ESC/ACCF/AHA/WHF) Task Force for the Redefinition of Myocardial Infarction criteria.[74] Stroke will be confirmed and categorized according to Trial of Org 10172 in Acute Stroke Treatment (TOAST) criteria.[75] Death due to a cardiovascular cause will be confirmed by convincing evidence of a CVD event from all available sources, including death certificates, hospital records, autopsy, and observer accounts (for deaths outside the hospital).

For reported deaths, the validation process will be similar. A condolence letter will be mailed to the family, requesting permission to obtain medical records and a copy of the death certificate. If the family does not provide the death certificate, a copy will be requested from the state vital records bureau where the participant died. The Endpoints Committee will review all records relevant to the death and assign an ICD code. At the end of the trial, we will search the National Death Index Plus (NDI-Plus) data base for known deaths for cases where we could not obtain records. NDI-Plus provides an ICD-coded cause of death based on death certificate information. We will also use the NDI to search for participants who become lost to follow-up.

G. DATA MANAGEMENT

Because participants will be followed solely by mail, our computing system is a critical feature of effective follow-up. This system, which was developed and fine-tuned in our previous trials, will track each participant's stage in the study and level of participation. It will automatically generate letters, questionnaires, and phone call reminders at the appropriate times. Names, addresses, telephone numbers, participation status, and processing information will be kept up to date, and data from questionnaires, letters, and phone calls will be entered into the study database. When talking to a participant, study personnel will need ready access to identifying information, participation level, and the content of previous study-related telephone calls. However, it is also

critical that these data be available only to authorized staff members. Our existing computer and security systems, which balance these considerations, will be used in VITAL.

Questionnaire data will be optically scanned into the computer. The relevant software—TELEform and Alchemy (Cardiff Software)—has been successfully used for several years in the WHS and PHS. Out-of-range, internally inconsistent, and unclear data will be reviewed by a verifier who will edit misread variables. Forms that cannot be scanned, and name and address changes, will be entered using traditional double-entry procedures. All data will undergo additional within-form and across-time checks to verify accuracy. The database will be maintained on a UNIX server. All data files will be backed up nightly, ensuring at least two current copies at all times. Each month, a set of data files will be taken off-site for long-term storage.

Participants' names and contact information will be accessible only to staff members who need the information for their jobs. Endpoint and health-related questionnaire data will be stored in separate files from the processing data and will be accessible only to approved investigators and programmers. In these files, participants are identified only by study ID.

VI. BIOSTATISTICAL ANALYSIS

A. DATA ANALYSIS

Analyses of treatment effects will be based on the intent-to-treat principle. The first analysis will compare baseline characteristics by randomized treatment assignment to ensure that balance was achieved by the randomization. Characteristics to be examined include known risk factors for cancer and CVD, including age; gender; race/ethnicity; BMI; smoking; alcohol use; physical activity; medical conditions such as hypertension, hyperlipidemia, diabetes, and family history of cancer and CVD; and baseline vitamin D and omega-3 fatty acid levels as assessed by dietary questionnaire in all participants and blood assays in a case-cohort subsample. The large sample size, as well as successful balance of known potential confounders, will provide assurance that unmeasured or unknown potential confounders will also be equally distributed across randomized treatment groups.

In this 2x2 factorial design, the primary aim is to compare the main effects of intention-to-treat with vitamin D and with fish oil on cancer and CVD. We will use the Cox proportional hazards model to allow for variable follow-up lengths[76] and will estimate the hazard ratio for each intervention using indicators for treatment exposure, controlling for the second intervention, age, and gender. Because the cohort will consist of older individuals, competing risks due to deaths from other causes will be considered. The primary analysis will estimate the cause-specific hazard and the hazard ratio comparing intervention groups for each outcome of interest by censoring individuals with deaths due to competing causes. To estimate the cumulative incidence function, the subdistribution of each endpoint will be plotted over time.[77, 78] The alternative Fine and Gray approach[79] models the effect of treatment on the subdistribution hazard or directly on the cumulative incidence function. While we will consider this and compare its fit to the model-free cumulative incidence curves,[78] the proportional hazards approach will be our primary analysis.

Similar analyses will be conducted for both cancer and CVD. For cancer, we will estimate the intervention effects on the primary endpoint of total cancer, as well as the secondary outcomes of breast, prostate, and colorectal cancer, and cancer mortality. For CVD we will estimate the

effects on the primary composite endpoint of major CVD (MI, stroke, and cardiovascular mortality); on the secondary composite endpoint that additionally includes coronary revascularization; and on the individual components of MI, stroke, revascularization, and cardiovascular mortality.

Beyond the primary analyses, we will examine effect modification by the other randomized intervention, by baseline risk factors, and by time. We have a particular interest in exploring interactions between the interventions, as well as with the baseline plasma biomarkers of 25(OH)D for vitamin D and of EPA+DHA for fish oil. We hypothesize that the intervention effects may be larger among those with below-median baseline levels, and will examine treatment effects by quartiles of these biomarkers. The biomarker data will be analyzed as a case-cohort study using methods for proportional hazards regression.[80] Because of the frequency matching by age and gender, appropriate stratum-specific weighting of the observations will be employed.[81] We also have a prior interest in the effects of the vitamin D intervention within groups defined by race/ethnicity and skin pigmentation, and by BMI. In addition, we will evaluate effect modification by age and gender, as well as by sunlight exposure, calcium and phosphorus intakes from the FFQ (as these nutrients affect vitamin D bioavailability[82]), and baseline risk factors for cancer and CVD. The latter interaction effects will be interpreted cautiously, as hypothesis generating. Finally, we will examine whether treatment effects vary over time and duration of treatment by examining survival plots and interactions with time. There may be a latent effect on cancer incidence, depending on the stage of carcinogenesis during which these agents act.

We will also compare the incidence of potential side effects in the active *v.* placebo groups for each agent, including the incidence of kidney stones with vitamin D assignment (cf. WHI calcium-vitamin D findings[44]) and the incidence of GI symptoms, skin abnormalities, and bleeding with fish oil assignment (cf. JELIS findings[41]).

B. POWER CALCULATIONS

To calculate power, the following assumptions were made: (1) a 2x2 factorial trial in 10,000 men aged >60 and 10,000 women aged >65; (2) independent and equal allocation of participants to each treatment; (3) an age distribution based on that observed at baseline in our past trials for men aged ≥60 and women aged ≥65; (4) age-specific event rates based on the observed rates in the first 5 years of the PHS II for men and the WHS for women; (5) trial follow-up of 5 years, with little loss to follow-up as achieved in our past trials; and (6) compliance similar to that in published trials upon which our estimated rate ratio (RR) reductions are based. The cited reductions are thus the observed effects we would see in the trial. The corresponding 'true' RR is given assuming an average compliance of 80%. Power is given for a two-sided test using a logrank analysis[83] with a significance level of 0.05.

Cancer: Assuming that only one agent is effective, with 5 years of treatment and follow-up we will have 91% power to detect an observed RR of 0.85 for the primary endpoint of total cancer incidence (Table

Table 1. Power for effects of a single agent on cancer in a factorial trial of 10,000 men aged≥60 and 10,000 women aged≥65 with 5 years of follow-up

Observed RR†	True RR	Total Cancer	Cancer Mortality	Colorectal Cancer	Breast Cancer (Women)	Prostate Cancer (Men)
0.90	0.875	58.7	-	-	-	-
0.85	0.812	91.1	-	-	-	-
0.80	0.750	99.4	52.0	31.8	34.6	70.1
0.75	0.687	>99.9	72.2	47.0	50.9	88.3
0.70	0.625	>99.9	87.3	63.1	67.5	96.9
0.65	0.560	>99.9	95.6	77.6	81.5	99.5
0.60	0.500	>99.9	98.9	88.4	91.2	>99.9

†Observed RR=intent-to-treat RR, including noncompliant participants. Compliance assumed to be 80%. True RR=that with perfect compliance.

1). For prostate cancer, we will have 88% power to detect a 25% risk reduction. For breast cancer, we will have 81% power to detect a 35% risk reduction. For colorectal cancer, we will have 78% power to detect a 35% risk reduction and 88% power to detect a 40% risk reduction. For cancer mortality, we will have 87% power to detect a 30% risk reduction. If both agents are effective in preventing cancer but act independently, power would be reduced slightly due to a somewhat smaller overall number of events. If the agents interact, however, power will be affected to the extent of the interaction. Should the agents act synergistically, power would increase, as illustrated in Table 2 for the outcome of total cancer.

For example, if the effect of each agent alone is a reduction of 10%, but in combination the effect is stronger, with an additional 10% decrease, the RR comparing the combined group to the all placebo group would be 0.73, versus 0.81 with additive effects (on the multiplicative scale). Power for the main effect of each agent would then increase to 86% (or higher with greater synergy). The trial would have power to detect moderate-to-large interactions, as shown in Table 2. Should the agents interact in a subadditive fashion, power would be reduced to the extent of the subadditivity. Power to explore effect modification by baseline blood levels of 25(OH)D and EPA+DHA among those above and below the median level in the cohort will be lower because we anticipate that about 60% of participants will provide blood specimens. We will have about 90% power to detect an interaction of size 0.6.

Power has been shown for a given observed reduction in risk, assuming compliance similar to that seen in current randomized trials such as the WHS and PHS. For total cancer, our power will be excellent with a 5-year follow-up even if compliance is lower. For example, if the true reduction in risk is 20%, the observed RR corresponding to compliance of 80% is 84%, and we have 94% power to detect this level of effect. If compliance is only 75%, the observed RR would be 0.85, for which we would have 91% power. Finally, if compliance is only 70%, the observed RR would be 0.86, and we would still have 87% power to detect an effect of this magnitude.

Table 2. Power for interaction effects on total cancer in a factorial trial of 10,000 men aged ≥60 and 10,000 women aged ≥65 with 5 years of follow-up

RR Single agent†	Interaction†	RR Both agents	Power Main Effect	Power Interaction
0.90	1.0	0.81	56.3	
	0.9	0.73	86.3	
	0.8	0.65	98.0	57.9
	0.7	0.57	99.9	92.1
0.85	1.0	0.72	88.7	
	0.9	0.65	98.1	
	0.8	0.58	99.8	55.0
	0.7	0.51	>99.9	90.2
0.80	1.0	0.64	98.8	
	0.9	0.58	99.9	
	0.8	0.51	>99.9	52.0
	0.7	0.45	>99.9	87.9

†RR = intent-to-treat RR, including noncompliant participants (assuming 80% compliance). Represents the effect among those not assigned to the other intervention and assumes the same effect for both agents. The interaction is the RR for the combined group divided by the product of risks for the two separate groups—i.e., RRint=RRboth/(RR vit D alone* RR fish oil alone). An interaction=1 implies additive effects (no interaction).

CVD: As shown in Table 3, assuming that only one agent is effective, with 5 years of treatment and follow-up we will have 92% power to detect an observed RR of 0.80 for the primary composite endpoint of MI, stroke, and cardiovascular mortality. For the secondary composite

endpoint of MI, stroke, cardiovascular mortality, and coronary revascularization, we will have 99% power to detect a 20% reduction and 90% power to detect a 15% reduction. With a RR of 0.70 we will have 87% power for cardiovascular mortality, 88% power for MI, and 90% power for stroke. If both agents are effective in preventing CVD but act independently, power would be reduced only slightly due to a smaller overall number of events. If the agents act synergistically, power would increase, as described for cancer. If the agents interact in subadditive fashion, power would be reduced to the extent of the subadditivity.

Table 3. Power for effects of a single agent on CVD in a factorial trial of 10,000 men aged ≥60 and 10,000 women aged ≥65, with 5 years of follow-up

Observed RR†	True RR	Major CVD	Total CVD	CVD Mortality	MI	Stroke
0.90	0.875	38.1	57.3	-	-	-
0.85	0.812	71.1	90.2	-	-	-
0.80	0.750	92.5	99.2	51.8	53.5	56.1
0.75	0.687	99.0	>99.9	71.9	73.8	76.3
0.70	0.625	99.9	>99.9	87.1	88.5	90.3
0.65	0.560	>99.9	>99.9	95.6	96.3	97.1
0.60	0.500	>99.9	>99.9	98.9	99.1	99.4

†Observed RR=intent-to-treat RR, including noncompliant participants. Compliance is assumed to be 80%. True RR=that with perfect compliance.

For total CVD, our power would be somewhat lower—but still adequate—with more limited compliance. If the true effect is a 25% reduction, power would be 92% if compliance is 80% (observed RR=0.80), 89% if compliance is 75% (observed RR=0.813), and 84% if compliance is 70% (observed RR=0.825). To achieve 80% power for CVD, we would need an observed RR of at least 0.83, which corresponds to a true reduction of 21% under 80% compliance and 24% under 70% compliance.

VII. RISKS AND DISCOMFORTS

The VITAL trial will test a dose of 2000 IU (50 µg) per day of vitamin D_3 (cholecalciferol) and 840 mg/d of EPA+DHA in VITAL. These doses provide the best balance of efficacy and safety, based on careful review of the literature and extensive consultation with numerous nutritional experts. We seek to obtain an adequate difference in vitamin D and EPA+DHA levels between the treatment and placebo groups to detect health benefits, and there appear to be few safety issues associated with these doses. For vitamin D, the selected dose is at the tolerable upper intake level (UL) for vitamin D_3 (50 µg, or 2000 IU) set by the Food and Nutrition Board of the Institute of Medicine (IOM) and the European Commission Scientific Committee on Food (EC SCF). Also, because we will exclude from the trial persons who report supplemental vitamin D intakes >800 IU/d, the selected dose ensures that no participants will be taking a supplemental vitamin D dose above 2800 IU, which is the no-observed-adverse-effect level (well below the 4000 IU NOAEL specified by the EC SCF. To further ensure participants' safety, we will exclude

from the trial persons with a history of kidney stones, hypercalcemia, renal failure, cirrhosis, or sarcoidosis or other granulomatous disease (Section 5.2), and will obtain calcium and PTH levels in a random subsample of participants. The dose of fish oil used in this trial (840 mg/day) is consistent with guidelines set by health authorities who recommend up to 1 gram/day for cardio-protection. For fish oil, there are no specific exclusions necessary except for those currently taking the supplement and unwilling to forego use during the trial.

Potential side effects of the study agents will be assessed on each follow-up questionnaire. For fish oil, these side effects include gastrointestinal (GI) symptoms (stomach upset or pain, nausea, constipation, diarrhea), bleeding (any GI bleed, GI bleeding requiring transfusion, hematuria, easy bruising, epistaxis), skin rash, colds or upper respiratory tract infection, flu-like symptoms, bad taste in mouth, and physician diagnosis of atrial fibrillation or other irregular rhythms. For vitamin D, these side effects include GI symptoms as listed above, and physician diagnosis of hypercalcemia or kidney stones.

There is also the possibility of social/psychological risk that could result from inadvertent disclosure of confidential information from the questionnaires or blood tests. However, we have many safeguards in place to avoid this possibility, and we have never had an inadvertent breach of confidentiality in any of our trials.

With regard to the risks of the study agents, information on potential side effects discussed above will be elicited on each follow-up questionnaire for monitoring by the Data and Safety Monitoring Board. We will compare the incidence of potential side effects in the active *v.* placebo groups for each agent, including the incidence of kidney stones with vitamin D assignment and the incidence of GI symptoms, skin abnormalities, and bleeding with fish oil assignment.

The potential risk of disclosure of confidential information is guarded against by maintaining completed questionnaires and blood test results in locked offices accessible by authorized personnel only. In questionnaire data files , participants are identified only by study ID. Subject identifiers are stored in separate computer files with password protection, and results will be presented only in the aggregate. In addition, employees involved with the proposed study will be asked to sign a form agreeing not to disclose any information to which they might have access, regardless of their personal perception about its confidentiality. Each employee of the study who has access to study data or has contact with subjects participates in an institutional (Brigham and Women's Hospital) human subjects educational program, which consists of reviewing regulatory and informational documents pertaining to human subjects research; passing a test on ethical principles and regulations governing human subjects research; and signing a statement of commitment to the protection of human subjects. Finally, all such employees are required to participate in HIPAA training

VIII. POTENTIAL BENEFITS

For the majority of participants, there will be few direct benefits from participating in this primary prevention study other than the awareness of being involved in a large endeavor to answer relevant and timely questions regarding the possible benefit of vitamin D and fish oil. During the trial itself, we will receive inquiries from the participants regarding both specific and general health concerns. We will respond to each of those questions, primarily directing participants to published sources or recommending that they see their local health provider who is familiar with their medical history.

The purported health benefits of vitamin D and marine omega-3 fatty acids are receiving increasing attention in the medical literature and the popular press. Sales of vitamin D supplements and omega-3 fatty acid supplements at U.S. stores have increased substantially in recent years. However, definitive data on health benefits and risks of these agents are lacking. Findings from this large clinical trial will clarify the role of vitamin D and marine omega-3 fatty acid supplements in the primary prevention of cancer and CVD in men and women and will help guide individual decisions, clinical recommendations, and public health guidelines.

IX. MONITORING AND QUALITY ASSURANCE

A. SAFETY MONITORING

An independent Data and Safety Monitoring Board (DSMB) will be assembled, consisting of experts in clinical trials, epidemiology, biostatistics, relevant clinical areas of cancer and CVD, and NIH representatives. The Physicians' Health Study, the Women's Health Study, and the Women's Antioxidant and Folic Acid Cardiovascular Study have been monitored by the same DSMB since their inception. As two of these trials have ended and the third is in its last years, we will ask our DSMB members if they would be willing to also monitor VITAL. If they are not able to do so, we will consult with them in assembling a new DSMB.

The DSMB will annually examine the progress of the trial and the unblinded data on study endpoints and possible adverse effects to recommend continuation, alteration of study design, or early termination, as appropriate. Interim trial results will be assessed with the Haybittle-Peto rule,[84, 85] adjusting for multiple looks. In this method, interim results are compared to a z-score of 3 standard deviations (p=0.0027) throughout the trial. The final results may then be interpreted as having close to nominal significance levels. This rule appropriately requires very strong evidence for early stopping, is more conservative than the Pocock[86] and O'Brien-Fleming[87] rules and the alpha-spending function,[88] and can be conducted at convenient times without inducing statistical complexity.[89]

While these rules are intended for the primary endpoints, the goal of VITAL is to assess the overall balance of benefits and risks of the two agents in the primary prevention of cancer and CVD. Thus, consideration will also be given to the secondary endpoints that are needed in the interpretation of overall results. In addition, the monitoring rules will serve solely as guidelines in decisions regarding continuation or stopping of treatment arms. All decisions must be made after examining the totality of evidence, including other trial data, on these agents.

B. MONITORING OF SOURCE DATA AND DRUG DISPENSEMENT

Redundancies will be built into the data processing systems to insure accurate recording of data and proper follow-up. All research forms will be scanned in and the data read by a character-recognition software program (Teleform). Out-of-range, internally inconsistent, and unclear data will be reviewed by a verifier who will edit misread variables. Forms that cannot be scanned, and name and address changes, will be entered using traditional double-entry procedures. All data will undergo additional within-form and across-time checks to verify accuracy. Following data entry, all questionnaire responses that require additional follow-up for missing data, participant comments on the form or for endpoint validation will be manually reviewed to insure correct processing and accurate follow-up. Address changes received from the post office or from participants will be manually keyed by data entry personnel and the resultant files compared and verified.

Great care will be taken to insure that participants receive their randomly assigned drugs. Upon receipt of drug shipments from the manufacturer, each of the four drug groups will be stored in segregated areas. A random sample of drugs bottles or calendar packs will be pulled from each drug group and blindly tested to insure that the packaged contents match the study label. At the time of packaging for shipment to individual participants, listings will be divided according to drug group assignment and only one group will be packaged at a time. The packaging area will be cleaned and cleared prior to packaging and shipment of the next drug group. If drug packages are returned by the postal service as undeliverable or declined by a participant, the contents of the package will be blindly tested as an additional level of quality control.

X. REFERENCES

1. Davis CD, Hartmuller V, Freedman DM, Hartge P, Picciano MF, Swanson CA, Milner JA. Vitamin D and cancer: current dilemmas and future needs. Nutr Rev 2007; 65:S71-4.
2. Institute of Medicine Food and Nutrition Board. Dietary Reference Intakes: Calcium, Phosphorous, Magnesium, Vitamin D and Fluoride. Washington DC: National Academies Press, 1999.
3. Giovannucci E. Strengths and limitations of current epidemiologic studies: vitamin D as a modifier of colon and prostate cancer risk. Nutr Rev 2007; 65:S77-9.
4. Looker AC, Dawson-Hughes B, Calvo MS, Gunter EW, Sahyoun NR. Serum 25-hydroxyvitamin D status of adolescents and adults in two seasonal subpopulations from NHANES III. Bone 2002; 30:771-7.
5. Holick MF. Vitamin D deficiency. N Engl J Med 2007; 357:266-81.
6. Harris SS. Vitamin D and African Americans. J Nutr 2006; 136:1126-9.
7. Moore CE, Murphy MM, Holick MF. Vitamin D intakes by children and adults in the United States differ among ethnic groups. J Nutr 2005; 135:2478-85.
8. Martins D, Wolf M, Pan D, Zadshir A, Tareen N, Thadhani R, Felsenfeld A, Levine B, Mehrotra R, Norris K. Prevalence of cardiovascular risk factors and the serum levels of 25-hydroxyvitamin D in the United States: data from the Third National Health and Nutrition Examination Survey. Arch Intern Med 2007; 167:1159-65.
9. Harris SS, Dawson-Hughes B. Reduced sun exposure does not explain the inverse association of 25-hydroxyvitamin D with percent body fat in older adults. J Clin Endocrinol Metab 2007; 92:3155-7.
10. Hedley AA, Ogden CL, Johnson CL, Carroll MD, Curtin LR, Flegal KM. Prevalence of overweight and obesity among US children, adolescents, and adults, 1999-2002. JAMA 2004; 291:2847-50.
11. Ogden CL, Carroll MD, Curtin LR, McDowell MA, Tabak CJ, Flegal KM. Prevalence of overweight and obesity in the United States, 1999-2004. JAMA 2006; 295:1549-55.
12. Kris-Etherton PM, Harris WS, Appel LJ. Fish consumption, fish oil, omega-3 fatty acids, and cardiovascular disease. Circulation 2002; 106:2747-57.
13. NIH Office of Dietary Supplements, National Heart Lung and Blood Institute. Working Group Report on Future Clinical Research Directions on Omega-3 Fatty Acids and Cardiovascular Disease. June 2, 2004. Bethesda MD. Available at: http://www.nhlbi.nih.gov/meetings/workshops/omega-3/omega-3-rpt.htm Accessed on March 28, 2008.
14. Stipp D. Fish-oil doses can be hard to swallow. Wall Street Journal: January 8, 2008; Page D1-D2.

15. Gosnell M. Top 100 science stories of 2007: #8: Can vitamin D save your life? Discover: December 12, 2007. Available at http://discovermagazine.com/2008/jan/year-in-science-2007/can-vitamin-d-save-your-life Accessed March 25, 2008.
16. Guthrie C. The 10 biggest medical breakthroughs. #10: Benefits of vitamin D. Time: December 24, 2007. Available at: http://www.time.com/time/specials/2007/top10/article/0,30583,1686204_1686252_1690393,00.html Accessed March 25, 2008.
17. Harvard Health Letter. The top 10 health stories of 2006. Harv Health Lett 2006; 32:1-3.
18. Frisinger C. New year, new you: 8 simple rules for a healthier 2008. #1. Get more vitamin D. Fort Worth Star-Telegram (Texas): January 1, 2008; Page E1.
19. Brody JE. An oldie vies for nutrient of the decade. New York Times: February 19, 2008; Page F7.
20. Stampfer MJ. Vitamin D in the spotlight. Newsweek: December 11, 2006. Available at: http://www.newsweek.com/id/43998 Accessed March 25, 2008.
21. Peng T. Don't forget your vitamins. Newsweek: March 24, 2008; Page 78.
22. Rosenfeld I. We may need more vitamin D. Parade Magazine: January 29, 2006; Page 15.
23. Rhodes P. Vitamin D essentials: what you need to know about this important nutrient and where best to obtain it. Cooking Light 2008; 22:44-50.
24. Foreman J. Is there any way to tell whether you are getting enough vitamin D? Boston Globe: February 4, 2008; Page C2.
25. Harvard Health Letter. A more D-manding diet. Some experts say we should have a lot more vitamin D in our diets because it's protective against several diseases. Harv Health Lett 2007; 32:3.
26. Simopoulos AP, Robinson J. The Omega Diet: The Lifesaving Nutritional Program Based on the Diet of the Island of Crete. New York: HarperCollins, 1999.
27. Sears B. The Omega Rx Zone: The Miracle of the New High-Dose Fish Oil. New York: HarperCollins, 2004.
28. Kaufman M. Fish oil may aid against manic depression. Washington Post, 1999:Z07.
29. Foreman J. Fatty acid imbalance hurts our health. Boston Globe: February 10, 2004.
30. Bakalar N. Fish oil linked to lower Alzheimer's risk. New York Times: November 14, 2006.
31. Munoz SS. Shopping around: DHA-enhanced foods. Wall Street Journal: March 27, 2008; Page D2.
32. Canadian Cancer Society. Canadian Cancer Society Announces Vitamin D Recommendation. Available at: http://www.cancer.ca/ccs/internet/mediareleaselist/0,,3172_1613121606_1997621989_langId-en.html. Accessed May 6, 2008.
33. Vieth R, Bischoff-Ferrari H, Boucher BJ, Dawson-Hughes B, Garland CF, Heaney RP, Holick MF, Hollis BW, Lamberg-Allardt C, McGrath JJ, Norman AW, Scragg R, Whiting SJ, Willett WC, Zittermann A. The urgent need to recommend an intake of vitamin D that is effective. Am J Clin Nutr 2007; 85:649-50.
34. Davis CD, Dwyer JT. The "sunshine vitamin": benefits beyond bone? J Natl Cancer Inst 2007; 99:1563-5.
35. Bardia A, Tleyjeh IM, Cerhan JR, Sood AK, Limburg PJ, Erwin PJ, Montori VM. Efficacy of antioxidant supplementation in reducing primary cancer incidence and mortality: systematic review and meta-analysis. Mayo Clin Proc 2008; 83:23-34.
36. Mosca L, Banka CL, Benjamin EJ, Berra K, Bushnell C, Dolor RJ, Ganiats TG, Gomes AS, Gornik HL, Gracia C, Gulati M, Haan CK, Judelson DR, Keenan N, Kelepouris E, Michos ED, Newby LK, Oparil S, Ouyang P, Oz MC, Petitti D, Pinn VW, Redberg RF, Scott R, Sherif K, Smith SC, Jr., Sopko G, Steinhorn RH, Stone NJ, Taubert KA, Todd

BA, Urbina E, Wenger NK. Evidence-based guidelines for cardiovascular disease prevention in women: 2007 update. Circulation 2007; 115:1481-501.
37. Kris-Etherton PM, Lichtenstein AH, Howard BV, Steinberg D, Witztum JL. Antioxidant vitamin supplements and cardiovascular disease. Circulation 2004; 110:637-41.
38. Lee IM, Cook NR, Gaziano JM, Gordon D, Ridker PM, Manson JE, Hennekens CH, Buring JE. Vitamin E in the primary prevention of cardiovascular disease and cancer: the Women's Health Study: a randomized controlled trial. JAMA 2005; 294:56-65.
39. Miller ER, 3rd, Pastor-Barriuso R, Dalal D, Riemersma RA, Appel LJ, Guallar E. Meta-analysis: high-dosage vitamin E supplementation may increase all-cause mortality. Ann Intern Med 2005; 142:37-46.
40. Bjelakovic G, Nikolova D, Gluud LL, Simonetti RG, Gluud C. Mortality in randomized trials of antioxidant supplements for primary and secondary prevention: systematic review and meta-analysis. JAMA 2007; 297:842-57.
41. Yokoyama M, Origasa H, Matsuzaki M, Matsuzawa Y, Saito Y, Ishikawa Y, Oikawa S, Sasaki J, Hishida H, Itakura H, Kita T, Kitabatake A, Nakaya N, Sakata T, Shimada K, Shirato K. Effects of eicosapentaenoic acid on major coronary events in hypercholesterolaemic patients (JELIS): a randomised open-label, blinded endpoint analysis. Lancet 2007; 369:1090-8.
42. Gruppo Italiano per lo Studio della Sopravvivenza nell'Infarto miocardico. Dietary supplementation with n-3 polyunsaturated fatty acids and vitamin E after myocardial infarction: results of the GISSI-Prevenzione trial. Gruppo Italiano per lo Studio della Sopravvivenza nell'Infarto miocardico. Lancet 1999; 354:447-55.
43. Lappe JM, Travers-Gustafson D, Davies KM, Recker RR, Heaney RP. Vitamin D and calcium supplementation reduces cancer risk: results of a randomized trial. Am J Clin Nutr 2007; 85:1586-91.
44. Wactawski-Wende J, Kotchen JM, Anderson GL, Assaf AR, Brunner RL, O'Sullivan MJ, Margolis KL, Ockene JK, Phillips L, Pottern L, Prentice RL, Robbins J, Rohan TE, Sarto GE, Sharma S, Stefanick ML, Van Horn L, Wallace RB, Whitlock E, Bassford T, Beresford SA, Black HR, Bonds DE, Brzyski RG, Caan B, Chlebowski RT, Cochrane B, Garland C, Gass M, Hays J, Heiss G, Hendrix SL, Howard BV, Hsia J, Hubbell FA, Jackson RD, Johnson KC, Judd H, Kooperberg CL, Kuller LH, LaCroix AZ, Lane DS, Langer RD, Lasser NL, Lewis CE, Limacher MC, Manson JE. Calcium plus vitamin D supplementation and the risk of colorectal cancer. N Engl J Med 2006; 354:684-96.
45. Hathcock JN, Shao A, Vieth R, Heaney R. Risk assessment for vitamin D. Am J Clin Nutr 2007; 85:6-18.
46. Lang JM, Buring JE, Rosner B, Cook N, Hennekens CH. Estimating the effect of the run-in on the power of the Physicians' Health Study. Stat Med 1991; 10:1585-93.
47. Talwar SA, Aloia JF, Pollack S, Yeh JK. Dose response to vitamin D supplementation among postmenopausal African American women. Am J Clin Nutr 2007; 86:1657-62.
48. Prentice RL. A case-cohort design for epidemiologic cohort studies and disease prevention trials. Biometrika 1986; 73:1-11.
49. Rimm EB, Giovannucci EL, Stampfer MJ, Colditz GA, Litin LB, Willett WC. Reproducibility and validity of an expanded self-administered semiquantitative food frequency questionnaire among male health professionals. Am J Epidemiol 1992; 135:1114-26.
50. Feskanich D, Rimm EB, Giovannucci EL, Colditz GA, Stampfer MJ, Litin LB, Willett WC. Reproducibility and validity of food intake measurements from a semiquantitative food frequency questionnaire. J Am Diet Assoc 1993; 93:790-6.
51. Salvini S, Hunter DJ, Sampson L, Stampfer MJ, Colditz GA, Rosner B, Willett WC. Food-based validation of a dietary questionnaire: the effects of week-to-week variation in food consumption. Int J Epidemiol 1989; 18:858-67.

52. Jacques PF, Sulsky SI, Sadowski JA, Phillips JC, Rush D, Willett WC. Comparison of micronutrient intake measured by a dietary questionnaire and biochemical indicators of micronutrient status. Am J Clin Nutr 1993; 57:182-9.
53. Feskanich D, Willett WC, Colditz GA. Calcium, vitamin D, milk consumption, and hip fractures: a prospective study among postmenopausal women. Am J Clin Nutr 2003; 77:504-11.
54. Hunter DJ, Rimm EB, Sacks FM, Stampfer MJ, Colditz GA, Litin LB, Willett WC. Comparison of measures of fatty acid intake by subcutaneous fat aspirate, food frequency questionnaire, and diet records in a free-living population of US men. Am J Epidemiol 1992; 135:418-27.
55. Vieth R. Critique of the considerations for establishing the tolerable upper intake level for vitamin D: critical need for revision upwards. J Nutr 2006; 136:1117-22.
56. Bischoff-Ferrari HA, Giovannucci E, Willett WC, Dietrich T, Dawson-Hughes B. Estimation of optimal serum concentrations of 25-hydroxyvitamin D for multiple health outcomes. Am J Clin Nutr 2006; 84:18-28.
57. Dawson-Hughes B, Heaney RP, Holick MF, Lips P, Meunier PJ, Vieth R. Estimates of optimal vitamin D status. Osteoporos Int 2005; 16:713-6.
58. Giovannucci E, Liu Y, Rimm EB, Hollis BW, Fuchs CS, Stampfer MJ, Willett WC. Prospective study of predictors of vitamin D status and cancer incidence and mortality in men. J Natl Cancer Inst 2006; 98:451-9.
59. Aloia JF, Patel M, DiMaano R, Li-Ng M, Talwar SA, Mikhail M, Pollack S, Yeh JK. Vitamin D intake to attain a desired serum 25-hydroxyvitamin D concentration. Am J Clin Nutr 2008;87:1952-1958
60. Harris WS. International recommendations for consumption of long-chain omega-3 fatty acids. J Cardiovasc Med (Hagerstown) 2007; 8 Suppl 1:S50-2.
61. Lee JH, O'Keefe JH, Lavie CJ, Marchioli R, Harris WS. Omega-3 fatty acids for cardioprotection. Mayo Clin Proc 2008; 83:324-32. [Docket No. 86G-0289], 1997.
62. Agency for Healthcare Research and Quality. Effects of Omega-3 Fatty Acids on Cardiovascular Disease. Evidence Report/Technology Assessment Number 94. AHRQ Pub. No. 04-E009-1. 2004. Available at: http://www.ahrq.gov/clinic/epcsums/o3cardsum.htm Accessed March 28, 2008.
63. Simopoulos AP. Evolutionary aspects of diet, the omega-6/omega-3 ratio and genetic variation: nutritional implications for chronic diseases. Biomed Pharmacother 2006; 60:502-7.
64. Kris-Etherton PM, Taylor DS, Yu-Poth S, Huth P, Moriarty K, Fishell V, Hargrove RL, Zhao G, Etherton TD. Polyunsaturated fatty acids in the food chain in the United States. Am J Clin Nutr 2000; 71:179S-88S.
65. Harris WS. The omega-6/omega-3 ratio and cardiovascular disease risk: uses and abuses. Curr Atheroscler Rep 2006; 8:453-9.
66. Stanley JC, Elsom RL, Calder PC, Griffin BA, Harris WS, Jebb SA, Lovegrove JA, Moore CS, Riemersma RA, Sanders TA. UK Food Standards Agency Workshop Report: the effects of the dietary n-6:n-3 fatty acid ratio on cardiovascular health. Br J Nutr 2007; 98:1305-10.
67. Harris WS, Poston WC, Haddock CK. Tissue n-3 and n-6 fatty acids and risk for coronary heart disease events. Atherosclerosis 2007; 193:1-10.
68. Mozaffarian D, Ascherio A, Hu FB, Stampfer MJ, Willett WC, Siscovick DS, Rimm EB. Interplay between different polyunsaturated fatty acids and risk of coronary heart disease in men. Circulation 2005; 111:157-64.
69. Department of Health and Human Services, U.S. Food and Drug Administration. Substances affirmed as generally recognized as safe: menhaden oil. Federal Register. June 5, 1997. Vol 62, No 108: pp 30751-30757. 21 CFR Part 184 [Docket No. 86G-

0289], 1997.52. Jacques PF, Sulsky SI, Sadowski JA, Phillips JC, Rush D, Willett WC. Comparison of micronutrient intake measured by a dietary questionnaire and biochemical indicators of micronutrient status. Am J Clin Nutr 1993; 57:182-9.
70. Harris WS. Expert opinion: omega-3 fatty acids and bleeding--cause for concern? Am J Cardiol 2007; 99:44C-46C.
71. Bays HE. Safety considerations with omega-3 fatty acid therapy. Am J Cardiol 2007; 99:35C-43C.
72. Satterfield S, Greco PJ, Goldhaber SZ, Stampfer MJ, Swartz SL, Stein EA, Kaplan L, Hennekens CH. Biochemical markers of compliance in the Physicians' Health Study. Am J Prev Med 1990; 6:290-4.
73. Fritz AG, Percy C, Jack A, Shanmugaratham K, Sobin LH, Parkin MD, Whelan S. International Classification of Diseases for Oncology (ICD-O), Third Edition. Geneva: World Health Organization, 2000.
74. Thygesen K, Alpert JS, White HD, Joint ESC/ACCF/AHA/WHF Task Force for the Redefinition of Myocardial Infarction. Universal definition of myocardial infarction. Circulation 2007; 116:2634-53.
75. Adams HP, Jr., Bendixen BH, Kappelle LJ, Biller J, Love BB, Gordon DL, Marsh EE, 3rd. Classification of subtype of acute ischemic stroke. Definitions for use in a multicenter clinical trial. TOAST. Trial of Org 10172 in Acute Stroke Treatment. Stroke 1993; 24:35-41.
76. Cox DR. Regression models and life-tables. J Royal Statist Soc B 1972; 34:187-220.
77. Gooley TA, Leisenring W, Crowley J, Storer BE. Estimation of failure probabilities in the presence of competing risks: new representations of old estimators. Stat Med 1999; 18:695-706.
78. Putter H, Fiocco M, Geskus RB. Tutorial in biostatistics: competing risks and multi-state models. Stat Med 2007; 26:2389-430.
79. Fine JP, Gray RJ. A proportional hazards model for the subdistribution of a competing risk. J Am Stat Assoc 1999; 94:496-509.
80. Barlow WE. Robust variance estimation for the case-cohort design. Biometrics 1994; 50:1064-72.
81. Borgan O, Langholz B, Samuelsen SO, Goldstein L, Pogoda J. Exposure stratified case-cohort designs. Lifetime Data Anal 2000; 6:39-58.
82. Holick MF. Vitamin D for health and in chronic kidney disease. Semin Dial 2005; 18:266-75
83. Freedman LS. Tables of the number of patients required in clinical trials using the logrank test. Stat Med 1982; 1:121-9.
84. Haybittle JL. Repeated assessment of results in clinical trials of cancer treatment. Br J Radiol 1971; 44:793-7.
85. Peto R, Pike MC, Armitage P, Breslow NE, Cox DR, Howard SV, Mantel N, McPherson K, Peto J, Smith PG. Design and analysis of randomized clinical trials requiring prolonged observation of each patient. I. Introduction and design. Br J Cancer 1976; 34:585-612.
86. Geller NL, Pocock SJ. Interim analyses in randomized clinical trials: ramifications and guidelines for practitioners. Biometrics 1987; 43:213-23.
87. O'Brien PC, Fleming TR. A multiple testing procedure for clinical trials. Biometrics 1979; 35:549-56.
88. DeMets DL, Lan KK. Interim analysis: the alpha spending function approach. Stat Med 1994; 13:1341-52.
89. Pocock SJ. Current controversies in data monitoring for clinical trials. Clin Trials 2006; 3:513-21.

PROTOCOL FOR THE VITAL TRIAL November 30, 2017, version 23

SUMMARY

The VITAL trial (***VIT****amin D and Omeg**A**-3 Tria**L*) is a randomized, double-blind, placebo-controlled, 2x2 factorial trial of vitamin D (in the form of vitamin D_3 [cholecalciferol] softgel capsules) and marine omega-3 fatty acid (Omacor® eicosapentaenoic acid [EPA] + docosahexaenoic acid [DHA]) supplements in the primary prevention of cancer and cardiovascular disease (CVD). The trial will be conducted among 26,500 men aged ≥50 and women aged ≥55, to be recruited from mailings to 9.25 million persons, including health and other professionals, members of AARP (formerly known as the American Association of Retired Persons), and others. Willing and eligible respondents to the mailings will be enrolled in a 3-month run-in, during which they will receive placebos. Upon successful completion of the run-in, participants who remain willing and eligible will be randomly assigned to one of four treatment groups for 5 years: vitamin D_3 (2000 IU/d) and fish oil (1000 mg capsule containing Omacor® 840 mg EPA+DHA); vitamin D_3 and fish oil placebo; placebo vitamin D_3 and fish oil; and placebo vitamin D_3 and placebo fish oil. Blood samples will be collected and stored to allow assessment of effect modification by baseline 25-hydroxyvitamin D and omega-3 fatty acid levels, as well as future ancillary studies of genetic/biochemical hypotheses. At 1-year intervals, participants will receive a new supply of capsules and a follow-up questionnaire on compliance, possible side effects, and incidence of endpoints. Endpoints will be confirmed by medical record review.

I. BACKGROUND AND SIGNIFICANCE

The VITAL Trial will evaluate the role of vitamin D and long-chain marine omega-3 polyunsaturated fatty acid (Omacor® eicosapentaenoic acid [EPA] plus docosahexaenoic acid [DHA]) supplements in the primary prevention of cancer and cardiovascular disease (CVD). Although there have been marked advances in our understanding of potentially beneficial effects of vitamin D and omega-3 fatty acids on cancer and cardiovascular risk in recent years, clear gaps in knowledge remain.

Data from laboratory studies, epidemiologic investigations, and small clinical trials strongly suggest a protective effect for vitamin D against cancer, but there are no completed or ongoing large randomized trials of high-dose vitamin D supplements for the primary prevention of cancer in a general population.[1] At a 2007 conference on the role of vitamin D in cancer prevention sponsored by the National Cancer Institute (NCI) and the National Institutes of Health Office of Dietary Supplements (ODS), most speakers "expressed a need for intervention studies with comprehensive assessment of vitamin D status.[1] To quote one such investigator, Dr. Edward Giovannucci, "Further observational studies—and randomized trials if feasible—would be useful in testing the hypothesis that vitamin D lowers cancer risk. … Confirming that vitamin D reduces the risk of cancer incidence or mortality is critical, because current health recommendations typically do not encourage high intakes of vitamin D and tend to discourage sun exposure. Current dietary recommendations are geared only to prevent quite low vitamin D levels. If the association between better vitamin D status and reduced cancer risk is a causal one, the levels of intake currently recommended are probably inadequate. [*Note*: The 2011 Institute of Medicine guidelines call for 600 IU/d of vitamin D for adults aged 51-70 and 800 IU/d for adults aged >70 to maintain bone health and normal calcium metabolism.[2]] Defining … the optimal levels of vitamin D for cancer prevention remains a challenge, but further study should be a high priority because the potential for benefit is substantial."[3]

Available data on the connection between vitamin D and CVD are less extensive but also support a possible protective effect. It is thus concerning that >1/2 of U.S. middle-aged and older women and >1/3 of similarly aged men have vitamin D insufficiency.[4, 5] African-American (black) individuals are particularly vulnerable, because darkly pigmented skin is less able to synthesize vitamin D in response to solar radiation and because they tend to have lower intakes of dietary and supplemental vitamin D than whites.[6, 7] Obese individuals are also at above-average risk, presumably due to decreased bioavailability of this fat-soluble vitamin.[8, 9] Given the aging population and soaring obesity prevalence,[10, 11] low vitamin D status is an increasingly important public health issue.

Marine omega-3 fatty acids have shown considerable promise for the primary prevention of CVD in laboratory and observational studies; large randomized trials in secondary prevention or high-risk settings also find benefit. However, there are no completed or ongoing trials of such supplements for the primary prevention of CVD in a general population that has been selected only on the basis of age and *not* on vascular risk factors such as diabetes. The Nutrition Committee of the American Heart Association (AHA) has stated that “Additional studies are needed to confirm and further define the health benefits of omega-3 fatty acid supplements for … primary … prevention.”[12] (p. 2775) The conclusion from a 2004 workshop of the ODS and the National Heart, Lung and Blood Institute (NHLBI) Working Group on Future Clinical Research Directions on Omega-3 Fatty Acids and CVD[13] is that “… the body of evidence is consistent with the hypothesis that intake of omega-3 fatty acids reduces CVD but … a definitive trial is needed” and that “A primary prevention trial [would] fill a major gap in knowledge because virtually none of the trials to date have been conducted in a primary prevention population and thus … it is unknown whether omega-3 fatty acids can prevent first events.” In terms of methodology, the ODS/NHLBI Working Group recommended a large simple trial, with fish oil given in supplement rather than in food form. It is critically important that such a trial be undertaken in a timely fashion, to avoid a possible dilution of effect from fortification of the food supply with omega-3 fats, an increasingly common practice among U.S. manufacturers.[14]

It is important to clarify the roles of vitamin D and omega-3 fatty acids in the primary prevention of cancer and CVD because their purported health benefits are receiving increasing attention in both the medical literature and the popular press.[15-31] The Canadian Cancer Society recently raised its recommended dose of vitamin D to 1000 IU/d,[32] and many experts are urging a similar move in the U.S.[33] Sales of individual vitamin D supplements at U.S. grocery, drug, and “big box/club” stores jumped 91% to nearly $30 million during 2007 (C. Reider of Pharmavite LLC, personal communication). In 2007, sales of omega-3 fatty acid supplements in the U.S. reached $600 million, an increase of 20% from the prior year; omega-3 supplements are now the fifth-best-selling dietary supplement.[14] In addition, an increasing number of foods are omega-3 fortified; >1200 such products were launched in 2006 alone.[14] The growing enthusiasm for vitamin D and fish oil underscores the urgent need for a timely initiation of a large randomized trial to test these agents rigorously, before their use becomes so prevalent (through supplements and fortification of food) as to render participant recruitment and hypothesis testing impossible. As Dr. Cindy Davis of the NCI has noted, “While vitamin D may well have multiple benefits beyond bone, health professionals and the public should not in a rush to judgment assume that vitamin D is a magic bullet and consume high amounts of vitamin D. More definitive data on both benefits and potential adverse effects of high doses are urgently needed.”[34] The same can be said of marine omega-3 fatty acids; the conclusion from a 2004 workshop on omega-3 fatty acids and CVD sponsored by ODS and the NHLBI was that “a definitive trial is needed.”[13]

Indeed, skeptics will note that most single-agent nutritional interventions have not withstood the test of rigorous trials for the primary prevention of cancer and CVD—witness the disappointing findings for antioxidant vitamins.[35-40] However, unlike the antioxidant scenario, the two agents in this proposal appear promising not only in observational studies but also in randomized settings. Omega-3 fatty acids have shown cardiovascular benefit in two large trials in high-risk individuals[41, 42] (one of which also tested vitamin E and found no benefit for it[42]), while vitamin D was suggestive of cancer benefit in a modestly sized trial[43] and of reductions in total and cancer mortality even at a low dose in another.[44] The proposed large-scale randomized controlled trial of vitamin D and omega-3 fatty acids will provide definitive data on their roles in the primary prevention of cancer and CVD.

II. SPECIFIC AIMS

The overall aim of the VITAL trial is to evaluate the role of vitamin D softgel capsules and long-chain marine omega-3 polyunsaturated fatty acid (Omacor® eicosapentaenoic acid [EPA] plus docosahexaenoic acid [DHA]) supplements in the primary prevention of cancer and cardiovascular disease (CVD).

Primary aims:

1. To test whether vitamin D_3 supplementation reduces the risk of (a) total cancer (excluding non-melanoma skin cancer) and (b) major CVD events (a composite endpoint of myocardial infarction [MI], stroke, and cardiovascular mortality).
2. To test whether EPA+DHA supplementation reduces the risk of (a) total cancer (excluding non-melanoma skin cancer) and (b) major CVD events.

Secondary aims:

1. To test whether vitamin D_3 or EPA+DHA supplementation reduces the risk of (a) incident colorectal cancer, (b) incident breast cancer (in women), (c) incident prostate cancer (in men), and (d) total cancer mortality.
2. To test whether vitamin D_3 or EPA+DHA supplementation reduces the risk of (a) an expanded composite cardiovascular endpoint of MI, stroke, cardiovascular mortality, and coronary revascularization (i.e., coronary artery bypass grafting [CABG] or percutaneous coronary intervention [PCI]) and (b) the individual components of the primary cardiovascular endpoint, particularly total CVD mortality.

Tertiary aims:

1. To explore whether vitamin D_3 and EPA+DHA supplementation exhibit synergistic or additive effects on the risk of (a) total cancer, (b) major CVD events, and (c) the secondary endpoints specified above.
2. To explore whether the effect of vitamin D_3 or EPA+DHA supplementation on cancer and CVD risk varies by (a) baseline blood levels of these nutrients, (b) race/skin pigmentation (for vitamin D_3), and (c) body mass index (BMI) (for vitamin D_3).
3. To explore whether vitamin D_3 and EPA+DHA supplementation reduces the risks of heart failure.

III. SUBJECT SELECTION

Study population: VITAL will be conducted among 26,500 apparently healthy participants. Included in the trial will be men aged ≥50 and women aged ≥55, ages at which rates of chronic disease increase substantially. Excluded from the study will be individuals who have (1) a history of cancer (except non-melanoma skin cancer), MI, stroke, TIA, CABG, or PCI; (2) have any of the following safety exclusions: use of anticoagulants at baseline, history of renal failure or dialysis, hypercalcemia, hypo- or hyperparathyroidism, severe liver disease (cirrhosis), or sarcoidosis or other granulomatous diseases such as active chronic tuberculosis or Wegener's granulomatosis; (3) have an allergy to fish (for EPA+DHA); (4) have another serious illness that would preclude participation; (5) are consuming greater than 800 IU of vitamin D from all supplemental sources combined (individual vitamin D supplements, vitamin D analogues, calcium+vitamin D supplements, medications with vitamin D [e.g., Fosamax Plus D], and multivitamins), and are not willing to decrease or forego such use during the trial (to ensure total supplemental vitamin D intake does not exceed the current safety limit [i.e., tolerable upper intake level of 4000 IU/d set by the Institute of Medicine[2]); (6) are consuming greater than 1200 mg/d of calcium (the RDA for men aged >70 years and women aged >50 years[2]) from all supplemental sources combined; (7) are taking fish oil supplements and are not willing to forego their use during the trial; and (8) are not willing to participate, as evidenced by not signing the informed consent form. Although thiazide diuretics and supplemental vitamin D may interact to increase hypercalcemia risk, it is unlikely that such an interaction would occur at the vitamin D dose we propose.[45] Thus, thiazide use will not be an exclusion criterion.

Cohort assembly: A master mailing tape containing 9.25 million names and addresses will be assembled by Listmart, a list broker with access to all commercially available U.S. mailing lists. The listings will include licensed health professionals (e.g., physicians, nurses, pharmacists, veterinarians, physical therapists), other professionals (e.g., executives, managers, financial service professionals, computer professionals, architects, engineers, accountants), AARP members, and subscribers to magazines that have minority readership or appeal to professionals and college-educated individuals (e.g., *Essence, National Geographic* and *Time*). Additionally, it will include individuals who have indicated interest in participating through the study website (www.vitaltrial.org; see paragraph below), publicized via advertisements and feature articles in "healthy-lifestyle" publications, as well as individuals who are currently participating in our other observational epidemiologic studies (e.g., Women's Health Study). Of the 9.25 million names on the mailing tape, an estimated 30% or more will be black or Hispanic. Each invited individual will be assigned a study identification number and mailed an invitation letter and screening questionnaire.

In addition, we will place advertisements and feature articles in magazines that have minority readership and in "healthy-lifestyle" publications, as well as setting up a booth at health fairs and distributing fliers on the study. The advertisement/flier would explain the objectives of the trial and the general eligibility criteria. Interested individuals would be encouraged to visit a study web site at www.vitaltrial.org where they could review in greater detail the purpose of the trial, what participation involves, and the eligibility criteria. By submitting their name, address and age at the web site, individuals would be assigned a study identification number and mailed the VITAL invitation letter and a screening questionnaire. If necessary, we will also consider inviting eligible participants from groups with large minority representation, such as church groups.

IV. SUBJECT ENROLLMENT

Screening : The W.A. Wilde Company, a direct marketer in Holliston, MA that we have used in our previous trials, will mail screening materials by non-profit bulk rate to each individual in the

assembled cohort. The mailings will occur in groupings of approximately ≥300,000 each, spaced at monthly intervals. The mailing package will include:

- A letter explaining the rationale for VITAL. The letter will outline what participation would entail. We will also send a targeted letter to individuals identified as African-American with a high degree of certainty (e.g., *Essence* subscribers). The special invitation letter would emphasize that African-Americans are at higher risk for vitamin D deficiency, as well as heart disease, stroke, and certain cancers.
- A very brief screening questionnaire with items on demographics (age, gender, race/ethnicity, education); medical history (cancer, MI, stroke, CABG and PCI); and current use of supplements containing vitamin D or fish oil.
- A self-addressed, pre-paid envelope for returning study forms.

If the initial response rates are lower than anticipated, a second enrollment request will be mailed to selected non-respondents, who will be identified from mailing groups that had high response and eligibility rates.

Eligible at screening : A computer program will evaluate the screening questionnaire data to classify respondents' eligibility to receive the baseline questionnaire and informed consent form as ineligible, eligibility pending, or eligible. Respondents with eligibility pending will be sent a follow-up form or called to clarify incomplete or inconsistent responses so that they can be classified as ineligible or eligible. Respondents will be eligible to receive the baseline questionnaire and informed consent form if they meet these criteria: (1) are age ≥50 (men) or ≥55 (women) years; (2) have no history of cancer (except non-melanoma skin cancer), MI, stroke, CABG, or PCI; (3) are consuming no more than 800 IU of vitamin D from all supplemental sources combined (individual vitamin D supplements, vitamin D analogues, calcium+vitamin D supplements, medications with vitamin D [e.g., Fosamax Plus D], and multivitamins), or, if taking, willing to decrease or forego such use during the trial (to ensure total supplemental vitamin D intake does not exceed the current safety limit [i.e., tolerable upper intake level of 4000 IU/d set by the Institute of Medicine[2]); (4) are not taking fish oil supplements, or, if taking, willing to forego their use during the trial.

We estimate that approximately 160,000 respondents will be potentially eligible for participation in the VITAL trial based upon their responses on the screening questionnaire. Respondents who are not eligible will be mailed a letter thanking them for their interest and informing them of the general criteria that determined eligibility.

Enrollment: At periodic intervals, the names/addresses of recent respondents, who are eligible to receive the baseline questionnaire, will be electronically compiled in preparation for the mailing of the baseline questionnaire and informed consent form. It is anticipated that there will be 5-10,000 entries per bi-weekly list. W.A. Wilde Company will mail these materials by non-profit bulk rate to each:

- A letter of appreciation for the initial response which also reiterates the purpose of the study and what participation involves.
- An informed consent form.
- A baseline questionnaire with items on demographics (date of birth); medical history (kidney stones, hypercalcemia, kidney failure, sarcoidosis, liver disease, thyroid disease, chronic tuberculosis, other major illnesses); allergy to fish; current use of calcium supplements and other supplements containing vitamin D or fish oil; height and weight;

use of anticoagulant medications; and potential effect modifiers such as skin pigmentation and sunlight exposure. Respondents will also be asked for telephone number(s) and an e-mail address in the event that follow-up needs to be conducted in order to clarify questionnaire responses and to provide their social security number (optional) which will be used for identification purposes only. In addition, the form will contain a question asking whether individuals would be willing to return a blood sample if mailed a convenient blood collection kit.

- A self-addressed, pre-paid envelope for returning study forms.

Eligibility: A computer program will evaluate the questionnaire data to classify respondents' eligibility as ineligible (to enter the run-in), eligibility pending, or eligible. The "eligibility pending" respondents will be sent a follow-up form to clarify incomplete or inconsistent responses. Respondents will be eligible if they meet these criteria: (1) have none of the following safety exclusions: use of anticoagulants, history of renal failure or dialysis, hypercalcemia, hypo- or hyperparathyroidism, severe liver disease (cirrhosis), or sarcoidosis or other granulomatous diseases such as active chronic tuberculosis or Wegener's granulomatosis; (2) have no allergy to fish (for EPA+DHA) or fish oil supplements; (3) have no other serious illness that would preclude participation; (4) are consuming no more than 800 IU of vitamin D from all supplemental sources combined (individual vitamin D supplements, vitamin D analogues, calcium+vitamin D supplements, medications with vitamin D [e.g., Fosamax Plus D], and multivitamins), or, if taking, willing to decrease or forego such use during the trial (to ensure total supplemental vitamin D intake does not exceed the current safety limit [i.e., tolerable upper intake level of 4000 IU/d set by the Institute of Medicine[2]); (5) are consuming no more than 1200 mg/d of calcium (the RDA for men aged >70 years and women aged >50 years[2]) from all supplemental sources combined; (6) are willing to participate, as evidenced by signing the informed consent form.

We estimate that approximately 40,000 individuals will be both willing and fully eligible to participate in the trial and enter the run-in. Respondents who are not eligible will be mailed a letter thanking them for their interest and informing them of the general criteria that determined eligibility.

Run-in: The experience of other studies conducted by our research group (PHS, WHS, and WAFACS) has demonstrated the success of a run-in period in identifying a group of excellent compliers for long-term follow-up. Because the health experience of participants will be analyzed according to randomized treatment assignment, the use of a run-in phase to eliminate poor compliers prior to randomization will increase the power of the study.[46] During a 3-month run-in period, all participants will take placebo vitamin D softgel capsules and placebo fish oil. As the effects of both agents on cancer prevention are likely to be chronic, it would not be scientifically appropriate to use active agent in the run-in and then randomize to placebo. Moreover, the use of placebos will allow us to most clearly analyze the side effects of the study agents during the randomized treatment period.

At trial enrollment, willing and eligible participants will be mailed a 6-month supply of run-in calendar packs (i.e., 3 months for the actual run-in and 3 months to allow pill-taking to continue uninterrupted while we check participants' responses for continued eligibility and then ship randomized capsules to participants who remain eligible). Participants will take two capsules per day (one vitamin D placebo softgel capsule and one fish oil placebo capsule). At 3 months, participants will receive a follow-up run-in questionnaire asking about continued eligibility,

willingness to continue in the trial, pill compliance, and potential side effects. Non-responders will be mailed up to 3 requests at 6-week intervals.

Participants will be randomized only if they: (1) demonstrate good compliance in pill taking, defined as taking at least two-thirds of the study pills during the run-in (i.e., missed 10 or fewer days of pill taking in the past month); (2) express willingness to continue in the trial; (3) report no new history of cancer (except non-melanoma skin cancer), MI, stroke, TIA, CABG, PCI, hypercalcemia, sarcoidosis, kidney failure or dialysis, parathyroid disease, cirrhosis or other severe liver disease, or other serious illness, use of anticoagulants or vitamin D analogues, during the run-in; (4) are consuming no more than 1200 mg/d of calcium (the RDA for men aged >70 years and women aged >50 years[2]) from all supplemental sources combined; and (5) demonstrate continued willingness to limit supplemental vitamin D intake to ≤800 IU/d and to forego the use of fish oil supplements.

Because we are close to reaching our original target sample of 20,000 (now revised to 26,500) participants randomized, but have not yet reached our target of 25% African-American participants, we will modify the compliance criterion to be more strict for white, non-Hispanic participants (starting October 2012) as follows: White, non-Hispanic participants who have not provided a blood sample will be randomized only if they demonstrate good compliance in pill taking, defined as taking at least 83% of study pills during the run-in (i.e., missed 5 or fewer days of pill taking in the past month). For white, non-Hispanic participants living in the catchment area for clinic visits (approved under protocol 2010P002331), the original compliance criterion (taking at least two-thirds of the study pills during the run-in pills) will still apply if they are willing to participate in a clinic visit.

We estimate that of the 40,000 initially willing and eligible individuals enrolled in the run-in, 66% (n=26,500) will be compliant and remain willing and eligible for randomization.

Randomization: Willing and eligible participants will be randomly assigned to one of four treatment groups: vitamin D_3 softgel capsules (2000 IU/d) and fish oil (Omacor® EPA+DHA, 840 mg/d); vitamin D_3 softgel capsules and fish oil placebo; placebo vitamin D_3 softgel capsules and Omacor® fish oil; and placebo vitamin D_3 softgel capsules and placebo fish oil. Individuals will be randomized to treatment within 5-year age groups using a computer-generated table of random numbers. Within each age group, treatment assignments will be generated in blocks of eight individuals, with two individuals in each of the four treatment combinations. The date of randomized treatment assignment will be the formal "in-study" date. (The use of age stratification during randomization will increase statistical efficiency and power.) Participants will be mailed an annual supply of capsules (in calendar packs) and instructed to take two capsules – one vitamin D softgel capsule or placebo and one of Omacor® fish oil or placebo – each day.

V. STUDY PROCEDURES

A. BLOOD COLLECTION

Baseline: Participants who indicate on their baseline questionnaire a willingness to provide an optional venous blood sample will be sent a blood collection kit during the run-in. Participants who indicate on the questionnaire that they are "not sure" about participating or unwilling to participate in the blood collection will be mailed a letter informing them that we can arrange to have their blood drawn at home by a technician from a national medical service company, if they are now willing. The blood collection kit will include a consent form, supplies and

instructions for having the blood drawn into two EDTA tubes, one heparin tube, and one serum tube, a gel-filled freezer pack, and an overnight courier air bill. We anticipate that most participants will have their blood drawn by their own healthcare providers or in another healthcare or blood-drawing facility. For those not able to do this on their own, VITAL staff will contact participants to schedule a visit to a blood-drawing facility, or to schedule a service for drawing blood from the participant at home. Participants will be instructed to provide a fasting sample, if possible, and to record the time of venipuncture and the time of their last meal. They will be instructed to send their sample to our blood laboratory via overnight courier in the freezer packs on the same day as the blood draw. Upon receipt, the samples will be centrifuged to separate plasma, red blood cells, and buffy coat; aliquoted into 2-ml Nunc vials (including multiple vials of EDTA heparin and serum plasma vials, and vials for EDTA red blood cells and buffy coat, and stored in individual vial locations in nitrogen freezers(-170 ºC). The process will be completed within several hours of specimen receipt to ensure that samples are frozen within 30-36 hours after venipuncture. Each freezer has an electronic alarm which is monitored 24 hours a day. If problems arise, staff members are paged by the alarm company and can adjust the levels of liquid nitrogen as needed. This system has protected stored samples from inadvertent thawing or warming for nearly 20 years.

The main reason for collecting pre-randomization blood specimens is to provide the opportunity to assess whether treatment effects are modified by baseline blood levels of 25(OH)D and EPA+DHA. When stratifying by 25(OH)D, season (assessed by date of return) and geographic location (assessed by zip code) will be considered. This resource will also be extremely valuable for future applications for funding of ancillary studies to explore other genetic and biochemical hypotheses in this well-characterized cohort. A nested case-control or case-cohort approach can be used, enabling the study of many promising biochemical and genetic markers (e.g., VDR polymorphisms) as potential predictors of disease risk and modifiers of intervention effects at very low incremental cost.

Follow-up: An optional follow-up blood sample will be collected in year 2 and year 4 from a subset of 6000 participants who provided a baseline fasting blood sample. These follow-up samples, to be collected using the same methods as for the baseline samples, will allow assessment of (a) pill-taking compliance, (b) changes in biomarkers with treatment, and (c) in the placebo group, the effect of changing trends in background fortification with vitamin D and omega-3 fats. The samples will be particularly important for an exploratory analysis of treatment-induced changes in 25(OH)D levels among black participants, as there are few data on this topic.[47] In addition, changes in blood calcium and parathyroid hormone (PTH) levels will be examined as markers of possible hypercalcemia, a potential side effect of high vitamin D intake. The bloods will also be archived for future ancillary studies to evaluate whether treatment with one or both of the study agents leads to changes in biochemical factors, including those in the lipid, glucose tolerance, inflammation, endothelial dysfunction, thrombosis, insulin, and insulin-like growth factor pathways.

Biochemical assays: Blood levels of 25(OH)D and EPA+DHA will be assayed on participants who provide an analyzable sample at baseline and all participants who provide a blood sample at follow-up. In addition, baseline and follow-up blood levels of calcium, PTH and lipoproteins will be assayed. These assays will be performed by Quest Diagnostics. Quest Diagnostics will also perform albumin, creatinine, liver function and lipid tests on a subset of participants. In addition, we will measure several biomarkers related to CVD (including cholesterol lipoprotein subfractions using the Vertical Auto Profile cholesterol test, apolipoproteins, thyroid function, vitamin D, albumin, creatinine, PCSK9 and a basic metabolic panel). These additional

measurements will be done at Atherotec Diagnostics Lab in Birmingham, AL, which will fund the costs of these studies. In addition, PCSK (plasma-based genotype will be assayed on participants who additionally gave genetic consent.

We will also measure metabolomic and lipidomic biomarkers on baseline and follow-up stored samples. Samples will be sent in a de-identified manner and measurements will be done at Dr. Mohit Jain's laboratory at the University of California San Diego, and at LabCorp, Inc., Raleigh, NC. In a small subset of participants, we will also 1) measure cholesterol efflux and epigenetic biomarkers on participants who gave genetic consent by sending de-identified samples to our academic collaborators in Quebec, Dr. Benoit Arsenault and Dr. Luigi Bouchard (Institut universitaire de cardiologie et de pneumologie de Québec); and 2) measure additional vitamin D biomarkers on de-identified samples sent to our academic collaborators at Massachusetts General Hospital, Dr. Masao Kaneki and Dr. Sadeq Quraishi.

Over 5 years of follow-up, we would expect to accrue approximately 1660 cancer cases and 990 CVD cases, with somewhat smaller numbers if the agents are highly effective. If 60% of participants provide blood specimens, this would include about 996 cancer cases and 594 CVD cases with bloods.

B. ASSESSMENT OF DIETARY AND SUPPLEMENTAL INTAKE

A modified version of a validated, self-administered semi-quantitative food frequency questionnaire (FFQ) will be mailed to participants during the run-in. The original questionnaire, developed by Walter Willett, MD, DrPH, and colleagues, is an efficient, reliable, and accurate instrument for categorizing individuals according to their intake of 32 nutrients, including vitamin D and marine omega-3 fats.[48-53] Participants will be asked to estimate their average intake over the past year of various foods, beverages, and supplements that contain vitamin D, marine omega-3 fats, and other nutrients. Dietary vitamin D intake will be estimated from participants' reported intakes of certain foods, including dairy products, fortified breakfast cereals, fortified juices, dark-meat fish, and cod liver oil. Dietary marine omega-3 fat intake will be estimated from intake of dark-meat fish, canned tuna, other fish, and seafood. Classifying participants by baseline intake of various nutrients will allow us to evaluate whether the study agents' effects vary by such intake. Participants will also be asked to complete the FFQ at 4+ years, enabling an examination of dietary changes over time.

Questions on use of non-study supplements or drugs containing vitamin D or EPA+DHA will be asked at baseline, 6 months, and on yearly follow-up questionnaires. We will also assess flaxseed and alpha-linolenic acid intakes. We will ascertain and analyze intake from food and supplements separately, and from both sources combined.

C. TREATMENT

In our research application, we proposed testing 1600 IU (40 µg) per day of vitamin D_3 (cholecalciferol) in VITAL. Since the application was submitted, based on new research and with approval from NCI, we now will test 2000 IU (50 µg) per day instead. We seek to obtain a large-enough difference in vitamin D status between the treatment and placebo groups to detect health benefits, and there appear to be few safety issues associated with this dose. Recent studies have found little toxicity risk with doses up to 10,000 IU/d.[45] (Nevertheless, to further

ensure participants' safety, we will exclude from the trial persons with a history of hypercalcemia, or sarcoidosis ; follow-up calcium and parathyroid hormone [PTH] levels will be obtained in a random subsample of participants.) Accumulating evidence suggests that vitamin D intakes well above currently recommended amounts of 600 IU/d for adults aged 51-70 and 800 IU/d for adults aged >70[2] are necessary for health benefits. In a review of studies of serum 25(OH)D in relation to bone mineral density, lower-extremity function, dental health, and risk of falls, fractures, and colorectal cancer, Bischoff-Ferrari et al.[54] found that, for all endpoints, advantageous 25(OH)D levels began at 75 nmol/L, and optimal levels were between 90-100 nmol/L. The average older individual requires an oral vitamin D_3 intake of at least 800-1000 IU/d (20-25 µg) to achieve a serum 25(OH)D of 75 nmol/L.[55] Among postmenopausal women in the WHI, 400 IU/d of vitamin D_3 raised median plasma 25(OH)D from 42.3 to only 54.1 nmol/L.[44, 56]

The primary reason for the dose change is a recent study by Aloia et al[57] which indicates that the dose-response for serum 25(OH)D with vitamin D intake is not linear, but that the rate of increase in serum levels decreases at higher levels of intake. At a dose of 800 IU (20 µg/day), the rate of change is 1.2 nmol/L. However, at doses ≥1,400 IU (35 µg/day), the rate of change decreases to 0.7 nmol/L. Thus, extrapolation of these data suggests that with the originally proposed 1600 IU/d (40 µg/day), the expected change in mean serum 25(OH)D levels would be only 28 nmol/L, or a change from an estimated 42 nmol/L at baseline to a final mean level of 70 nmol/L. With 2000 IU/d (50 µg/day), the increase would be 35 nmol/L from baseline to a final mean level 77 nmol/L, which would be above the 75 nmol/L threshold level at which health benefits are believed to occur [54] , as well as a level believed to be free of any risks.

Potential side effects of vitamin D are rare. They include gastrointestinal (GI) upset (presence or absence of symptoms of peptic ulcer, nausea, constipation, diarrhea); and physician diagnosis of hypercalcemia or kidney stones, both of which are not expected to occur at the dose used in VITAL.[2] To minimize the risks of hypercalcemia and kidney stones, we will require that participants limit their total intake of calcium from all sources, including multivitamins, single supplements of calcium, and other drugs that contain calcium to 1200 mg or less a day.

Active vitamin D_3 (2000 IU [50 µg] per softgel capsule) and matching inert placebo will be provided by Pharmavite LLC.

In our research application, we proposed testing a total EPA+DHA dose of 1 mg/d (EPA [500 mg/d] + DHA [500 mg/d]). Since the application was submitted, we have worked with various drug companies to develop soft-gel capsules containing these doses. We now propose to test a total dose of Omacor® EPA+DHA, 840 mg/d instead, since this is the highest dose of EPA + DHA currently available in a 1000 mg capsule.

We believe that this dose provides the best balance of feasibility, efficacy and safety. We seek to obtain a large-enough difference in omega-3 fatty acid status between the treatment and placebo groups to detect health benefits, and there appear to be few safety issues associated with this dose. Health authorities recommend 400 mg to 1 g/d for cardioprotection.[58] A total dose of 850 mg/d was used in the GISSI-Prevenzione Trial (EPA to DHA ratio, 1:2) and AREDS 2 (EPA to DHA ratio, 2:1), while a dose of 1.8 g/d of EPA was used in JELIS. For this trial, we have selected a total dose of omega-3 fatty acids recommended by the American Heart Association for cardioprotection and demonstrated to be beneficial in one secondary prevention population with minimal side effects.[42] Because the optimal ratio of EPA to DHA is unknown,[59,60] we have selected a 40:30 ratio of EPA to DHA. The ratio of omega-3 to omega-6 fatty acid intake is between 1:10 and 1:20 in most Western countries, including the U.S., whereas the

optimal ratio for disease prevention has been hypothesized to be closer to 1:1 or 1:2,[61,62] although this is controversial.[63] Indeed, there is growing consensus that the absolute intake of omega-3 is a more important predictor of health than is the ratio of omega-3 to omega-6 intake, at least for cardiovascular outcomes.[63-66] However, given that the average intake of EPA+DHA is 100-200 mg/d among U.S. adults,[58] the proposed intervention of 840 mg/d would be expected to increase the average participant's omega-3 intake by a factor of 4 to 8. Assuming no concurrent change in omega-6 intake, the proposed omega-3 dose would thus have the effect of achieving the purported optimal omega-3 to omega-6 ratio and providing intakes associated with benefits in previous studies.

As with vitamin D, potential side effects of omega-3 fatty acids are rare. They include gastrointestinal (GI) symptoms (burping, stomach upset or pain, nausea, constipation, diarrhea), bleeding (any GI bleed, GI bleeding requiring transfusion, hematuria, easy bruising, epistaxis), skin rash, colds or upper respiratory tract infection, flu-like symptoms, and bad taste in mouth. The FDA has concluded that marine omega-3 fatty acid doses of up to 3 g/d are "Generally Recognized as Safe."[67] The AHA also has concluded that these risks are very low or low at doses of up to 1 g/d and low to moderate at doses of 1-3 g/d.[12] Because they undergo an extensive purification process, high-quality fish oil supplements are free of environmental toxins (e.g., methylmercury, polychlorinated biphenyls [PCBs], and dioxins) found in some fish.[12, 68]

Active fish oil (1000 mg capsule containing Omacor® 840 mg EPA + DHA) and matching inert placebo will be provided by Pronova BioPharma ASA of Norway.

D. FOLLOW-UP

With regard to follow-up, at 6 months, participants will be mailed a very brief follow-up questionnaire and at each anniversary date a more detailed questionnaire and a re-supply of calendar packs. The questionnaire will include items on compliance with randomized treatments, use of non-trial supplements of vitamin D and fish oil, use of calcium supplements, development of major illnesses, dietary intakes of vitamin D and fish, and updated risk factors related to cancer and CVD. Information on potential side effects of the study agents will be elicited for monitoring by the Data and Safety Monitoring Board. For fish oil, these side effects include GI symptoms (burping, stomach upset or pain, nausea, constipation, diarrhea), bleeding (any GI bleed, GI bleeding, hematuria, easy bruising, epistaxis), skin rash, colds or upper respiratory tract infection, flu-like symptoms, and bad taste in mouth. For vitamin D, these side effects include GI symptoms, and physician diagnosis of hypercalcemia or kidney stones, both of which are not expected to occur at the dose used in VITAL.[2] Non-responders will be mailed a second and third request and will then be telephoned to collect study data. If phone contact fails, a fourth request will be mailed in order to ascertain the participant's study status and to obtain minimum information regarding the primary study outcomes. Participants will also be given the option of completing an electronic questionnaire sent via e-mail. These data will be collected and managed using REDCap electronic data capture tools. REDCap (Research Electronic Data Capture) is a secure, HIPAA-compliant web-based application hosted by PartnersHealthCare Research Computing.

Internet sources will be used to assist in the tracking (new address, new phone number) of participants who are potentially lost to follow-up. At the very least, vital status will be ascertained. Participants will be instructed to discontinue their study capsules if, during follow-up, they receive a diagnosis of hypercalcemia, sarcoidosis, or other safety-exclusion conditions and to inform their physicians about their participation in VITAL. If, in the course of follow-up,

participants report taking supplementary vitamin D (not from diet) that amounts to greater than 800 IU per day or daily calcium supplements that amount to greater than 1200 mg per day, they will be asked to limit their intakes to study guideline levels. These instructions will be included in the study guidelines which accompany each shipment of calendar packs.

At 6-month intervals between annual follow-ups, participants will be mailed an interim form with questions on the development of primary endpoints (cancer, MI, and stroke), difficulties with pill compliance, and address changes. Participants will be asked to return the form only if one or more questions apply. The interim follow-up will help the staff maintain accurate contact information and provide a head start in addressing compliance issues and timely collection of medical records to confirm endpoints.

A newsletter will be mailed to participants twice each year. The newsletter will keep participants up-to-date regarding the progress of the study and also touch upon health topics that are related to the study such as prevention of cardiovascular disease and cancer or current research findings.

At the end of pill-taking, for the following two years, an annual questionnaire will be mailed to participants. The form will be similar to the conventional follow-up questionnaires, collecting information about potential risk factors for cancer and cardiovascular disease, use of medications, and medical history. The additional data will be used to address the main research questions and to examine new hypotheses regarding health promotion and disease prevention.

E. ENDPOINT ASCERTAINMENT AND VALIDATION

The primary cancer endpoint of VITAL will be total cancer incidence (excluding non-melanoma skin cancer), with cancer mortality and the individual sites of colorectal, breast, and prostate cancer as secondary endpoints. For CVD, the primary endpoint will be a composite endpoint of MI, stroke, and CVD mortality. Secondary endpoints will include a second composite endpoint adding revascularization procedures of CABG and PCI, as well as the individual endpoints of MI, stroke, revascularization, and CVD mortality. Additional endpoints of interest include heart failure, colorectal polyps, and periodontal disease.

Participants who self-report one of the above study endpoints will be asked to sign a medical release form authorizing VITAL staff to obtain hospital/physician records. The request will be accompanied by a cover letter expressing sympathy for the diagnosis and explaining HIPAA guidelines and the scientific importance of record validation. Non-responders will be sent two additional requests for the medical release form. After the release is obtained, a copy will be sent to the treating hospital/physician. If there is no response within 1 month, a second request will be mailed, followed by a phone call.

Beginning in the fall 2016, to increase the completeness of outcome information regarding MI, stroke, revascularization procedures of CABG and PCI, and cancer, information will be requested from Medicare (the Centers for Medicare and Medicaid Services). To obtain the information, participants will be identified using date of birth and social security number (if previously provided by the participant). The information will include medical diagnoses from hospitalizations, emergency room visits, as well as the names of procedures funded by Medicare. The information does not include test result or actual medical records. To obtain this

specific information, participants will be contacted directly in order to gain their written permission for the release of this information (see above.)

After all records are obtained, an Endpoints Committee of physicians, blinded to the randomized treatment assignment, will review the file and, using a defined protocol, will confirm or disconfirm the case. A cancer diagnosis will be confirmed with histologic or cytologic evidence. In the absence of these diagnostic tests, strong clinical evidence accompanied by radiologic evidence or laboratory markers (e.g., PSA levels) will be used to confirm cancer occurrence. The histologic type, grade, and stage of cancer will be recorded.[70] MI will be confirmed using Joint European Society of Cardiology/American College of Cardiology Foundation/ American Heart Association/World Heart Federation (ESC/ACCF/AHA/WHF) Task Force for the Redefinition of Myocardial Infarction criteria.[71] Stroke will be confirmed and categorized according to Trial of Org 10172 in Acute Stroke Treatment (TOAST) criteria.[72] Death due to a cardiovascular cause will be confirmed by convincing evidence of a CVD event from all available sources, including death certificates, hospital records, autopsy, and observer accounts (for deaths outside the hospital).

For reported deaths, the validation process will be similar. A condolence letter will be mailed to the family, requesting permission to obtain medical records and a copy of the death certificate. If the family does not provide the death certificate, a copy will be requested from the state vital records bureau where the participant died. The Endpoints Committee will review all records relevant to the death and assign an ICD code. At the end of the trial, we will search the National Death Index Plus (NDI-Plus) data base for known deaths for cases where we could not obtain records. NDI-Plus provides an ICD-coded cause of death based on death certificate information. We will also use the NDI to search for participants who become lost to follow-up.

F. DATA MANAGEMENT

Because participants will be followed solely by mail, our computing system is a critical feature of effective follow-up. This system, which was developed and fine-tuned in our previous trials, will track each participant’s stage in the study and level of participation. It will automatically generate letters, questionnaires, and phone call reminders at the appropriate times. Names, addresses, telephone numbers, participation status, and processing information will be kept up to date, and data from questionnaires, letters, and phone calls will be entered into the study database. When talking to a participant, study personnel will need ready access to identifying information, participation level, and the content of previous study-related telephone calls. However, it is also critical that these data be available only to authorized staff members. Our existing computer and security systems, which balance these considerations, will be used in VITAL.

Questionnaire data will be optically scanned into the computer. The relevant software—TELEform and Alchemy (Cardiff Software)—has been successfully used for several years in the WHS and PHS. Out-of-range, internally inconsistent, and unclear data will be reviewed by a verifier who will edit misread variables. Forms that cannot be scanned, and name and address changes, will be entered using traditional double-entry procedures. All data will undergo additional within-form and across-time checks to verify accuracy. The database will be maintained on a UNIX server. All data files will be backed up nightly, ensuring at least two current copies at all times. Each month, a set of data files will be taken off-site for long-term storage.

Beginning in 2014, some study data will be collected via electronic forms sent to study participants via e-mail. The data will be collected and managed using REDCap electronic data capture tools. REDCap (Research Electronic Data Capture) is a free, secure, HIPAA-compliant web-based application hosted by Partners HealthCare Research Computing, Enterprise Research Infrastructure & Services (ERIS) group. Vanderbilt University with collaboration from a consortium of academic and non-profit institutional partners has developed this software toolset and workflow methodology for electronic collection and management of research study data. Our research team develops the data collection tools with planning assistance from Harvard Catalyst / The Harvard Clinical and Transitional Science Center EDC Support Staff. The development and testing process results in a well-planned data coillection strategy. REDCAp provides flexible features that can be used for a variety of research projects and provides an intuitive interface to enter data with real-time validation (automated data type and range checks). The system offers easy data manipulation with audit trails, reports for monitoring and querying participant records, and an automated export mechanism to common statistical packages (e.g., SAS).

Participants' names and contact information will be accessible only to staff members who need the information for their jobs. Endpoint and health-related questionnaire data will be stored in separate files from the processing data and will be accessible only to approved investigators and programmers. In these files, participants are identified only by study ID.

VI. BIOSTATISTICAL ANALYSIS

A. DATA ANALYSIS

Analyses of treatment effects will be based on the intent-to-treat principle. The first analysis will compare baseline characteristics by randomized treatment assignment to ensure that balance was achieved by the randomization. Characteristics to be examined include known risk factors for cancer and CVD, including age; gender; race/ethnicity; BMI; smoking; alcohol use; physical activity; medical conditions such as hypertension, hyperlipidemia, diabetes, and family history of cancer and CVD; and baseline vitamin D and omega-3 fatty acid levels as assessed by dietary questionnaire in all participants and blood assays in a case-cohort subsample. The large sample size, as well as successful balance of known potential confounders, will provide assurance that unmeasured or unknown potential confounders will also be equally distributed across randomized treatment groups.

In this 2x2 factorial design, the primary aim is to compare the main effects of intention-to-treat with vitamin D softgel capsules and with Omacor® fish oil on cancer and CVD. We will use the Cox proportional hazards model to allow for variable follow-up lengths[73] and will estimate the hazard ratio for each intervention using indicators for treatment exposure, controlling for the second intervention, age, and gender. Because the cohort will consist of older individuals, competing risks due to deaths from other causes will be considered. The primary analysis will estimate the cause-specific hazard and the hazard ratio comparing intervention groups for each outcome of interest by censoring individuals with deaths due to competing causes. To estimate the cumulative incidence function, the subdistribution of each endpoint will be plotted over time.[74,75] The alternative Fine and Gray approach[76] models the effect of treatment on the subdistribution hazard or directly on the cumulative incidence function. While we will consider this and compare its fit to the model-free cumulative incidence curves,[75] the proportional hazards approach will be our primary analysis.

Similar analyses will be conducted for both cancer and CVD. For cancer, we will estimate the intervention effects on the primary endpoint of total cancer, as well as the secondary outcomes of breast, prostate, and colorectal cancer, and cancer mortality. For CVD we will estimate the effects on the primary composite endpoint of major CVD (MI, stroke, and cardiovascular mortality); on the secondary composite endpoint that additionally includes coronary revascularization; and on the individual components of MI, stroke, revascularization, and cardiovascular mortality.

Beyond the primary analyses, we will conduct prespecified secondary analyses to examine effect modification by the other randomized intervention, by baseline risk factors, by compliance, and by time. We have a particular interest in exploring interactions between the interventions, as well as with the baseline plasma biomarkers of 25(OH)D for vitamin D and of EPA+DHA for fish oil. We hypothesize that the intervention effects may be larger among those with below-median baseline levels, and will examine treatment effects by quartiles of these biomarkers. The biomarker data will be analyzed as a case-cohort study using methods for proportional hazards regression.[77] Because of the frequency matching by age and gender, appropriate stratum-specific weighting of the observations will be employed.[78] We also have a prior interest in the effects of the vitamin D intervention within groups defined by race/ethnicity and skin pigmentation, and by BMI. In addition, we will evaluate effect modification by age and gender, as well as by sunlight exposure, calcium and phosphorus intakes from the FFQ (as these nutrients affect vitamin D bioavailability[79]), and baseline risk factors for cancer and CVD. The latter interaction effects will be interpreted cautiously, as hypothesis generating. We will also examine as a prespecified secondary analysis the association among compliers, in which participants' follow-up time will be censored when they are no longer compliant with taking at least two-thirds of their assigned active agents or placebos. Finally, we will examine whether treatment effects vary over time and duration of treatment by examining survival plots and interactions with time. We will additionally conduct analyses for the primary endpoints of cancer and cardiovascular disease excluding events occurring in Year 1, as well as in Years 1 and 2, of follow-up. There may be a latent effect on both outcomes, depending on the mechanisms by which these agents act.

We will also compare the incidence of potential side effects in the active *v.* placebo groups for each agent, including the incidence of kidney stones with vitamin D assignment (cf. WHI calcium-vitamin D findings[44]) and the incidence of GI symptoms, skin abnormalities, and bleeding with fish oil assignment (cf. JELIS findings[41]).

B. POWER CALCULATIONS

To calculate power, the following assumptions were made: (1) a 2x2 factorial trial in 12,500 men aged ≥50 and 12,500 women aged ≥55; (2) independent and equal allocation of participants to each treatment; (3) an age distribution based on that observed at baseline in our past trials for men aged ≥50 and women aged ≥55; (4) age-specific event rates based on the observed rates in the first 5 years of the PHS II for men and the WHS for women; (5) trial follow-up of 5 years, with little loss to follow-up as achieved in our past trials; and (6) compliance similar to that in published trials upon which our estimated rate ratio (RR) reductions are based. The cited reductions are thus the observed effects we would see in the trial. The corresponding 'true' RR is given assuming an average compliance of 80%. Power is given for a two-sided test using a logrank analysis[80] with a significance level of 0.05.

Cancer: Assuming that only one agent is effective, with 5 years of treatment and follow-up we will have 93% power to detect an observed RR of 0.85 for the primary endpoint of total cancer incidence (Table 1). For prostate cancer, we will have 93% power to detect a 25% risk reduction. For breast cancer, we will have 86% power to detect a 35% risk reduction. For colorectal cancer, we will have 77% power to detect a 35% risk reduction and 88% power to detect a 40% risk reduction. For cancer mortality, we will have 86% power to detect a 30% risk reduction. If both agents are effective in preventing cancer but act independently, power would be reduced slightly due to a somewhat smaller overall number of events. If the agents interact, however, power will be affected to the extent of the interaction. Should the agents act synergistically, power would increase, as illustrated in Table 2 for the outcome of total cancer.

Table 1. Power for effects of a single agent on cancer in VITAL, a 2x2 factorial trial of 12,500 men aged≥50 and 12,500 women aged≥55, with 5 years of follow-up

Observed RR[a]	True RR[b]	Total Cancer	Cancer Mortality	Colorectal Cancer	Breast Cancer (Women)	Prostate Cancer (Men)
0.90	0.875	61.4	-	-	-	-
0.85	0.812	92.7	-	-	-	-
0.80	0.750	99.6	50.5	31.7	38.7	77.0
0.75	0.687	>99.9	70.6	46.8	56.3	92.8
0.70	0.625	>99.9	86.1	62.9	73.2	98.6
0.65	0.560	>99.9	95.0	77.3	86.3	99.8
0.60	0.500	>99.9	98.7	88.2	94.3	>99.9

a. Observed RR=intent-to-treat RR, including noncompliant participants. Compliance is assumed to be 80%.
b. True RR=that with perfect compliance.
RR, rate ratio.

For example, if the effect of each agent alone is a reduction of 10%, but in combination the effect is stronger, with an additional 10% decrease, the RR comparing the combined group to the all placebo group would be 0.73, versus 0.81 with additive effects (on the multiplicative scale). Power for the main effect of each agent would then increase to 88% (or higher with greater synergy). The trial would have power to detect moderate-to-large interactions, as shown in Table 2. Should the agents interact in a subadditive fashion, power would be reduced

Table 2. Power for effects of a single agent on CVD in VITAL, a 2x2 factorial trial of 12,500 men aged ≥50 and 12,500 women aged ≥55, with 5 years of follow-up

Observed RR[a]	True RR[b]	Major CVD[c]	Total CVD[d]	CVD Mortality	MI	Stroke
0.90	0.875	41.6	62.2	-	-	-
0.85	0.812	75.6	93.2	-	-	-
0.80	0.750	94.8	99.6	53.7	60.1	60.3
0.75	0.687	99.5	>99.9	74.0	80.2	80.3
0.70	0.625	>99.9	>99.9	88.7	92.8	92.8
0.65	0.560	>99.9	>99.9	96.3	98.2	98.2
0.60	0.500	>99.9	>99.9	99.2	99.7	99.7

a. Observed RR=intent-to-treat RR, including noncompliant participants. Compliance is assumed to be 80%.
b. True RR=that with perfect compliance.
c. Major CVD=myocardial infarction, stroke, and CVD mortality
d. Total CVD=myocardial infarction, stroke, CVD mortality, and coronary revascularization (coronary artery bypass grafting or percutaneous coronary intervention)
CVD, cardiovascular disease; *MI*, myocardial infarction; *RR*, rate ratio.

to the extent of the subadditivity. Power to explore effect modification by baseline blood levels of 25(OH)D and EPA+DHA among those above and below the median level in the cohort will be lower because we anticipate that about 60% of participants will provide blood specimens.

Power has been shown for a given observed reduction in risk, assuming compliance similar to that seen in current randomized trials such as the WHS and PHS. For total cancer, our power will be excellent with a 5-year follow-up even if compliance is lower.

CVD: As shown in Table 3, assuming that only one agent is effective, with 5 years of treatment and follow-up we will have 95% power to detect an observed RR of 0.80 for the primary composite endpoint of MI, stroke, and cardiovascular mortality. For the secondary composite endpoint of MI, stroke, cardiovascular mortality, and coronary revascularization, we will have over 99% power to detect a 20% reduction and 93% power to detect a 15% reduction. With a RR of 0.70 we will have 89% power for cardiovascular mortality, and we will have over 80% power to detect an RR of 0.75 for MI and stroke. If both agents are effective in preventing CVD but act independently, power would be reduced only slightly due to a smaller overall number of events. If the agents act synergistically, power would increase, as described for cancer. If the agents interact in subadditive fashion, power would be reduced to the extent of the subadditivity.

Table 3. Power for interaction effects on total cancer in VITAL, a 2x2 factorial trial of 12,500 men aged ≥50 and 12,500 women aged ≥55, with 5 years of follow-up

RR Single agent[a]	Interaction[b]	RR Both agents	Power Main Effect	Power Interaction
0.90	1.0	0.81	59.1	
	0.9	0.73	88.4	
	0.8	0.65	98.5	60.7
	0.7	0.57	99.9	93.6
0.85	1.0	0.72	90.6	
	0.9	0.65	98.6	
	0.8	0.58	99.9	57.8
	0.7	0.51	>99.9	92.0
0.80	1.0	0.64	99.2	
	0.9	0.58	99.9	
	0.8	0.51	>99.9	54.7
	0.7	0.45	>99.9	89.9

a. RR = intent-to-treat RR, including noncompliant participants (assuming 80% compliance). Represents the effect among those not assigned to the other intervention and assumes the same effect for both agents.
b. The interaction is the RR for the combined group divided by the product of risks for the two separate groups—i.e., RRint=RRboth/(RR vitamin D alone*RR omega-3 fatty acids alone). An interaction=1 implies additive effects (no interaction).
RR, rate ratio.

VII. RISKS AND DISCOMFORTS

The VITAL trial will test a dose of 2000 IU (50 µg) per day of vitamin D_3 (cholecalciferol) softgel capsules and 840 mg/d of Omacor® EPA+DHA in VITAL. These doses provide the best balance of efficacy and safety, based on careful review of the literature and extensive consultation with numerous nutritional experts. We seek to obtain an adequate difference in vitamin D and EPA+DHA levels between the treatment and placebo groups to detect health benefits, and there appear to be few safety issues associated with these doses. For vitamin D, the selected dose is below the tolerable upper intake level (UL) for vitamin D_3 (100 µg, or 4000 IU) set by the Food and Nutrition Board of the Institute of Medicine (IOM). Additionally, because we will exclude from the trial persons who report supplemental vitamin D intakes >800 IU/d, the selected dose ensures that no participants will be taking a supplemental vitamin D dose above 2800 IU, which is well below the 4000 IU tolerable upper intake level specified by the Institute of Medicine.[2] To further ensure participants' safety, we will exclude from the trial persons with a history of hypercalcemia, renal failure, cirrhosis, or sarcoidosis or other granulomatous disease, and will obtain calcium and PTH levels in a random subsample of participants. The dose of

Omacor® fish oil used in this trial (840 mg/day) is consistent with guidelines set by health authorities who recommend up to 1 gram/day for cardio-protection. For fish oil, there are no specific exclusions necessary except for those currently taking the supplement and unwilling to forego use during the trial.

Potential side effects of the study agents will be assessed on each follow-up questionnaire. For fish oil, these side effects include gastrointestinal (GI) symptoms (burping, stomach upset or pain, nausea, constipation, diarrhea), bleeding (any GI bleed, GI bleeding requiring transfusion, hematuria, easy bruising, epistaxis), skin rash, colds or upper respiratory tract infection, flu-like symptoms, and bad taste in mouth. A recent study reported that men with high blood levels of omega-3 fatty acids, which are found in fish and fish oil supplements, were more likely to develop prostate cancer than men with low blood levels of omega-3 fatty acids.[81] However, because this was an observational study and not a randomized trial, the findings could be spurious, resulting from confounding bias. The VITAL trial will be collecting information on new diagnoses of prostate cancer, assessed on each follow-up questionnaire by self-report and confirmed on medical record review. For vitamin D, side effects include GI symptoms; and physician diagnosis of hypercalcemia or kidney stones, both of which are not expected to occur at the dose used in VITAL.[2]

There is also the possibility of social/psychological risk that could result from inadvertent disclosure of confidential information from the questionnaires or blood tests. However, we have many safeguards in place to avoid this possibility, and we have never had an inadvertent breach of confidentiality in any of our trials.

With regard to the risks of the study agents, information on potential side effects discussed above will be elicited on each follow-up questionnaire for monitoring by the Data and Safety Monitoring Board. We will compare the incidence of potential side effects in the active *v.* placebo groups for each agent, including the incidence of kidney stones with vitamin D assignment and the incidence of GI symptoms, skin abnormalities, and bleeding with fish oil assignment.

The potential risk of disclosure of confidential information is guarded against by maintaining completed questionnaires and blood test results in locked offices accessible by authorized personnel only. In questionnaire data files, participants are identified only by study ID. Subject identifiers are stored in separate computer files with password protection, and results will be presented only in the aggregate. In addition, employees involved with the proposed study will be asked to sign a form agreeing not to disclose any information to which they might have access, regardless of their personal perception about its confidentiality. Each employee of the study who has access to study data or has contact with subjects participates in an institutional (Brigham and Women's Hospital) human subjects educational program, which consists of reviewing regulatory and informational documents pertaining to human subjects research; passing a test on ethical principles and regulations governing human subjects research; and signing a statement of commitment to the protection of human subjects. Finally, all such employees are required to participate in HIPAA training

VIII. POTENTIAL BENEFITS

For the majority of participants, there will be few direct benefits from participating in this primary prevention study other than the awareness of being involved in a large endeavor to answer relevant and timely questions regarding the possible benefit of vitamin D and fish oil. During the

trial itself, we will receive inquiries from the participants regarding both specific and general health concerns. We will respond to each of those questions, primarily directing participants to published sources or recommending that they see their local health provider who is familiar with their medical history.

The purported health benefits of vitamin D and marine omega-3 fatty acids are receiving increasing attention in the medical literature and the popular press. Sales of vitamin D supplements and omega-3 fatty acid supplements at U.S. stores have increased substantially in recent years. However, definitive data on health benefits and risks of these agents are lacking. Findings from this large clinical trial will clarify the role of vitamin D and marine omega-3 fatty acid supplements in the primary prevention of cancer and CVD in men and women and will help guide individual decisions, clinical recommendations, and public health guidelines.

IX. MONITORING AND QUALITY ASSURANCE

A. SAFETY MONITORING

An independent Data and Safety Monitoring Board (DSMB) will be assembled, consisting of experts in clinical trials, epidemiology, biostatistics, relevant clinical areas of cancer and CVD, and NIH representatives. The Physicians' Health Study, the Women's Health Study, and the Women's Antioxidant and Folic Acid Cardiovascular Study have been monitored by the same DSMB since their inception. As two of these trials have ended and the third is in its last years, we have asked our DSMB members if they would be willing to also monitor VITAL. Four have accepted the invitation, and three new members have been added. The full 7-member DSMB has been approved by the NCI.

The DSMB will annually examine the progress of the trial and the unblinded data on study endpoints and possible adverse effects to recommend continuation, alteration of study design, or early termination, as appropriate. Interim trial results will be assessed with the Haybittle-Peto rule,[82,83] adjusting for multiple looks. In this method, interim results are compared to a z-score of 3 standard deviations (p=0.0027) throughout the trial. The final results may then be interpreted as having close to nominal significance levels. This rule appropriately requires very strong evidence for early stopping, is more conservative than the Pocock[84] and O'Brien-Fleming[85] rules and the alpha-spending function,[86] and can be conducted at convenient times without inducing statistical complexity.[87]

While these rules are intended for the primary endpoints, the goal of VITAL is to assess the overall balance of benefits and risks of the two agents in the primary prevention of cancer and CVD. Thus, consideration will also be given to the secondary endpoints that are needed in the interpretation of overall results. In addition, the monitoring rules will serve solely as guidelines in decisions regarding continuation or stopping of treatment arms. All decisions must be made after examining the totality of evidence, including other trial data, on these agents.

B. MONITORING OF SOURCE DATA AND DRUG DISPENSEMENT

Redundancies will be built into the data processing systems to insure accurate recording of data and proper follow-up. All research forms will be scanned in and the data read by a character-recognition software program (Teleform). Out-of-range, internally inconsistent, and unclear data will be reviewed by a verifier who will edit misread variables. Forms that cannot be scanned, and name and address changes, will be entered using traditional double-entry procedures. All

data will undergo additional within-form and across-time checks to verify accuracy. Following data entry, all questionnaire responses that require additional follow-up for missing data, participant comments on the form or for endpoint validation will be manually reviewed to insure correct processing and accurate follow-up. Address changes received from the post office or from participants will be manually keyed by data entry personnel and the resultant files compared and verified.

Great care will be taken to insure that participants receive their randomly assigned drugs. Upon receipt of drug shipments from the manufacturer, each of the four drug groups will be stored in segregated areas. A random sample of calendar packs will be pulled from each drug group and blindly tested to insure that the packaged contents match the study label. At the time of packaging for shipment to individual participants, listings will be divided according to drug group assignment and only one group will be packaged at a time. The packaging area will be cleaned and cleared prior to packaging and shipment of the next drug group. If drug packages are returned by the postal service as undeliverable or declined by a participant, the contents of the package will be blindly tested as an additional level of quality control.

X. REFERENCES

1. Davis CD, Hartmuller V, Freedman DM, Hartge P, Picciano MF, Swanson CA, Milner JA. Vitamin D and cancer: current dilemmas and future needs. Nutr Rev 2007; 65:S71-4.
2. Institute of Medicine. Dietary Reference Intakes for Calcium and Vitamin D. Washington DC: National Academies Press, 2011.
3. Giovannucci E. Strengths and limitations of current epidemiologic studies: vitamin D as a modifier of colon and prostate cancer risk. Nutr Rev 2007; 65:S77-9.
4. Looker AC, Dawson-Hughes B, Calvo MS, Gunter EW, Sahyoun NR. Serum 25-hydroxyvitamin D status of adolescents and adults in two seasonal subpopulations from NHANES III. Bone 2002; 30:771-7.
5. Holick MF. Vitamin D deficiency. N Engl J Med 2007; 357:266-81.
6. Harris SS. Vitamin D and African Americans. J Nutr 2006; 136:1126-9.
7. Moore CE, Murphy MM, Holick MF. Vitamin D intakes by children and adults in the United States differ among ethnic groups. J Nutr 2005; 135:2478-85.
8. Martins D, Wolf M, Pan D, Zadshir A, Tareen N, Thadhani R, Felsenfeld A, Levine B, Mehrotra R, Norris K. Prevalence of cardiovascular risk factors and the serum levels of 25-hydroxyvitamin D in the United States: data from the Third National Health and Nutrition Examination Survey. Arch Intern Med 2007; 167:1159-65.
9. Harris SS, Dawson-Hughes B. Reduced sun exposure does not explain the inverse association of 25-hydroxyvitamin D with percent body fat in older adults. J Clin Endocrinol Metab 2007; 92:3155-7.
10. Hedley AA, Ogden CL, Johnson CL, Carroll MD, Curtin LR, Flegal KM. Prevalence of overweight and obesity among US children, adolescents, and adults, 1999-2002. JAMA 2004; 291:2847-50.
11. Ogden CL, Carroll MD, Curtin LR, McDowell MA, Tabak CJ, Flegal KM. Prevalence of overweight and obesity in the United States, 1999-2004. JAMA 2006; 295:1549-55.
12. Kris-Etherton PM, Harris WS, Appel LJ. Fish consumption, fish oil, omega-3 fatty acids, and cardiovascular disease. Circulation 2002; 106:2747-57.
13. NIH Office of Dietary Supplements, National Heart Lung and Blood Institute. Working Group Report on Future Clinical Research Directions on Omega-3 Fatty Acids and Cardiovascular Disease. June 2, 2004. Bethesda MD. Available at:

http://www.nhlbi.nih.gov/meetings/workshops/omega-3/omega-3-rpt.htm Accessed on March 28, 2008.

14. Stipp D. Fish-oil doses can be hard to swallow. Wall Street Journal: January 8, 2008; Page D1-D2.
15. Gosnell M. Top 100 science stories of 2007: #8: Can vitamin D save your life? Discover: December 12, 2007. Available at http://discovermagazine.com/2008/jan/year-in-science-2007/can-vitamin-d-save-your-life Accessed March 25, 2008.
16. Guthrie C. The 10 biggest medical breakthroughs. #10: Benefits of vitamin D. Time: December 24, 2007. Available at: http://www.time.com/time/specials/2007/top10/article/0,30583,1686204_1686252_1690393,00.html Accessed March 25, 2008.
17. Harvard Health Letter. The top 10 health stories of 2006. Harv Health Lett 2006; 32:1-3.
18. Frisinger C. New year, new you: 8 simple rules for a healthier 2008. #1. Get more vitamin D. Fort Worth Star-Telegram (Texas): January 1, 2008; Page E1.
19. Brody JE. An oldie vies for nutrient of the decade. New York Times: February 19, 2008; Page F7.
20. Stampfer MJ. Vitamin D in the spotlight. Newsweek: December 11, 2006. Available at: http://www.newsweek.com/id/43998 Accessed March 25, 2008.
21. Peng T. Don't forget your vitamins. Newsweek: March 24, 2008; Page 78.
22. Rosenfeld I. We may need more vitamin D. Parade Magazine: January 29, 2006; Page 15.
23. Rhodes P. Vitamin D essentials: what you need to know about this important nutrient and where best to obtain it. Cooking Light 2008; 22:44-50.
24. Foreman J. Is there any way to tell whether you are getting enough vitamin D? Boston Globe: February 4, 2008; Page C2.
25. Harvard Health Letter. A more D-manding diet. Some experts say we should have a lot more vitamin D in our diets because it's protective against several diseases. Harv Health Lett 2007; 32:3.
26. Simopoulos AP, Robinson J. The Omega Diet: The Lifesaving Nutritional Program Based on the Diet of the Island of Crete. New York: HarperCollins, 1999.
27. Sears B. The Omega Rx Zone: The Miracle of the New High-Dose Fish Oil. New York: HarperCollins, 2004.
28. Kaufman M. Fish oil may aid against manic depression. Washington Post, 1999:Z07.
29. Foreman J. Fatty acid imbalance hurts our health. Boston Globe: February 10, 2004.
30. Bakalar N. Fish oil linked to lower Alzheimer's risk. New York Times: November 14, 2006.
31. Munoz SS. Shopping around: DHA-enhanced foods. Wall Street Journal: March 27, 2008; Page D2.
32. Canadian Cancer Society. Canadian Cancer Society Announces Vitamin D Recommendation. Available at: http://www.cancer.ca/ccs/internet/mediareleaselist/0,,3172_1613121606_1997621989_langId-en.html. Accessed May 6, 2008.
33. Vieth R, Bischoff-Ferrari H, Boucher BJ, Dawson-Hughes B, Garland CF, Heaney RP, Holick MF, Hollis BW, Lamberg-Allardt C, McGrath JJ, Norman AW, Scragg R, Whiting SJ, Willett WC, Zittermann A. The urgent need to recommend an intake of vitamin D that is effective. Am J Clin Nutr 2007; 85:649-50.
34. Davis CD, Dwyer JT. The "sunshine vitamin": benefits beyond bone? J Natl Cancer Inst 2007; 99:1563-5.
35. Bardia A, Tleyjeh IM, Cerhan JR, Sood AK, Limburg PJ, Erwin PJ, Montori VM. Efficacy of antioxidant supplementation in reducing primary cancer incidence and mortality: systematic review and meta-analysis. Mayo Clin Proc 2008; 83:23-34.

36. Mosca L, Banka CL, Benjamin EJ, Berra K, Bushnell C, Dolor RJ, Ganiats TG, Gomes AS, Gornik HL, Gracia C, Gulati M, Haan CK, Judelson DR, Keenan N, Kelepouris E, Michos ED, Newby LK, Oparil S, Ouyang P, Oz MC, Petitti D, Pinn VW, Redberg RF, Scott R, Sherif K, Smith SC, Jr., Sopko G, Steinhorn RH, Stone NJ, Taubert KA, Todd BA, Urbina E, Wenger NK. Evidence-based guidelines for cardiovascular disease prevention in women: 2007 update. Circulation 2007; 115:1481-501.
37. Kris-Etherton PM, Lichtenstein AH, Howard BV, Steinberg D, Witztum JL. Antioxidant vitamin supplements and cardiovascular disease. Circulation 2004; 110:637-41.
38. Lee IM, Cook NR, Gaziano JM, Gordon D, Ridker PM, Manson JE, Hennekens CH, Buring JE. Vitamin E in the primary prevention of cardiovascular disease and cancer: the Women's Health Study: a randomized controlled trial. JAMA 2005; 294:56-65.
39. Miller ER, 3rd, Pastor-Barriuso R, Dalal D, Riemersma RA, Appel LJ, Guallar E. Meta-analysis: high-dosage vitamin E supplementation may increase all-cause mortality. Ann Intern Med 2005; 142:37-46.
40. Bjelakovic G, Nikolova D, Gluud LL, Simonetti RG, Gluud C. Mortality in randomized trials of antioxidant supplements for primary and secondary prevention: systematic review and meta-analysis. JAMA 2007; 297:842-57.
41. Yokoyama M, Origasa H, Matsuzaki M, Matsuzawa Y, Saito Y, Ishikawa Y, Oikawa S, Sasaki J, Hishida H, Itakura H, Kita T, Kitabatake A, Nakaya N, Sakata T, Shimada K, Shirato K. Effects of eicosapentaenoic acid on major coronary events in hypercholesterolaemic patients (JELIS): a randomised open-label, blinded endpoint analysis. Lancet 2007; 369:1090-8.
42. Gruppo Italiano per lo Studio della Sopravvivenza nell'Infarto miocardico. Dietary supplementation with n-3 polyunsaturated fatty acids and vitamin E after myocardial infarction: results of the GISSI-Prevenzione trial. Gruppo Italiano per lo Studio della Sopravvivenza nell'Infarto miocardico. Lancet 1999; 354:447-55.
43. Lappe JM, Travers-Gustafson D, Davies KM, Recker RR, Heaney RP. Vitamin D and calcium supplementation reduces cancer risk: results of a randomized trial. Am J Clin Nutr 2007; 85:1586-91.
44. Wactawski-Wende J, Kotchen JM, Anderson GL, Assaf AR, Brunner RL, O'Sullivan MJ, Margolis KL, Ockene JK, Phillips L, Pottern L, Prentice RL, Robbins J, Rohan TE, Sarto GE, Sharma S, Stefanick ML, Van Horn L, Wallace RB, Whitlock E, Bassford T, Beresford SA, Black HR, Bonds DE, Brzyski RG, Caan B, Chlebowski RT, Cochrane B, Garland C, Gass M, Hays J, Heiss G, Hendrix SL, Howard BV, Hsia J, Hubbell FA, Jackson RD, Johnson KC, Judd H, Kooperberg CL, Kuller LH, LaCroix AZ, Lane DS, Langer RD, Lasser NL, Lewis CE, Limacher MC, Manson JE. Calcium plus vitamin D supplementation and the risk of colorectal cancer. N Engl J Med 2006; 354:684-96.
45. Hathcock JN, Shao A, Vieth R, Heaney R. Risk assessment for vitamin D. Am J Clin Nutr 2007; 85:6-18.
46. Lang JM, Buring JE, Rosner B, Cook N, Hennekens CH. Estimating the effect of the run-in on the power of the Physicians' Health Study. Stat Med 1991; 10:1585-93.
47. Talwar SA, Aloia JF, Pollack S, Yeh JK. Dose response to vitamin D supplementation among postmenopausal African American women. Am J Clin Nutr 2007; 86:1657-62.
48. Rimm EB, Giovannucci EL, Stampfer MJ, Colditz GA, Litin LB, Willett WC. Reproducibility and validity of an expanded self-administered semiquantitative food frequency questionnaire among male health professionals. Am J Epidemiol 1992; 135:1114-26.
49. Feskanich D, Rimm EB, Giovannucci EL, Colditz GA, Stampfer MJ, Litin LB, Willett WC. Reproducibility and validity of food intake measurements from a semiquantitative food frequency questionnaire. J Am Diet Assoc 1993; 93:790-6.

50. Salvini S, Hunter DJ, Sampson L, Stampfer MJ, Colditz GA, Rosner B, Willett WC. Food-based validation of a dietary questionnaire: the effects of week-to-week variation in food consumption. Int J Epidemiol 1989; 18:858-67.
51. Jacques PF, Sulsky SI, Sadowski JA, Phillips JC, Rush D, Willett WC. Comparison of micronutrient intake measured by a dietary questionnaire and biochemical indicators of micronutrient status. Am J Clin Nutr 1993; 57:182-9.
52. Feskanich D, Willett WC, Colditz GA. Calcium, vitamin D, milk consumption, and hip fractures: a prospective study among postmenopausal women. Am J Clin Nutr 2003; 77:504-11.
53. Hunter DJ, Rimm EB, Sacks FM, Stampfer MJ, Colditz GA, Litin LB, Willett WC. Comparison of measures of fatty acid intake by subcutaneous fat aspirate, food frequency questionnaire, and diet records in a free-living population of US men. Am J Epidemiol 1992; 135:418-27.
54. Bischoff-Ferrari HA, Giovannucci E, Willett WC, Dietrich T, Dawson-Hughes B. Estimation of optimal serum concentrations of 25-hydroxyvitamin D for multiple health outcomes. Am J Clin Nutr 2006; 84:18-28.
55. Dawson-Hughes B, Heaney RP, Holick MF, Lips P, Meunier PJ, Vieth R. Estimates of optimal vitamin D status. Osteoporos Int 2005; 16:713-6.
56. Giovannucci E, Liu Y, Rimm EB, Hollis BW, Fuchs CS, Stampfer MJ, Willett WC. Prospective study of predictors of vitamin D status and cancer incidence and mortality in men. J Natl Cancer Inst 2006; 98:451-9.
57. Aloia JF, Patel M, DiMaano R, Li-Ng M, Talwar SA, Mikhail M, Pollack S, Yeh JK. Vitamin D intake to attain a desired serum 25-hydroxyvitamin D concentration. Am J Clin Nutr 2008;87:1952-1958
58. Harris WS. International recommendations for consumption of long-chain omega-3 fatty acids. J Cardiovasc Med (Hagerstown) 2007; 8 Suppl 1:S50-2.
59. Lee JH, O'Keefe JH, Lavie CJ, Marchioli R, Harris WS. Omega-3 fatty acids for cardioprotection. Mayo Clin Proc 2008; 83:324-32. [Docket No. 86G-0289], 1997.
60. Agency for Healthcare Research and Quality. Effects of Omega-3 Fatty Acids on Cardiovascular Disease. Evidence Report/Technology Assessment Number 94. AHRQ Pub. No. 04-E009-1. 2004. Available at: http://www.ahrq.gov/clinic/epcsums/o3cardsum.htm Accessed March 28, 2008.
61. Simopoulos AP. Evolutionary aspects of diet, the omega-6/omega-3 ratio and genetic variation: nutritional implications for chronic diseases. Biomed Pharmacother 2006; 60:502-7.
62. Kris-Etherton PM, Taylor DS, Yu-Poth S, Huth P, Moriarty K, Fishell V, Hargrove RL, Zhao G, Etherton TD. Polyunsaturated fatty acids in the food chain in the United States. Am J Clin Nutr 2000; 71:179S-88S.
63. Harris WS. The omega-6/omega-3 ratio and cardiovascular disease risk: uses and abuses. Curr Atheroscler Rep 2006; 8:453-9.
64. Stanley JC, Elsom RL, Calder PC, Griffin BA, Harris WS, Jebb SA, Lovegrove JA, Moore CS, Riemersma RA, Sanders TA. UK Food Standards Agency Workshop Report: the effects of the dietary n-6:n-3 fatty acid ratio on cardiovascular health. Br J Nutr 2007; 98:1305-10.
65. Harris WS, Poston WC, Haddock CK. Tissue n-3 and n-6 fatty acids and risk for coronary heart disease events. Atherosclerosis 2007; 193:1-10.
66. Mozaffarian D, Ascherio A, Hu FB, Stampfer MJ, Willett WC, Siscovick DS, Rimm EB. Interplay between different polyunsaturated fatty acids and risk of coronary heart disease in men. Circulation 2005; 111:157-64.
67. Department of Health and Human Services, U.S. Food and Drug Administration. Substances affirmed as generally recognized as safe: menhaden oil. Federal Register.

June 5, 1997. Vol 62, No 108: pp 30751-30757. 21 CFR Part 184 [Docket No. 86G-0289], 1997.
68. Bays HE. Safety considerations with omega-3 fatty acid therapy. Am J Cardiol 2007; 99:35C-43C.
69. Satterfield S, Greco PJ, Goldhaber SZ, Stampfer MJ, Swartz SL, Stein EA, Kaplan L, Hennekens CH. Biochemical markers of compliance in the Physicians' Health Study. Am J Prev Med 1990; 6:290-4.
70. Fritz AG, Percy C, Jack A, Shanmugaratham K, Sobin LH, Parkin MD, Whelan S. International Classification of Diseases for Oncology (ICD-O), Third Edition. Geneva: World Health Organization, 2000.
71. Thygesen K, Alpert JS, White HD, Joint ESC/ACCF/AHA/WHF Task Force for the Redefinition of Myocardial Infarction. Universal definition of myocardial infarction. Circulation 2007; 116:2634-53.
72. Adams HP, Jr., Bendixen BH, Kappelle LJ, Biller J, Love BB, Gordon DL, Marsh EE, 3rd. Classification of subtype of acute ischemic stroke. Definitions for use in a multicenter clinical trial. TOAST. Trial of Org 10172 in Acute Stroke Treatment. Stroke 1993; 24:35-41.
73. Cox DR. Regression models and life-tables. J Royal Statist Soc B 1972; 34:187-220.
74. Gooley TA, Leisenring W, Crowley J, Storer BE. Estimation of failure probabilities in the presence of competing risks: new representations of old estimators. Stat Med 1999; 18:695-706.
75. Putter H, Fiocco M, Geskus RB. Tutorial in biostatistics: competing risks and multi-state models. Stat Med 2007; 26:2389-430.
76. Fine JP, Gray RJ. A proportional hazards model for the subdistribution of a competing risk. J Am Stat Assoc 1999; 94:496-509.
77. Barlow WE. Robust variance estimation for the case-cohort design. Biometrics 1994; 50:1064-72.
78. Borgan O, Langholz B, Samuelsen SO, Goldstein L, Pogoda J. Exposure stratified case-cohort designs. Lifetime Data Anal 2000; 6:39-58.
79. Holick MF. Vitamin D for health and in chronic kidney disease. Semin Dial 2005; 18:266-75
80. Freedman LS. Tables of the number of patients required in clinical trials using the logrank test. Stat Med 1982; 1:121-9.
81. Brasky TM, Darke AK, Song X, et al. Plasma phospholipid fatty acids and prostate cancer risk in the SELECT trial. J Natl Cancer Inst 2013; 105:1132-41.
82. Haybittle JL. Repeated assessment of results in clinical trials of cancer treatment. Br J Radiol 1971; 44:793-7.
83. Peto R, Pike MC, Armitage P, Breslow NE, Cox DR, Howard SV, Mantel N, McPherson K, Peto J, Smith PG. Design and analysis of randomized clinical trials requiring prolonged observation of each patient. I. Introduction and design. Br J Cancer 1976; 34:585-612.
84. Geller NL, Pocock SJ. Interim analyses in randomized clinical trials: ramifications and guidelines for practitioners. Biometrics 1987; 43:213-23.
85. O'Brien PC, Fleming TR. A multiple testing procedure for clinical trials. Biometrics 1979; 35:549-56.
86. DeMets DL, Lan KK. Interim analysis: the alpha spending function approach. Stat Med 1994; 13:1341-52.
87. Pocock SJ. Current controversies in data monitoring for clinical trials. Clin Trials 2006; 3:513-21.

August 21, 2018

The VITamin D and OmegA-3 TriaL (VITAL): Summary of Changes to the Protocol:

Our initial IRB application, submitted in June 2009, contained a draft protocol.

January 28, 2010 (v. 2) was the date that the first full protocol was approved by the IRB. A copy of this protocol is provided.

January 24, 2011 (v. 3)

- Removed history of kidney stones as an exclusion criterion
- Updated information on the RDA for vitamin D and calcium based on the Nov 2010 IOM report
- For blood draw, replaced wording of “1 tube sodium citrate” with “1 serum tube”
- Specified that we expected to mail recruitment materials to 3.0 million people
- Specified that we would use an abbreviated version of the Willett Diet Assessment Questionnaire
- Specified that the vitamin D agents will be in the form of a soft-gel capsule
- Specified the omega-3 fatty acid agent as Omacor

March 31, 2011 (v. 4)

- Removed the education restriction that participants must have at least a high school education

June 8, 2011 (v.5)

- Eligible age range was lowered to 50 years or older for males and 55 or older for females, to reflect the younger age of development of heart disease and cancer among underrepresented minorities, and thus facilitate minority recruitment.

December 1, 2011 (v. 6)

- Volume of recruitment mailings was adjusted to 6 million people
- Angina was deleted as an exclusion
- Use of vitamin D analogs was added as an exclusion
- Recruitment via local health fairs was added as a recruitment strategy
- Added kidney failure or dialysis, parathyroid disease, cirrhosis or severe liver disease, use of anticoagulants as additional safety exclusions
- Added that staff will contact willing participants to schedule a home visit or clinic visit for collection of blood sample
- Specified that blood assays would be performed at Quest Diagnostics (other labs were deleted)
- Revised power calculations based on lowering the eligibility age range on 6/8/11.

January 6, 2012 (v. 7)

- Add “burping” as a side effect of fish oil
- Removed atrial fibrillation as a side effect of fish oil due to inconsistent evidence.

December 3, 2012 (v. 8)

- Specified that study will be conducted among 25,000 men and women
- Specified that the mailing company will conduct recruitment mailings in volume of 0.5-1 million per drop

- The number of willing and eligible persons to receive screening questionnaire (V-2) was projected to be 160,000
- Specified that non-responders to run-in follow-up questionnaire (V-3) will receive up to 3 requests for data
- Specified that toward the end of the enrollment phase, as a mechanism to enhance minority representation in the trial, we will impose a stricter compliance standard on white, non-Hispanic enrollees who live outside the 60-mile range for CTSC recruitment: not eligible if a blood sample is not returned and missed more than 5 days of run-in placebo pills in past month
- Contacting the “not sure” and “no” respondents to question about providing a blood sample to inform them that we can arrange a home visit if they wanted
- Study guidelines amended to encourage randomized participants to inform their physicians about their participation in the trial if they receive a post-randomization diagnosis of hypercalcemia, sarcoidosis, or other safety issues
- Provided new power calculations based upon a study population of 25,000

April 1, 2013 (v. 9)

- Blood sample analyses now to include measures of biomarkers related to CVD (cholesterol lipoprotein sub-fractions) – to be conducted at Atherotech Diagnostic Labs
- Specified that non-responders to annual follow-up questionnaires will receive up to 5 requests for information

September 10, 2013 (v. 10)

- Added information about the SELECT Study results (observational association between omega-3 fatty acids and risk of prostate cancer) to the “Background and Significance” section of protocol

September 17, 2013 (v. 11)

- Specified that the projected randomized population would be a maximum of 26,500
- Added biochemical assays of blood specimens of metabolomic/lipidomic biomarkers for baseline and follow-up samples

December 5, 2013 (v. 12)

- Specified that internet sources will be used to assist in the tracking (i.e., search for new address and phone numbers) of participants potentially lost to follow-up

December 17, 2013 (v. 13)

- Added measurement of plasma levels of PCSK9 and plasma-based genotypes for PCSK9

March 19, 2014 (v. 14)

- Specified an increase in the target for optional follow-up blood sample from 2,000 to 6,000. Deletion of “random” to describe selection of follow-up samples, as participants asked to provide the samples will be those scheduled for an ancillary blood draw at a home visit.

May 21, 2014 (v. 15)

- Specified that participants will be given the option of completing their annual questionnaire via the Internet.

June 19, 2014 (v. 16)

- Specified the planned collection of the follow-up Diet Assessment at 3 years instead of 2 years
- Added as a tertiary aim whether vitamin D or omega-3 supplementation reduces the risks of heart failure (ancillary study)

November 11, 2015 (v. 17)

- Specified that the follow-up Diet Assessment would be conducted at 4.5 years instead of 3 years
- Added to "Endpoint Ascertainment" section: Additional endpoints of interest include heart failure, colorectal polyps, and periodontal disease.

January 6, 2016 (v. 18)

- Added biochemical assays: measures of metabolomics and lipidomic biomarkers to be done at LabCorp in addition to the metabolomics laboratory at UCSD

April 29, 2016 (v. 19)

- Deleted reference to an additional Diet Assessment at conclusion of the trial (to minimize participant burden).

September 12, 2016 (v. 20)

- To increase completeness of ascertainment of study outcome data (for MI, stroke, revascularization, and cancer), specified that Medicare/CMS linkage would be done. However, participants will be directly asked to provide authorization to obtain medical records to validate outcomes ascertained from Medicare/CMS.

January 27, 2017 (v. 21)

- Specified the addition of biochemical assays: albumin, creatinine, liver function, lipid tests, cholesterol efflux, epigenetic biomarkers.

September 13, 2017 (v. 22)

- Specified that, at the conclusion of the randomized trial, we will send participants two additional annual questionnaires to collect supplemental study data during the post-intervention phase.

November 30, 2017 (v. 23) is the current protocol. A copy is provided.

- Clarified that we will additionally conduct analyses excluding events occurring in year 1 and years 1-2 of follow-up (our original protocol on 1/28/10 [v.2] specified: "Finally, we will examine whether treatment effects vary over time and duration of treatment by examining survival plots and interactions with time. There may be a latent effect on cancer incidence, depending on the stage of carcinogenesis during which these agents act." In the 11/30/17 protocol, we formalized this as analyses excluding events during year 1 and years 1-2 of follow-up.)
- Data analysis section amplified: specified sensitivity analyses by compliance, with participants' follow-up time censored when they are no longer compliant with taking at least two-thirds of their assigned active agents or placebos (as in our previous trials).

<u>NOTE</u>: VITAL investigators were unblinded to randomization treatment codes on December 6, 2017 prior to a teleconference with VITAL DSMB members. All of the above protocol changes were made PRIOR to the investigator unblinding on December 6, 2017. The intervention phase of VITAL ended on December 31, 2017.

VI. BIOSTATISTICAL ANALYSIS

A. DATA ANALYSIS

Analyses of treatment effects will be based on the intent-to-treat principle. The first analysis will compare baseline characteristics by randomized treatment assignment to ensure that balance was achieved by the randomization. Characteristics to be examined include known risk factors for cancer and CVD, including age; gender; race/ethnicity; BMI; smoking; alcohol use; physical activity; medical conditions such as hypertension, hyperlipidemia, diabetes, and family history of cancer and CVD; and baseline vitamin D and omega-3 fatty acid levels as assessed by dietary questionnaire in all participants and blood assays in a case-cohort subsample. The large sample size, as well as successful balance of known potential confounders, will provide assurance that unmeasured or unknown potential confounders will also be equally distributed across randomized treatment groups.

In this 2x2 factorial design, the primary aim is to compare the main effects of intention-to-treat with vitamin D and with fish oil on cancer and CVD. We will use the Cox proportional hazards model to allow for variable follow-up lengths[76] and will estimate the hazard ratio for each intervention using indicators for treatment exposure, controlling for the second intervention, age, and gender. Because the cohort will consist of older individuals, competing risks due to deaths from other causes will be considered. The primary analysis will estimate the cause-specific hazard and the hazard ratio comparing intervention groups for each outcome of interest by censoring individuals with deaths due to competing causes. To estimate the cumulative incidence function, the subdistribution of each endpoint will be plotted over time.[77, 78] The alternative Fine and Gray approach[79] models the effect of treatment on the subdistribution hazard or directly on the cumulative incidence function. While we will consider this and compare its fit to the model-free cumulative incidence curves,[78] the proportional hazards approach will be our primary analysis.

Similar analyses will be conducted for both cancer and CVD. For cancer, we will estimate the intervention effects on the primary endpoint of total cancer, as well as the secondary outcomes of breast, prostate, and colorectal cancer, and cancer mortality. For CVD we will estimate the effects on the primary composite endpoint of major CVD (MI, stroke, and cardiovascular mortality); on the secondary composite endpoint that additionally includes coronary revascularization; and on the individual components of MI, stroke, revascularization, and cardiovascular mortality.

Beyond the primary analyses, we will examine effect modification by the other randomized intervention, by baseline risk factors, and by time. We have a particular interest in exploring interactions between the interventions, as well as with the baseline plasma biomarkers of 25(OH)D for vitamin D and of EPA+DHA for fish oil. We hypothesize that the intervention effects may be larger among those with below-median baseline levels, and will examine treatment effects by quartiles of these biomarkers. The biomarker data will be analyzed as a case-cohort study using methods for proportional hazards regression.[80] Because of the frequency matching by age and gender, appropriate stratum-specific weighting of the observations will be employed.[81] We also have a prior interest in the effects of the vitamin D intervention within

groups defined by race/ethnicity and skin pigmentation, and by BMI. In addition, we will evaluate effect modification by age and gender, as well as by sunlight exposure, calcium and phosphorus intakes from the FFQ (as these nutrients affect vitamin D bioavailability[82]), and baseline risk factors for cancer and CVD. The latter interaction effects will be interpreted cautiously, as hypothesis generating. Finally, we will examine whether treatment effects vary over time and duration of treatment by examining survival plots and interactions with time. There may be a latent effect on cancer incidence, depending on the stage of carcinogenesis during which these agents act.

We will also compare the incidence of potential side effects in the active *v.* placebo groups for each agent, including the incidence of kidney stones with vitamin D assignment (cf. WHI calcium-vitamin D findings[44]) and the incidence of GI symptoms, skin abnormalities, and bleeding with fish oil assignment (cf. JELIS findings[41]).

B. POWER CALCULATIONS

To calculate power, the following assumptions were made: (1) a 2x2 factorial trial in 10,000 men aged ≥60 and 10,000 women aged ≥65; (2) independent and equal allocation of participants to each treatment; (3) an age distribution based on that observed at baseline in our past trials for men aged ≥60 and women aged ≥65; (4) age-specific event rates based on the observed rates in the first 5 years of the PHS II for men and the WHS for women; (5) trial follow-up of 5 years, with little loss to follow-up as achieved in our past trials; and (6) compliance similar to that in published trials upon which our estimated rate ratio (RR) reductions are based. The cited reductions are thus the observed effects we would see in the trial. The corresponding 'true' RR is given assuming an average compliance of 80%. Power is given for a two-sided test using a logrank analysis[83] with a significance level of 0.05.

Table 1. Power for effects of a single agent on cancer in a factorial trial of 10,000 men aged≥60 and 10,000 women aged≥65 with 5 years of follow-up

Observed RR†	True RR	Total Cancer	Cancer Mortality	Colorectal Cancer	Breast Cancer (Women)	Prostate Cancer (Men)
0.90	0.875	58.7	-	-	-	-
0.85	0.812	91.1	-	-	-	-
0.80	0.750	99.4	52.0	31.8	34.6	70.1
0.75	0.687	>99.9	72.2	47.0	50.9	88.3
0.70	0.625	>99.9	87.3	63.1	67.5	96.9
0.65	0.560	>99.9	95.6	77.6	81.5	99.5
0.60	0.500	>99.9	98.9	88.4	91.2	>99.9

†Observed RR=intent-to-treat RR, including noncompliant participants. Compliance assumed to be 80%. True RR=that with perfect compliance.

Cancer: Assuming that only one agent is effective, with 5 years of treatment and follow-up we will have 91% power to detect an observed RR of 0.85 for the primary endpoint of total cancer incidence (Table 1). For prostate cancer, we will have 88% power to detect a 25% risk reduction. For breast cancer, we will have 81% power to detect a 35% risk reduction. For colorectal cancer, we will have 78% power to detect a 35% risk reduction and 88% power to detect a 40% risk reduction. For cancer mortality, we will have 87% power to detect a 30% risk reduction. If both agents are effective in preventing cancer but act independently, power would be reduced slightly due to a somewhat smaller overall number of events. If the agents interact, however, power will be affected to the extent of the interaction. Should the agents act synergistically, power would increase, as illustrated in Table 2 for the outcome of total cancer.

For example, if the effect of each agent alone is a reduction of 10%, but in combination the effect is stronger, with an additional 10% decrease, the RR comparing the combined group to the all placebo group would be 0.73, versus 0.81 with additive effects (on the multiplicative

scale). Power for the main effect of each agent would then increase to 86% (or higher with greater synergy). The trial would have power to detect moderate-to-large interactions, as shown in Table 2. Should the agents interact in a subadditive fashion, power would be reduced to the extent of the subadditivity. Power to explore effect modification by baseline blood levels of 25(OH)D and EPA+DHA among those above and below the median level in the cohort will be lower because we anticipate that about 60% of participants will provide blood specimens. We will have about 90% power to detect an interaction of size 0.6.

Power has been shown for a given observed reduction in risk, assuming compliance similar to that seen in current randomized trials such as the WHS and PHS. For total cancer, our power will be excellent with a 5-year follow-up even if compliance is lower. For example, if the true reduction in risk is 20%, the observed RR corresponding to compliance of 80% is 84%, and we have 94% power to detect this level of effect. If compliance is only 75%, the observed RR would be 0.85, for which we would have 91% power. Finally, if compliance is only 70%, the observed RR would be 0.86, and we would still have 87% power to detect an effect of this magnitude.

Table 2. Power for interaction effects on total cancer in a factorial trial of 10,000 men aged ≥60 and 10,000 women aged ≥65 with 5 years of follow-up

RR Single agent†	Interaction†	RR Both agents	Power Main Effect	Power Interaction
0.90	1.0	0.81	56.3	
	0.9	0.73	86.3	
	0.8	0.65	98.0	57.9
	0.7	0.57	99.9	92.1
0.85	1.0	0.72	88.7	
	0.9	0.65	98.1	
	0.8	0.58	99.8	55.0
	0.7	0.51	>99.9	90.2
0.80	1.0	0.64	98.8	
	0.9	0.58	99.9	
	0.8	0.51	>99.9	52.0
	0.7	0.45	>99.9	87.9

†RR = intent-to-treat RR, including noncompliant participants (assuming 80% compliance). Represents the effect among those not assigned to the other intervention and assumes the same effect for both agents. The interaction is the RR for the combined group divided by the product of risks for the two separate groups—i.e., RRint=RRboth/(RR vit D alone* RR fish oil alone). An interaction=1 implies additive effects (no interaction).

CVD: As shown in Table 3, assuming that only one agent is effective, with 5 years of treatment and follow-up we will have 92% power to detect an observed RR of 0.80 for the primary composite endpoint of MI, stroke, and cardiovascular mortality. For the secondary composite endpoint of MI, stroke, cardiovascular mortality, and coronary revascularization, we will have 99% power to detect a 20% reduction and 90% power to detect a 15% reduction. With a RR of 0.70 we will have 87% power for cardiovascular mortality, 88% power for MI, and 90% power for stroke. If both agents are effective in preventing CVD but act independently, power would be reduced only slightly due to a smaller overall number of events. If the agents act synergistically, power would increase, as described for cancer. If the agents interact in subadditive fashion, power would be reduced to the extent of the subadditivity.

Table 3. Power for effects of a single agent on CVD in a factorial trial of 10,000 men aged ≥60 and 10,000 women aged ≥65, with 5 years of follow-up						
Observed RR†	True RR	Major CVD	Total CVD	CVD Mortality	MI	Stroke
0.90	0.875	38.1	57.3	-	-	-
0.85	0.812	71.1	90.2	-	-	-
0.80	0.750	92.5	99.2	51.8	53.5	56.1
0.75	0.687	99.0	>99.9	71.9	73.8	76.3
0.70	0.625	99.9	>99.9	87.1	88.5	90.3
0.65	0.560	>99.9	>99.9	95.6	96.3	97.1
0.60	0.500	>99.9	>99.9	98.9	99.1	99.4

†Observed RR=intent-to-treat RR, including noncompliant participants. Compliance is assumed to be 80%. True RR=that with perfect compliance.

For total CVD, our power would be somewhat lower—but still adequate—with more limited compliance. If the true effect is a 25% reduction, power would be 92% if compliance is 80% (observed RR=0.80), 89% if compliance is 75% (observed RR=0.813), and 84% if compliance is 70% (observed RR=0.825). To achieve 80% power for CVD, we would need an observed RR of at least 0.83, which corresponds to a true reduction of 21% under 80% compliance and 24% under 70% compliance.

IX. MONITORING AND QUALITY ASSURANCE

A. SAFETY MONITORING

An independent Data and Safety Monitoring Board (DSMB) will be assembled, consisting of experts in clinical trials, epidemiology, biostatistics, relevant clinical areas of cancer and CVD, and NIH representatives. The Physicians' Health Study, the Women's Health Study, and the Women's Antioxidant and Folic Acid Cardiovascular Study have been monitored by the same DSMB since their inception. As two of these trials have ended and the third is in its last years, we will ask our DSMB members if they would be willing to also monitor VITAL. If they are not able to do so, we will consult with them in assembling a new DSMB.

The DSMB will annually examine the progress of the trial and the unblinded data on study endpoints and possible adverse effects to recommend continuation, alteration of study design, or early termination, as appropriate. Interim trial results will be assessed with the Haybittle-Peto rule,[84, 85] adjusting for multiple looks. In this method, interim results are compared to a z-score of 3 standard deviations (p=0.0027) throughout the trial. The final results may then be interpreted as having close to nominal significance levels. This rule appropriately requires very strong evidence for early stopping, is more conservative than the Pocock[86] and O'Brien-Fleming[87] rules and the alpha-spending function,[88] and can be conducted at convenient times without inducing statistical complexity.[89]

While these rules are intended for the primary endpoints, the goal of VITAL is to assess the overall balance of benefits and risks of the two agents in the primary prevention of cancer and CVD. Thus, consideration will also be given to the secondary endpoints that are needed in the interpretation of overall results. In addition, the monitoring rules will serve solely as guidelines in

decisions regarding continuation or stopping of treatment arms. All decisions must be made after examining the totality of evidence, including other trial data, on these agents.

X. REFERENCES

1. Davis CD, Hartmuller V, Freedman DM, Hartge P, Picciano MF, Swanson CA, Milner JA. Vitamin D and cancer: current dilemmas and future needs. Nutr Rev 2007; 65:S71-4.
2. Institute of Medicine Food and Nutrition Board. Dietary Reference Intakes: Calcium, Phosphorous, Magnesium, Vitamin D and Fluoride. Washington DC: National Academies Press, 1999.
3. Giovannucci E. Strengths and limitations of current epidemiologic studies: vitamin D as a modifier of colon and prostate cancer risk. Nutr Rev 2007; 65:S77-9.
4. Looker AC, Dawson-Hughes B, Calvo MS, Gunter EW, Sahyoun NR. Serum 25-hydroxyvitamin D status of adolescents and adults in two seasonal subpopulations from NHANES III. Bone 2002; 30:771-7.
5. Holick MF. Vitamin D deficiency. N Engl J Med 2007; 357:266-81.
6. Harris SS. Vitamin D and African Americans. J Nutr 2006; 136:1126-9.
7. Moore CE, Murphy MM, Holick MF. Vitamin D intakes by children and adults in the United States differ among ethnic groups. J Nutr 2005; 135:2478-85.
8. Martins D, Wolf M, Pan D, Zadshir A, Tareen N, Thadhani R, Felsenfeld A, Levine B, Mehrotra R, Norris K. Prevalence of cardiovascular risk factors and the serum levels of 25-hydroxyvitamin D in the United States: data from the Third National Health and Nutrition Examination Survey. Arch Intern Med 2007; 167:1159-65.
9. Harris SS, Dawson-Hughes B. Reduced sun exposure does not explain the inverse association of 25-hydroxyvitamin D with percent body fat in older adults. J Clin Endocrinol Metab 2007; 92:3155-7.
10. Hedley AA, Ogden CL, Johnson CL, Carroll MD, Curtin LR, Flegal KM. Prevalence of overweight and obesity among US children, adolescents, and adults, 1999-2002. JAMA 2004; 291:2847-50.
11. Ogden CL, Carroll MD, Curtin LR, McDowell MA, Tabak CJ, Flegal KM. Prevalence of overweight and obesity in the United States, 1999-2004. JAMA 2006; 295:1549-55.
12. Kris-Etherton PM, Harris WS, Appel LJ. Fish consumption, fish oil, omega-3 fatty acids, and cardiovascular disease. Circulation 2002; 106:2747-57.
13. NIH Office of Dietary Supplements, National Heart Lung and Blood Institute. Working Group Report on Future Clinical Research Directions on Omega-3 Fatty Acids and Cardiovascular Disease. June 2, 2004. Bethesda MD. Available at: http://www.nhlbi.nih.gov/meetings/workshops/omega-3/omega-3-rpt.htm Accessed on March 28, 2008.
14. Stipp D. Fish-oil doses can be hard to swallow. Wall Street Journal: January 8, 2008; Page D1-D2.
15. Gosnell M. Top 100 science stories of 2007: #8: Can vitamin D save your life? Discover: December 12, 2007. Available at http://discovermagazine.com/2008/jan/year-in-science-2007/can-vitamin-d-save-your-life Accessed March 25, 2008.
16. Guthrie C. The 10 biggest medical breakthroughs. #10: Benefits of vitamin D. Time: December 24, 2007. Available at: http://www.time.com/time/specials/2007/top10/article/0,30583,1686204_1686252_1690393,00.html Accessed March 25, 2008.
17. Harvard Health Letter. The top 10 health stories of 2006. Harv Health Lett 2006; 32:1-3.
18. Frisinger C. New year, new you: 8 simple rules for a healthier 2008. #1. Get more vitamin D. Fort Worth Star-Telegram (Texas): January 1, 2008; Page E1.

19. Brody JE. An oldie vies for nutrient of the decade. New York Times: February 19, 2008; Page F7.
20. Stampfer MJ. Vitamin D in the spotlight. Newsweek: December 11, 2006. Available at: http://www.newsweek.com/id/43998 Accessed March 25, 2008.
21. Peng T. Don't forget your vitamins. Newsweek: March 24, 2008; Page 78.
22. Rosenfeld I. We may need more vitamin D. Parade Magazine: January 29, 2006; Page 15.
23. Rhodes P. Vitamin D essentials: what you need to know about this important nutrient and where best to obtain it. Cooking Light 2008; 22:44-50.
24. Foreman J. Is there any way to tell whether you are getting enough vitamin D? Boston Globe: February 4, 2008; Page C2.
25. Harvard Health Letter. A more D-manding diet. Some experts say we should have a lot more vitamin D in our diets because it's protective against several diseases. Harv Health Lett 2007; 32:3.
26. Simopoulos AP, Robinson J. The Omega Diet: The Lifesaving Nutritional Program Based on the Diet of the Island of Crete. New York: HarperCollins, 1999.
27. Sears B. The Omega Rx Zone: The Miracle of the New High-Dose Fish Oil. New York: HarperCollins, 2004.
28. Kaufman M. Fish oil may aid against manic depression. Washington Post, 1999:Z07.
29. Foreman J. Fatty acid imbalance hurts our health. Boston Globe: February 10, 2004.
30. Bakalar N. Fish oil linked to lower Alzheimer's risk. New York Times: November 14, 2006.
31. Munoz SS. Shopping around: DHA-enhanced foods. Wall Street Journal: March 27, 2008; Page D2.
32. Canadian Cancer Society. Canadian Cancer Society Announces Vitamin D Recommendation. Available at: http://www.cancer.ca/ccs/internet/mediareleaselist/0,,3172_1613121606_1997621989_langId-en.html. Accessed May 6, 2008.
33. Vieth R, Bischoff-Ferrari H, Boucher BJ, Dawson-Hughes B, Garland CF, Heaney RP, Holick MF, Hollis BW, Lamberg-Allardt C, McGrath JJ, Norman AW, Scragg R, Whiting SJ, Willett WC, Zittermann A. The urgent need to recommend an intake of vitamin D that is effective. Am J Clin Nutr 2007; 85:649-50.
34. Davis CD, Dwyer JT. The "sunshine vitamin": benefits beyond bone? J Natl Cancer Inst 2007; 99:1563-5.
35. Bardia A, Tleyjeh IM, Cerhan JR, Sood AK, Limburg PJ, Erwin PJ, Montori VM. Efficacy of antioxidant supplementation in reducing primary cancer incidence and mortality: systematic review and meta-analysis. Mayo Clin Proc 2008; 83:23-34.
36. Mosca L, Banka CL, Benjamin EJ, Berra K, Bushnell C, Dolor RJ, Ganiats TG, Gomes AS, Gornik HL, Gracia C, Gulati M, Haan CK, Judelson DR, Keenan N, Kelepouris E, Michos ED, Newby LK, Oparil S, Ouyang P, Oz MC, Petitti D, Pinn VW, Redberg RF, Scott R, Sherif K, Smith SC, Jr., Sopko G, Steinhorn RH, Stone NJ, Taubert KA, Todd BA, Urbina E, Wenger NK. Evidence-based guidelines for cardiovascular disease prevention in women: 2007 update. Circulation 2007; 115:1481-501.
37. Kris-Etherton PM, Lichtenstein AH, Howard BV, Steinberg D, Witztum JL. Antioxidant vitamin supplements and cardiovascular disease. Circulation 2004; 110:637-41.
38. Lee IM, Cook NR, Gaziano JM, Gordon D, Ridker PM, Manson JE, Hennekens CH, Buring JE. Vitamin E in the primary prevention of cardiovascular disease and cancer: the Women's Health Study: a randomized controlled trial. JAMA 2005; 294:56-65.
39. Miller ER, 3rd, Pastor-Barriuso R, Dalal D, Riemersma RA, Appel LJ, Guallar E. Meta-analysis: high-dosage vitamin E supplementation may increase all-cause mortality. Ann Intern Med 2005; 142:37-46.

40. Bjelakovic G, Nikolova D, Gluud LL, Simonetti RG, Gluud C. Mortality in randomized trials of antioxidant supplements for primary and secondary prevention: systematic review and meta-analysis. JAMA 2007; 297:842-57.
41. Yokoyama M, Origasa H, Matsuzaki M, Matsuzawa Y, Saito Y, Ishikawa Y, Oikawa S, Sasaki J, Hishida H, Itakura H, Kita T, Kitabatake A, Nakaya N, Sakata T, Shimada K, Shirato K. Effects of eicosapentaenoic acid on major coronary events in hypercholesterolaemic patients (JELIS): a randomised open-label, blinded endpoint analysis. Lancet 2007; 369:1090-8.
42. Gruppo Italiano per lo Studio della Sopravvivenza nell'Infarto miocardico. Dietary supplementation with n-3 polyunsaturated fatty acids and vitamin E after myocardial infarction: results of the GISSI-Prevenzione trial. Gruppo Italiano per lo Studio della Sopravvivenza nell'Infarto miocardico. Lancet 1999; 354:447-55.
43. Lappe JM, Travers-Gustafson D, Davies KM, Recker RR, Heaney RP. Vitamin D and calcium supplementation reduces cancer risk: results of a randomized trial. Am J Clin Nutr 2007; 85:1586-91.
44. Wactawski-Wende J, Kotchen JM, Anderson GL, Assaf AR, Brunner RL, O'Sullivan MJ, Margolis KL, Ockene JK, Phillips L, Pottern L, Prentice RL, Robbins J, Rohan TE, Sarto GE, Sharma S, Stefanick ML, Van Horn L, Wallace RB, Whitlock E, Bassford T, Beresford SA, Black HR, Bonds DE, Brzyski RG, Caan B, Chlebowski RT, Cochrane B, Garland C, Gass M, Hays J, Heiss G, Hendrix SL, Howard BV, Hsia J, Hubbell FA, Jackson RD, Johnson KC, Judd H, Kooperberg CL, Kuller LH, LaCroix AZ, Lane DS, Langer RD, Lasser NL, Lewis CE, Limacher MC, Manson JE. Calcium plus vitamin D supplementation and the risk of colorectal cancer. N Engl J Med 2006; 354:684-96.
45. Hathcock JN, Shao A, Vieth R, Heaney R. Risk assessment for vitamin D. Am J Clin Nutr 2007; 85:6-18.
46. Lang JM, Buring JE, Rosner B, Cook N, Hennekens CH. Estimating the effect of the run-in on the power of the Physicians' Health Study. Stat Med 1991; 10:1585-93.
47. Talwar SA, Aloia JF, Pollack S, Yeh JK. Dose response to vitamin D supplementation among postmenopausal African American women. Am J Clin Nutr 2007; 86:1657-62.
48. Prentice RL. A case-cohort design for epidemiologic cohort studies and disease prevention trials. Biometrika 1986; 73:1-11.
49. Rimm EB, Giovannucci EL, Stampfer MJ, Colditz GA, Litin LB, Willett WC. Reproducibility and validity of an expanded self-administered semiquantitative food frequency questionnaire among male health professionals. Am J Epidemiol 1992; 135:1114-26.
50. Feskanich D, Rimm EB, Giovannucci EL, Colditz GA, Stampfer MJ, Litin LB, Willett WC. Reproducibility and validity of food intake measurements from a semiquantitative food frequency questionnaire. J Am Diet Assoc 1993; 93:790-6.
51. Salvini S, Hunter DJ, Sampson L, Stampfer MJ, Colditz GA, Rosner B, Willett WC. Food-based validation of a dietary questionnaire: the effects of week-to-week variation in food consumption. Int J Epidemiol 1989; 18:858-67.
52. Jacques PF, Sulsky SI, Sadowski JA, Phillips JC, Rush D, Willett WC. Comparison of micronutrient intake measured by a dietary questionnaire and biochemical indicators of micronutrient status. Am J Clin Nutr 1993; 57:182-9.
53. Feskanich D, Willett WC, Colditz GA. Calcium, vitamin D, milk consumption, and hip fractures: a prospective study among postmenopausal women. Am J Clin Nutr 2003; 77:504-11.
54. Hunter DJ, Rimm EB, Sacks FM, Stampfer MJ, Colditz GA, Litin LB, Willett WC. Comparison of measures of fatty acid intake by subcutaneous fat aspirate, food frequency questionnaire, and diet records in a free-living population of US men. Am J Epidemiol 1992; 135:418-27.

55. Vieth R. Critique of the considerations for establishing the tolerable upper intake level for vitamin D: critical need for revision upwards. J Nutr 2006; 136:1117-22.
56. Bischoff-Ferrari HA, Giovannucci E, Willett WC, Dietrich T, Dawson-Hughes B. Estimation of optimal serum concentrations of 25-hydroxyvitamin D for multiple health outcomes. Am J Clin Nutr 2006; 84:18-28.
57. Dawson-Hughes B, Heaney RP, Holick MF, Lips P, Meunier PJ, Vieth R. Estimates of optimal vitamin D status. Osteoporos Int 2005; 16:713-6.
58. Giovannucci E, Liu Y, Rimm EB, Hollis BW, Fuchs CS, Stampfer MJ, Willett WC. Prospective study of predictors of vitamin D status and cancer incidence and mortality in men. J Natl Cancer Inst 2006; 98:451-9.
59. Aloia JF, Patel M, DiMaano R, Li-Ng M, Talwar SA, Mikhail M, Pollack S, Yeh JK. Vitamin D intake to attain a desired serum 25-hydroxyvitamin D concentration. Am J Clin Nutr 2008;87:1952-1958
60. Harris WS. International recommendations for consumption of long-chain omega-3 fatty acids. J Cardiovasc Med (Hagerstown) 2007; 8 Suppl 1:S50-2.
61. Lee JH, O'Keefe JH, Lavie CJ, Marchioli R, Harris WS. Omega-3 fatty acids for cardioprotection. Mayo Clin Proc 2008; 83:324-32. [Docket No. 86G-0289], 1997.
62. Agency for Healthcare Research and Quality. Effects of Omega-3 Fatty Acids on Cardiovascular Disease. Evidence Report/Technology Assessment Number 94. AHRQ Pub. No. 04-E009-1. 2004. Available at: http://www.ahrq.gov/clinic/epcsums/o3cardsum.htm Accessed March 28, 2008.
63. Simopoulos AP. Evolutionary aspects of diet, the omega-6/omega-3 ratio and genetic variation: nutritional implications for chronic diseases. Biomed Pharmacother 2006; 60:502-7.
64. Kris-Etherton PM, Taylor DS, Yu-Poth S, Huth P, Moriarty K, Fishell V, Hargrove RL, Zhao G, Etherton TD. Polyunsaturated fatty acids in the food chain in the United States. Am J Clin Nutr 2000; 71:179S-88S.
65. Harris WS. The omega-6/omega-3 ratio and cardiovascular disease risk: uses and abuses. Curr Atheroscler Rep 2006; 8:453-9.
66. Stanley JC, Elsom RL, Calder PC, Griffin BA, Harris WS, Jebb SA, Lovegrove JA, Moore CS, Riemersma RA, Sanders TA. UK Food Standards Agency Workshop Report: the effects of the dietary n-6:n-3 fatty acid ratio on cardiovascular health. Br J Nutr 2007; 98:1305-10.
67. Harris WS, Poston WC, Haddock CK. Tissue n-3 and n-6 fatty acids and risk for coronary heart disease events. Atherosclerosis 2007; 193:1-10.
68. Mozaffarian D, Ascherio A, Hu FB, Stampfer MJ, Willett WC, Siscovick DS, Rimm EB. Interplay between different polyunsaturated fatty acids and risk of coronary heart disease in men. Circulation 2005; 111:157-64.
69. Department of Health and Human Services, U.S. Food and Drug Administration. Substances affirmed as generally recognized as safe: menhaden oil. Federal Register. June 5, 1997. Vol 62, No 108: pp 30751-30757. 21 CFR Part 184 [Docket No. 86G-0289], 1997.52. Jacques PF, Sulsky SI, Sadowski JA, Phillips JC, Rush D, Willett WC. Comparison of micronutrient intake measured by a dietary questionnaire and biochemical indicators of micronutrient status. Am J Clin Nutr 1993; 57:182-9.
70. Harris WS. Expert opinion: omega-3 fatty acids and bleeding--cause for concern? Am J Cardiol 2007; 99:44C-46C.
71. Bays HE. Safety considerations with omega-3 fatty acid therapy. Am J Cardiol 2007; 99:35C-43C.
72. Satterfield S, Greco PJ, Goldhaber SZ, Stampfer MJ, Swartz SL, Stein EA, Kaplan L, Hennekens CH. Biochemical markers of compliance in the Physicians' Health Study. Am J Prev Med 1990; 6:290-4.

73. Fritz AG, Percy C, Jack A, Shanmugaratham K, Sobin LH, Parkin MD, Whelan S. International Classification of Diseases for Oncology (ICD-O), Third Edition. Geneva: World Health Organization, 2000.
74. Thygesen K, Alpert JS, White HD, Joint ESC/ACCF/AHA/WHF Task Force for the Redefinition of Myocardial Infarction. Universal definition of myocardial infarction. Circulation 2007; 116:2634-53.
75. Adams HP, Jr., Bendixen BH, Kappelle LJ, Biller J, Love BB, Gordon DL, Marsh EE, 3rd. Classification of subtype of acute ischemic stroke. Definitions for use in a multicenter clinical trial. TOAST. Trial of Org 10172 in Acute Stroke Treatment. Stroke 1993; 24:35-41.
76. Cox DR. Regression models and life-tables. J Royal Statist Soc B 1972; 34:187-220.
77. Gooley TA, Leisenring W, Crowley J, Storer BE. Estimation of failure probabilities in the presence of competing risks: new representations of old estimators. Stat Med 1999; 18:695-706.
78. Putter H, Fiocco M, Geskus RB. Tutorial in biostatistics: competing risks and multi-state models. Stat Med 2007; 26:2389-430.
79. Fine JP, Gray RJ. A proportional hazards model for the subdistribution of a competing risk. J Am Stat Assoc 1999; 94:496-509.
80. Barlow WE. Robust variance estimation for the case-cohort design. Biometrics 1994; 50:1064-72.
81. Borgan O, Langholz B, Samuelsen SO, Goldstein L, Pogoda J. Exposure stratified case-cohort designs. Lifetime Data Anal 2000; 6:39-58.
82. Holick MF. Vitamin D for health and in chronic kidney disease. Semin Dial 2005; 18:266-75
83. Freedman LS. Tables of the number of patients required in clinical trials using the logrank test. Stat Med 1982; 1:121-9.
84. Haybittle JL. Repeated assessment of results in clinical trials of cancer treatment. Br J Radiol 1971; 44:793-7.
85. Peto R, Pike MC, Armitage P, Breslow NE, Cox DR, Howard SV, Mantel N, McPherson K, Peto J, Smith PG. Design and analysis of randomized clinical trials requiring prolonged observation of each patient. I. Introduction and design. Br J Cancer 1976; 34:585-612.
86. Geller NL, Pocock SJ. Interim analyses in randomized clinical trials: ramifications and guidelines for practitioners. Biometrics 1987; 43:213-23.
87. O'Brien PC, Fleming TR. A multiple testing procedure for clinical trials. Biometrics 1979; 35:549-56.
88. DeMets DL, Lan KK. Interim analysis: the alpha spending function approach. Stat Med 1994; 13:1341-52.
89. Pocock SJ. Current controversies in data monitoring for clinical trials. Clin Trials 2006; 3:513-21.

VI. BIOSTATISTICAL ANALYSIS

A. DATA ANALYSIS

Analyses of treatment effects will be based on the intent-to-treat principle. The first analysis will compare baseline characteristics by randomized treatment assignment to ensure that balance was achieved by the randomization. Characteristics to be examined include known risk factors for cancer and CVD, including age; gender; race/ethnicity; BMI; smoking; alcohol use; physical activity; medical conditions such as hypertension, hyperlipidemia, diabetes, and family history of cancer and CVD; and baseline vitamin D and omega-3 fatty acid levels as assessed by dietary questionnaire in all participants and blood assays in a case-cohort subsample. The large sample size, as well as successful balance of known potential confounders, will provide assurance that unmeasured or unknown potential confounders will also be equally distributed across randomized treatment groups.

In this 2x2 factorial design, the primary aim is to compare the main effects of intention-to-treat with vitamin D softgel capsules and with Omacor® fish oil on cancer and CVD. We will use the Cox proportional hazards model to allow for variable follow-up lengths[73] and will estimate the hazard ratio for each intervention using indicators for treatment exposure, controlling for the second intervention, age, and gender. Because the cohort will consist of older individuals, competing risks due to deaths from other causes will be considered. The primary analysis will estimate the cause-specific hazard and the hazard ratio comparing intervention groups for each outcome of interest by censoring individuals with deaths due to competing causes. To estimate the cumulative incidence function, the subdistribution of each endpoint will be plotted over time.[74,75] The alternative Fine and Gray approach[76] models the effect of treatment on the subdistribution hazard or directly on the cumulative incidence function. While we will consider this and compare its fit to the model-free cumulative incidence curves,[75] the proportional hazards approach will be our primary analysis.

Similar analyses will be conducted for both cancer and CVD. For cancer, we will estimate the intervention effects on the primary endpoint of total cancer, as well as the secondary outcomes of breast, prostate, and colorectal cancer, and cancer mortality. For CVD we will estimate the effects on the primary composite endpoint of major CVD (MI, stroke, and cardiovascular mortality); on the secondary composite endpoint that additionally includes coronary revascularization; and on the individual components of MI, stroke, revascularization, and cardiovascular mortality.

Beyond the primary analyses, we will conduct prespecified secondary analyses to examine effect modification by the other randomized intervention, by baseline risk factors, by compliance, and by time. We have a particular interest in exploring interactions between the interventions, as well as with the baseline plasma biomarkers of 25(OH)D for vitamin D and of EPA+DHA for fish oil. We hypothesize that the intervention effects may be larger among those with below-median baseline levels, and will examine treatment effects by quartiles of these biomarkers. The biomarker data will be analyzed as a case-cohort study using methods for proportional hazards regression.[77] Because of the frequency matching by age and gender, appropriate stratum-specific weighting of the observations will be employed.[78] We also have a prior interest in the effects of the vitamin D intervention within groups defined by race/ethnicity and skin

pigmentation, and by BMI. In addition, we will evaluate effect modification by age and gender, as well as by sunlight exposure, calcium and phosphorus intakes from the FFQ (as these nutrients affect vitamin D bioavailability[79]), and baseline risk factors for cancer and CVD. The latter interaction effects will be interpreted cautiously, as hypothesis generating. We will also examine as a prespecified secondary analysis the association among compliers, in which participants' follow-up time will be censored when they are no longer compliant with taking at least two-thirds of their assigned active agents or placebos. Finally, we will examine whether treatment effects vary over time and duration of treatment by examining survival plots and interactions with time. We will additionally conduct analyses for the primary endpoints of cancer and cardiovascular disease excluding events occurring in Year 1, as well as in Years 1 and 2, of follow-up. There may be a latent effect on both outcomes, depending on the mechanisms by which these agents act.

We will also compare the incidence of potential side effects in the active *v.* placebo groups for each agent, including the incidence of kidney stones with vitamin D assignment (cf. WHI calcium-vitamin D findings[44]) and the incidence of GI symptoms, skin abnormalities, and bleeding with fish oil assignment (cf. JELIS findings[41]).

B. POWER CALCULATIONS

To calculate power, the following assumptions were made: (1) a 2x2 factorial trial in 12,500 men aged ≥50 and 12,500 women aged ≥55; (2) independent and equal allocation of participants to each treatment; (3) an age distribution based on that observed at baseline in our past trials for men aged ≥50 and women aged ≥55; (4) age-specific event rates based on the observed rates in the first 5 years of the PHS II for men and the WHS for women; (5) trial follow-up of 5 years, with little loss to follow-up as achieved in our past trials; and (6) compliance similar to that in published trials upon which our estimated rate ratio (RR) reductions are based. The cited reductions are thus the observed effects we would see in the trial. The corresponding 'true' RR is given assuming an average compliance of 80%. Power is given for a two-sided test using a logrank analysis[80] with a significance level of 0.05.

Cancer: Assuming that only one agent is effective, with 5 years of treatment and follow-up we will have 93% power to detect an observed RR of 0.85 for the primary endpoint of total cancer incidence (Table 1). For prostate cancer, we will have 93% power to detect a 25% risk reduction. For breast cancer, we will have 86% power to detect a 35% risk reduction. For colorectal cancer, we will have 77% power to detect a 35% risk reduction and 88% power to detect a 40% risk reduction. For cancer mortality, we will have 86% power to detect a 30% risk reduction. If both agents are effective in preventing cancer but act independently, power would be reduced slightly due to a somewhat smaller overall

Table 1. Power for effects of a single agent on cancer in VITAL, a 2x2 factorial trial of 12,500 men aged≥50 and 12,500 women aged≥55, with 5 years of follow-up

Observed RR[a]	True RR[b]	Total Cancer	Cancer Mortality	Colorectal Cancer	Breast Cancer (Women)	Prostate Cancer (Men)
0.90	0.875	61.4	-	-	-	-
0.85	0.812	92.7	-	-	-	-
0.80	0.750	99.6	50.5	31.7	38.7	77.0
0.75	0.687	>99.9	70.6	46.8	56.3	92.8
0.70	0.625	>99.9	86.1	62.9	73.2	98.6
0.65	0.560	>99.9	95.0	77.3	86.3	99.8
0.60	0.500	>99.9	98.7	88.2	94.3	>99.9

a. Observed RR=intent-to-treat RR, including noncompliant participants. Compliance is assumed to be 80%.
b. True RR=that with perfect compliance.
RR, rate ratio.

number of events. If the agents interact, however, power will be affected to the extent of the interaction. Should the agents act synergistically, power would increase, as illustrated in Table 2 for the outcome of total cancer.

For example, if the effect of each agent alone is a reduction of 10%, but in combination the effect is stronger, with an additional 10% decrease, the RR comparing the combined group to the all placebo group would be 0.73, versus 0.81 with additive effects (on the multiplicative scale). Power for the main effect of each agent would then increase to 88% (or higher with greater synergy). The trial would have power to detect moderate-to-large interactions, as shown in Table 2. Should the agents interact in a subadditive fashion, power would be reduced to the extent of the subadditivity. Power to explore effect modification by baseline blood levels of 25(OH)D and EPA+DHA among those above and below the median level in the cohort will be lower because we anticipate that about 60% of participants will provide blood specimens.

Table 2. Power for effects of a single agent on CVD in VITAL, a 2x2 factorial trial of 12,500 men aged ≥50 and 12,500 women aged ≥55, with 5 years of follow-up

Observed RR[a]	True RR[b]	Major CVD[c]	Total CVD[d]	CVD Mortality	MI	Stroke
0.90	0.875	41.6	62.2	-	-	-
0.85	0.812	75.6	93.2	-	-	-
0.80	0.750	94.8	99.6	53.7	60.1	60.3
0.75	0.687	99.5	>99.9	74.0	80.2	80.3
0.70	0.625	>99.9	>99.9	88.7	92.8	92.8
0.65	0.560	>99.9	>99.9	96.3	98.2	98.2
0.60	0.500	>99.9	>99.9	99.2	99.7	99.7

a. Observed RR=intent-to-treat RR, including noncompliant participants. Compliance is assumed to be 80%.
b. True RR=that with perfect compliance.
c. Major CVD=myocardial infarction, stroke, and CVD mortality
d. Total CVD=myocardial infarction, stroke, CVD mortality, and coronary revascularization (coronary artery bypass grafting or percutaneous coronary intervention)
CVD, cardiovascular disease; *MI*, myocardial infarction; *RR*, rate ratio.

Power has been shown for a given observed reduction in risk, assuming compliance similar to that seen in current randomized trials such as the WHS and PHS. For total cancer, our power will be excellent with a 5-year follow-up even if compliance is lower.

CVD: As shown in Table 3, assuming that only one agent is effective, with 5 years of treatment and follow-up we will have 95% power to detect an observed RR of 0.80 for the primary composite endpoint of MI, stroke, and cardiovascular mortality. For the secondary composite endpoint of MI, stroke, cardiovascular mortality, and coronary revascularization, we will have over 99% power to detect a 20% reduction and 93% power to detect a 15% reduction. With a RR of 0.70 we will have 89% power for cardiovascular mortality, and we will have over 80% power to detect an RR of 0.75 for MI and stroke. If both agents are effective in preventing CVD but act independently, power would be reduced only slightly due to

Table 3. Power for interaction effects on total cancer in VITAL, a 2x2 factorial trial of 12,500 men aged ≥50 and 12,500 women aged ≥55, with 5 years of follow-up

RR Single agent[a]	Interaction[b]	RR Both agents	Power Main Effect	Power Interaction
0.90	1.0	0.81	59.1	
	0.9	0.73	88.4	
	0.8	0.65	98.5	60.7
	0.7	0.57	99.9	93.6
0.85	1.0	0.72	90.6	
	0.9	0.65	98.6	
	0.8	0.58	99.9	57.8
	0.7	0.51	>99.9	92.0
0.80	1.0	0.64	99.2	
	0.9	0.58	99.9	
	0.8	0.51	>99.9	54.7
	0.7	0.45	>99.9	89.9

a. RR = intent-to-treat RR, including noncompliant participants (assuming 80% compliance). Represents the effect among those not assigned to the other intervention and assumes the same effect for both agents.
b. The interaction is the RR for the combined group divided by the product of risks for the two separate groups—i.e., RRint=RRboth/(RR vitamin D alone*RR omega-3 fatty acids alone). An interaction=1 implies additive effects (no interaction).
RR, rate ratio.

a smaller overall number of events. If the agents act synergistically, power would increase, as described for cancer. If the agents interact in subadditive fashion, power would be reduced to the extent of the subadditivity.

X. REFERENCES

1. Davis CD, Hartmuller V, Freedman DM, Hartge P, Picciano MF, Swanson CA, Milner JA. Vitamin D and cancer: current dilemmas and future needs. Nutr Rev 2007; 65:S71-4.
2. Institute of Medicine. Dietary Reference Intakes for Calcium and Vitamin D. Washington DC: National Academies Press, 2011.
3. Giovannucci E. Strengths and limitations of current epidemiologic studies: vitamin D as a modifier of colon and prostate cancer risk. Nutr Rev 2007; 65:S77-9.
4. Looker AC, Dawson-Hughes B, Calvo MS, Gunter EW, Sahyoun NR. Serum 25-hydroxyvitamin D status of adolescents and adults in two seasonal subpopulations from NHANES III. Bone 2002; 30:771-7.
5. Holick MF. Vitamin D deficiency. N Engl J Med 2007; 357:266-81.
6. Harris SS. Vitamin D and African Americans. J Nutr 2006; 136:1126-9.
7. Moore CE, Murphy MM, Holick MF. Vitamin D intakes by children and adults in the United States differ among ethnic groups. J Nutr 2005; 135:2478-85.
8. Martins D, Wolf M, Pan D, Zadshir A, Tareen N, Thadhani R, Felsenfeld A, Levine B, Mehrotra R, Norris K. Prevalence of cardiovascular risk factors and the serum levels of 25-hydroxyvitamin D in the United States: data from the Third National Health and Nutrition Examination Survey. Arch Intern Med 2007; 167:1159-65.
9. Harris SS, Dawson-Hughes B. Reduced sun exposure does not explain the inverse association of 25-hydroxyvitamin D with percent body fat in older adults. J Clin Endocrinol Metab 2007; 92:3155-7.
10. Hedley AA, Ogden CL, Johnson CL, Carroll MD, Curtin LR, Flegal KM. Prevalence of overweight and obesity among US children, adolescents, and adults, 1999-2002. JAMA 2004; 291:2847-50.
11. Ogden CL, Carroll MD, Curtin LR, McDowell MA, Tabak CJ, Flegal KM. Prevalence of overweight and obesity in the United States, 1999-2004. JAMA 2006; 295:1549-55.
12. Kris-Etherton PM, Harris WS, Appel LJ. Fish consumption, fish oil, omega-3 fatty acids, and cardiovascular disease. Circulation 2002; 106:2747-57.
13. NIH Office of Dietary Supplements, National Heart Lung and Blood Institute. Working Group Report on Future Clinical Research Directions on Omega-3 Fatty Acids and Cardiovascular Disease. June 2, 2004. Bethesda MD. Available at: http://www.nhlbi.nih.gov/meetings/workshops/omega-3/omega-3-rpt.htm Accessed on March 28, 2008.
14. Stipp D. Fish-oil doses can be hard to swallow. Wall Street Journal: January 8, 2008; Page D1-D2.
15. Gosnell M. Top 100 science stories of 2007: #8: Can vitamin D save your life? Discover: December 12, 2007. Available at http://discovermagazine.com/2008/jan/year-in-science-2007/can-vitamin-d-save-your-life Accessed March 25, 2008.
16. Guthrie C. The 10 biggest medical breakthroughs. #10: Benefits of vitamin D. Time: December 24, 2007. Available at: http://www.time.com/time/specials/2007/top10/article/0,30583,1686204_1686252_1690393,00.html Accessed March 25, 2008.
17. Harvard Health Letter. The top 10 health stories of 2006. Harv Health Lett 2006; 32:1-3.

18. Frisinger C. New year, new you: 8 simple rules for a healthier 2008. #1. Get more vitamin D. Fort Worth Star-Telegram (Texas): January 1, 2008; Page E1.
19. Brody JE. An oldie vies for nutrient of the decade. New York Times: February 19, 2008; Page F7.
20. Stampfer MJ. Vitamin D in the spotlight. Newsweek: December 11, 2006. Available at: http://www.newsweek.com/id/43998 Accessed March 25, 2008.
21. Peng T. Don't forget your vitamins. Newsweek: March 24, 2008; Page 78.
22. Rosenfeld I. We may need more vitamin D. Parade Magazine: January 29, 2006; Page 15.
23. Rhodes P. Vitamin D essentials: what you need to know about this important nutrient and where best to obtain it. Cooking Light 2008; 22:44-50.
24. Foreman J. Is there any way to tell whether you are getting enough vitamin D? Boston Globe: February 4, 2008; Page C2.
25. Harvard Health Letter. A more D-manding diet. Some experts say we should have a lot more vitamin D in our diets because it's protective against several diseases. Harv Health Lett 2007; 32:3.
26. Simopoulos AP, Robinson J. The Omega Diet: The Lifesaving Nutritional Program Based on the Diet of the Island of Crete. New York: HarperCollins, 1999.
27. Sears B. The Omega Rx Zone: The Miracle of the New High-Dose Fish Oil. New York: HarperCollins, 2004.
28. Kaufman M. Fish oil may aid against manic depression. Washington Post, 1999:Z07.
29. Foreman J. Fatty acid imbalance hurts our health. Boston Globe: February 10, 2004.
30. Bakalar N. Fish oil linked to lower Alzheimer's risk. New York Times: November 14, 2006.
31. Munoz SS. Shopping around: DHA-enhanced foods. Wall Street Journal: March 27, 2008; Page D2.
32. Canadian Cancer Society. Canadian Cancer Society Announces Vitamin D Recommendation. Available at: http://www.cancer.ca/ccs/internet/mediareleaselist/0,,3172_1613121606_1997621989_langId-en.html. Accessed May 6, 2008.
33. Vieth R, Bischoff-Ferrari H, Boucher BJ, Dawson-Hughes B, Garland CF, Heaney RP, Holick MF, Hollis BW, Lamberg-Allardt C, McGrath JJ, Norman AW, Scragg R, Whiting SJ, Willett WC, Zittermann A. The urgent need to recommend an intake of vitamin D that is effective. Am J Clin Nutr 2007; 85:649-50.
34. Davis CD, Dwyer JT. The "sunshine vitamin": benefits beyond bone? J Natl Cancer Inst 2007; 99:1563-5.
35. Bardia A, Tleyjeh IM, Cerhan JR, Sood AK, Limburg PJ, Erwin PJ, Montori VM. Efficacy of antioxidant supplementation in reducing primary cancer incidence and mortality: systematic review and meta-analysis. Mayo Clin Proc 2008; 83:23-34.
36. Mosca L, Banka CL, Benjamin EJ, Berra K, Bushnell C, Dolor RJ, Ganiats TG, Gomes AS, Gornik HL, Gracia C, Gulati M, Haan CK, Judelson DR, Keenan N, Kelepouris E, Michos ED, Newby LK, Oparil S, Ouyang P, Oz MC, Petitti D, Pinn VW, Redberg RF, Scott R, Sherif K, Smith SC, Jr., Sopko G, Steinhorn RH, Stone NJ, Taubert KA, Todd BA, Urbina E, Wenger NK. Evidence-based guidelines for cardiovascular disease prevention in women: 2007 update. Circulation 2007; 115:1481-501.
37. Kris-Etherton PM, Lichtenstein AH, Howard BV, Steinberg D, Witztum JL. Antioxidant vitamin supplements and cardiovascular disease. Circulation 2004; 110:637-41.
38. Lee IM, Cook NR, Gaziano JM, Gordon D, Ridker PM, Manson JE, Hennekens CH, Buring JE. Vitamin E in the primary prevention of cardiovascular disease and cancer: the Women's Health Study: a randomized controlled trial. JAMA 2005; 294:56-65.

39. Miller ER, 3rd, Pastor-Barriuso R, Dalal D, Riemersma RA, Appel LJ, Guallar E. Meta-analysis: high-dosage vitamin E supplementation may increase all-cause mortality. Ann Intern Med 2005; 142:37-46.
40. Bjelakovic G, Nikolova D, Gluud LL, Simonetti RG, Gluud C. Mortality in randomized trials of antioxidant supplements for primary and secondary prevention: systematic review and meta-analysis. JAMA 2007; 297:842-57.
41. Yokoyama M, Origasa H, Matsuzaki M, Matsuzawa Y, Saito Y, Ishikawa Y, Oikawa S, Sasaki J, Hishida H, Itakura H, Kita T, Kitabatake A, Nakaya N, Sakata T, Shimada K, Shirato K. Effects of eicosapentaenoic acid on major coronary events in hypercholesterolaemic patients (JELIS): a randomised open-label, blinded endpoint analysis. Lancet 2007; 369:1090-8.
42. Gruppo Italiano per lo Studio della Sopravvivenza nell'Infarto miocardico. Dietary supplementation with n-3 polyunsaturated fatty acids and vitamin E after myocardial infarction: results of the GISSI-Prevenzione trial. Gruppo Italiano per lo Studio della Sopravvivenza nell'Infarto miocardico. Lancet 1999; 354:447-55.
43. Lappe JM, Travers-Gustafson D, Davies KM, Recker RR, Heaney RP. Vitamin D and calcium supplementation reduces cancer risk: results of a randomized trial. Am J Clin Nutr 2007; 85:1586-91.
44. Wactawski-Wende J, Kotchen JM, Anderson GL, Assaf AR, Brunner RL, O'Sullivan MJ, Margolis KL, Ockene JK, Phillips L, Pottern L, Prentice RL, Robbins J, Rohan TE, Sarto GE, Sharma S, Stefanick ML, Van Horn L, Wallace RB, Whitlock E, Bassford T, Beresford SA, Black HR, Bonds DE, Brzyski RG, Caan B, Chlebowski RT, Cochrane B, Garland C, Gass M, Hays J, Heiss G, Hendrix SL, Howard BV, Hsia J, Hubbell FA, Jackson RD, Johnson KC, Judd H, Kooperberg CL, Kuller LH, LaCroix AZ, Lane DS, Langer RD, Lasser NL, Lewis CE, Limacher MC, Manson JE. Calcium plus vitamin D supplementation and the risk of colorectal cancer. N Engl J Med 2006; 354:684-96.
45. Hathcock JN, Shao A, Vieth R, Heaney R. Risk assessment for vitamin D. Am J Clin Nutr 2007; 85:6-18.
46. Lang JM, Buring JE, Rosner B, Cook N, Hennekens CH. Estimating the effect of the run-in on the power of the Physicians' Health Study. Stat Med 1991; 10:1585-93.
47. Talwar SA, Aloia JF, Pollack S, Yeh JK. Dose response to vitamin D supplementation among postmenopausal African American women. Am J Clin Nutr 2007; 86:1657-62.
48. Rimm EB, Giovannucci EL, Stampfer MJ, Colditz GA, Litin LB, Willett WC. Reproducibility and validity of an expanded self-administered semiquantitative food frequency questionnaire among male health professionals. Am J Epidemiol 1992; 135:1114-26.
49. Feskanich D, Rimm EB, Giovannucci EL, Colditz GA, Stampfer MJ, Litin LB, Willett WC. Reproducibility and validity of food intake measurements from a semiquantitative food frequency questionnaire. J Am Diet Assoc 1993; 93:790-6.
50. Salvini S, Hunter DJ, Sampson L, Stampfer MJ, Colditz GA, Rosner B, Willett WC. Food-based validation of a dietary questionnaire: the effects of week-to-week variation in food consumption. Int J Epidemiol 1989; 18:858-67.
51. Jacques PF, Sulsky SI, Sadowski JA, Phillips JC, Rush D, Willett WC. Comparison of micronutrient intake measured by a dietary questionnaire and biochemical indicators of micronutrient status. Am J Clin Nutr 1993; 57:182-9.
52. Feskanich D, Willett WC, Colditz GA. Calcium, vitamin D, milk consumption, and hip fractures: a prospective study among postmenopausal women. Am J Clin Nutr 2003; 77:504-11.
53. Hunter DJ, Rimm EB, Sacks FM, Stampfer MJ, Colditz GA, Litin LB, Willett WC. Comparison of measures of fatty acid intake by subcutaneous fat aspirate, food

frequency questionnaire, and diet records in a free-living population of US men. Am J Epidemiol 1992; 135:418-27.
54. Bischoff-Ferrari HA, Giovannucci E, Willett WC, Dietrich T, Dawson-Hughes B. Estimation of optimal serum concentrations of 25-hydroxyvitamin D for multiple health outcomes. Am J Clin Nutr 2006; 84:18-28.
55. Dawson-Hughes B, Heaney RP, Holick MF, Lips P, Meunier PJ, Vieth R. Estimates of optimal vitamin D status. Osteoporos Int 2005; 16:713-6.
56. Giovannucci E, Liu Y, Rimm EB, Hollis BW, Fuchs CS, Stampfer MJ, Willett WC. Prospective study of predictors of vitamin D status and cancer incidence and mortality in men. J Natl Cancer Inst 2006; 98:451-9.
57. Aloia JF, Patel M, DiMaano R, Li-Ng M, Talwar SA, Mikhail M, Pollack S, Yeh JK. Vitamin D intake to attain a desired serum 25-hydroxyvitamin D concentration. Am J Clin Nutr 2008;87:1952-1958
58. Harris WS. International recommendations for consumption of long-chain omega-3 fatty acids. J Cardiovasc Med (Hagerstown) 2007; 8 Suppl 1:S50-2.
59. Lee JH, O'Keefe JH, Lavie CJ, Marchioli R, Harris WS. Omega-3 fatty acids for cardioprotection. Mayo Clin Proc 2008; 83:324-32. [Docket No. 86G-0289], 1997.
60. Agency for Healthcare Research and Quality. Effects of Omega-3 Fatty Acids on Cardiovascular Disease. Evidence Report/Technology Assessment Number 94. AHRQ Pub. No. 04-E009-1. 2004. Available at: http://www.ahrq.gov/clinic/epcsums/o3cardsum.htm Accessed March 28, 2008.
61. Simopoulos AP. Evolutionary aspects of diet, the omega-6/omega-3 ratio and genetic variation: nutritional implications for chronic diseases. Biomed Pharmacother 2006; 60:502-7.
62. Kris-Etherton PM, Taylor DS, Yu-Poth S, Huth P, Moriarty K, Fishell V, Hargrove RL, Zhao G, Etherton TD. Polyunsaturated fatty acids in the food chain in the United States. Am J Clin Nutr 2000; 71:179S-88S.
63. Harris WS. The omega-6/omega-3 ratio and cardiovascular disease risk: uses and abuses. Curr Atheroscler Rep 2006; 8:453-9.
64. Stanley JC, Elsom RL, Calder PC, Griffin BA, Harris WS, Jebb SA, Lovegrove JA, Moore CS, Riemersma RA, Sanders TA. UK Food Standards Agency Workshop Report: the effects of the dietary n-6:n-3 fatty acid ratio on cardiovascular health. Br J Nutr 2007; 98:1305-10.
65. Harris WS, Poston WC, Haddock CK. Tissue n-3 and n-6 fatty acids and risk for coronary heart disease events. Atherosclerosis 2007; 193:1-10.
66. Mozaffarian D, Ascherio A, Hu FB, Stampfer MJ, Willett WC, Siscovick DS, Rimm EB. Interplay between different polyunsaturated fatty acids and risk of coronary heart disease in men. Circulation 2005; 111:157-64.
67. Department of Health and Human Services, U.S. Food and Drug Administration. Substances affirmed as generally recognized as safe: menhaden oil. Federal Register. June 5, 1997. Vol 62, No 108: pp 30751-30757. 21 CFR Part 184 [Docket No. 86G-0289], 1997.
68. Bays HE. Safety considerations with omega-3 fatty acid therapy. Am J Cardiol 2007; 99:35C-43C.
69. Satterfield S, Greco PJ, Goldhaber SZ, Stampfer MJ, Swartz SL, Stein EA, Kaplan L, Hennekens CH. Biochemical markers of compliance in the Physicians' Health Study. Am J Prev Med 1990; 6:290-4.
70. Fritz AG, Percy C, Jack A, Shanmugaratham K, Sobin LH, Parkin MD, Whelan S. International Classification of Diseases for Oncology (ICD-O), Third Edition. Geneva: World Health Organization, 2000.

71. Thygesen K, Alpert JS, White HD, Joint ESC/ACCF/AHA/WHF Task Force for the Redefinition of Myocardial Infarction. Universal definition of myocardial infarction. Circulation 2007; 116:2634-53.
72. Adams HP, Jr., Bendixen BH, Kappelle LJ, Biller J, Love BB, Gordon DL, Marsh EE, 3rd. Classification of subtype of acute ischemic stroke. Definitions for use in a multicenter clinical trial. TOAST. Trial of Org 10172 in Acute Stroke Treatment. Stroke 1993; 24:35-41.
73. Cox DR. Regression models and life-tables. J Royal Statist Soc B 1972; 34:187-220.
74. Gooley TA, Leisenring W, Crowley J, Storer BE. Estimation of failure probabilities in the presence of competing risks: new representations of old estimators. Stat Med 1999; 18:695-706.
75. Putter H, Fiocco M, Geskus RB. Tutorial in biostatistics: competing risks and multi-state models. Stat Med 2007; 26:2389-430.
76. Fine JP, Gray RJ. A proportional hazards model for the subdistribution of a competing risk. J Am Stat Assoc 1999; 94:496-509.
77. Barlow WE. Robust variance estimation for the case-cohort design. Biometrics 1994; 50:1064-72.
78. Borgan O, Langholz B, Samuelsen SO, Goldstein L, Pogoda J. Exposure stratified case-cohort designs. Lifetime Data Anal 2000; 6:39-58.
79. Holick MF. Vitamin D for health and in chronic kidney disease. Semin Dial 2005; 18:266-75
80. Freedman LS. Tables of the number of patients required in clinical trials using the logrank test. Stat Med 1982; 1:121-9.
81. Brasky TM, Darke AK, Song X, et al. Plasma phospholipid fatty acids and prostate cancer risk in the SELECT trial. J Natl Cancer Inst 2013; 105:1132-41.
82. Haybittle JL. Repeated assessment of results in clinical trials of cancer treatment. Br J Radiol 1971; 44:793-7.
83. Peto R, Pike MC, Armitage P, Breslow NE, Cox DR, Howard SV, Mantel N, McPherson K, Peto J, Smith PG. Design and analysis of randomized clinical trials requiring prolonged observation of each patient. I. Introduction and design. Br J Cancer 1976; 34:585-612.
84. Geller NL, Pocock SJ. Interim analyses in randomized clinical trials: ramifications and guidelines for practitioners. Biometrics 1987; 43:213-23.
85. O'Brien PC, Fleming TR. A multiple testing procedure for clinical trials. Biometrics 1979; 35:549-56.
86. DeMets DL, Lan KK. Interim analysis: the alpha spending function approach. Stat Med 1994; 13:1341-52.
87. Pocock SJ. Current controversies in data monitoring for clinical trials. Clin Trials 2006; 3:513-21.

September 2018

The VITamin D and OmegA-3 TriaL (VITAL): Summary of Changes to the Statistical Analysis Plan:

January 28, 2010 (v. 2) was the date that the first full protocol was approved by the IRB. A copy of the data analysis plan in this protocol is provided. (Our initial IRB application, submitted in June 2009, contained a draft protocol.)

June 8, 2011 (v.5)

- Eligible age range was lowered to 50 years or older for males and 55 or older for females, to reflect the younger age of development of heart disease and cancer among underrepresented minorities, and thus facilitate minority recruitment.

December 1, 2011 (v. 6)

- Revised power calculations based on lowering the eligibility age range on 6/8/11.

December 3, 2012 (v. 8)

- Provided new power calculations based upon a study population of 25,000

September 17, 2013 (v. 11)

- Specified that the projected randomized population would be a maximum of 26,500

September 13, 2017 (v. 22)

- Specified that, at the conclusion of the randomized trial, we will send participants two additional annual questionnaires to collect supplemental study data during the post-intervention phase.

November 30, 2017 (v. 23) is the current protocol. A copy of the data analysis plan in this version is provided.

- Clarified that we will additionally conduct analyses excluding events occurring in year 1 and years 1-2 of follow-up (our original protocol on 1/28/10 [v.2] specified: "Finally, we will examine whether treatment effects vary over time and duration of treatment by examining survival plots and interactions with time. There may be a latent effect on cancer incidence, depending on the stage of carcinogenesis during which these agents act." In the 11/30/17 protocol, we formalized this as analyses excluding events during year 1 and years 1-2 of follow-up.)

- Data analysis section amplified: specified sensitivity analyses by compliance, with participants' follow-up time censored when they are no longer compliant with taking at least two-thirds of their assigned active agents or placebos (as in our previous trials).

NOTE: VITAL investigators were unblinded to randomization treatment codes on December 6, 2017 prior to a teleconference with VITAL DSMB members. All of the above protocol changes were made PRIOR to the investigator unblinding on December 6, 2017. The intervention phase of VITAL ended on December 31, 2017.

TRIAL PROTOCOL AND STATISTICAL ANALYSIS PLAN (SAP)

Supplemental Vitamin D and Incident Fractures in Midlife and Older Adults

This supplement contains the following items:

1. Original protocol. There were no changes made.
2. Original statistical analysis plan. There were no changes made.

I. BACKGROUND

Vitamin D: Vitamin D is synthesized as a pro-hormone in the skin in response to ultraviolet B (UVB) radiation and is also absorbed from the gastrointestinal tract; both sources are activated in the liver to 25-hydroxyvitamin D [25(OH)D] and in the kidney to 1,25-dihydroxyvitamin D. Emerging data show that extra-renal activation of vitamin D occurs in a number of tissues, with potential autocrine and paracrine effects on various tissues, including human marrow stromal cells.[1-5] Circulating serum 25(OH)D levels indicate vitamin D status. Steady-state 25(OH)D levels are lower when there is reduced generation of vitamin D in the skin (higher latitude, winter/late fall seasons), and when there is decreased vitamin D intake or malabsorption.[6, 7] While concentrations of 25(OH)D > 75-80 nmol/L are considered optimal, this threshold may change as we learn more about the effects of higher doses of vitamin D on musculoskeletal and other outcomes.[8-10]

Clinical and epidemiological studies show that inadequate vitamin D levels are common. Elders are at greater risk as their skin has a reduced capacity to produce vitamin D, and they often have limited solar exposure.[11] According to data from National Health and Nutrition Examination Survey III (NHANES III), the prevalence of vitamin D insufficiency in the U.S. was 46% and 35% in white women and men, respectively and 89% and 82% in black women and men.[12] Black individuals are at higher risk than white individuals because darkly pigmented skin is less able to generate vitamin D in response to UVB radiation and they tend to have lower vitamin D intakes.[13, 14] Overweight individuals are also at greater risk, possibly from decreased availability of this fat-soluble vitamin.[15, 16] Moreover, 50% of postmenopausal women with osteoporosis have vitamin D insufficiency.[17] In the osteoporotic fractures in men (MrOS) study, 72% of older men had vitamin D insufficiency.[18, 19] In studies of elders with hip fractures, 96% of women and men in Baltimore and Boston, and in a multinational study 90% had vitamin D insufficiency.[20-22] In the WHI-Observational (WHI-OS) study, low 25(OH)D levels (<47 nmol/L) were associated with a 40% increased hip fracture risk compared with higher levels of 25(OH)D.[23] Thus, vitamin D insufficiency is a major public health problem associated with fractures that may be modified by high doses of supplemental vitamin D.

Vitamin D and Fractures: Data from observational studies and randomized clinical trials (RCTs) suggest the protective effects of vitamin D supplements against fracture, but the evidence is inconsistent.[24, 25], [26] In a 2005 meta-analysis of 5 RCTs for hip fractures and 7 RCTs for non-vertebral fractures, supplementation with oral vitamin D3 700-800 IU/d appeared to reduce the risk of any non-vertebral fracture by 23% and hip fracture

by 26% in older adults, but 400 IU/d was not sufficient to elicit fracture prevention.[27] A meta-regression analysis revealed anti-fracture effects at higher achieved circulating 25(OH) vitamin D levels. A meta-analysis of RCTs in 2009 further supports a benefit of vitamin D on fracture reduction. Among studies of women or men > 65 years treated with vitamin D with or without calcium, the pooled analysis of 12 double-blind RCTs for non-vertebral fractures and 8 RCTs for hip fractures showed a dose-dependent effect of vitamin D on fractures, with daily doses above 400 IU/d reducing non-vertebral fractures by 20% and hip fractures by 19% (from 5 trials).[28] An analysis of community-dwelling older adults showed that higher doses of vitamin D reduced non-vertebral fractures by 29% and hip fractures by 21%.[28] Although, recent meta-analyses indicate that the anti-fracture efficacy of vitamin D is most effective at doses of vitamin >700 IU/d and the higher achieved 25-(OH)D level, primary prevention RCTs with 2000IU/d of vitamin D are lacking.

Rationale for Ancillary Study of High-dose Vitamin D and Prevention of Fractures

There has been considerable controversy regarding the effects of vitamin D on fractures and falls. Study designs according to an evidence-based review are inconsistent, often without analysis of baseline or achieved vitamin D levels, gender or race/ethnicity, or effects of vitamin D alone independent from those combined with calcium supplementation[24, 25] In an overview of the NIH conference on "Vitamin D and Health in the 21st Century: An Update," an evidence-based report indicated that "the available evidence on the relation of 25(OHD), functional health, and adverse outcomes has significant limitations because most studies have been short term, have failed to consider important confounders such as baseline vitamin D status, BMI, and did not study key populations."[24, 29] In an editorial, Dawson-Hughes and other panelists on the optimal vitamin D dose for bone concluded that "more data are needed on the utility of vitamin D doses beyond 20 mcg (800 IU)/day."[10] Previously, we found occult vitamin D deficiency among postmenopausal women with acute hip fractures.[22] In subsequent analyses, vitamin insufficiency [25(OH)D level < 80 nmol/L] was found in 96% of 110 hip fracture subjects from Boston and Baltimore, and 38% had extremely low levels (< 22.5 mmol/L).[20] Other studies affirmed the high incidence of low vitamin D levels in subjects with hip fractures including recent evidence of vitamin D insufficiency in 90% of hip fracture subjects in a multi-national zoledronic acid study.[21] Prior studies from overseas had indicated low vitamin D levels in patients with hip fractures, but this was not expected in the U.S. because of vitamin D fortification of milk.[30, 31] Even when compared with osteoporotic women without hip fractures, those with hip fractures had significantly more vitamin D-deficiency and secondary hyperparathyroidism.[32] Those findings raised awareness of the prevalence of vitamin D-deficiency in the U.S.[33-35] We also found that, in a series of postmenopausal women with advanced osteoarthritis, 25% had occult osteoporosis and 46% were vitamin D-deficient.[36] Our findings reject the hypothesis that osteoarthritic women

are protected against bone loss, but the impact of vitamin D status on either condition is not understood. From our evidence on the high prevalence of vitamin D deficiency in hip fracture patients, Dr. LeBoff and Dr. Glowacki worked to advance fracture care at Brigham and Women's Hospital (BWH) with implementation of an interdisciplinary fracture pathway between Endocrinology and Orthopedic Surgery now called the Brigham Fracture Intervention Team Initiative or "BFit".[35, 37, 38] A greater understanding of the effects of high dose daily vitamin D on fracture reduction has the potential to transform fracture care in the U.S. To understand the effects of high-dose daily vitamin D supplementation on fractures, double-blind RCTS with large sample sizes and long duration designed specifically to assess fractures and falls are needed. To our knowledge, no such trials in the U.S. are either ongoing or have been completed.

Omega-3 Fatty Acids and Skeletal Health: Vitamin D, calcium, and protein are among the dietary essentials for skeletal health. Fatty fishes are the predominant source of the omega-3 (n-3) fatty acids. Concerns about recent changes in the dietary balance of omega-6 (n-6) to n-3 fatty acids in the diet [39] have raised interest in their cellular and skeletal effects. Information about the skeletal effects of fish oil is available from animal studies and some RCTs. Dietary fish oil protected mice from bone loss following ovariectomy, and also prevented the elevation of RANKL seen in T-cells from ovariectomized mice supplemented with corn oil.[40] That study supports the view that one of the mechanisms by which dietary n-3 fatty acids reduces bone loss in ovariectomized mice is by inhibition of osteoclast generation and activation. Other research shows direct evidence of beneficial effects on bone formation. Dietary fish oil protected retired breeder rats from bone loss following ovariectomy, with a marked increase in mineral apposition rate.[41]

In many animal studies, n-3 fatty acids or a low ratio of n-6 to n-3 fatty acids show a positive influence on bone. Bone loss was inhibited by EPA in ovariectomized rats on a low-calcium diet.[42] Both EPA and DHA prevented increases in bone fragility in diabetic rats; and EPA prevented osteopenia even in diabetic rats fed a low-zinc diet, which was used as a potent accelerator of diabetic osteopenia.[43] Subsequently, it was shown that n-3 polyunsaturated fatty acid (PUFA) deficiency caused severe osteoporosis in rats. Further, when deficient animals were replenished with n-3 PUFA, the ratio of n-3 to n-6 PUFA in bone compartments was restored and the process of bone degradation was reversed.[44] The beneficial effects of n-3 fatty acids appear to be associated with down regulation of PGE2 formation with a net enhancement of bone formation and diminished resorption.[45] Although not all studies show beneficial skeletal effects of n-3 in ovariectomized rats[46] it has been suggested that study design and the type of fatty acid in the diet may be more important than the overall ratio of n-3 to n-6 PUFA.[47] A recent study with intact female mice showed that long-term intake of n-3 fatty acids, especially EPA, improved structural and mechanical properties of cortical

bone in the femur, without detectable effects on age-related loss of trabecular bone or BMD.[48] A recent review concluded that most animal studies confirm the beneficial effects of n-3 fatty acids on bone health.[49] Differences in lipoprotein metabolism between animals and humans may limit understanding the biochemical basis of some effects of n-3 fatty acids and observed differences between marine and plant n-3 fatty acids.[50, 51]

RCTs in humans are limited. Results from a small number of studies in humans are inconclusive and vary in type, concentration, and dosing of n-3 fatty acids. In a randomized trial of 40 patients with osteoporosis, subjects taking a supplement rich in n3 PUFA showed better calcium absorption and increased markers of bone formation, while those taking a placebo did not.[52] In a clinical trial of 65 elderly women whose diet was low in calcium, supplementation with a properly balanced ratio of n-6 and n-3 PUFA plus calcium decreased bone turnover and increased BMD. Subjects receiving linoleic acid +EPA treatment for 18-months showed increases of 3.1% in lumbar spine density and 4.7% in femoral BMD.[53] In contrast, 12-month trials with a combination supplement of n-6 PUFA, n-3 PUFA, and calcium in pre- and post-menopausal women failed to affect BMD,[54] possibly attributable to the trial's length, and its use of only total-body BMD measurements. One investigation suggested that PUFAs are actually harmful to bone in peri-menopausal women.[55] To control for confounders due to background diets, a three-period cross-over feeding trial was designed in adults with 3 test diets: an average American diet and two high-PUFA diets with different ratios of n6/n-3 fatty acids.[56] Bone turnover was reduced and bone formation was maintained following the diet with the lowest n-6/n-3 composition. In a recent observational study among elders in an assisted living facility, greater self-reported intakes of n-3 fatty acids were associated with higher BMD.[57] There was no association between physical function and intakes of n-3 fatty acids. These data suggest a possible effect of omega-3 fatty acids on bone health, but definitive studies are necessary. This ancillary study will test whether omega-3 fatty acids reduce fracture.

<u>Significance of this Ancillary Study</u>: The proposed ancillary study is significant because 1) with the growing older population it is important to investigate modifiable factors affecting fractures and bone loss; 2) high dose vitamin D (2000 IU/d) has not been tested for its skeletal effects in a large primary prevention trial; 3) small studies suggest that omega-3 fatty acids may also have beneficial skeletal effects, but primary prevention trials for their effects on preventing fractures are lacking; 4) complementary and cost-effective use of the VITAL primary prevention trial makes it possible to test the effects of high doses of daily vitamin D on fractures 5) results of this proposed ancillary study will help guide clinical practice and public health guidelines related to correction of vitamin D insufficiency 6) these nutritional supplements may offer low-cost preventative interventions; and 7) if the proposed Aims are achieved, high dose daily

vitamin D and/or omega-3 fatty acids may reduce the burden of osteoporotic fractures, with the potential for substantial individual and public health benefits.

II. SPECIFIC AIMS

In the overall cohort (N=40,000), to test whether Vitamin D3 and/or EPA+DHA supplementation
a) will reduce incident total fractures according to annual questionnaires, medical record review, and fracture adjudication.
b) will reduce incident hip and non-vertebral fractures, when examined separately and according to annual questionnaires, medical record review, and fracture adjudication.

III. SUBJECT SELECTION

VITAL will be conducted among 40,000 apparently healthy participants. Included in the trial will be men aged ≥60 and women aged ≥65, ages at which rates of chronic disease increase substantially. All participants will need to have minimally a high school education (to complete mail-based questionnaires). Excluded from the study will be individuals who have (1) a history of cancer (except non-melanoma skin cancer), MI, stroke, TIA, angina, CABG, or PCI; (2) have any of the following safety exclusions: use of anticoagulants at baseline, history of kidney stones, renal failure or dialysis, hypercalcemia, hypo- or hyperparathyroidism, severe liver disease (cirrhosis), or sarcoidosis or other granulomatous diseases such as active chronic tuberculosis or Wegener's granulomatosis; (3) have an allergy to fish (for EPA+DHA); (4) have another serious illness that would preclude participation; (5) are consuming greater than 800 IU of vitamin D from all supplemental sources combined (individual vitamin D supplements, calcium+vitamin D supplements, medications with vitamin D [e.g., Fosamax Plus D], and multivitamins), or, and are not willing to decrease or forego such use during the trial (to ensure total supplemental vitamin D intake does not exceed the current safety limit [i.e., NOAEL] of 4000 IU/d set by the European Commission Scientific Committee on Food (EC SCF)); (6) are consuming greater than 1200 mg/d of calcium (the RDA for individuals aged >50[58]) from all supplemental sources combined; (7) are taking fish oil supplements and are not willing to forego their use during the trial; and (8) are not willing to participate, as evidenced by not signing the informed consent form. Although thiazide diuretics and supplemental vitamin D may interact to increase hypercalcemia risk, it is unlikely that such an interaction would occur at the vitamin D dose we propose.[59] Thus, thiazide use will not be an exclusion criterion.

These procedures will be conducted by the ***VITAL*** study staff.

IV. ENROLLMENT

These procedures will be conducted by the VITAL study staff.

V. STUDY PROCEDURES

Basic study procedures will be carried out by the VITAL study staff. The ancillary procedures are below.

All eligible subjects for the main study will receive annual questionnaires on fractures, and will be adjudicated on fractures as described below.

Does vitamin D3 and/or EPA+DHA supplementation reduce fractures?

Endpoint ascertainment and validation: The primary endpoint of the proposed ancillary study will be reduction of incident total, non-vertebral, and hip fractures. Fractures of head, shoulder/humerus/wrist/fingers, spine, rib/chest/sternum, pelvis, coccyx, leg, and ankle/foot/toes will be self-reported according to procedures used in the WHI.[70] Self-reported fractures will be confirmed with detailed medical record review for fracture validation and adjudication. Participants who self-report a fracture will be asked to sign a medical release form authorizing ***VITAL*** ancillary staff to obtain hospital/physician records. The request will be accompanied by a cover letter expressing concern for the diagnosis and explaining HIPAA guidelines and the scientific importance of validating study records. Non-responders will be sent 2 additional requests for the medical release form according to the parent RCT procedures. During the 10-year WHS trial, only 5% of cases refused to sign a medical release. After the release is obtained, a copy will be sent to the treating hospital/physician. In the WHS, provided that the release form was in order, the staff was able to obtain 99% of hospital/physician records. The Endpoints Committee of physicians, blinded to the randomized treatment assignment, will review the file and, using a defined protocol, will confirm or disconfirm the case. ***Medical Record Review:*** Medicare is a U.S. federal health insurance program that reimburses medical costs for most citizens and permanent residents aged >65 years, as well as some younger people with disabilities. On an annual basis, we will obtain Identifiable (Medicare) Data Files through ResDAC (www.resdac.unm.edu/Index.asp), a Centers for Medicare and Medicaid Services (CMS) contractor that provides free assistance to academic and non-profit researchers interested in using Medicare or Medicaid data (see Appendix B). For each hospitalization, we will obtain data on age, sex, race, admission date, discharge date, length of stay, date of death, principal and additional diagnoses using ICD-9-CM codes, and discharge status. Emergency room visits will be obtained from MedPAR, if there was a hospital admission; and from the Standard Analytical Files (SAF) if there was no admission. SAF records are based on inpatient and outpatient claims. Data from physician claims include a Common Procedure Terminology code for

each billed procedure that must be accompanied by an ICD-9-CM diagnosis code describing the reason for the service.[71, 72]

Questionnaires and Assessments: All participants will be sent yearly questionnaires on medical history, calcium and vitamin D intake (diet and supplements), sun exposure, physical activity, detailed nutritional intakes, lifestyle variables, falls, and risk factors for osteoporosis,.[73-83] Risk factor assessments for osteoporosis include questions about a fracture history in parent or other first-degree family members, and self history of fracture >age 50 (see Appendix A). Participants will be questioned whether they had history of certain medical conditions including premature menopause, diabetes, rheumatoid arthritis, hypo-hyperthyroidism, hypo/hyperparathyroidism, bowel diseases, celiac disease, inflammatory bowel disease, or gastrointestinal (including bariatric) surgery. The participants will also be asked about the current use of: oral diabetes medications, insulin, corticosteroids, diuretics, thyroid hormone, anti-convulsant medications, hormone therapy, bisphosphonates, proton pump inhibitors, selective-serotonin reuptake inhibitors, aromatase inhibitors, or anti-androgens and other medications that affect bone. ***Adjudication of Fractures:*** The adjudication process will include classification of all incident fracture events. The ancillary study investigators at BWH will collect fracture records, including x-ray reports and orthopedic notes, for each non-vertebral fracture reported during the trial. Fractures will be characterized by physician adjudication as traumatic if the circumstances included a fall from a more than a standing height.[70, 84, 85] The following are the fracture adjudication procedures: completion of the fracture event form for all non-vertebral fracture events reported during the proposed 4-year ancillary study; tracking of documents for case packets; and clarification of data queries. The ***VITAL*** ancillary study investigators will process all fracture reports according to the procedures documented in the manual of operations and provide validation and adjudication of fracture location and diagnosis.[66, 70, 84, 86] As reported in the WHIOS, the validity of self-reports is high, as agreement with medical records was 78% for hip and 81% for forearm/wrist fractures.[84]

Quality and Accuracy: BWH ancillary study investigators will be responsible for the quality and accuracy of the study database, data entry on the study forms, and identification and correction of inconsistent or missing data. Final review of the data will be performed by the Endpoints Committee.

VI. BIOSTATISTICAL ANALYSIS

<u>Data Analysis for Fracture Study</u>: Analyses of treatment effects will be based on the intent-to-treat principle. The first analysis will compare baseline characteristics by randomized treatment assignment to ensure that balance has been achieved by the randomization. This will be done in the total cohort. Risk factors for fractures to be assessed will include age; gender; race/ethnicity; parental history of a hip fracture; rheumatoid arthritis; BMI; smoking; alcohol use; the many medical conditions

associated with fragility fractures; and baseline vitamin D, omega-3 fatty acid, and calcium intakes.[23, 78, 79, 83, 87, 88] This fracture ancillary study will also assess baseline distributions of calcium intake, prior falls and fractures, physical activity level, physical function, use of a walking device, and use of medications such as glucocorticoids and antidepressants. The large sample size, as well as successful balance of known potential confounders, assures that unmeasured or unknown potential confounders will be equally distributed across randomized treatment groups, in the total cohort.

Analyses in the Total Cohort: In this 2x2 factorial design, the primary aim is to compare the main effects of intention-to-treat with vitamin D with fish oil on the study outcomes. For the time-to-event outcomes of fractures, falls, and the development of frailty, we will use the Cox proportional hazards model to allow for variable follow-up lengths,[89] and will estimate the hazard ratio for each intervention using indicators for treatment exposure, controlling for the second intervention, age, and gender. The primary analysis will estimate the cause-specific hazard and the hazard ratio comparing intervention groups for each outcome of interest by censoring individuals with deaths due to competing causes. We will also adjust for any imbalances by treatment group in baseline risk factors, particularly age, smoking, alcohol, BMI, physical activity, fall history, and calcium intake. Beyond the primary analyses of these outcomes, we will examine effect modification by the other randomized intervention, by baseline risk factors, and by time. We will explore interactions between the interventions and with the baseline dietary intake of vitamin D, calcium, and EPA+DHA in the entire cohort (N=20,000) and plasma levels of 25(OH)D, and EPA+DHA in 3,000 subjects. We hypothesize that the intervention effects will be larger among those with below-median baseline levels, and we will analyze treatment effects by quartiles of these nutrients. We will also evaluate effect modification by gender, race/skin pigmentation (for vitamin D), and BMI (for vitamin D). The latter interaction effects will be interpreted cautiously, as hypothesis generating. During the course of the trial, it is possible that an imbalance will emerge due to an effect of one of the agents on cancer or CVD disease, which may in turn lead to a difference in fractures or falls. Our primary analysis will remain an intent-to-treat analysis looking at any of these outcomes without regard to such intervening effects. In order to more thoroughly examine the causal relations and intervening mechanisms, we will also conduct a secondary analysis adjusting for the time-varying effects of such intervening events in a Cox model. We will further determine whether there is effect modification of the intervention effects among those who develop cancer

Table 1. Power for fracture in the total cohort.

Observed RR	True RR	Any Fracture	Non-Vertebral Fracture	Hip Fracture	Vertebral Fracture
0.90	0.875	73.7	71.6	17.4	12.7
0.85	0.812	97.6	97.0	34.2	23.3
0.80	0.750	>99.9	>99.9	55.5	38.2
0.75	0.687	>99.9	>99.9	75.8	55.7
0.70	0.625	>99.9	>99.9	89.9	72.6
0.65	0.560	>99.9	>99.9	96.9	85.8
0.60	0.500	>99.9	>99.9	99.4	94.0

or a CVD event using time-varying interaction terms. Alternatively, we will censor at the occurrence of these events, treating them as competing events and estimating the effect on fractures in their absence. These adjusted analyses will be regarded as exploratory.

Power for Ancillary Study: Power calculations for analysis of time-to-event outcomes in the entire cohort, namely fractures, falls, and frailty, are based on assumptions made for the parent grant, including a 2x2 factorial trial of 20,000 women and men. Power for bone loss outcomes assessed within the CTSC sub-cohort is based on measurements taken at baseline and at the year 2 follow-up among the 700 participants. For the CTSC sub-cohort we have conducted power calculations allowing for 15% and 20% drop out, or sample sizes of 595 and 560.

Fractures: Power for the primary aim of fractures is shown in Table 1. Rates are based on age-specific rates from the large population-based studies SOF (women) and MrOS (men).[78, 87, 90, 91] The cited reductions are the observed effects we would see. The corresponding 'true' RR is given assuming an average compliance of 80%. Power is shown for a two-sided test using a log-rank analysis[92] with a significance level of 0.05. Assuming that only one agent is effective, with 5 years of treatment and follow-up we will have 80% power to detect an observed RR of 0.89 (true RR = 86% if 80% compliance) for any fractures, RR of 0.88 (true RR=0.85) for clinical non-vertebral fractures, RR of 0.73 (true RR= 0.66) for hip fractures and RR of 0.67 (true RR = 0.59) for clinical vertebral fractures. These effect sizes, especially for any or non-vertebral fractures, are consistent with the 19-29% reductions seen in meta-analyses or observational studies of higher doses of vitamin D.[28] Power has been shown for a given observed reduction in risk, assuming an average compliance of 80%, similar to that achieved in our previous RCTs. Power for total fracture will be excellent with a 5-year follow-up even if compliance is lower. If the risk reduction is 15%, the observed RR corresponding to compliance of 80% is 0.88, and we have 88% power to detect this level of effect. If compliance is only 70%, the observed RR would be 0.89, with 81% power to detect an effect of this magnitude (Table 1).

VI. RISKS AND DISCOMFORTS

There is also the possibility of social/psychological risk from inadvertent disclosure of confidential information from the questionnaires or blood tests. However, we have many safeguards in place to avoid this possibility, and we have never had an inadvertent breach of confidentiality in any of our trials. The potential risk of disclosure of confidential information is guarded against by maintaining completed questionnaires in locked areas accessible by authorized personnel only. In questionnaire data files, participants are identified only by study ID. Subject identifiers are stored in separate

computer files with password protection, and results will be presented only in the aggregate. In addition, employees involved with the proposed study will be asked to sign a form agreeing not to disclose any information to which they might have access, regardless of their personal perception about its confidentiality. Each employee of the study who has access to study data or has contact with subjects participates in an institutional (Brigham and Women's Hospital) human subjects educational program, which consists of reviewing regulatory and informational documents pertaining to human subjects research; passing a test on ethical principles and regulations governing human subjects research; and signing a statement of commitment to the protection of human subjects. Finally, all such employees are required to participate in HIPAA training.

VII. POTENTIAL BENEFITS:

There are minimal direct benefits to the study participants. The study is intended to advance knowledge about possible benefits of vitamin D and fish oil on fractures, bone density, falls, and frailty. The data from the study will guide clinical practice and public health concerns about use of vitamin D and/or fish oil for bone health and fracture prevention.

IX. MONITORING AND QUALITY ASSURANCE

A. SAFETY MONITORING

An independent Data and Safety Monitoring Board (DSMB) will be assembled, consisting of NIH representatives and experts in clinical trials, epidemiology, biostatistics, orthopedics, endocrinology, and relevant clinical areas of bone and metabolic bone disease. The DSMB will make an annual examination of the progress of the trial and the un-blinded data on study endpoints and possible adverse effects to recommend continuation, alteration of study design, or early termination, as appropriate.

B. MONITORING OF SOURCE DATA AND DRUG DISPENSEMENT

Redundancies will be built into the data processing systems to insure accurate recording of data and proper follow-up. All research forms will be scanned in and the data read by a character-recognition software program (Teleform). Out-of-range, internally inconsistent, and unclear data will be reviewed by a verifier who will edit misread variables. Forms that cannot be scanned, and name and address changes, will be entered using traditional double-entry procedures. All data will undergo additional within-form and across-time checks to verify accuracy. Following data entry, all questionnaire responses that require additional follow-up for missing data, participant

comments on the form or for endpoint validation will be manually reviewed to insure correct processing and accurate follow-up. Address changes received from the post office or from participants will be manually keyed by data entry personnel and the resultant files compared and verified.

1. Adams, J.S., et al., *Vitamin D in defense of the human immune response.* Ann N Y Acad Sci, 2007. 1117: p. 94-105.
2. Chun, R.F., J.S. Adams, and M. Hewison, *Back to the future: a new look at 'old' vitamin D.* J Endocrinol, 2008. 198(2): p. 261-9.
3. Hewison, M., et al., *Extra-renal 25-hydroxyvitamin D3-1[alpha]-hydroxylase in human health and disease.* The Journal of Steroid Biochemistry and Molecular Biology, 2007. 103(3-5): p. 316-321.
4. Holick, M.F., *Vitamin D deficiency.* N Engl J Med, 2007. 357(3): p. 266-81.
5. Zhou, S., M.S. LeBoff, and J. Glowacki, *Vitamin D metabolism and action in human bone marrow stromal cells.* Endocrinology. 151(1): p. 14-22.
6. Holick, M.F. and T.C. Chen, *Vitamin D deficiency: a worldwide problem with health consequences.* Am J Clin Nutr, 2008. 87(4): p. 1080S-6S.
7. Heaney, R.P., *Vitamin D and calcium interactions: functional outcomes.* Am J Clin Nutr, 2008. 88(2): p. 541S-544S.
8. Giovannucci, E., *The epidemiology of vitamin D and cancer incidence and mortality: a review (United States).* Cancer Causes Control, 2005. 16(2): p. 83-95.
9. Zerwekh, J.E., *Blood biomarkers of vitamin D status.* Am J Clin Nutr, 2008. 87(4): p. 1087S-91S.
10. Dawson-Hughes, B., et al., *Estimates of optimal vitamin D status.* Osteoporos Int, 2005. 16(7): p. 713-6.
11. MacLaughlin, J. and M.F. Holick, *Aging decreases the capacity of human skin to produce vitamin D3.* J Clin Invest, 1985. 76(4): p. 1536-8.
12. Zadshir, A., et al., *The prevalence of hypovitaminosis D among US adults: data from the NHANES III.* Ethn Dis, 2005. 15(4 Suppl 5): p. S5-97-101.
13. Harris, S.S., *Vitamin D and African Americans.* J Nutr, 2006. 136(4): p. 1126-9.
14. Moore, C.E., M.M. Murphy, and M.F. Holick, *Vitamin D intakes by children and adults in the United States differ among ethnic groups.* J Nutr, 2005. 135(10): p. 2478-85.
15. Martins, D., et al., *Prevalence of cardiovascular risk factors and the serum levels of 25-hydroxyvitamin D in the United States: data from the Third National Health and Nutrition Examination Survey.* Arch Intern Med, 2007. 167(11): p. 1159-65.
16. Harris, S.S. and B. Dawson-Hughes, *Reduced sun exposure does not explain the inverse association of 25-hydroxyvitamin D with percent body fat in older adults.* J Clin Endocrinol Metab, 2007. 92(8): p. 3155-7.
17. Holick, M.F., et al., *Prevalence of Vitamin D inadequacy among postmenopausal North American women receiving osteoporosis therapy.* J Clin Endocrinol Metab, 2005. 90(6): p. 3215-24.
18. Holick, M.F., *MrOs is D-ficient.* J Clin Endocrinol Metab, 2009. 94(4): p. 1092-3.
19. Orwoll, E., et al., *Vitamin D deficiency in older men.* J Clin Endocrinol Metab, 2009. 94(4): p. 1214-22.
20. LeBoff, M.S., et al., *Vitamin D-deficiency and post-fracture changes in lower extremity function and falls in women with hip fractures.* Osteoporos Int, 2008. 19(9): p. 1283-90.

21. Pieper, C.F., et al., *Distribution and correlates of serum 25-hydroxyvitamin D levels in a sample of patients with hip fracture.* Am J Geriatr Pharmacother, 2007. 5(4): p. 335-40.
22. LeBoff, M.S., et al., *Occult vitamin D deficiency in postmenopausal US women with acute hip fracture.* Jama, 1999. 281(16): p. 1505-11.
23. Cauley, J.A., et al., *Serum 25-hydroxyvitamin D concentrations and risk for hip fractures.* Ann Intern Med, 2008. 149(4): p. 242-50.
24. Cranney, A., et al., *Summary of evidence-based review on vitamin D efficacy and safety in relation to bone health.* Am J Clin Nutr, 2008. 88(2): p. 513S-519S.
25. Cranney, A., et al., *Effectiveness and safety of vitamin D in relation to bone health.* Evid Rep Technol Assess (Full Rep), 2007(158): p. 1-235.
26. Feskanich, D., W.C. Willett, and G.A. Colditz, *Calcium, vitamin D, milk consumption, and hip fractures: a prospective study among postmenopausal women.* Am J Clin Nutr, 2003. 77(2): p. 504-11.
27. Bischoff-Ferrari, H.A., et al., *Fracture prevention with vitamin D supplementation: a meta-analysis of randomized controlled trials.* Jama, 2005. 293(18): p. 2257-64.
28. Bischoff-Ferrari, H.A., et al., *Prevention of nonvertebral fractures with oral vitamin D and dose dependency: a meta-analysis of randomized controlled trials.* Arch Intern Med, 2009. 169(6): p. 551-61.
29. Brannon, P.M., et al., *Overview of the conference "Vitamin D and Health in the 21st Century: an Update".* Am J Clin Nutr, 2008. 88(2): p. 483S-490S.
30. Boonen, S., J. Aerssens, and J. Dequeker, *Age-related endocrine deficiencies and fractures of the proximal femur. II implications of vitamin D deficiency in the elderly.* J Endocrinol, 1996. 149(1): p. 13-7.
31. Harma, M., et al., *Bone density, histomorphometry and biochemistry in patients with fractures of the hip or spine.* Ann Clin Res, 1987. 19(6): p. 378-82.
32. LeBoff M.S., et al., *Compared with osteoporotic controls acute hip fracture patients show high PTH and low vitamin D levels.*, in *79th Annual Meeting of The Endocrine Society*. 1997.
33. Simon, J., et al., *Fractures in the elderly and vitamin D.* J Nutr Health Aging, 2002. 6(6): p. 406-12.
34. Glowacki, J. and L. M.S., *Vitamin D insufficiency and fracture risk.* Current Opinion in Endocrinology and Diabetes, 2004. 11(6): p. 353-358.
35. Glowacki, J., et al., *Importance of vitamin D in hospital-based fracture care pathways.* J Nutr Health Aging, 2008. 12(5): p. 291-3.
36. Glowacki, J., et al., *Osteoporosis and vitamin-D deficiency among postmenopausal women with osteoarthritis undergoing total hip arthroplasty.* J Bone Joint Surg Am, 2003. 85-A(12): p. 2371-7.
37. Lewiecki, E.M., et al., *2008 santa fe bone symposium: update on osteoporosis.* J Clin Densitom, 2009. 12(2): p. 135-57.
38. Harris, M.B., et al., *Evolution of comprehensive hospital care pathways to advance the treatment of fragility fractures.* The Orthopaedic Journal at Harvard Medical School, 2007(9): p. 95-97.
39. Simopoulos, A.P., *Evolutionary aspects of omega-3 fatty acids in the food supply.* Prostaglandins Leukot Essent Fatty Acids, 1999. 60(5-6): p. 421-9.
40. Sun, D., et al., *Dietary n-3 fatty acids decrease osteoclastogenesis and loss of bone mass in ovariectomized mice.* J Bone Miner Res, 2003. 18(7): p. 1206-16.
41. Matsushita, H., et al., *Dietary fish oil results in a greater bone mass and bone formation indices in aged ovariectomized rats.* J Bone Miner Metab, 2008. 26(3): p. 241-7.

42. Sakaguchi, K., I. Morita, and S. Murota, *Eicosapentaenoic acid inhibits bone loss due to ovariectomy in rats.* Prostaglandins Leukot Essent Fatty Acids, 1994. 50(2): p. 81-4.
43. Yamada, Y., et al., *Effect of eicosapentaenoic acid and docosahexaenoic acid on diabetic osteopenia.* Diabetes Res Clin Pract, 1995. 30(1): p. 37-42.
44. Reinwald, S., et al., *Repletion with (n-3) fatty acids reverses bone structural deficits in (n-3)-deficient rats.* J Nutr, 2004. 134(2): p. 388-94.
45. Watkins, B.A., et al., *Omega-3 polyunsaturated fatty acids and skeletal health.* Exp Biol Med (Maywood), 2001. 226(6): p. 485-97.
46. Poulsen, R.C. and M.C. Kruger, *Detrimental effect of eicosapentaenoic acid supplementation on bone following ovariectomy in rats.* Prostaglandins Leukot Essent Fatty Acids, 2006. 75(6): p. 419-27.
47. Poulsen, R.C., et al., *Specific effects of gamma-linolenic, eicosapentaenoic, and docosahexaenoic ethyl esters on bone post-ovariectomy in rats.* Calcif Tissue Int, 2007. 81(6): p. 459-71.
48. Bonnet, N. and S. Ferrari, *A long-term diet enriched in omega-3 fatty acids improves cortical bone structure and mechanical properties in mice. Abstract#M47*, in *New Frontiers in Skeletal Research: Bone, Fat, and Brain Connections*. April 27-28, 2009: Bethesda, MD.
49. Salari, P., et al., *A systematic review of the impact of n-3 fatty acids in bone health and osteoporosis.* Med Sci Monit, 2008. 14(3): p. RA37-44.
50. Harris, W.S., *n-3 fatty acids and serum lipoproteins: animal studies.* Am J Clin Nutr, 1997. 65(5 Suppl): p. 1611S-1616S.
51. Harris, W.S., *n-3 fatty acids and serum lipoproteins: human studies.* Am J Clin Nutr, 1997. 65(5 Suppl): p. 1645S-1654S.
52. Van Papendrop, D.H., H. Coetzer, and M.G. Kruger, *Biochemical profile of osteoporotic patients on essential fatty acid supplementation.* Nutr Res, 1995(15): p. 325-34.
53. Kruger, M.C., et al., *Calcium, gamma-linolenic acid and eicosapentaenoic acid supplementation in senile osteoporosis.* Aging (Milano), 1998. 10(5): p. 385-94.
54. Bassey, E.J., et al., *Lack of effect of supplementation with essential fatty acids on bone mineral density in healthy pre- and postmenopausal women: two randomized controlled trials of Efacal v. calcium alone.* Br J Nutr, 2000. 83(6): p. 629-35.
55. Macdonald, H.M., et al., *Nutritional associations with bone loss during the menopausal transition: evidence of a beneficial effect of calcium, alcohol, and fruit and vegetable nutrients and of a detrimental effect of fatty acids.* Am J Clin Nutr, 2004. 79(1): p. 155-65.
56. Griel, A.E., et al., *An increase in dietary n-3 fatty acids decreases a marker of bone resorption in humans.* Nutr J, 2007. 6: p. 2.
57. Rousseau, J.H., A. Kleppinger, and A.M. Kenny, *Self-reported dietary intake of omega-3 fatty acids and association with bone and lower extremity function.* J Am Geriatr Soc, 2009. 57(10): p. 1781-8.
58. Institute of Medicine Food and Nutrition Board, *Dietary Reference Intakes: Calcium, Phosphorous, Magnesium, Vitamin D and Fluoride*. 1999, Washington DC: National Academies Press.
59. Hathcock, J.N., et al., *Risk assessment for vitamin D.* Am J Clin Nutr, 2007. 85(1): p. 6-18.
60. Lang, J.M., et al., *Estimating the effect of the run-in on the power of the Physicians' Health Study.* Stat Med, 1991. 10(10): p. 1585-93.

61. Fitzpatrick, T.B., *The validity and practicality of sun-reactive skin types I through VI.* Arch Dermatol, 1988. 124(6): p. 869-71.
62. Binkley, N., et al., *Correlation among 25-hydroxy-vitamin D assays.* J Clin Endocrinol Metab, 2008. 93(5): p. 1804-8.
63. Binkley, N., et al., *Assay variation confounds the diagnosis of hypovitaminosis D: a call for standardization.* J Clin Endocrinol Metab, 2004. 89(7): p. 3152-7.
64. Bischoff-Ferrari, H.A., et al., *Estimation of optimal serum concentrations of 25-hydroxyvitamin D for multiple health outcomes.* Am J Clin Nutr, 2006. 84(1): p. 18-28.
65. Aloia, J.F., et al., *Vitamin D intake to attain a desired serum 25-hydroxyvitamin D concentration.* Am J Clin Nutr, 2008. 87(6): p. 1952-8.
66. Jackson, R.D., et al., *Calcium plus vitamin D supplementation and the risk of fractures.* N Engl J Med, 2006. 354(7): p. 669-83.
67. Bolland, M.J., et al., *Vascular events in healthy older women receiving calcium supplementation: randomised controlled trial.* BMJ, 2008. 336(7638): p. 262-6.
68. Radimer, K., et al., *Dietary supplement use by US adults: data from the National Health and Nutrition Examination Survey, 1999-2000.* Am J Epidemiol, 2004. 160(4): p. 339-49.
69. Satterfield, S., et al., *Biochemical markers of compliance in the Physicians' Health Study.* Am J Prev Med, 1990. 6(5): p. 290-4.
70. *Design of the Women's Health Initiative clinical trial and observational study. The Women's Health Initiative Study Group.* Control Clin Trials, 1998. 19(1): p. 61-109.
71. Klabunde, C.N., et al., *Development of a comorbidity index using physician claims data.* J Clin Epidemiol, 2000. 53(12): p. 1258-67.
72. Fisher, E.S., et al., *The accuracy of Medicare's hospital claims data: progress has been made, but problems remain.* Am J Public Health, 1992. 82(2): p. 243-8.
73. Woods, N.F., et al., *Frailty: emergence and consequences in women aged 65 and older in the Women's Health Initiative Observational Study.* J Am Geriatr Soc, 2005. 53(8): p. 1321-30.
74. Pereira, M.A., et al., *A collection of Physical Activity Questionnaires for health-related research.* Med Sci Sports Exerc, 1997. 29(6 Suppl): p. S1-205.
75. Robbins, J., et al., *Body mass index is not a good predictor of bone density: results from WHI, CHS, and EPIDOS.* J Clin Densitom, 2006. 9(3): p. 329-34.
76. Ensrud, K.E., et al., *Comparison of 2 frailty indexes for prediction of falls, disability, fractures, and death in older women.* Arch Intern Med, 2008. 168(4): p. 382-9.
77. Ensrud, K.E., et al., *A comparison of frailty indexes for the prediction of falls, disability, fractures, and mortality in older men.* J Am Geriatr Soc, 2009. 57(3): p. 492-8.
78. Cummings, S.R., et al., *Risk factors for hip fracture in white women. Study of Osteoporotic Fractures Research Group.* N Engl J Med, 1995. 332(12): p. 767-73.
79. Watts, N.B., B. Ettinger, and M.S. LeBoff, *FRAX facts.* J Bone Miner Res 2009. 24(6): p. 975-979.
80. Israel, E., et al., *Effects of inhaled glucocorticoids on bone density in premenopausal women.* N Engl J Med, 2001. 345(13): p. 941-7.
81. LeBoff M.S. Guest Editor, et al., *The Spectrum of Renal Bone Disease.* Seminars in Nephrology, 2009.
82. Bernstein, C.N., W.D. Leslie, and M.S. Leboff, *AGA technical review on osteoporosis in gastrointestinal diseases.* Gastroenterology, 2003. 124(3): p. 795-841.

83. Leboff, M.S., et al., *Homocysteine levels and risk of hip fracture in postmenopausal women.* J Clin Endocrinol Metab, 2009. 94(4): p. 1207-13.
84. Chen, Z., et al., *Validity of self-report for fractures among a multiethnic cohort of postmenopausal women: results from the Women's Health Initiative observational study and clinical trials.* Menopause, 2004. 11(3): p. 264-74.
85. Chen, Z., et al., *Fracture risk among breast cancer survivors: results from the Women's Health Initiative Observational Study.* Arch Intern Med, 2005. 165(5): p. 552-8.
86. Cauley, J.A., et al., *Effects of estrogen plus progestin on risk of fracture and bone mineral density: the Women's Health Initiative randomized trial.* Jama, 2003. 290(13): p. 1729-38.
87. Orwoll, E., et al., *Design and baseline characteristics of the osteoporotic fractures in men (MrOS) study--a large observational study of the determinants of fracture in older men.* Contemp Clin Trials, 2005. 26(5): p. 569-85.
88. Robbins, J., et al., *Factors associated with 5-year risk of hip fracture in postmenopausal women.* Jama, 2007. 298(20): p. 2389-98.
89. Cox, D.R., *Regression models and life-tables.* J Royal Statist Soc B, 1972. 34(2): p. 187-220.
90. Cummings, S.R. and L.J. Melton, *Epidemiology and outcomes of osteoporotic fractures.* Lancet, 2002. 359(9319): p. 1761-7.
91. Cawthon, P.M., *Personal Communication*, L. M.S., Editor. 2009.
92. Freedman, L.S., *Tables of the number of patients required in clinical trials using the logrank test.* Stat Med, 1982. 1(2): p. 121-9.
93. Haybittle, J.L., *Repeated assessment of results in clinical trials of cancer treatment.* Br J Radiol, 1971. 44(526): p. 793-7.
94. Peto, R., et al., *Design and analysis of randomized clinical trials requiring prolonged observation of each patient. I. Introduction and design.* Br J Cancer, 1976. 34(6): p. 585-612.
95. Geller, N.L. and S.J. Pocock, *Interim analyses in randomized clinical trials: ramifications and guidelines for practitioners.* Biometrics, 1987. 43(1): p. 213-23.
96. O'Brien, P.C. and T.R. Fleming, *A multiple testing procedure for clinical trials.* Biometrics, 1979. 35(3): p. 549-56.
97. DeMets, D.L. and K.K. Lan, *Interim analysis: the alpha spending function approach.* Stat Med, 1994. 13(13-14): p. 1341-52; discussion 1353-6.
98. Pocock, S.J., *Current controversies in data monitoring for clinical trials.* Clin Trials, 2006. 3(6): p. 513-21.

Supplementary Online Content

Orkaby AR, Dushkes R, Ward R, et al. Effect of vitamin D_3 and omega-3 fatty acid supplementation on risk of frailty: an ancillary study of a randomized clinical trial. *JAMA Netw Open*. 2022;5(9):e2231206. doi:10.1001/jamanetworkopen.2022.31206

eTable 1. Cumulative Deficit Frailty Index Components for the VITAL Trial

eTable 2. Fried Physical Phenotype of Frailty in VITAL Subcohort (n=1054)

eTable 3. Baseline Characteristics of the VITAL Trial (n=25 057) According to Frailty Status

eTable 4. Adjusted Means at Baseline and Mean Change (95% CI) in Frailty Level at Each Year Since Randomization Compared to Baseline, According to Vitamin D vs Placebo and Omega-3 vs Placebo Groups

eTable 5. Baseline Characteristics of 1054 Participants in the VITAL Trial in Person Subgroup

eTable 6A. Adjusted Means at Baseline and Mean Change (95% CI) in Fried Frailty Level at Each Year Since Randomization Compared to Baseline, According to Vitamin D and Placebo Groups

eTable 6B. Adjusted Means at Baseline and Mean Change (95% CI) in Fried Frailty Level at Each Year Since Randomization Compared to Baseline, According to n-3 and Placebo Groups

eFigure 1. Distribution of Frailty Index at Baseline

eFigure 2. Distribution of Frailty Index at Baseline According to Randomization Group

eFigure 3. Validation of the FI: Association Between FI Level and Mortality

eFigure 4A. Effect of Vitamin D_3 on Incident Frailty Over Time

eFigure 4B. Effect of Omega-3 on Incident Frailty Over Time

eFigure 5. Vitamin D_3 Supplementation and Frailty Subgroups

eFigure 6. Omega-3 Supplementation and Frailty Subgroups

eFigure 7. Effect of Vitamin D_3 on Incident Fried Frailty Over Time

eFigure 8. Effect of Omega-3 on Incident Fried Frailty Over Time

eReferences

This supplementary material has been provided by the authors to give readers additional information about their work.

© 2022 Orkaby AR et al. *JAMA Network Open.*

eTable 1. Cumulative Deficit Frailty Index components for the VITAL trial

Author note: According to the accumulated deficit theory of frailty, deficits included in the frailty index are not weighted. Rather within an individual, the number of deficits present are counted and divided by the total variables determined at the outset (in this case, 36).[1,2]
The upper limit of the proportion of deficits that can be positive and still be compatible life is about 2/3, or a frailty index score or 0.7.

Count	Variable	Coding for the Frailty Index
	Health Status and Function	
	Does your health now limit you in these activities, and, if so, how much?	
1	Moderate activities such as moving a table, vacuuming, bowling, or golf	A lot =1, a little = 0.5, No = 0
2	Lifting or carrying groceries	A lot =1, a little = 0.5, No = 0
3	Climbing several flights of stairs	A lot =1, a little = 0.5, No = 0
4	Climbing one flight of stairs	A lot =1, a little = 0.5, No = 0
5	Bending, kneeling, stooping	A lot =1, a little = 0.5, No = 0
6	Walking more than a mile	A lot =1, a little = 0.5, No = 0
7	Walking several blocks	A lot =1, a little = 0.5, No = 0
8	Walking one block	A lot =1, a little = 0.5, No = 0
9	Bathing or dressing yourself	A lot =1, a little = 0.5, No = 0
	Activities of Daily Living	
10	Can you feed yourself	Without help =0, some help = 0.5, Unable = 1
11	Can you dress and undress yourself	Without help =0, some help = 0.5, Unable = 1
12	Can you get in and out of bed by yourself	Without help =0, some help = 0.5, Unable = 1
13	Can you take a bath or shower	Without help =0, some help = 0.5, Unable = 1
	Mood	
	Over the past 2 weeks, how often have you been bothered by any of the following:	
14	Little interest or pleasure in doing things	Not at all = 0, several days = 0.33, More than half the days = 0.67, nearly every day = 1
15	Feeling down, depressed or hopeless	Not at all = 0, several days = 0.33, More than half the days = 0.67, nearly every day = 1
16	Trouble falling or staying asleep, or sleeping too much	Not at all = 0, several days = 0.33, More than half the days = 0.67, nearly every day = 1
17	Feeling tired or having little energy	Not at all = 0, several days = 0.33, More than half the days = 0.67, nearly every day = 1
18	Poor appetite or overeating	Not at all = 0, several days = 0.33, More than half the days = 0.67, nearly every day = 1
19	Feeling bad about yourself or that you are a failure or have let yourself or your family down	Not at all = 0, several days = 0.33, More than half the days = 0.67, nearly every day = 1
20	Trouble concentrating on things like reading the paper or watching T.V.	Not at all = 0, several days = 0.33, More than half the days = 0.67, nearly every day = 1
21	Moving or speaking so slowly that others could have noticed? Or the opposite, being fidgety, restless, or moving a lot more than usual	Not at all = 0, several days = 0.33, More than half the days = 0.67, nearly every day = 1

© 2022 Orkaby AR et al. *JAMA Network Open.*

22	Diagnosis of depression	Yes =1, No =0
	Comorbidities	
23	Anemia	Yes = 1, No =0
24	Atrial Fibrillation	Yes=1, No =0
25	Hypertension	Yes=1, No =0
26	Lung comorbidity: asthma, chronic bronchitis, emphysema, or COPD	If answered Yes to any = 1, If all No then =0
27	Liver disease	Yes=1, No =0
28	Kidney Disease	Yes=1, No =0
29	Diabetes	Yes=1, No =0
30	Heart Failure	Yes = 1, No =0
31	Arthritis comorbidity: osteoarthritis, polymyalgia rheumatica, rheumatoid disease, psoriatic arthritis	If any are Yes = 1<br>If all No then =0
32	Parkinson's disease	Yes = 1, No =0
33	Macular degeneration	Yes = 1, No =0
34	Periodontal disease	Yes = 1, No =0
	Measured variables	
35	BMI	<18.5 or >30=1,<br>25-<30 = 0.5<br>18.5-<25 = 0
36	Weight loss	Unintentional weight loss of ≥5 lbs in the last year =1, otherwise =0

© 2022 Orkaby AR et al. *JAMA Network Open.*

eTable 2. Fried physical phenotype of frailty in VITAL subcohort (n=1054)

Variable	Men	Women
Shrinking: Weight loss	Unintentional weight loss ≥5 lb in the last 2 years	
Exhaustion	Answered “More than half the days” or “nearly every day” to the question “Feeling tired or having little energy”	
Low physical activity	kcal of energy expended in a week in the lowest 20% for men	kcal of energy expended in a week in the lowest 20% for women
Slow: walking speed m/s	Height ≤173 cm: ≤0.96<br>Height >173 cm: ≤1.04	Height ≤173 cm: ≤0.90<br>Height >173 cm: ≤1.02
Weakness: Grip strength (kg)	BMI ≤ 24.0: ≤ 29<br>BMI 24.1-26.0: ≤ 30<br>BMI 26.1-28.0: ≤ 30<br>BMI > 28.0: ≤ 32	BMI ≤ 23.0: ≤ 17<br>BMI 23.1 to 26.0: ≤ 17.3<br>BMI 26.1 to 29.0: ≤ 18<br>BMI > 29.0: ≤ 21

Calculating frailty: Participants with ≥3 criteria are considered frail, those with 1-2 criteria are pre-frail while those with none are robust.

© 2022 Orkaby AR et al. *JAMA Network Open.*

eTable 3. Baseline Characteristics of the VITAL trial (n=25,057) According to Frailty Status

	**Non-frail (n=14598)**	**Pre-frail (n=7285)**	**Frail (n=3174)**
**Age (mean, SD)**	66.8 (6.7)	67.8 (7.3)	67.2 (7.8)
**Female sex %**	43.4	57.7	68.1
**Race or ethnic group**[a]			
**Asian/Pacific Islander**	1.7	1.2	1.0
**Black or African American**	14.3	23.0	37.5
**Native American/Alaskan Native**	0.8	1.0	1.4
**Non-Black Hispanic**	3.8	3.9	4.6
**Non-Hispanic white**	77.7	68.8	52.7
**Other or unknown**	1.8	2.2	2.9
**Body Mass Index(kg/m^2)**[b]	26.3 (4.2)	29.5 (5.7)	33.1 (7.8)
**Current smoker %**	5.1	8.1	14.3
**Daily alcohol use %**	30.8	22.7	13.2
**Baseline aspirin use %**	42.9	49.5	48.0
**Baseline statin use %**	30.7	40.3	43.0
**Baseline dietary fish consumption ≥1.5 servings/wk %**	47.4	46.1	46.0
**Baseline 25(OH)D levels (ng/ml) (mean, SD)**	31.55 (9.7)	30.5 (10.1)	28.2 (10.8)
**Leisure-time physical activity and stair climbing, total MET-hours/week, mean (SD)**	27.2 (27.18)	18.7 (22.5)	10.9 (19.5)
**Randomized to omega-3 %**	50.1	49.6	50.4
**Randomized to vitamin D_3 %**	49.7	49.9	50.6

[a] Other race or ethnicity categories include: patients who indicated other or unknown race or ethnicity
[b] Body mass index is weight in kilograms divided by the square of height in meters.
[c] Alcohol use >= 1 drink per day wine (5oz glass), liquor (shot) or beer (bottle, can or glass)
[d] At least monthly

© 2022 Orkaby AR et al. *JAMA Network Open.*

eTable 4. Adjusted Means at Baseline and Mean Change (95% CI) in Frailty level at Each Year Since Randomization Compared to Baseline, according to Vitamin D vs placebo and Omega-3 vs Placebo Groups[a]

	No. participants	Mean change (SE)[b]	No. participants	Mean change (SE)[b]	Mean difference in change (95% CI)[c]	P-value	P-interaction[d]
	**Vitamin D group**		**Placebo group**				
Baseline	12497	0.1097 (0.0008)	12560	0.1092 (0.0008)	--	--	
Year 3 vs Baseline	11356	0.0069 (0.00056)	11405	0.0068 (0.00056)	0.00016 (-0.0014, 0.0017)	0.84	0.98
Year 4 vs Baseline	10760	0.0117 (0.00061)	10689	0.0118 (0.00062)	-0.00004 (-0.0018, 0.0017)	0.96	
Year 5 vs Baseline	7154	0.0177 (0.00072)	7133	0.0179 (0.00071)	-0.0002 (-0.0022, 0.0018)	0.85	
**Average (across Years 3-5) vs Baseline[e]**	**12497**		**12560**		**0.00005 (-0.0014, 0.0015)**	**0.94**	
	**Omega-3 group**		**Placebo group**				
Baseline	12529	0.1092 (0.0008)	12528	0.1097 (0.0008)	--	--	
Year 3 vs Baseline	11376	0.0064 (0.00056)	11385	0.0073 (0.00056)	-0.00087 (-0.00243, 0.00069)	0.27	0.13
Year 4 vs Baseline	10741	0.0109 (0.00061)	10708	0.0126 (0.00062)	-0.00167 (-0.00338, 0.00003)	0.05	
Year 5 vs Baseline	7179	0.0178 (0.00072)	7108	0.0179 (0.00071)	-0.00013 (-0.00211, 0.00185)	0.90	
**Average (across Years 3-5) vs Baseline[e]**	**12529**		**12528**		**-0.00099 (-0.00245, 0.00047)**	**0.18**	

© 2022 Orkaby AR et al. *JAMA Network Open.*

Legend: Abbreviation: CI, confidence interval: Rockwood frailty level; SE, standard error.

P-values are provided for the mean difference in frailty level change over time.

[a]Analyses are presented from general linear models of response profiles to estimate the means, with time modeled as indicator variables, and models were controlled for randomization group (vitamin D_3 or omega-3). Adjusted means (95% CI) within each treatment group are shown at baseline and adjusted mean differences in change (95% CI) within each treatment group are shown for each follow-up time point. The repeated measures model used the available responses. All participants contributed data to the repeated measures analysis at one or more times.

[b]The first row shows mean (SE) of the FI at baseline, subsequent rows show the mean change (SE)

[c]Net difference in change comparing active and placebo group from the repeated measures model.

[d]P-interaction is from the test of the interaction term treatment-x-time in the model

[e]The final raw shows the net difference between the treatment groups in frailty level averaged across follow-up years (3-5)

© 2022 Orkaby AR et al. *JAMA Network Open.*

**eTable 5** Baseline Characteristics of 1054 participants in the VITAL trial – in person subgroup				
	**Vitamin D_3 and Omega-3s N=261**	**Vitamin D_3 only N=259**	**Omega-3 only N=266**	**Placebo only N=268**
**Age (mean, SD)**	64.7 (6.5)	64.6 (6.2)	65.0 (6.5)	65.2 (6.7)
**Female sex %**	49.4	49.0	49.3	47.8
**Race or ethnic group %** [a]				
**Asian/Pacific Islander**	2.4	2.0	1.9	0.8
**Black or African American**	8.6	7.8	8.1	9.5
**Native American/Alaskan Native**	0.4	0.4	1.5	0
**Non Black Hispanic**	3.5	1.2	2.7	3.8
**Non-Hispanic white**	82.8	85.2	84.2	85.5
**Other or unknown**	2.4	3.5	1.5	0.4
**Body Mass Index(kg/m^2)** [b]	28.4 (5.3)	27.8 (5.3)	28.9 (5.4)	27.7 (4.9)
**Current smoker %**	5.8	5.9	4.1	6.0
**Daily alcohol use %**[c]	30.7	31.3	32.9	29.7
**Current regular aspirin use %**[d]	47.7	41.7	46.8	38.7
**Current use of statin %**	36.9	31.4	32.8	32.2
**Baseline 25(OH)D levels (ng/ml) (mean, SD)**	27.7 (8.7)	27.5 (8.9)	28.9 (10.2)	28.4 (8.4)
**Baseline dietary fish consumption ≥1.5 servings/wk %**	60.1	53.3	57.3	57.4
**Leisure-time physical activity and stair climbing, total MET-hours/week, mean (SD)**	30.9 (31.9)	24.1 (21.2)	26.1 (25.3)	27.4 (25.4)
**Fried frailty score, Median (IQR)**	0.0 (0.0-1.0)	0.0 (0.0-1.0)	0.0 (0.0-1.0)	0.0 (0.0-1.0)

There were no significant differences between groups with regards to baseline characteristics.
[a] Race and ethnic group were reported by participants. Other race or ethnicity categories include: patients who indicated other or unknown race or ethnicity.
[b] Body mass index is weight in kilograms divided by the square of height in meters.
[c] Alcohol use >= 1 drink per day wine (5oz glass), liquor (shot) or beer (bottle, can or glass)
[d] At least monthly.

© 2022 Orkaby AR et al. *JAMA Network Open.*

eTable 6A. Adjusted Means at Baseline and Mean Change (95% CI) in Fried frailty level at Each Year Since Randomization Compared to Baseline, according to Vitamin D and Placebo Groups*

	**Vitamin D**		**Placebo group**		**Mean difference in change (95% CI)** [b]	**P-value**	**P-interaction** [a]
	No. of participants	Mean (SE)	No. of participants	Mean (SE)			
Baseline	520	0.5558 (0.0345)	534	0.5262 (0.0344)			**0.59**
Year 2 vs Baseline	472	0.1697 (0.0334)	495	0.1696 (0.0321)	0.00005 (-0.0908-0.0909)	0.999	
Year 4 vs Baseline	350	0.1711 (0.0445)	338	0.2339 (0.0445)	-0.063(-0.1863-0.0608)	0.32	
**Average (across Years 2, 4) vs Baseline**	**520**		**534**		**-0.1922 (-0.1001-0.0616)**	**0.64**	

© 2022 Orkaby AR et al. *JAMA Network Open.*

eTable 6B. Adjusted Means at Baseline and Mean Change (95% CI) in Fried frailty score at Each Year Since Randomization Compared to Baseline, according to n-3 and Placebo Groups*

	**Omega-3 group**		**Placebo group**		**Mean difference in change (95% CI)** [b]	**P-value**	**P-interaction**[a]
	No. of participants	Mean (SE)	No. of participants	Mean (SE)			**0.53**
Baseline	527	0.5408 (0.0348	527	0.5408 (0.0341)			
Year 2 vs Baseline	481	0.1706(0.0331)	486	0.1685 (0.0324)	0.0021 (-0.0887-0.0929)	0.96	
Year 4 vs Baseline	344	0.1681(0.0456)	344	0.2360 (0.0435)	-0.0679 (-0.1914-0.0557)	0.28	
**Average (across Years 2, 4) vs Baseline ****	**527**		**527**		**-0.0194 (-0.1002-0.0615)**	**0.64**	

Abbreviation: CI, confidence interval: Fried frailty.

*** Analyses are presented from general linear models of response profiles to estimate the means, with time modeled as indicator variables; models were controlled for randomization group (vitamin D , Omega-3). Adjusted means (95% CI) within each treatment group are shown at baseline and adjusted mean differences in change (95% CI) are shown within each treatment group for each follow-up time point. All participants contributed data to the repeated measures analysis one or more times.**

[a]P-interaction is from the test of the interaction term treatment-x-time.

[b]Net mean difference in change comparing active and placebo group from the repeated measures model.

[**]The final raw shows the net difference between the treatment groups in frailty level averaged across follow-up years (2,4) .

P-value is from Type 3 Tests of Fixed Effects treatment-x-time (years 2 and 4)) in the repeated measures model.

© 2022 Orkaby AR et al. *JAMA Network Open.*

eFigure 1. Distribution of Frailty Index at baseline

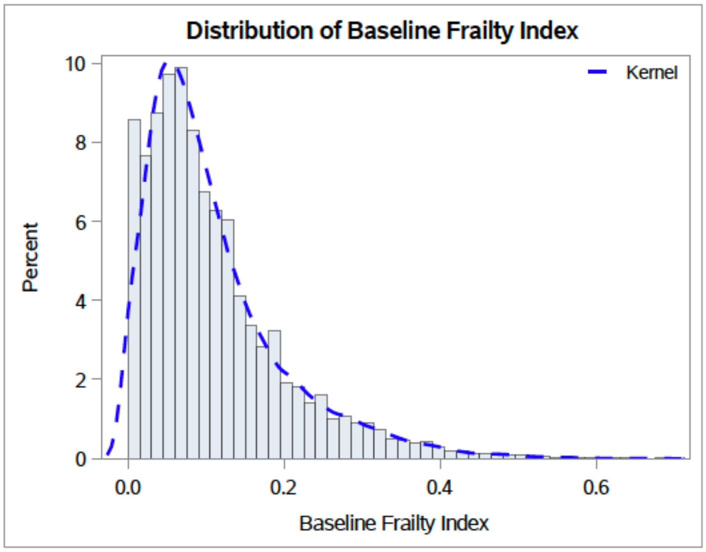

© 2022 Orkaby AR et al. *JAMA Network Open.*

eFigure 2. Distribution of Frailty Index at baseline according to randomization group

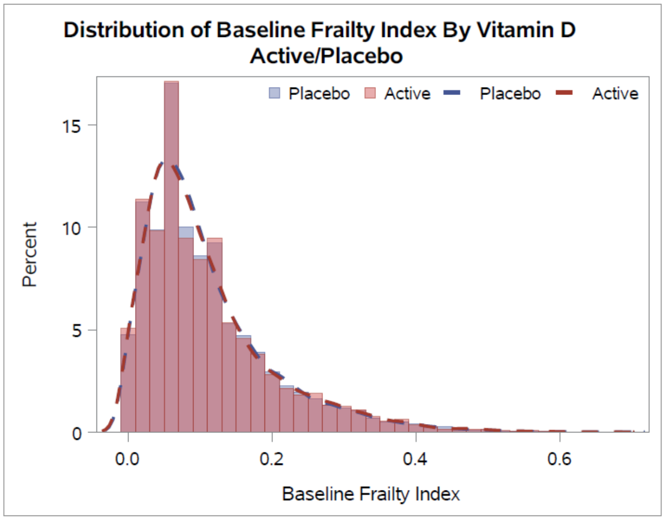

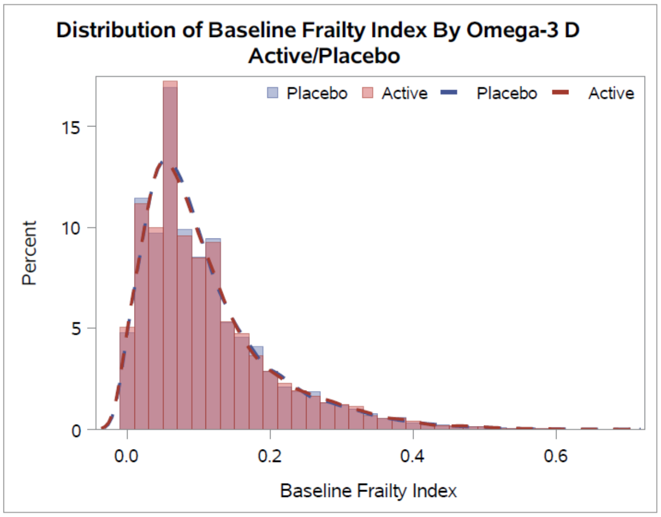

© 2022 Orkaby AR et al. *JAMA Network Open.*

eFigure 3. Validation of the FI: Association between FI level and mortality

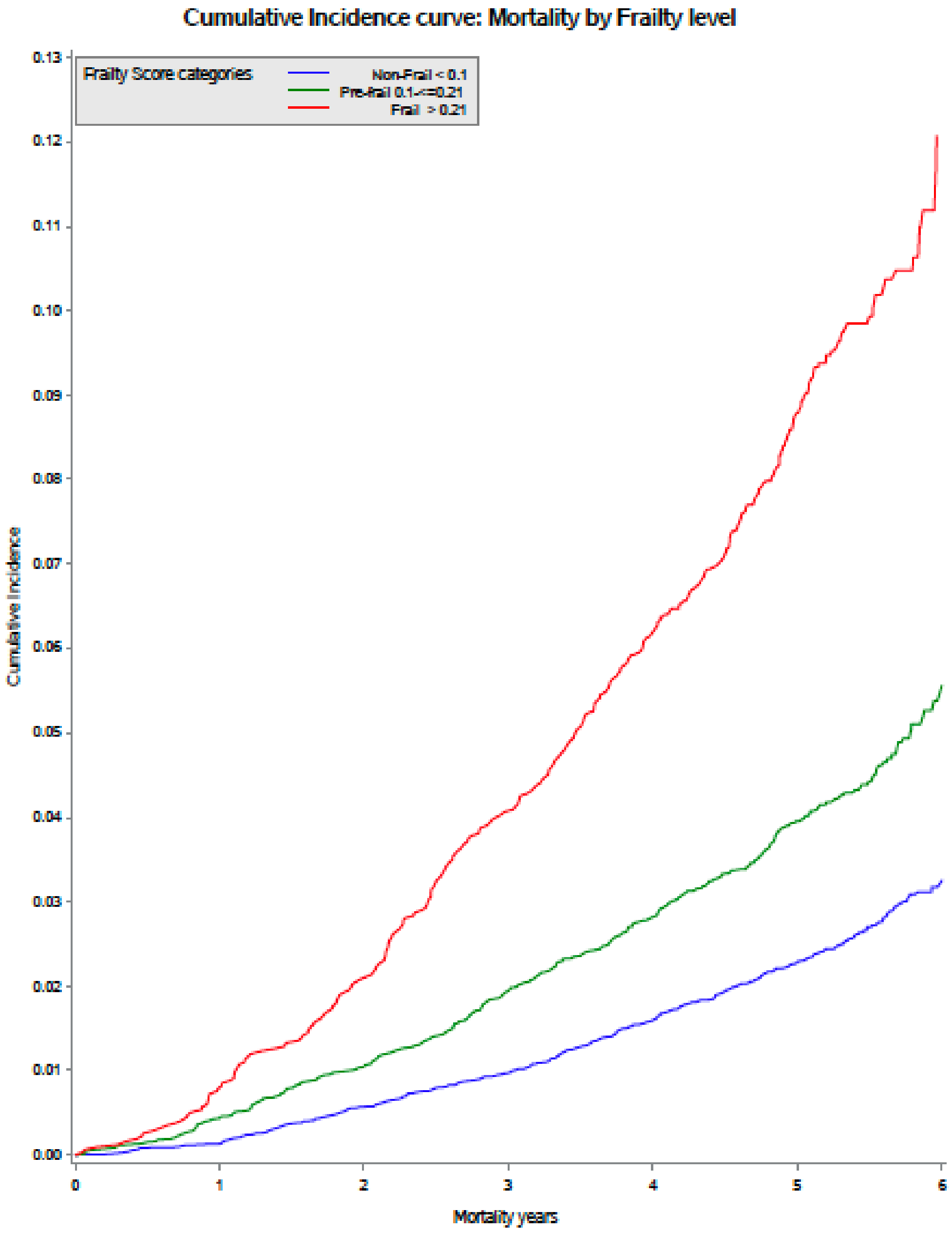

Frailty level	Hazard Ratio for mortality	95% CI	p-value
Robust (<0.1)	Ref	--	--
Pre-frail (0.1-0.2)	1.67	1.44-1.95	<0.001
Frail (>0.21)	4.10	3.51-4.78	<0.001

© 2022 Orkaby AR et al. *JAMA Network Open.*

e**Figure 4A.** Effect of vitamin D_3 on incident frailty over time

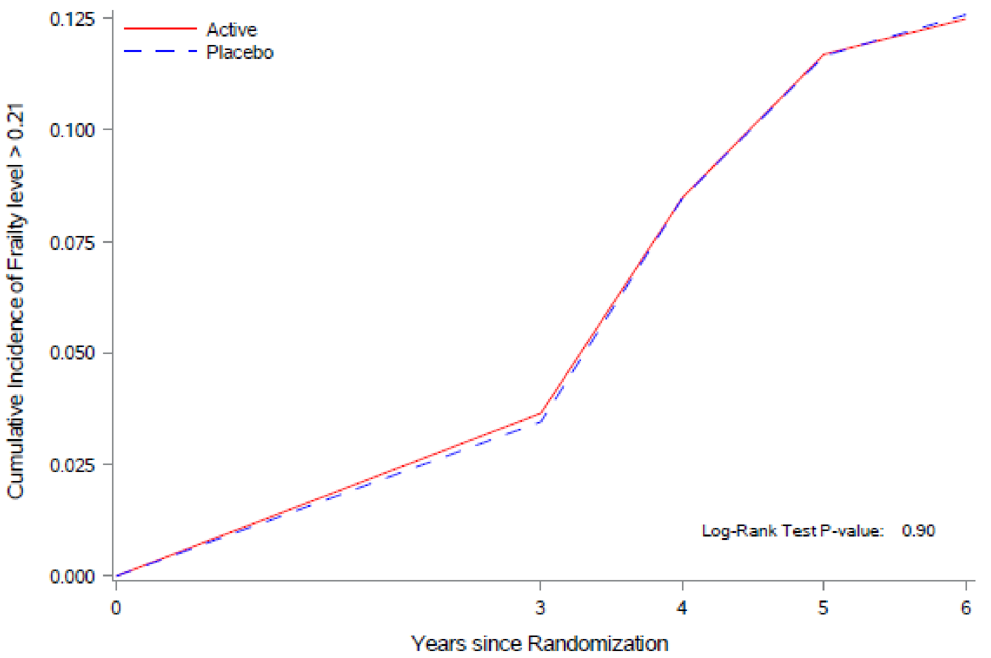

No. at Risk

Placebo	12560	10129	9778	9151	7212
Active	12497	10064	9697	9092	7168

eFigure 4B Effect of omega-3 on incident frailty over time

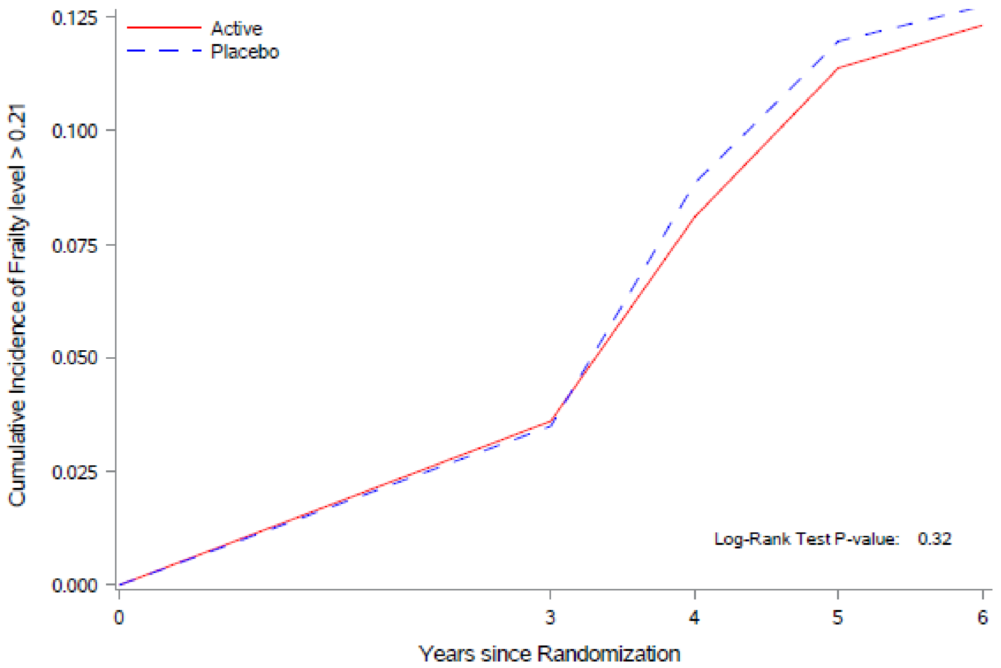

No. at Risk

Placebo	12528	10125	9770	9110	7203
Active	12529	10068	9705	9133	7177

© 2022 Orkaby AR et al. *JAMA Network Open.*

eFigure 5. Vitamin D_3 supplementation and frailty subgroups

a. Vitamin D_3 supplementation and frailty, stratified by age*

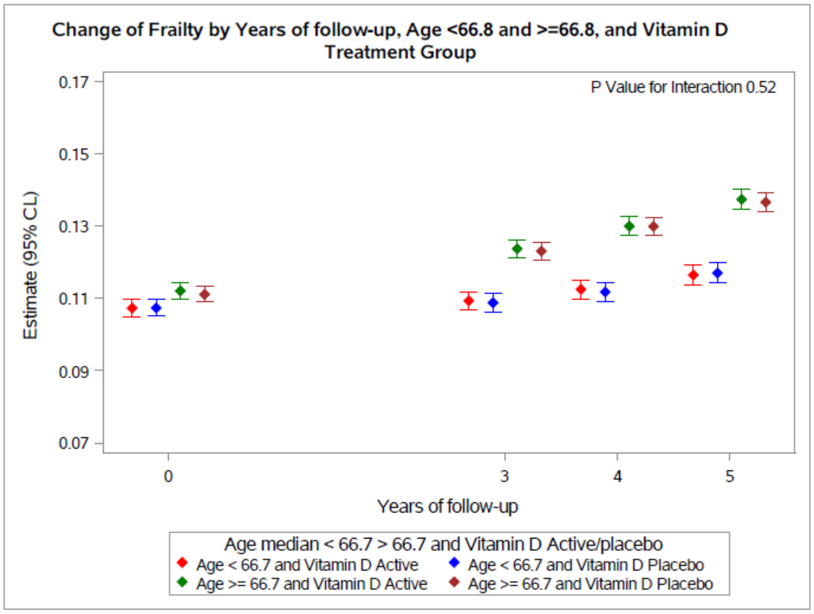

*p-interaction is between vitamin D and median age

b. Vitamin D_3 supplementation and frailty, stratified by sex

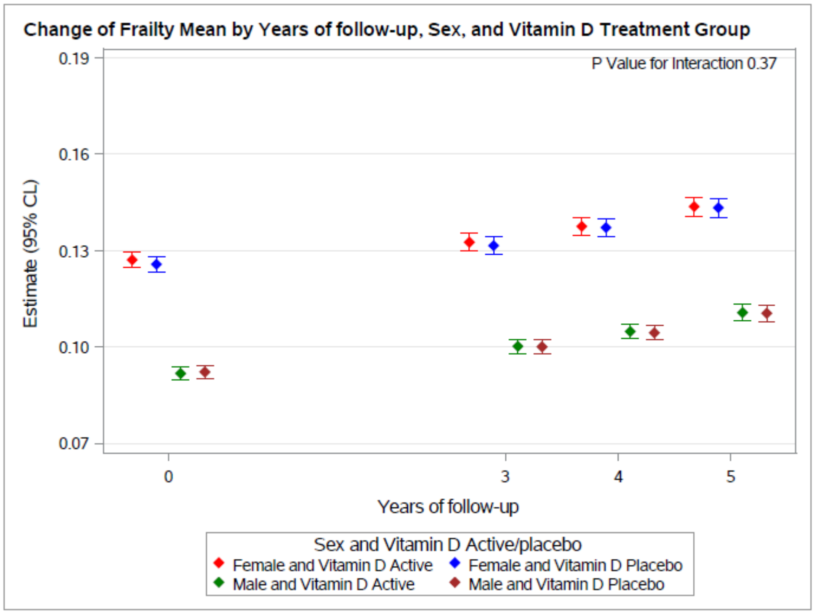

© 2022 Orkaby AR et al. *JAMA Network Open.*

c. Vitamin D_3 supplementation and frailty, stratified by race/ethnicity

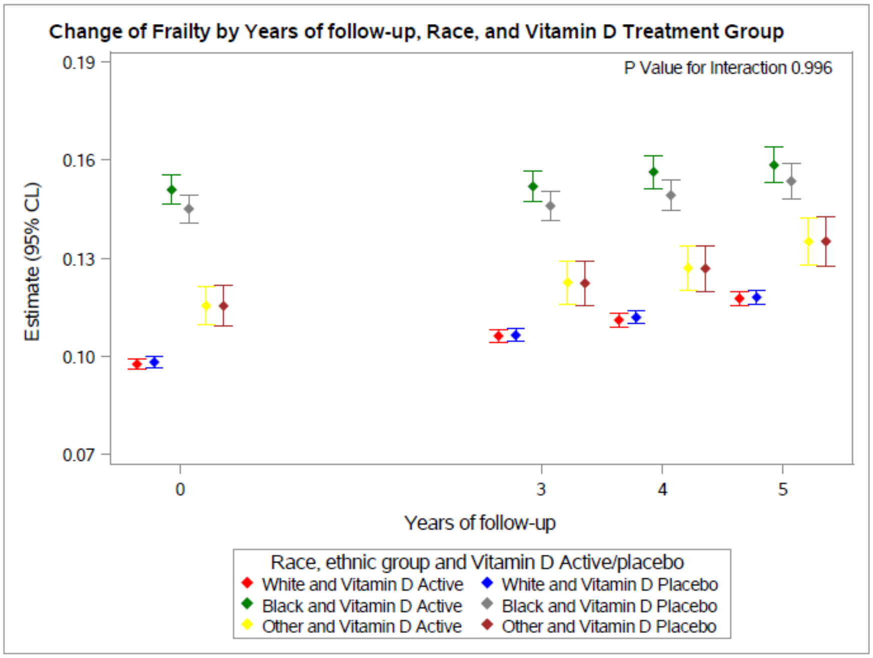

d. Vitamin D_3 supplementation and frailty, stratified by baseline 25(OH)D

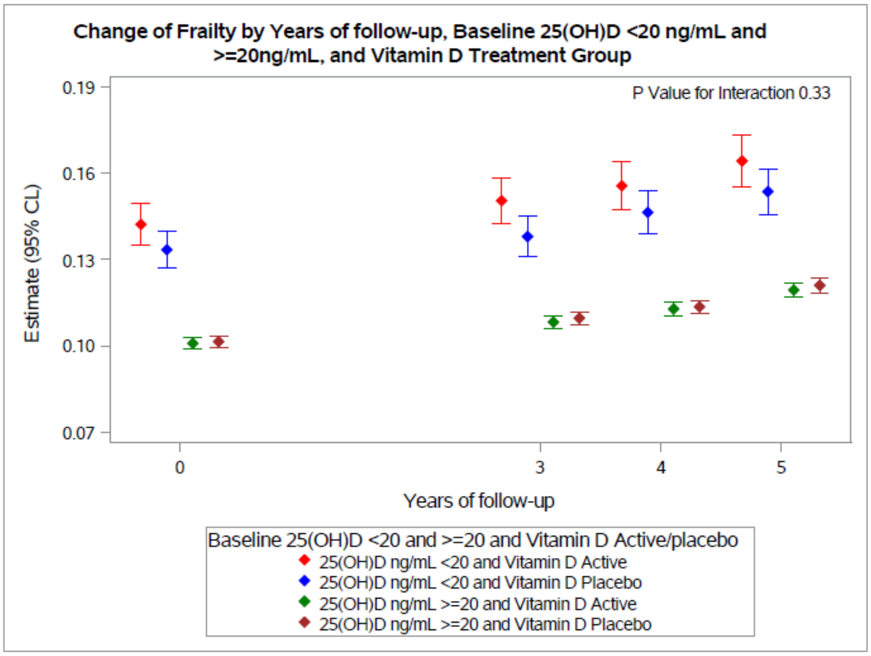

© 2022 Orkaby AR et al. *JAMA Network Open.*

e. Vitamin D3 supplementation and frailty, stratified by BMI

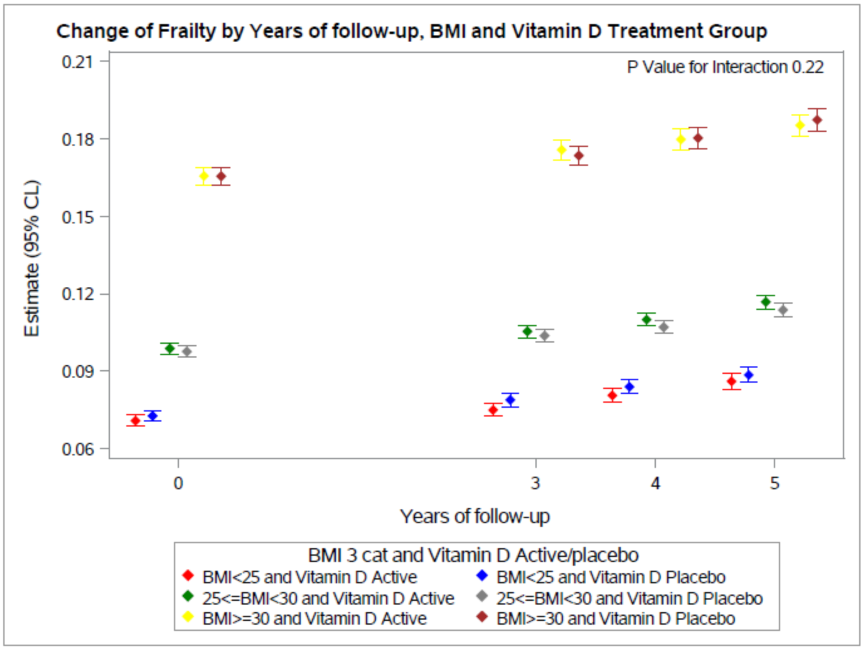

© 2022 Orkaby AR et al. *JAMA Network Open.*

eFigure 6. Omega-3 Supplementation and frailty subgroups

a. Omega 3 supplementation and frailty, stratified by age

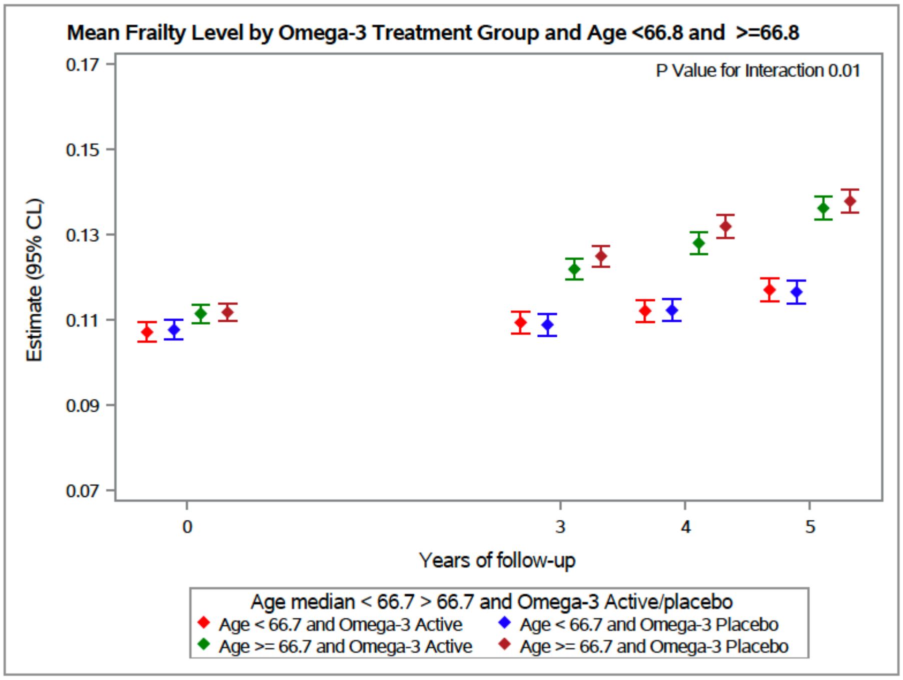

b. Omega 3 supplementation and frailty, stratified by sex

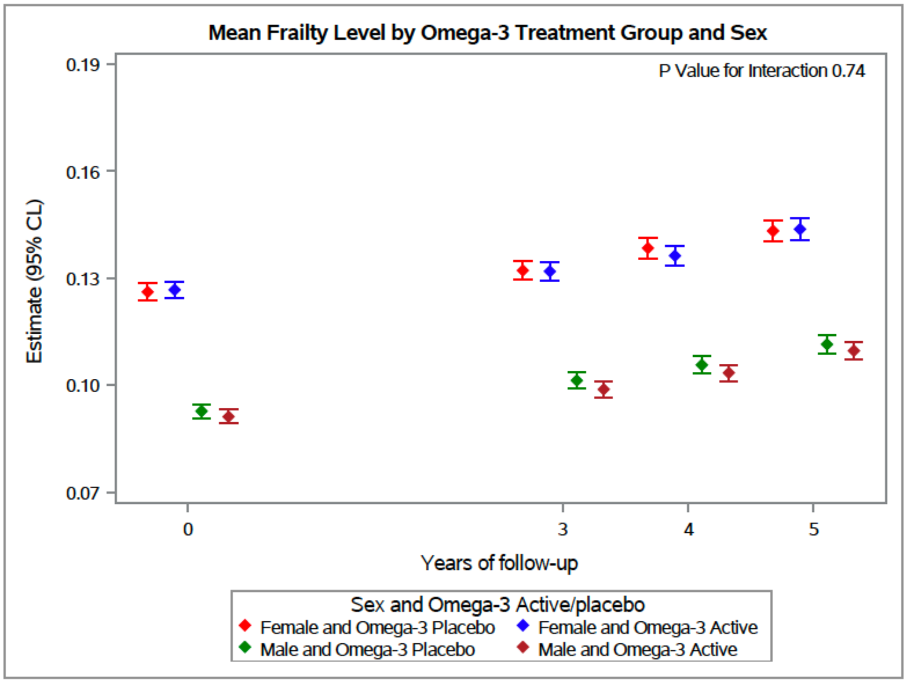

© 2022 Orkaby AR et al. *JAMA Network Open.*

c. Omega 3 supplementation and frailty, stratified by race/ethnicity

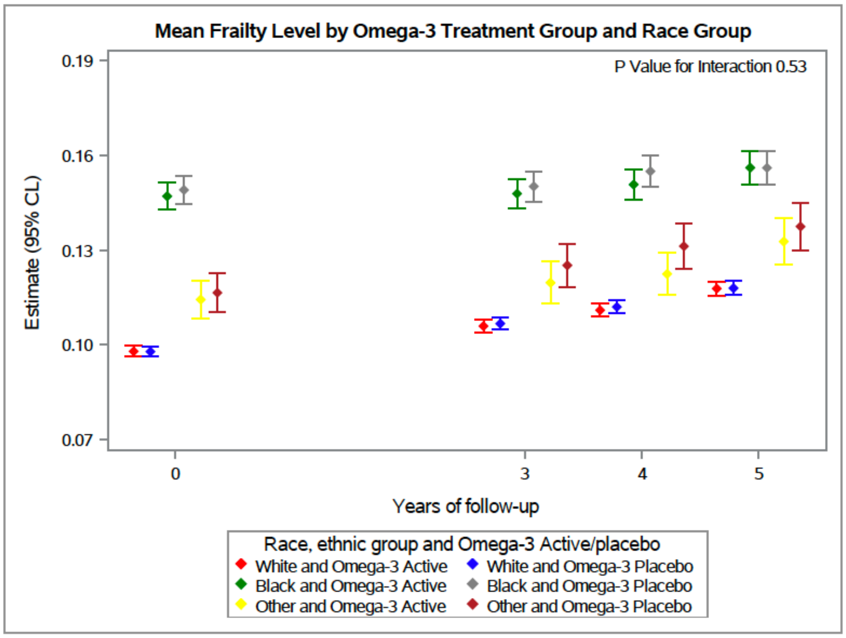

d. Omega 3 supplementation and frailty, stratified by baseline fish intake

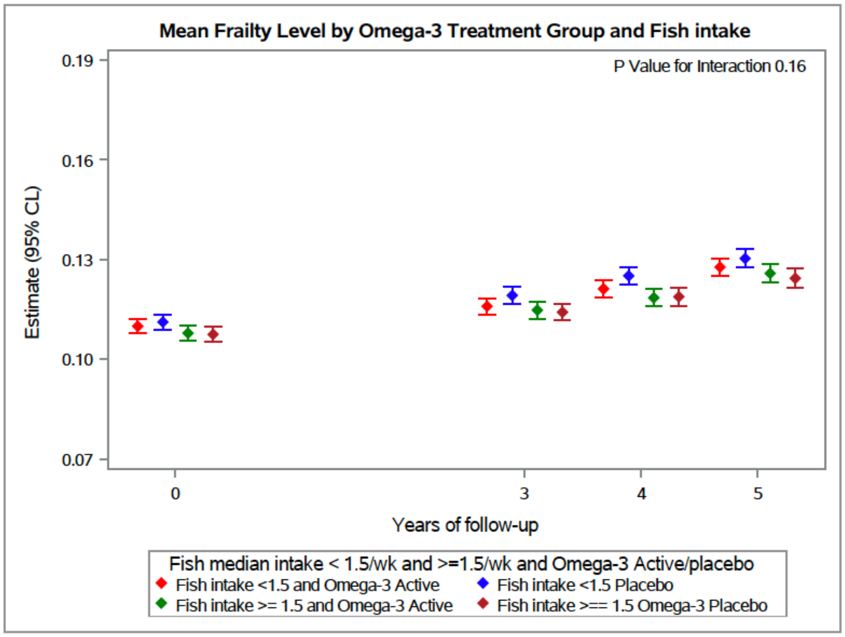

© 2022 Orkaby AR et al. *JAMA Network Open.*

eFigure 7. Effect of Vitamin D_3 on incident Fried frailty over time

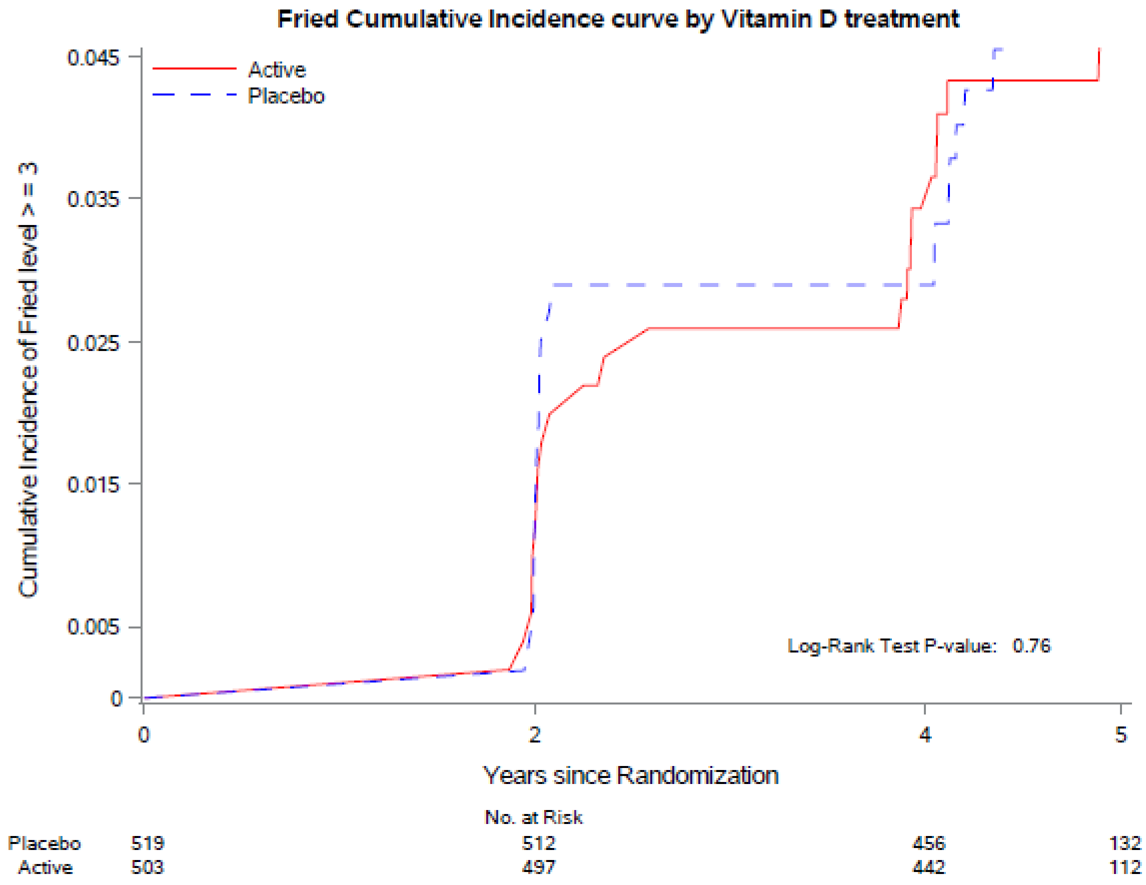

© 2022 Orkaby AR et al. *JAMA Network Open.*

eFigure 8. Effect of omega-3 on incident Fried frailty over time

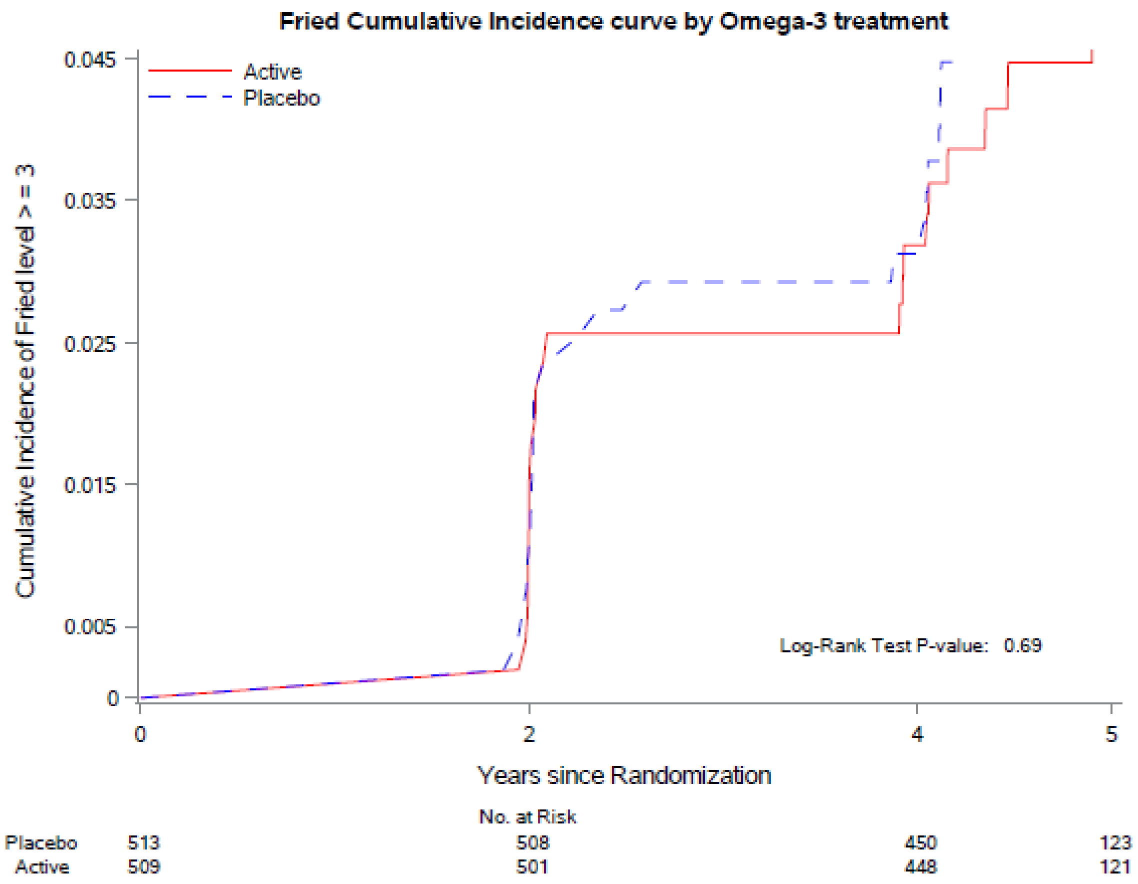

© 2022 Orkaby AR et al. *JAMA Network Open.*

eReferences

1. Searle SD, Mitnitski A, Gahbauer EA, Gill TM, Rockwood K. A standard procedure for creating a frailty index. *BMC geriatrics* 2008; **8**: 24.
2. Rockwood K, Mitnitski A. Frailty in relation to the accumulation of deficits. *The journals of gerontology Series A, Biological sciences and medical sciences* 2007; **62**(7): 722-7.

Data Sharing Statement

Orkaby. Effect of Vitamin D_3 and Omega-3 Fatty Acid Supplementation on Risk of Frailty. *JAMA Netw Open*. Published September 13, 2022. doi:10.1001/jamanetworkopen.2022.31206

Data
Data available: Yes
Data types: Deidentified participant data, Data dictionary
How to access data: The PI of this analysis (aorkaby@bwh.harvard.edu) can be contacted for de-identified data requests
When available: With publication

Supporting Documents
Document types: None

Additional Information
Who can access the data: Researchers where the proposed use of the data is approved as outlined in the data sharing statement
Types of analyses: Analyses will need to be for a specified purpose
Mechanisms of data availability: Analyses proposal requests will require review and approval by the VITAL Publications & Presentations Committee and appropriate IRB approval. Once approved and a data access agreement has been executed, deidentified data generated from this research will be made available to affiliated investigators through secure databases for the prespecified analysis.
