# Supplementary material for: Effect of Vitamin D3 and Omega-3 Fatty Acid Supplementation on Risk of Frailty: An Ancillary Study of a Randomized Clinical Trial
Source: JAMA Netw Open. 2022 Sep 13;5(9):e2231206. doi: 10.1001/jamanetworkopen.2022.31206 (PMC9471979; doi:10.1001/jamanetworkopen.2022.31206)
Supplement: Supplementary file 2 [file jamanetwopen-e2231206-s002.pdf]

TRIAL PROTOCOL AND STATISTICAL ANALYSIS PLAN (SAP)

Supplemental Vitamin D and Incident Fractures in Midlife and Older Adults

This supplement contains the following items:

1. Original protocol. There were no changes made.
2. Original statistical analysis plan. There were no changes made.

I. BACKGROUND

Vitamin D: Vitamin D is synthesized as a pro-hormone in the skin in response to ultraviolet B (UVB) radiation and is also absorbed from the gastrointestinal tract; both sources are activated in the liver to 25-hydroxyvitamin D [25(OH)D] and in the kidney to 1,25-dihydroxyvitamin D. Emerging data show that extra-renal activation of vitamin D occurs in a number of tissues, with potential autocrine and paracrine effects on various tissues, including human marrow stromal cells.[1-5] Circulating serum 25(OH)D levels indicate vitamin D status. Steady-state 25(OH)D levels are lower when there is reduced generation of vitamin D in the skin (higher latitude, winter/late fall seasons), and when there is decreased vitamin D intake or malabsorption.[6, 7] While concentrations of 25(OH)D > 75-80 nmol/L are considered optimal, this threshold may change as we learn more about the effects of higher doses of vitamin D on musculoskeletal and other outcomes.[8-10]

Clinical and epidemiological studies show that inadequate vitamin D levels are common. Elders are at greater risk as their skin has a reduced capacity to produce vitamin D, and they often have limited solar exposure.[11] According to data from National Health and Nutrition Examination Survey III (NHANES III), the prevalence of vitamin D insufficiency in the U.S. was 46% and 35% in white women and men, respectively and 89% and 82% in black women and men.[12] Black individuals are at higher risk than white individuals because darkly pigmented skin is less able to generate vitamin D in response to UVB radiation and they tend to have lower vitamin D intakes.[13, 14] Overweight individuals are also at greater risk, possibly from decreased availability of this fat-soluble vitamin.[15, 16] Moreover, 50% of postmenopausal women with osteoporosis have vitamin D insufficiency.[17] In the osteoporotic fractures in men (MrOS) study, 72% of older men had vitamin D insufficiency.[18, 19] In studies of elders with hip fractures, 96% of women and men in Baltimore and Boston, and in a multinational study 90% had vitamin D insufficiency.[20-22] In the WHI-Observational (WHI-OS) study, low 25(OH)D levels (<47 nmol/L) were associated with a 40% increased hip fracture risk compared with higher levels of 25(OH)D.[23] Thus, vitamin D insufficiency is a major public health problem associated with fractures that may be modified by high doses of supplemental vitamin D.

Vitamin D and Fractures: Data from observational studies and randomized clinical trials (RCTs) suggest the protective effects of vitamin D supplements against fracture, but the evidence is inconsistent.[24, 25], [26] In a 2005 meta-analysis of 5 RCTs for hip fractures and 7 RCTs for non-vertebral fractures, supplementation with oral vitamin D3 700-800 IU/d appeared to reduce the risk of any non-vertebral fracture by 23% and hip fracture

by 26% in older adults, but 400 IU/d was not sufficient to elicit fracture prevention.[27] A meta-regression analysis revealed anti-fracture effects at higher achieved circulating 25(OH) vitamin D levels. A meta-analysis of RCTs in 2009 further supports a benefit of vitamin D on fracture reduction. Among studies of women or men > 65 years treated with vitamin D with or without calcium, the pooled analysis of 12 double-blind RCTs for non-vertebral fractures and 8 RCTs for hip fractures showed a dose-dependent effect of vitamin D on fractures, with daily doses above 400 IU/d reducing non-vertebral fractures by 20% and hip fractures by 19% (from 5 trials).[28] An analysis of community-dwelling older adults showed that higher doses of vitamin D reduced non-vertebral fractures by 29% and hip fractures by 21%.[28] Although, recent meta-analyses indicate that the anti-fracture efficacy of vitamin D is most effective at doses of vitamin >700 IU/d and the higher achieved 25-(OH)D level, primary prevention RCTs with 2000IU/d of vitamin D are lacking.

Rationale for Ancillary Study of High-dose Vitamin D and Prevention of Fractures

There has been considerable controversy regarding the effects of vitamin D on fractures and falls. Study designs according to an evidence-based review are inconsistent, often without analysis of baseline or achieved vitamin D levels, gender or race/ethnicity, or effects of vitamin D alone independent from those combined with calcium supplementation[24, 25] In an overview of the NIH conference on "Vitamin D and Health in the 21st Century: An Update," an evidence-based report indicated that "the available evidence on the relation of 25(OHD), functional health, and adverse outcomes has significant limitations because most studies have been short term, have failed to consider important confounders such as baseline vitamin D status, BMI, and did not study key populations."[24, 29] In an editorial, Dawson-Hughes and other panelists on the optimal vitamin D dose for bone concluded that "more data are needed on the utility of vitamin D doses beyond 20 mcg (800 IU)/day."[10] Previously, we found occult vitamin D deficiency among postmenopausal women with acute hip fractures.[22] In subsequent analyses, vitamin insufficiency [25(OH)D level < 80 nmol/L] was found in 96% of 110 hip fracture subjects from Boston and Baltimore, and 38% had extremely low levels (< 22.5 mmol/L).[20] Other studies affirmed the high incidence of low vitamin D levels in subjects with hip fractures including recent evidence of vitamin D insufficiency in 90% of hip fracture subjects in a multi-national zoledronic acid study.[21] Prior studies from overseas had indicated low vitamin D levels in patients with hip fractures, but this was not expected in the U.S. because of vitamin D fortification of milk.[30, 31] Even when compared with osteoporotic women without hip fractures, those with hip fractures had significantly more vitamin D-deficiency and secondary hyperparathyroidism.[32] Those findings raised awareness of the prevalence of vitamin D-deficiency in the U.S.[33-35] We also found that, in a series of postmenopausal women with advanced osteoarthritis, 25% had occult osteoporosis and 46% were vitamin D-deficient.[36] Our findings reject the hypothesis that osteoarthritic women

are protected against bone loss, but the impact of vitamin D status on either condition is not understood. From our evidence on the high prevalence of vitamin D deficiency in hip fracture patients, Dr. LeBoff and Dr. Glowacki worked to advance fracture care at Brigham and Women's Hospital (BWH) with implementation of an interdisciplinary fracture pathway between Endocrinology and Orthopedic Surgery now called the Brigham Fracture Intervention Team Initiative or "BFit".[35, 37, 38] A greater understanding of the effects of high dose daily vitamin D on fracture reduction has the potential to transform fracture care in the U.S. To understand the effects of high-dose daily vitamin D supplementation on fractures, double-blind RCTS with large sample sizes and long duration designed specifically to assess fractures and falls are needed. To our knowledge, no such trials in the U.S. are either ongoing or have been completed.

Omega-3 Fatty Acids and Skeletal Health: Vitamin D, calcium, and protein are among the dietary essentials for skeletal health. Fatty fishes are the predominant source of the omega-3 (n-3) fatty acids. Concerns about recent changes in the dietary balance of omega-6 (n-6) to n-3 fatty acids in the diet [39] have raised interest in their cellular and skeletal effects. Information about the skeletal effects of fish oil is available from animal studies and some RCTs. Dietary fish oil protected mice from bone loss following ovariectomy, and also prevented the elevation of RANKL seen in T-cells from ovariectomized mice supplemented with corn oil.[40] That study supports the view that one of the mechanisms by which dietary n-3 fatty acids reduces bone loss in ovariectomized mice is by inhibition of osteoclast generation and activation. Other research shows direct evidence of beneficial effects on bone formation. Dietary fish oil protected retired breeder rats from bone loss following ovariectomy, with a marked increase in mineral apposition rate.[41]

In many animal studies, n-3 fatty acids or a low ratio of n-6 to n-3 fatty acids show a positive influence on bone. Bone loss was inhibited by EPA in ovariectomized rats on a low-calcium diet.[42] Both EPA and DHA prevented increases in bone fragility in diabetic rats; and EPA prevented osteopenia even in diabetic rats fed a low-zinc diet, which was used as a potent accelerator of diabetic osteopenia.[43] Subsequently, it was shown that n-3 polyunsaturated fatty acid (PUFA) deficiency caused severe osteoporosis in rats. Further, when deficient animals were replenished with n-3 PUFA, the ratio of n-3 to n-6 PUFA in bone compartments was restored and the process of bone degradation was reversed.[44] The beneficial effects of n-3 fatty acids appear to be associated with down regulation of PGE2 formation with a net enhancement of bone formation and diminished resorption.[45] Although not all studies show beneficial skeletal effects of n-3 in ovariectomized rats[46] it has been suggested that study design and the type of fatty acid in the diet may be more important than the overall ratio of n-3 to n-6 PUFA.[47] A recent study with intact female mice showed that long-term intake of n-3 fatty acids, especially EPA, improved structural and mechanical properties of cortical

bone in the femur, without detectable effects on age-related loss of trabecular bone or BMD.[48] A recent review concluded that most animal studies confirm the beneficial effects of n-3 fatty acids on bone health.[49] Differences in lipoprotein metabolism between animals and humans may limit understanding the biochemical basis of some effects of n-3 fatty acids and observed differences between marine and plant n-3 fatty acids.[50, 51]

RCTs in humans are limited. Results from a small number of studies in humans are inconclusive and vary in type, concentration, and dosing of n-3 fatty acids. In a randomized trial of 40 patients with osteoporosis, subjects taking a supplement rich in n3 PUFA showed better calcium absorption and increased markers of bone formation, while those taking a placebo did not.[52] In a clinical trial of 65 elderly women whose diet was low in calcium, supplementation with a properly balanced ratio of n-6 and n-3 PUFA plus calcium decreased bone turnover and increased BMD. Subjects receiving linoleic acid +EPA treatment for 18-months showed increases of 3.1% in lumbar spine density and 4.7% in femoral BMD.[53] In contrast, 12-month trials with a combination supplement of n-6 PUFA, n-3 PUFA, and calcium in pre- and post-menopausal women failed to affect BMD,[54] possibly attributable to the trial's length, and its use of only total-body BMD measurements. One investigation suggested that PUFAs are actually harmful to bone in peri-menopausal women.[55] To control for confounders due to background diets, a three-period cross-over feeding trial was designed in adults with 3 test diets: an average American diet and two high-PUFA diets with different ratios of n6/n-3 fatty acids.[56] Bone turnover was reduced and bone formation was maintained following the diet with the lowest n-6/n-3 composition. In a recent observational study among elders in an assisted living facility, greater self-reported intakes of n-3 fatty acids were associated with higher BMD.[57] There was no association between physical function and intakes of n-3 fatty acids. These data suggest a possible effect of omega-3 fatty acids on bone health, but definitive studies are necessary. This ancillary study will test whether omega-3 fatty acids reduce fracture.

Significance of this Ancillary Study: The proposed ancillary study is significant because 1) with the growing older population it is important to investigate modifiable factors affecting fractures and bone loss; 2) high dose vitamin D (2000 IU/d) has not been tested for its skeletal effects in a large primary prevention trial; 3) small studies suggest that omega-3 fatty acids may also have beneficial skeletal effects, but primary prevention trials for their effects on preventing fractures are lacking; 4) complementary and cost-effective use of the VITAL primary prevention trial makes it possible to test the effects of high doses of daily vitamin D on fractures 5) results of this proposed ancillary study will help guide clinical practice and public health guidelines related to correction of vitamin D insufficiency 6) these nutritional supplements may offer low-cost preventative interventions; and 7) if the proposed Aims are achieved, high dose daily

vitamin D and/or omega-3 fatty acids may reduce the burden of osteoporotic fractures, with the potential for substantial individual and public health benefits.

II. SPECIFIC AIMS

In the overall cohort (N=40,000), to test whether Vitamin D3 and/or EPA+DHA supplementation
a) will reduce incident total fractures according to annual questionnaires, medical record review, and fracture adjudication.
b) will reduce incident hip and non-vertebral fractures, when examined separately and according to annual questionnaires, medical record review, and fracture adjudication.

III. SUBJECT SELECTION

VITAL will be conducted among 40,000 apparently healthy participants. Included in the trial will be men aged ≥60 and women aged ≥65, ages at which rates of chronic disease increase substantially. All participants will need to have minimally a high school education (to complete mail-based questionnaires). Excluded from the study will be individuals who have (1) a history of cancer (except non-melanoma skin cancer), MI, stroke, TIA, angina, CABG, or PCI; (2) have any of the following safety exclusions: use of anticoagulants at baseline, history of kidney stones, renal failure or dialysis, hypercalcemia, hypo- or hyperparathyroidism, severe liver disease (cirrhosis), or sarcoidosis or other granulomatous diseases such as active chronic tuberculosis or Wegener's granulomatosis; (3) have an allergy to fish (for EPA+DHA); (4) have another serious illness that would preclude participation; (5) are consuming greater than 800 IU of vitamin D from all supplemental sources combined (individual vitamin D supplements, calcium+vitamin D supplements, medications with vitamin D [e.g., Fosamax Plus D], and multivitamins), or, and are not willing to decrease or forego such use during the trial (to ensure total supplemental vitamin D intake does not exceed the current safety limit [i.e., NOAEL] of 4000 IU/d set by the European Commission Scientific Committee on Food (EC SCF)); (6) are consuming greater than 1200 mg/d of calcium (the RDA for individuals aged >50[58]) from all supplemental sources combined; (7) are taking fish oil supplements and are not willing to forego their use during the trial; and (8) are not willing to participate, as evidenced by not signing the informed consent form. Although thiazide diuretics and supplemental vitamin D may interact to increase hypercalcemia risk, it is unlikely that such an interaction would occur at the vitamin D dose we propose.[59] Thus, thiazide use will not be an exclusion criterion.

These procedures will be conducted by the ***VITAL*** study staff.

IV. ENROLLMENT

These procedures will be conducted by the VITAL study staff.

V. STUDY PROCEDURES

Basic study procedures will be carried out by the VITAL study staff. The ancillary procedures are below.

All eligible subjects for the main study will receive annual questionnaires on fractures, and will be adjudicated on fractures as described below.

Does vitamin D3 and/or EPA+DHA supplementation reduce fractures?

Endpoint ascertainment and validation: The primary endpoint of the proposed ancillary study will be reduction of incident total, non-vertebral, and hip fractures. Fractures of head, shoulder/humerus/wrist/fingers, spine, rib/chest/sternum, pelvis, coccyx, leg, and ankle/foot/toes will be self-reported according to procedures used in the WHI.[70] Self-reported fractures will be confirmed with detailed medical record review for fracture validation and adjudication. Participants who self-report a fracture will be asked to sign a medical release form authorizing ***VITAL*** ancillary staff to obtain hospital/physician records. The request will be accompanied by a cover letter expressing concern for the diagnosis and explaining HIPAA guidelines and the scientific importance of validating study records. Non-responders will be sent 2 additional requests for the medical release form according to the parent RCT procedures. During the 10-year WHS trial, only 5% of cases refused to sign a medical release. After the release is obtained, a copy will be sent to the treating hospital/physician. In the WHS, provided that the release form was in order, the staff was able to obtain 99% of hospital/physician records. The Endpoints Committee of physicians, blinded to the randomized treatment assignment, will review the file and, using a defined protocol, will confirm or disconfirm the case. ***Medical Record Review:*** Medicare is a U.S. federal health insurance program that reimburses medical costs for most citizens and permanent residents aged >65 years, as well as some younger people with disabilities. On an annual basis, we will obtain Identifiable (Medicare) Data Files through ResDAC (www.resdac.unm.edu/Index.asp), a Centers for Medicare and Medicaid Services (CMS) contractor that provides free assistance to academic and non-profit researchers interested in using Medicare or Medicaid data (see Appendix B). For each hospitalization, we will obtain data on age, sex, race, admission date, discharge date, length of stay, date of death, principal and additional diagnoses using ICD-9-CM codes, and discharge status. Emergency room visits will be obtained from MedPAR, if there was a hospital admission; and from the Standard Analytical Files (SAF) if there was no admission. SAF records are based on inpatient and outpatient claims. Data from physician claims include a Common Procedure Terminology code for

each billed procedure that must be accompanied by an ICD-9-CM diagnosis code describing the reason for the service.[71, 72]

Questionnaires and Assessments: All participants will be sent yearly questionnaires on medical history, calcium and vitamin D intake (diet and supplements), sun exposure, physical activity, detailed nutritional intakes, lifestyle variables, falls, and risk factors for osteoporosis,.[73-83] Risk factor assessments for osteoporosis include questions about a fracture history in parent or other first-degree family members, and self history of fracture >age 50 (see Appendix A). Participants will be questioned whether they had history of certain medical conditions including premature menopause, diabetes, rheumatoid arthritis, hypo-hyperthyroidism, hypo/hyperparathyroidism, bowel diseases, celiac disease, inflammatory bowel disease, or gastrointestinal (including bariatric) surgery. The participants will also be asked about the current use of: oral diabetes medications, insulin, corticosteroids, diuretics, thyroid hormone, anti-convulsant medications, hormone therapy, bisphosphonates, proton pump inhibitors, selective-serotonin reuptake inhibitors, aromatase inhibitors, or anti-androgens and other medications that affect bone. ***Adjudication of Fractures:*** The adjudication process will include classification of all incident fracture events. The ancillary study investigators at BWH will collect fracture records, including x-ray reports and orthopedic notes, for each non-vertebral fracture reported during the trial. Fractures will be characterized by physician adjudication as traumatic if the circumstances included a fall from a more than a standing height.[70, 84, 85] The following are the fracture adjudication procedures: completion of the fracture event form for all non-vertebral fracture events reported during the proposed 4-year ancillary study; tracking of documents for case packets; and clarification of data queries. The ***VITAL*** ancillary study investigators will process all fracture reports according to the procedures documented in the manual of operations and provide validation and adjudication of fracture location and diagnosis.[66, 70, 84, 86] As reported in the WHIOS, the validity of self-reports is high, as agreement with medical records was 78% for hip and 81% for forearm/wrist fractures.[84]

Quality and Accuracy: BWH ancillary study investigators will be responsible for the quality and accuracy of the study database, data entry on the study forms, and identification and correction of inconsistent or missing data. Final review of the data will be performed by the Endpoints Committee.

VI. BIOSTATISTICAL ANALYSIS

Data Analysis for Fracture Study: Analyses of treatment effects will be based on the intent-to-treat principle. The first analysis will compare baseline characteristics by randomized treatment assignment to ensure that balance has been achieved by the randomization. This will be done in the total cohort. Risk factors for fractures to be assessed will include age; gender; race/ethnicity; parental history of a hip fracture; rheumatoid arthritis; BMI; smoking; alcohol use; the many medical conditions

associated with fragility fractures; and baseline vitamin D, omega-3 fatty acid, and calcium intakes.[23, 78, 79, 83, 87, 88] This fracture ancillary study will also assess baseline distributions of calcium intake, prior falls and fractures, physical activity level, physical function, use of a walking device, and use of medications such as glucocorticoids and antidepressants. The large sample size, as well as successful balance of known potential confounders, assures that unmeasured or unknown potential confounders will be equally distributed across randomized treatment groups, in the total cohort.

Analyses in the Total Cohort: In this 2x2 factorial design, the primary aim is to compare the main effects of intention-to-treat with vitamin D with fish oil on the study outcomes. For the time-to-event outcomes of fractures, falls, and the development of frailty, we will use the Cox proportional hazards model to allow for variable follow-up lengths,[89] and will estimate the hazard ratio for each intervention using indicators for treatment exposure, controlling for the second intervention, age, and gender. The primary analysis will estimate the cause-specific hazard and the hazard ratio comparing intervention groups for each outcome of interest by censoring individuals with deaths due to competing causes. We will also adjust for any imbalances by treatment group in baseline risk factors, particularly age, smoking, alcohol, BMI, physical activity, fall history, and calcium intake. Beyond the primary analyses of these outcomes, we will examine effect modification by the other randomized intervention, by baseline risk factors, and by time. We will explore interactions between the interventions and with the baseline dietary intake of vitamin D, calcium, and EPA+DHA in the entire cohort (N=20,000) and plasma levels of 25(OH)D, and EPA+DHA in 3,000 subjects. We hypothesize that the intervention effects will be larger among those with below-median baseline levels, and we will analyze treatment effects by quartiles of these nutrients. We will also evaluate effect modification by gender, race/skin pigmentation (for vitamin D), and BMI (for vitamin D). The latter interaction effects will be interpreted cautiously, as hypothesis generating. During the course of the trial, it is possible that an imbalance will emerge due to an effect of one of the agents on cancer or CVD disease, which may in turn lead to a difference in fractures or falls. Our primary analysis will remain an intent-to-treat analysis looking at any of these outcomes without regard to such intervening effects. In order to more thoroughly examine the causal relations and intervening mechanisms, we will also conduct a secondary analysis adjusting for the time-varying effects of such intervening events in a Cox model. We will further determine whether there is effect modification of the intervention effects among those who develop cancer

Table 1. Power for fracture in the total cohort.

Observed RR	True RR	Any Fracture	Non-Vertebral Fracture	Hip Fracture	Vertebral Fracture
0.90	0.875	73.7	71.6	17.4	12.7
0.85	0.812	97.6	97.0	34.2	23.3
0.80	0.750	>99.9	>99.9	55.5	38.2
0.75	0.687	>99.9	>99.9	75.8	55.7
0.70	0.625	>99.9	>99.9	89.9	72.6
0.65	0.560	>99.9	>99.9	96.9	85.8
0.60	0.500	>99.9	>99.9	99.4	94.0

or a CVD event using time-varying interaction terms. Alternatively, we will censor at the occurrence of these events, treating them as competing events and estimating the effect on fractures in their absence. These adjusted analyses will be regarded as exploratory.

Power for Ancillary Study: Power calculations for analysis of time-to-event outcomes in the entire cohort, namely fractures, falls, and frailty, are based on assumptions made for the parent grant, including a 2x2 factorial trial of 20,000 women and men. Power for bone loss outcomes assessed within the CTSC sub-cohort is based on measurements taken at baseline and at the year 2 follow-up among the 700 participants. For the CTSC sub-cohort we have conducted power calculations allowing for 15% and 20% drop out, or sample sizes of 595 and 560.

Fractures: Power for the primary aim of fractures is shown in Table 1. Rates are based on age-specific rates from the large population-based studies SOF (women) and MrOS (men).[78, 87, 90, 91] The cited reductions are the observed effects we would see. The corresponding 'true' RR is given assuming an average compliance of 80%. Power is shown for a two-sided test using a log-rank analysis[92] with a significance level of 0.05. Assuming that only one agent is effective, with 5 years of treatment and follow-up we will have 80% power to detect an observed RR of 0.89 (true RR = 86% if 80% compliance) for any fractures, RR of 0.88 (true RR=0.85) for clinical non-vertebral fractures, RR of 0.73 (true RR= 0.66) for hip fractures and RR of 0.67 (true RR = 0.59) for clinical vertebral fractures. These effect sizes, especially for any or non-vertebral fractures, are consistent with the 19-29% reductions seen in meta-analyses or observational studies of higher doses of vitamin D.[28] Power has been shown for a given observed reduction in risk, assuming an average compliance of 80%, similar to that achieved in our previous RCTs. Power for total fracture will be excellent with a 5-year follow-up even if compliance is lower. If the risk reduction is 15%, the observed RR corresponding to compliance of 80% is 0.88, and we have 88% power to detect this level of effect. If compliance is only 70%, the observed RR would be 0.89, with 81% power to detect an effect of this magnitude (Table 1).

VI. RISKS AND DISCOMFORTS

There is also the possibility of social/psychological risk from inadvertent disclosure of confidential information from the questionnaires or blood tests. However, we have many safeguards in place to avoid this possibility, and we have never had an inadvertent breach of confidentiality in any of our trials. The potential risk of disclosure of confidential information is guarded against by maintaining completed questionnaires in locked areas accessible by authorized personnel only. In questionnaire data files, participants are identified only by study ID. Subject identifiers are stored in separate

computer files with password protection, and results will be presented only in the aggregate. In addition, employees involved with the proposed study will be asked to sign a form agreeing not to disclose any information to which they might have access, regardless of their personal perception about its confidentiality. Each employee of the study who has access to study data or has contact with subjects participates in an institutional (Brigham and Women's Hospital) human subjects educational program, which consists of reviewing regulatory and informational documents pertaining to human subjects research; passing a test on ethical principles and regulations governing human subjects research; and signing a statement of commitment to the protection of human subjects. Finally, all such employees are required to participate in HIPAA training.

VII. POTENTIAL BENEFITS:

There are minimal direct benefits to the study participants. The study is intended to advance knowledge about possible benefits of vitamin D and fish oil on fractures, bone density, falls, and frailty. The data from the study will guide clinical practice and public health concerns about use of vitamin D and/or fish oil for bone health and fracture prevention.

IX. MONITORING AND QUALITY ASSURANCE

A. SAFETY MONITORING

An independent Data and Safety Monitoring Board (DSMB) will be assembled, consisting of NIH representatives and experts in clinical trials, epidemiology, biostatistics, orthopedics, endocrinology, and relevant clinical areas of bone and metabolic bone disease. The DSMB will make an annual examination of the progress of the trial and the un-blinded data on study endpoints and possible adverse effects to recommend continuation, alteration of study design, or early termination, as appropriate.

B. MONITORING OF SOURCE DATA AND DRUG DISPENSEMENT

Redundancies will be built into the data processing systems to insure accurate recording of data and proper follow-up. All research forms will be scanned in and the data read by a character-recognition software program (Teleform). Out-of-range, internally inconsistent, and unclear data will be reviewed by a verifier who will edit misread variables. Forms that cannot be scanned, and name and address changes, will be entered using traditional double-entry procedures. All data will undergo additional within-form and across-time checks to verify accuracy. Following data entry, all questionnaire responses that require additional follow-up for missing data, participant

comments on the form or for endpoint validation will be manually reviewed to insure correct processing and accurate follow-up. Address changes received from the post office or from participants will be manually keyed by data entry personnel and the resultant files compared and verified.

1. Adams, J.S., et al., *Vitamin D in defense of the human immune response.* Ann N Y Acad Sci, 2007. 1117: p. 94-105.
2. Chun, R.F., J.S. Adams, and M. Hewison, *Back to the future: a new look at 'old' vitamin D.* J Endocrinol, 2008. 198(2): p. 261-9.
3. Hewison, M., et al., *Extra-renal 25-hydroxyvitamin D3-1[alpha]-hydroxylase in human health and disease.* The Journal of Steroid Biochemistry and Molecular Biology, 2007. 103(3-5): p. 316-321.
4. Holick, M.F., *Vitamin D deficiency.* N Engl J Med, 2007. 357(3): p. 266-81.
5. Zhou, S., M.S. LeBoff, and J. Glowacki, *Vitamin D metabolism and action in human bone marrow stromal cells.* Endocrinology. 151(1): p. 14-22.
6. Holick, M.F. and T.C. Chen, *Vitamin D deficiency: a worldwide problem with health consequences.* Am J Clin Nutr, 2008. 87(4): p. 1080S-6S.
7. Heaney, R.P., *Vitamin D and calcium interactions: functional outcomes.* Am J Clin Nutr, 2008. 88(2): p. 541S-544S.
8. Giovannucci, E., *The epidemiology of vitamin D and cancer incidence and mortality: a review (United States).* Cancer Causes Control, 2005. 16(2): p. 83-95.
9. Zerwekh, J.E., *Blood biomarkers of vitamin D status.* Am J Clin Nutr, 2008. 87(4): p. 1087S-91S.
10. Dawson-Hughes, B., et al., *Estimates of optimal vitamin D status.* Osteoporos Int, 2005. 16(7): p. 713-6.
11. MacLaughlin, J. and M.F. Holick, *Aging decreases the capacity of human skin to produce vitamin D3.* J Clin Invest, 1985. 76(4): p. 1536-8.
12. Zadshir, A., et al., *The prevalence of hypovitaminosis D among US adults: data from the NHANES III.* Ethn Dis, 2005. 15(4 Suppl 5): p. S5-97-101.
13. Harris, S.S., *Vitamin D and African Americans.* J Nutr, 2006. 136(4): p. 1126-9.
14. Moore, C.E., M.M. Murphy, and M.F. Holick, *Vitamin D intakes by children and adults in the United States differ among ethnic groups.* J Nutr, 2005. 135(10): p. 2478-85.
15. Martins, D., et al., *Prevalence of cardiovascular risk factors and the serum levels of 25-hydroxyvitamin D in the United States: data from the Third National Health and Nutrition Examination Survey.* Arch Intern Med, 2007. 167(11): p. 1159-65.
16. Harris, S.S. and B. Dawson-Hughes, *Reduced sun exposure does not explain the inverse association of 25-hydroxyvitamin D with percent body fat in older adults.* J Clin Endocrinol Metab, 2007. 92(8): p. 3155-7.
17. Holick, M.F., et al., *Prevalence of Vitamin D inadequacy among postmenopausal North American women receiving osteoporosis therapy.* J Clin Endocrinol Metab, 2005. 90(6): p. 3215-24.
18. Holick, M.F., *MrOs is D-ficient.* J Clin Endocrinol Metab, 2009. 94(4): p. 1092-3.
19. Orwoll, E., et al., *Vitamin D deficiency in older men.* J Clin Endocrinol Metab, 2009. 94(4): p. 1214-22.
20. LeBoff, M.S., et al., *Vitamin D-deficiency and post-fracture changes in lower extremity function and falls in women with hip fractures.* Osteoporos Int, 2008. 19(9): p. 1283-90.

21. Pieper, C.F., et al., *Distribution and correlates of serum 25-hydroxyvitamin D levels in a sample of patients with hip fracture.* Am J Geriatr Pharmacother, 2007. 5(4): p. 335-40.
22. LeBoff, M.S., et al., *Occult vitamin D deficiency in postmenopausal US women with acute hip fracture.* Jama, 1999. 281(16): p. 1505-11.
23. Cauley, J.A., et al., *Serum 25-hydroxyvitamin D concentrations and risk for hip fractures.* Ann Intern Med, 2008. 149(4): p. 242-50.
24. Cranney, A., et al., *Summary of evidence-based review on vitamin D efficacy and safety in relation to bone health.* Am J Clin Nutr, 2008. 88(2): p. 513S-519S.
25. Cranney, A., et al., *Effectiveness and safety of vitamin D in relation to bone health.* Evid Rep Technol Assess (Full Rep), 2007(158): p. 1-235.
26. Feskanich, D., W.C. Willett, and G.A. Colditz, *Calcium, vitamin D, milk consumption, and hip fractures: a prospective study among postmenopausal women.* Am J Clin Nutr, 2003. 77(2): p. 504-11.
27. Bischoff-Ferrari, H.A., et al., *Fracture prevention with vitamin D supplementation: a meta-analysis of randomized controlled trials.* Jama, 2005. 293(18): p. 2257-64.
28. Bischoff-Ferrari, H.A., et al., *Prevention of nonvertebral fractures with oral vitamin D and dose dependency: a meta-analysis of randomized controlled trials.* Arch Intern Med, 2009. 169(6): p. 551-61.
29. Brannon, P.M., et al., *Overview of the conference "Vitamin D and Health in the 21st Century: an Update".* Am J Clin Nutr, 2008. 88(2): p. 483S-490S.
30. Boonen, S., J. Aerssens, and J. Dequeker, *Age-related endocrine deficiencies and fractures of the proximal femur. II implications of vitamin D deficiency in the elderly.* J Endocrinol, 1996. 149(1): p. 13-7.
31. Harma, M., et al., *Bone density, histomorphometry and biochemistry in patients with fractures of the hip or spine.* Ann Clin Res, 1987. 19(6): p. 378-82.
32. LeBoff M.S., et al., *Compared with osteoporotic controls acute hip fracture patients show high PTH and low vitamin D levels.*, in *79th Annual Meeting of The Endocrine Society*. 1997.
33. Simon, J., et al., *Fractures in the elderly and vitamin D.* J Nutr Health Aging, 2002. 6(6): p. 406-12.
34. Glowacki, J. and L. M.S., *Vitamin D insufficiency and fracture risk.* Current Opinion in Endocrinology and Diabetes, 2004. 11(6): p. 353-358.
35. Glowacki, J., et al., *Importance of vitamin D in hospital-based fracture care pathways.* J Nutr Health Aging, 2008. 12(5): p. 291-3.
36. Glowacki, J., et al., *Osteoporosis and vitamin-D deficiency among postmenopausal women with osteoarthritis undergoing total hip arthroplasty.* J Bone Joint Surg Am, 2003. 85-A(12): p. 2371-7.
37. Lewiecki, E.M., et al., *2008 santa fe bone symposium: update on osteoporosis.* J Clin Densitom, 2009. 12(2): p. 135-57.
38. Harris, M.B., et al., *Evolution of comprehensive hospital care pathways to advance the treatment of fragility fractures.* The Orthopaedic Journal at Harvard Medical School, 2007(9): p. 95-97.
39. Simopoulos, A.P., *Evolutionary aspects of omega-3 fatty acids in the food supply.* Prostaglandins Leukot Essent Fatty Acids, 1999. 60(5-6): p. 421-9.
40. Sun, D., et al., *Dietary n-3 fatty acids decrease osteoclastogenesis and loss of bone mass in ovariectomized mice.* J Bone Miner Res, 2003. 18(7): p. 1206-16.
41. Matsushita, H., et al., *Dietary fish oil results in a greater bone mass and bone formation indices in aged ovariectomized rats.* J Bone Miner Metab, 2008. 26(3): p. 241-7.

42. Sakaguchi, K., I. Morita, and S. Murota, *Eicosapentaenoic acid inhibits bone loss due to ovariectomy in rats.* Prostaglandins Leukot Essent Fatty Acids, 1994. 50(2): p. 81-4.
43. Yamada, Y., et al., *Effect of eicosapentaenoic acid and docosahexaenoic acid on diabetic osteopenia.* Diabetes Res Clin Pract, 1995. 30(1): p. 37-42.
44. Reinwald, S., et al., *Repletion with (n-3) fatty acids reverses bone structural deficits in (n-3)-deficient rats.* J Nutr, 2004. 134(2): p. 388-94.
45. Watkins, B.A., et al., *Omega-3 polyunsaturated fatty acids and skeletal health.* Exp Biol Med (Maywood), 2001. 226(6): p. 485-97.
46. Poulsen, R.C. and M.C. Kruger, *Detrimental effect of eicosapentaenoic acid supplementation on bone following ovariectomy in rats.* Prostaglandins Leukot Essent Fatty Acids, 2006. 75(6): p. 419-27.
47. Poulsen, R.C., et al., *Specific effects of gamma-linolenic, eicosapentaenoic, and docosahexaenoic ethyl esters on bone post-ovariectomy in rats.* Calcif Tissue Int, 2007. 81(6): p. 459-71.
48. Bonnet, N. and S. Ferrari, *A long-term diet enriched in omega-3 fatty acids improves cortical bone structure and mechanical properties in mice. Abstract#M47*, in *New Frontiers in Skeletal Research: Bone, Fat, and Brain Connections*. April 27-28, 2009: Bethesda, MD.
49. Salari, P., et al., *A systematic review of the impact of n-3 fatty acids in bone health and osteoporosis.* Med Sci Monit, 2008. 14(3): p. RA37-44.
50. Harris, W.S., *n-3 fatty acids and serum lipoproteins: animal studies.* Am J Clin Nutr, 1997. 65(5 Suppl): p. 1611S-1616S.
51. Harris, W.S., *n-3 fatty acids and serum lipoproteins: human studies.* Am J Clin Nutr, 1997. 65(5 Suppl): p. 1645S-1654S.
52. Van Papendrop, D.H., H. Coetzer, and M.G. Kruger, *Biochemical profile of osteoporotic patients on essential fatty acid supplementation.* Nutr Res, 1995(15): p. 325-34.
53. Kruger, M.C., et al., *Calcium, gamma-linolenic acid and eicosapentaenoic acid supplementation in senile osteoporosis.* Aging (Milano), 1998. 10(5): p. 385-94.
54. Bassey, E.J., et al., *Lack of effect of supplementation with essential fatty acids on bone mineral density in healthy pre- and postmenopausal women: two randomized controlled trials of Efacal v. calcium alone.* Br J Nutr, 2000. 83(6): p. 629-35.
55. Macdonald, H.M., et al., *Nutritional associations with bone loss during the menopausal transition: evidence of a beneficial effect of calcium, alcohol, and fruit and vegetable nutrients and of a detrimental effect of fatty acids.* Am J Clin Nutr, 2004. 79(1): p. 155-65.
56. Griel, A.E., et al., *An increase in dietary n-3 fatty acids decreases a marker of bone resorption in humans.* Nutr J, 2007. 6: p. 2.
57. Rousseau, J.H., A. Kleppinger, and A.M. Kenny, *Self-reported dietary intake of omega-3 fatty acids and association with bone and lower extremity function.* J Am Geriatr Soc, 2009. 57(10): p. 1781-8.
58. Institute of Medicine Food and Nutrition Board, *Dietary Reference Intakes: Calcium, Phosphorous, Magnesium, Vitamin D and Fluoride*. 1999, Washington DC: National Academies Press.
59. Hathcock, J.N., et al., *Risk assessment for vitamin D.* Am J Clin Nutr, 2007. 85(1): p. 6-18.
60. Lang, J.M., et al., *Estimating the effect of the run-in on the power of the Physicians' Health Study.* Stat Med, 1991. 10(10): p. 1585-93.

61. Fitzpatrick, T.B., *The validity and practicality of sun-reactive skin types I through VI.* Arch Dermatol, 1988. 124(6): p. 869-71.
62. Binkley, N., et al., *Correlation among 25-hydroxy-vitamin D assays.* J Clin Endocrinol Metab, 2008. 93(5): p. 1804-8.
63. Binkley, N., et al., *Assay variation confounds the diagnosis of hypovitaminosis D: a call for standardization.* J Clin Endocrinol Metab, 2004. 89(7): p. 3152-7.
64. Bischoff-Ferrari, H.A., et al., *Estimation of optimal serum concentrations of 25-hydroxyvitamin D for multiple health outcomes.* Am J Clin Nutr, 2006. 84(1): p. 18-28.
65. Aloia, J.F., et al., *Vitamin D intake to attain a desired serum 25-hydroxyvitamin D concentration.* Am J Clin Nutr, 2008. 87(6): p. 1952-8.
66. Jackson, R.D., et al., *Calcium plus vitamin D supplementation and the risk of fractures.* N Engl J Med, 2006. 354(7): p. 669-83.
67. Bolland, M.J., et al., *Vascular events in healthy older women receiving calcium supplementation: randomised controlled trial.* BMJ, 2008. 336(7638): p. 262-6.
68. Radimer, K., et al., *Dietary supplement use by US adults: data from the National Health and Nutrition Examination Survey, 1999-2000.* Am J Epidemiol, 2004. 160(4): p. 339-49.
69. Satterfield, S., et al., *Biochemical markers of compliance in the Physicians' Health Study.* Am J Prev Med, 1990. 6(5): p. 290-4.
70. *Design of the Women's Health Initiative clinical trial and observational study. The Women's Health Initiative Study Group.* Control Clin Trials, 1998. 19(1): p. 61-109.
71. Klabunde, C.N., et al., *Development of a comorbidity index using physician claims data.* J Clin Epidemiol, 2000. 53(12): p. 1258-67.
72. Fisher, E.S., et al., *The accuracy of Medicare's hospital claims data: progress has been made, but problems remain.* Am J Public Health, 1992. 82(2): p. 243-8.
73. Woods, N.F., et al., *Frailty: emergence and consequences in women aged 65 and older in the Women's Health Initiative Observational Study.* J Am Geriatr Soc, 2005. 53(8): p. 1321-30.
74. Pereira, M.A., et al., *A collection of Physical Activity Questionnaires for health-related research.* Med Sci Sports Exerc, 1997. 29(6 Suppl): p. S1-205.
75. Robbins, J., et al., *Body mass index is not a good predictor of bone density: results from WHI, CHS, and EPIDOS.* J Clin Densitom, 2006. 9(3): p. 329-34.
76. Ensrud, K.E., et al., *Comparison of 2 frailty indexes for prediction of falls, disability, fractures, and death in older women.* Arch Intern Med, 2008. 168(4): p. 382-9.
77. Ensrud, K.E., et al., *A comparison of frailty indexes for the prediction of falls, disability, fractures, and mortality in older men.* J Am Geriatr Soc, 2009. 57(3): p. 492-8.
78. Cummings, S.R., et al., *Risk factors for hip fracture in white women. Study of Osteoporotic Fractures Research Group.* N Engl J Med, 1995. 332(12): p. 767-73.
79. Watts, N.B., B. Ettinger, and M.S. LeBoff, *FRAX facts.* J Bone Miner Res 2009. 24(6): p. 975-979.
80. Israel, E., et al., *Effects of inhaled glucocorticoids on bone density in premenopausal women.* N Engl J Med, 2001. 345(13): p. 941-7.
81. LeBoff M.S. Guest Editor, et al., *The Spectrum of Renal Bone Disease.* Seminars in Nephrology, 2009.
82. Bernstein, C.N., W.D. Leslie, and M.S. Leboff, *AGA technical review on osteoporosis in gastrointestinal diseases.* Gastroenterology, 2003. 124(3): p. 795-841.

83. Leboff, M.S., et al., *Homocysteine levels and risk of hip fracture in postmenopausal women.* J Clin Endocrinol Metab, 2009. 94(4): p. 1207-13.
84. Chen, Z., et al., *Validity of self-report for fractures among a multiethnic cohort of postmenopausal women: results from the Women's Health Initiative observational study and clinical trials.* Menopause, 2004. 11(3): p. 264-74.
85. Chen, Z., et al., *Fracture risk among breast cancer survivors: results from the Women's Health Initiative Observational Study.* Arch Intern Med, 2005. 165(5): p. 552-8.
86. Cauley, J.A., et al., *Effects of estrogen plus progestin on risk of fracture and bone mineral density: the Women's Health Initiative randomized trial.* Jama, 2003. 290(13): p. 1729-38.
87. Orwoll, E., et al., *Design and baseline characteristics of the osteoporotic fractures in men (MrOS) study--a large observational study of the determinants of fracture in older men.* Contemp Clin Trials, 2005. 26(5): p. 569-85.
88. Robbins, J., et al., *Factors associated with 5-year risk of hip fracture in postmenopausal women.* Jama, 2007. 298(20): p. 2389-98.
89. Cox, D.R., *Regression models and life-tables.* J Royal Statist Soc B, 1972. 34(2): p. 187-220.
90. Cummings, S.R. and L.J. Melton, *Epidemiology and outcomes of osteoporotic fractures.* Lancet, 2002. 359(9319): p. 1761-7.
91. Cawthon, P.M., *Personal Communication*, L. M.S., Editor. 2009.
92. Freedman, L.S., *Tables of the number of patients required in clinical trials using the logrank test.* Stat Med, 1982. 1(2): p. 121-9.
93. Haybittle, J.L., *Repeated assessment of results in clinical trials of cancer treatment.* Br J Radiol, 1971. 44(526): p. 793-7.
94. Peto, R., et al., *Design and analysis of randomized clinical trials requiring prolonged observation of each patient. I. Introduction and design.* Br J Cancer, 1976. 34(6): p. 585-612.
95. Geller, N.L. and S.J. Pocock, *Interim analyses in randomized clinical trials: ramifications and guidelines for practitioners.* Biometrics, 1987. 43(1): p. 213-23.
96. O'Brien, P.C. and T.R. Fleming, *A multiple testing procedure for clinical trials.* Biometrics, 1979. 35(3): p. 549-56.
97. DeMets, D.L. and K.K. Lan, *Interim analysis: the alpha spending function approach.* Stat Med, 1994. 13(13-14): p. 1341-52; discussion 1353-6.
98. Pocock, S.J., *Current controversies in data monitoring for clinical trials.* Clin Trials, 2006. 3(6): p. 513-21.
