# Supplementary material for: Effect of Vitamin D3 and Omega-3 Fatty Acid Supplementation on Risk of Frailty: An Ancillary Study of a Randomized Clinical Trial
Source: JAMA Netw Open. 2022 Sep 13;5(9):e2231206. doi: 10.1001/jamanetworkopen.2022.31206 (PMC9471979; doi:10.1001/jamanetworkopen.2022.31206)
Supplement: Supplementary file 3 [file jamanetwopen-e2231206-s003.pdf]

Supplementary Online Content

Orkaby AR, Dushkes R, Ward R, et al. Effect of vitamin D_3 and omega-3 fatty acid supplementation on risk of frailty: an ancillary study of a randomized clinical trial. *JAMA Netw Open*. 2022;5(9):e2231206. doi:10.1001/jamanetworkopen.2022.31206

eTable 1. Cumulative Deficit Frailty Index Components for the VITAL Trial

eTable 2. Fried Physical Phenotype of Frailty in VITAL Subcohort (n=1054)

eTable 3. Baseline Characteristics of the VITAL Trial (n=25 057) According to Frailty Status

eTable 4. Adjusted Means at Baseline and Mean Change (95% CI) in Frailty Level at Each Year Since Randomization Compared to Baseline, According to Vitamin D vs Placebo and Omega-3 vs Placebo Groups

eTable 5. Baseline Characteristics of 1054 Participants in the VITAL Trial in Person Subgroup

eTable 6A. Adjusted Means at Baseline and Mean Change (95% CI) in Fried Frailty Level at Each Year Since Randomization Compared to Baseline, According to Vitamin D and Placebo Groups

eTable 6B. Adjusted Means at Baseline and Mean Change (95% CI) in Fried Frailty Level at Each Year Since Randomization Compared to Baseline, According to n-3 and Placebo Groups

eFigure 1. Distribution of Frailty Index at Baseline

eFigure 2. Distribution of Frailty Index at Baseline According to Randomization Group

eFigure 3. Validation of the FI: Association Between FI Level and Mortality

eFigure 4A. Effect of Vitamin D_3 on Incident Frailty Over Time

eFigure 4B. Effect of Omega-3 on Incident Frailty Over Time

eFigure 5. Vitamin D_3 Supplementation and Frailty Subgroups

eFigure 6. Omega-3 Supplementation and Frailty Subgroups

eFigure 7. Effect of Vitamin D_3 on Incident Fried Frailty Over Time

eFigure 8. Effect of Omega-3 on Incident Fried Frailty Over Time

eReferences

This supplementary material has been provided by the authors to give readers additional information about their work.

© 2022 Orkaby AR et al. *JAMA Network Open.*

eTable 1. Cumulative Deficit Frailty Index components for the VITAL trial

Author note: According to the accumulated deficit theory of frailty, deficits included in the frailty index are not weighted. Rather within an individual, the number of deficits present are counted and divided by the total variables determined at the outset (in this case, 36).[1,2]
The upper limit of the proportion of deficits that can be positive and still be compatible life is about 2/3, or a frailty index score or 0.7.

**Count**	**Variable**	Coding for the Frailty Index
	Health Status and Function	
	Does your health now limit you in these activities, and, if so, how much?	
1	Moderate activities such as moving a table, vacuuming, bowling, or golf	A lot =1, a little = 0.5, No = 0
2	Lifting or carrying groceries	A lot =1, a little = 0.5, No = 0
3	Climbing several flights of stairs	A lot =1, a little = 0.5, No = 0
4	Climbing one flight of stairs	A lot =1, a little = 0.5, No = 0
5	Bending, kneeling, stooping	A lot =1, a little = 0.5, No = 0
6	Walking more than a mile	A lot =1, a little = 0.5, No = 0
7	Walking several blocks	A lot =1, a little = 0.5, No = 0
8	Walking one block	A lot =1, a little = 0.5, No = 0
9	Bathing or dressing yourself	A lot =1, a little = 0.5, No = 0
	Activities of Daily Living	
10	Can you feed yourself	Without help =0, some help = 0.5, Unable = 1
11	Can you dress and undress yourself	Without help =0, some help = 0.5, Unable = 1
12	Can you get in and out of bed by yourself	Without help =0, some help = 0.5, Unable = 1
13	Can you take a bath or shower	Without help =0, some help = 0.5, Unable = 1
	Mood	
	Over the past 2 weeks, how often have you been bothered by any of the following:	
14	Little interest or pleasure in doing things	Not at all = 0, several days = 0.33, More than half the days = 0.67, nearly every day = 1
15	Feeling down, depressed or hopeless	Not at all = 0, several days = 0.33, More than half the days = 0.67, nearly every day = 1
16	Trouble falling or staying asleep, or sleeping too much	Not at all = 0, several days = 0.33, More than half the days = 0.67, nearly every day = 1
17	Feeling tired or having little energy	Not at all = 0, several days = 0.33, More than half the days = 0.67, nearly every day = 1
18	Poor appetite or overeating	Not at all = 0, several days = 0.33, More than half the days = 0.67, nearly every day = 1
19	Feeling bad about yourself or that you are a failure or have let yourself or your family down	Not at all = 0, several days = 0.33, More than half the days = 0.67, nearly every day = 1
20	Trouble concentrating on things like reading the paper or watching T.V.	Not at all = 0, several days = 0.33, More than half the days = 0.67, nearly every day = 1
21	Moving or speaking so slowly that others could have noticed? Or the opposite, being fidgety, restless, or moving a lot more than usual	Not at all = 0, several days = 0.33, More than half the days = 0.67, nearly every day = 1

© 2022 Orkaby AR et al. *JAMA Network Open.*

22	Diagnosis of depression	Yes =1, No =0
	Comorbidities	
23	Anemia	Yes = 1, No =0
24	Atrial Fibrillation	Yes=1, No =0
25	Hypertension	Yes=1, No =0
26	Lung comorbidity: asthma, chronic bronchitis, emphysema, or COPD	If answered Yes to any = 1, If all No then =0
27	Liver disease	Yes=1, No =0
28	Kidney Disease	Yes=1, No =0
29	Diabetes	Yes=1, No =0
30	Heart Failure	Yes = 1, No =0
31	Arthritis comorbidity: osteoarthritis, polymyalgia rheumatica, rheumatoid disease, psoriatic arthritis	If any are Yes = 1<br>If all No then =0
32	Parkinson's disease	Yes = 1, No =0
33	Macular degeneration	Yes = 1, No =0
34	Periodontal disease	Yes = 1, No =0
	Measured variables	
35	BMI	<18.5 or >30=1,<br>25-<30 = 0.5<br>18.5-<25 = 0
36	Weight loss	Unintentional weight loss of ≥5 lbs in the last year =1, otherwise =0

© 2022 Orkaby AR et al. *JAMA Network Open.*

eTable 2. Fried physical phenotype of frailty in VITAL subcohort (n=1054)

Variable	Men	Women
Shrinking: Weight loss	Unintentional weight loss ≥5 lb in the last 2 years	
Exhaustion	Answered “More than half the days” or “nearly every day” to the question “Feeling tired or having little energy”	
Low physical activity	kcal of energy expended in a week in the lowest 20% for men	kcal of energy expended in a week in the lowest 20% for women
Slow: walking speed m/s	Height ≤173 cm: ≤0.96<br>Height >173 cm: ≤1.04	Height ≤173 cm: ≤0.90<br>Height >173 cm: ≤1.02
Weakness: Grip strength (kg)	BMI ≤ 24.0: ≤ 29<br>BMI 24.1-26.0: ≤ 30<br>BMI 26.1-28.0: ≤ 30<br>BMI > 28.0: ≤ 32	BMI ≤ 23.0: ≤ 17<br>BMI 23.1 to 26.0: ≤ 17.3<br>BMI 26.1 to 29.0: ≤ 18<br>BMI > 29.0: ≤ 21

Calculating frailty: Participants with ≥3 criteria are considered frail, those with 1-2 criteria are pre-frail while those with none are robust.

© 2022 Orkaby AR et al. *JAMA Network Open.*

eTable 3. Baseline Characteristics of the VITAL trial (n=25,057) According to Frailty Status

	**Non-frail (n=14598)**	**Pre-frail (n=7285)**	**Frail (n=3174)**
**Age (mean, SD)**	66.8 (6.7)	67.8 (7.3)	67.2 (7.8)
**Female sex %**	43.4	57.7	68.1
**Race or ethnic group**[a]			
**Asian/Pacific Islander**	1.7	1.2	1.0
**Black or African American**	14.3	23.0	37.5
**Native American/Alaskan Native**	0.8	1.0	1.4
**Non-Black Hispanic**	3.8	3.9	4.6
**Non-Hispanic white**	77.7	68.8	52.7
**Other or unknown**	1.8	2.2	2.9
**Body Mass Index(kg/m^2)**[b]	26.3 (4.2)	29.5 (5.7)	33.1 (7.8)
**Current smoker %**	5.1	8.1	14.3
**Daily alcohol use %**	30.8	22.7	13.2
**Baseline aspirin use %**	42.9	49.5	48.0
**Baseline statin use %**	30.7	40.3	43.0
**Baseline dietary fish consumption ≥1.5 servings/wk %**	47.4	46.1	46.0
**Baseline 25(OH)D levels (ng/ml) (mean, SD)**	31.55 (9.7)	30.5 (10.1)	28.2 (10.8)
**Leisure-time physical activity and stair climbing, total MET-hours/week, mean (SD)**	27.2 (27.18)	18.7 (22.5)	10.9 (19.5)
**Randomized to omega-3 %**	50.1	49.6	50.4
**Randomized to vitamin D_3 %**	49.7	49.9	50.6

[a] Other race or ethnicity categories include: patients who indicated other or unknown race or ethnicity
[b] Body mass index is weight in kilograms divided by the square of height in meters.
[c] Alcohol use >= 1 drink per day wine (5oz glass), liquor (shot) or beer (bottle, can or glass)
[d] At least monthly

© 2022 Orkaby AR et al. *JAMA Network Open.*

eTable 4. Adjusted Means at Baseline and Mean Change (95% CI) in Frailty level at Each Year Since Randomization Compared to Baseline, according to Vitamin D vs placebo and Omega-3 vs Placebo Groups[a]

	No. participants	Mean change (SE)[b]	No. participants	Mean change (SE)[b]	Mean difference in change (95% CI)[c]	P-value	P-interaction[d]
	Vitamin D group		Placebo group				
Baseline	12497	0.1097 (0.0008)	12560	0.1092 (0.0008)	--	--	
Year 3 vs Baseline	11356	0.0069 (0.00056)	11405	0.0068 (0.00056)	0.00016 (-0.0014, 0.0017)	0.84	
Year 4 vs Baseline	10760	0.0117 (0.00061)	10689	0.0118 (0.00062)	-0.00004 (-0.0018, 0.0017)	0.96	0.98
Year 5 vs Baseline	7154	0.0177 (0.00072)	7133	0.0179 (0.00071)	-0.0002 (-0.0022, 0.0018)	0.85	
**Average (across Years 3-5) vs Baseline[e]**	**12497**		**12560**		**0.00005 (-0.0014, 0.0015)**	**0.94**	
	Omega-3 group		Placebo group				
Baseline	12529	0.1092 (0.0008)	12528	0.1097 (0.0008)	--	--	
Year 3 vs Baseline	11376	0.0064 (0.00056)	11385	0.0073 (0.00056)	-0.00087 (-0.00243, 0.00069)	0.27	
Year 4 vs Baseline	10741	0.0109 (0.00061)	10708	0.0126 (0.00062)	-0.00167 (-0.00338, 0.00003)	0.05	
Year 5 vs Baseline	7179	0.0178 (0.00072)	7108	0.0179 (0.00071)	-0.00013 (-0.00211, 0.00185)	0.90	0.13
**Average (across Years 3-5) vs Baseline[e]**	**12529**		**12528**		**-0.00099 (-0.00245, 0.00047)**	**0.18**	

© 2022 Orkaby AR et al. *JAMA Network Open.*

Legend: Abbreviation: CI, confidence interval: Rockwood frailty level; SE, standard error.

P-values are provided for the mean difference in frailty level change over time.

[a]Analyses are presented from general linear models of response profiles to estimate the means, with time modeled as indicator variables, and models were controlled for randomization group (vitamin D_3 or omega-3). Adjusted means (95% CI) within each treatment group are shown at baseline and adjusted mean differences in change (95% CI) within each treatment group are shown for each follow-up time point. The repeated measures model used the available responses. All participants contributed data to the repeated measures analysis at one or more times.

[b]The first row shows mean (SE) of the FI at baseline, subsequent rows show the mean change (SE)

[c]Net difference in change comparing active and placebo group from the repeated measures model.

[d]P-interaction is from the test of the interaction term treatment-x-time in the model

[e]The final raw shows the net difference between the treatment groups in frailty level averaged across follow-up years (3-5)

© 2022 Orkaby AR et al. *JAMA Network Open.*

**eTable 5** Baseline Characteristics of 1054 participants in the VITAL trial – in person subgroup				
	**Vitamin D_3 and Omega-3s N=261**	**Vitamin D_3 only N=259**	**Omega-3 only N=266**	**Placebo only N=268**
**Age (mean, SD)**	64.7 (6.5)	64.6 (6.2)	65.0 (6.5)	65.2 (6.7)
**Female sex %**	49.4	49.0	49.3	47.8
**Race or ethnic group %** [a]				
**Asian/Pacific Islander**	2.4	2.0	1.9	0.8
**Black or African American**	8.6	7.8	8.1	9.5
**Native American/Alaskan Native**	0.4	0.4	1.5	0
**Non Black Hispanic**	3.5	1.2	2.7	3.8
**Non-Hispanic white**	82.8	85.2	84.2	85.5
**Other or unknown**	2.4	3.5	1.5	0.4
**Body Mass Index(kg/m^2)** [b]	28.4 (5.3)	27.8 (5.3)	28.9 (5.4)	27.7 (4.9)
**Current smoker %**	5.8	5.9	4.1	6.0
**Daily alcohol use %**[c]	30.7	31.3	32.9	29.7
**Current regular aspirin use %**[d]	47.7	41.7	46.8	38.7
**Current use of statin %**	36.9	31.4	32.8	32.2
**Baseline 25(OH)D levels (ng/ml) (mean, SD)**	27.7 (8.7)	27.5 (8.9)	28.9 (10.2)	28.4 (8.4)
**Baseline dietary fish consumption ≥1.5 servings/wk %**	60.1	53.3	57.3	57.4
**Leisure-time physical activity and stair climbing, total MET-hours/week, mean (SD)**	30.9 (31.9)	24.1 (21.2)	26.1 (25.3)	27.4 (25.4)
**Fried frailty score, Median (IQR)**	0.0 (0.0-1.0)	0.0 (0.0-1.0)	0.0 (0.0-1.0)	0.0 (0.0-1.0)

There were no significant differences between groups with regards to baseline characteristics.
[a] Race and ethnic group were reported by participants. Other race or ethnicity categories include: patients who indicated other or unknown race or ethnicity.
[b] Body mass index is weight in kilograms divided by the square of height in meters.
[c] Alcohol use >= 1 drink per day wine (5oz glass), liquor (shot) or beer (bottle, can or glass)
[d] At least monthly.

© 2022 Orkaby AR et al. *JAMA Network Open.*

eTable 6A. Adjusted Means at Baseline and Mean Change (95% CI) in Fried frailty level at Each Year Since Randomization Compared to Baseline, according to Vitamin D and Placebo Groups*

	**Vitamin D**		**Placebo group**		**Mean difference in change (95% CI)** [b]	**P-value**	**P-interaction**[a]
	No. of participants	Mean (SE)	No. of participants	Mean (SE)			
Baseline	520	0.5558 (0.0345)	534	0.5262 (0.0344)			**0.59**
Year 2 vs Baseline	472	0.1697 (0.0334)	495	0.1696 (0.0321)	0.00005 (-0.0908-0.0909)	0.999	
Year 4 vs Baseline	350	0.1711 (0.0445)	338	0.2339 (0.0445)	-0.063(-0.1863-0.0608)	0.32	
**Average (across Years 2, 4) vs Baseline**	**520**		**534**		**-0.1922 (-0.1001-0.0616)**	**0.64**	

© 2022 Orkaby AR et al. *JAMA Network Open.*

eTable 6B. Adjusted Means at Baseline and Mean Change (95% CI) in Fried frailty score at Each Year Since Randomization Compared to Baseline, according to n-3 and Placebo Groups*

	**Omega-3 group**		**Placebo group**		**Mean difference in change (95% CI)** [b]	**P-value**	**P-interaction**[a]
	No. of participants	Mean (SE)	No. of participants	Mean (SE)			**0.53**
Baseline	527	0.5408 (0.0348	527	0.5408 (0.0341)			
Year 2 vs Baseline	481	0.1706(0.0331)	486	0.1685 (0.0324)	0.0021 (-0.0887-0.0929)	0.96	
Year 4 vs Baseline	344	0.1681(0.0456)	344	0.2360 (0.0435)	-0.0679 (-0.1914-0.0557)	0.28	
**Average (across Years 2, 4) vs Baseline ****	**527**		**527**		**-0.0194 (-0.1002-0.0615)**	**0.64**	

Abbreviation: CI, confidence interval: Fried frailty.

*** Analyses are presented from general linear models of response profiles to estimate the means, with time modeled as indicator variables; models were controlled for randomization group (vitamin D , Omega-3). Adjusted means (95% CI) within each treatment group are shown at baseline and adjusted mean differences in change (95% CI) are shown within each treatment group for each follow-up time point. All participants contributed data to the repeated measures analysis one or more times.**

[a]P-interaction is from the test of the interaction term treatment-x-time.

[b]Net mean difference in change comparing active and placebo group from the repeated measures model.

[**]The final raw shows the net difference between the treatment groups in frailty level averaged across follow-up years (2,4) .

P-value is from Type 3 Tests of Fixed Effects treatment-x-time (years 2 and 4)) in the repeated measures model.

© 2022 Orkaby AR et al. *JAMA Network Open.*

eFigure 1. Distribution of Frailty Index at baseline

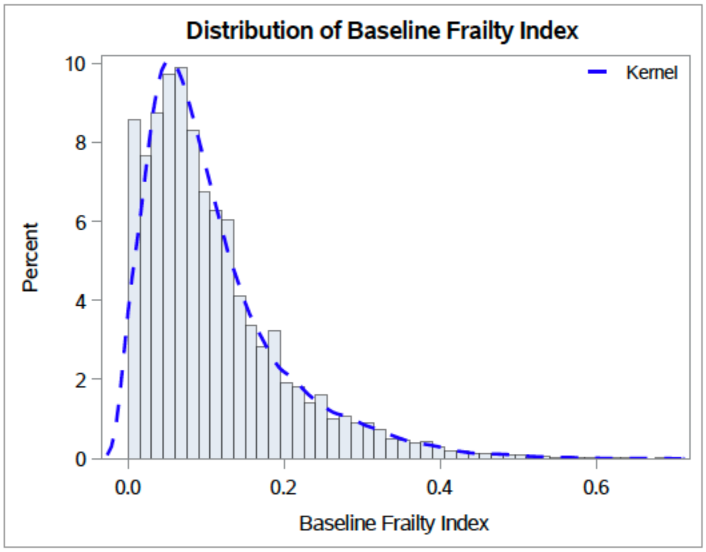

© 2022 Orkaby AR et al. *JAMA Network Open.*

eFigure 2. Distribution of Frailty Index at baseline according to randomization group

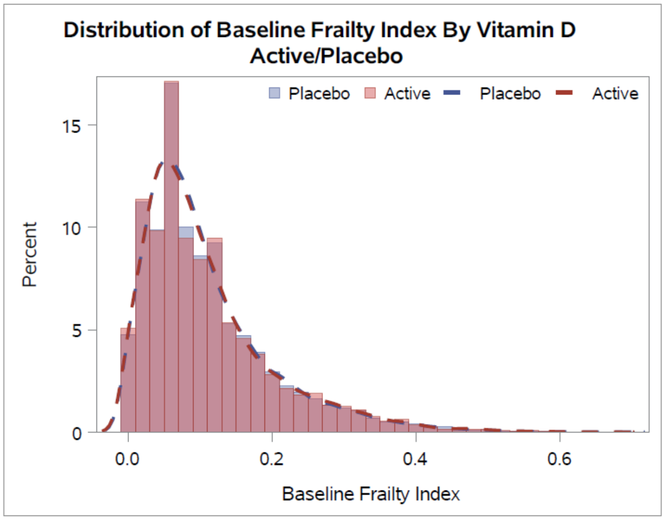

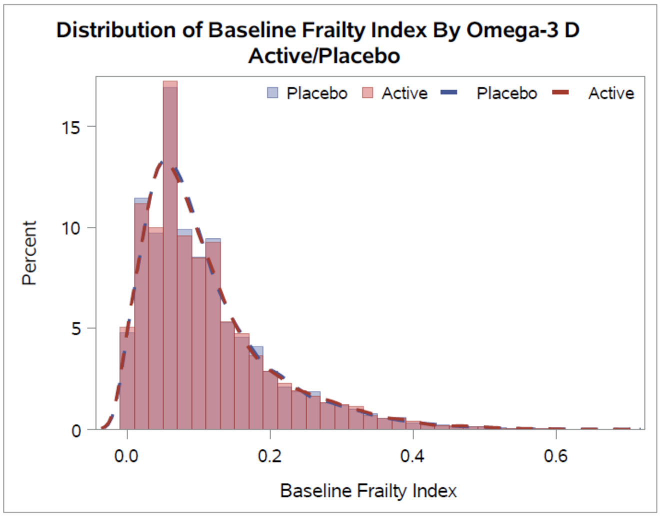

© 2022 Orkaby AR et al. *JAMA Network Open.*

eFigure 3. Validation of the FI: Association between FI level and mortality

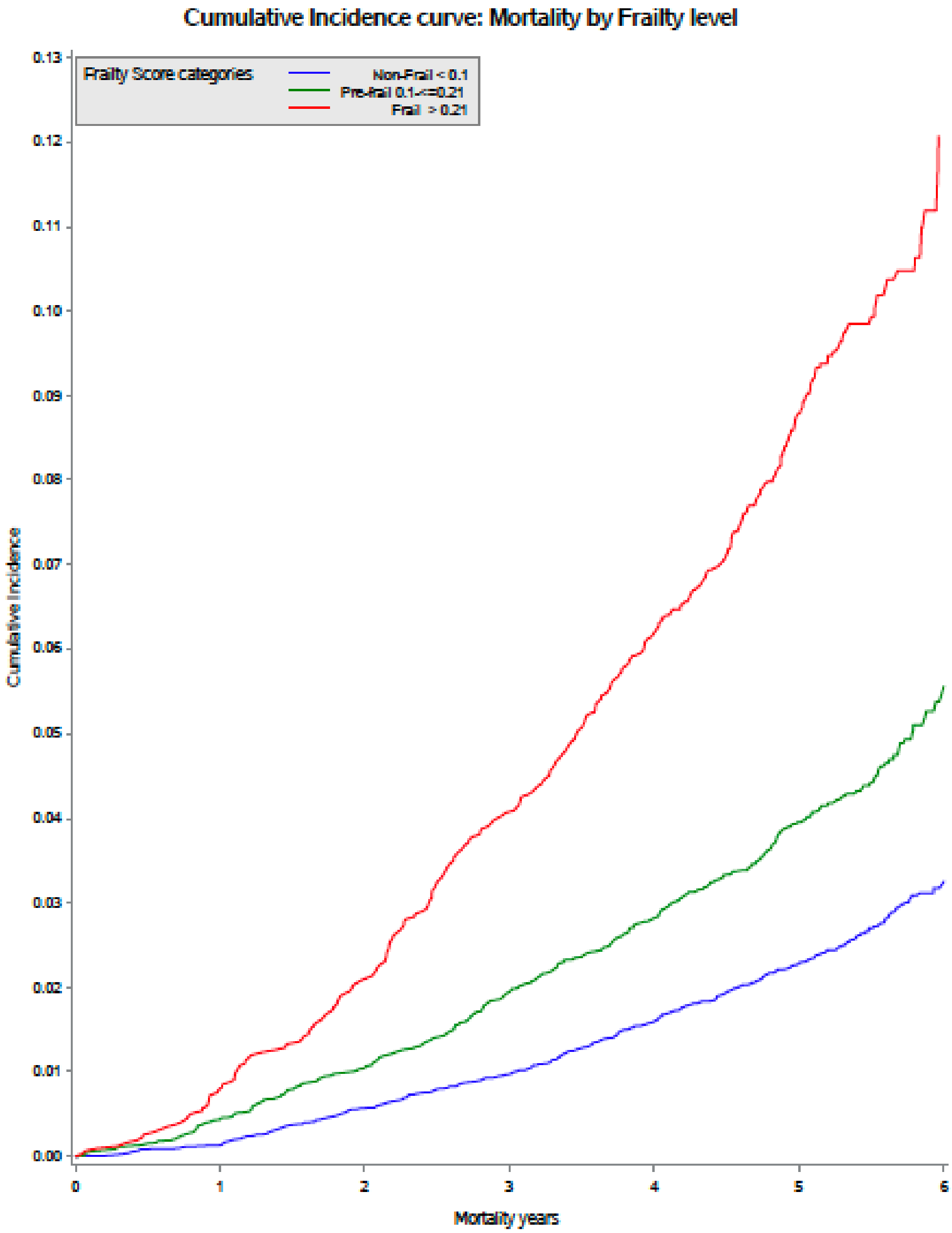

Frailty level	Hazard Ratio for mortality	95% CI	p-value
Robust (<0.1)	Ref	--	--
Pre-frail (0.1-0.2)	1.67	1.44-1.95	<0.001
Frail (>0.21)	4.10	3.51-4.78	<0.001

© 2022 Orkaby AR et al. *JAMA Network Open.*

eFigure 4A. Effect of vitamin D_3 on incident frailty over time

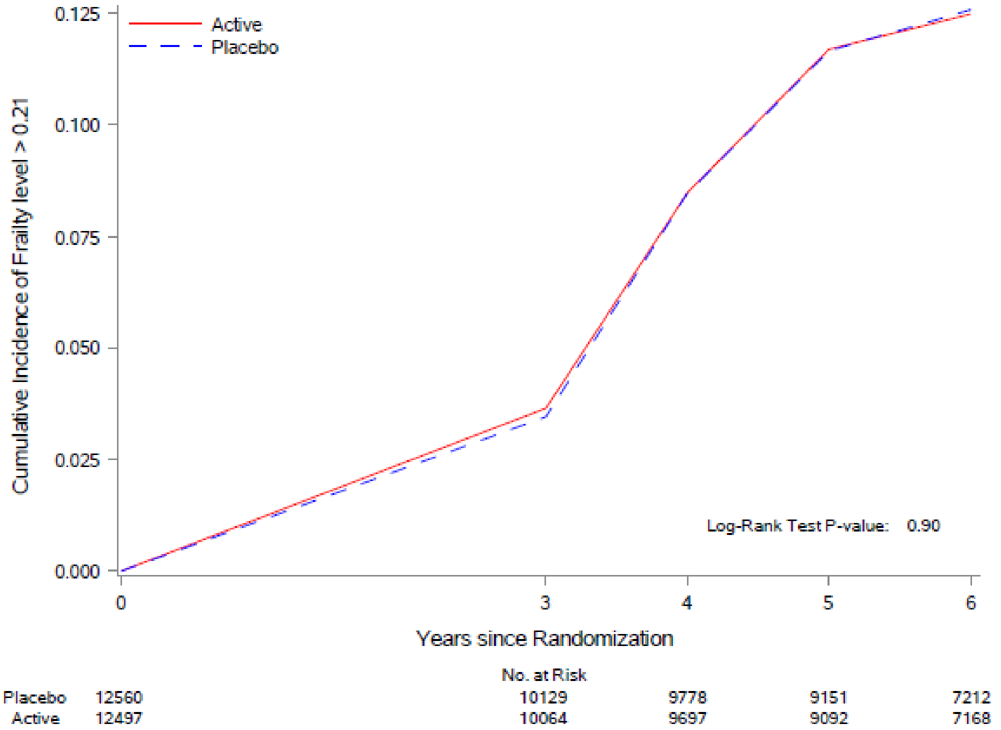

eFigure 4B Effect of omega-3 on incident frailty over time

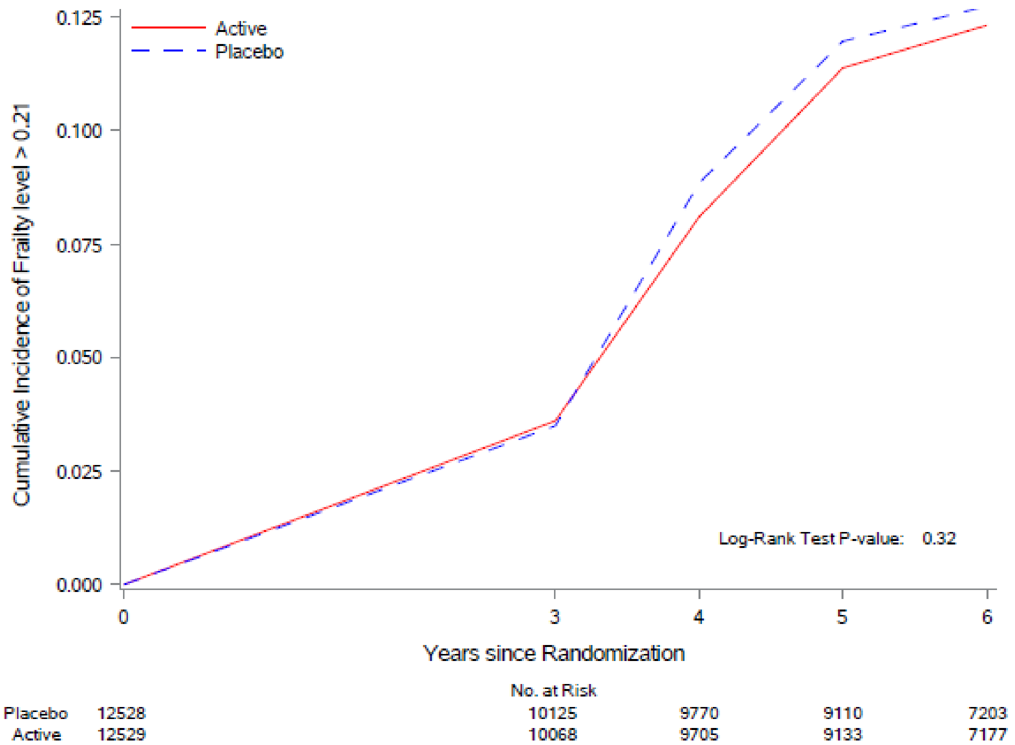

© 2022 Orkaby AR et al. *JAMA Network Open.*

eFigure 5. Vitamin D_3 supplementation and frailty subgroups

a. Vitamin D_3 supplementation and frailty, stratified by age*

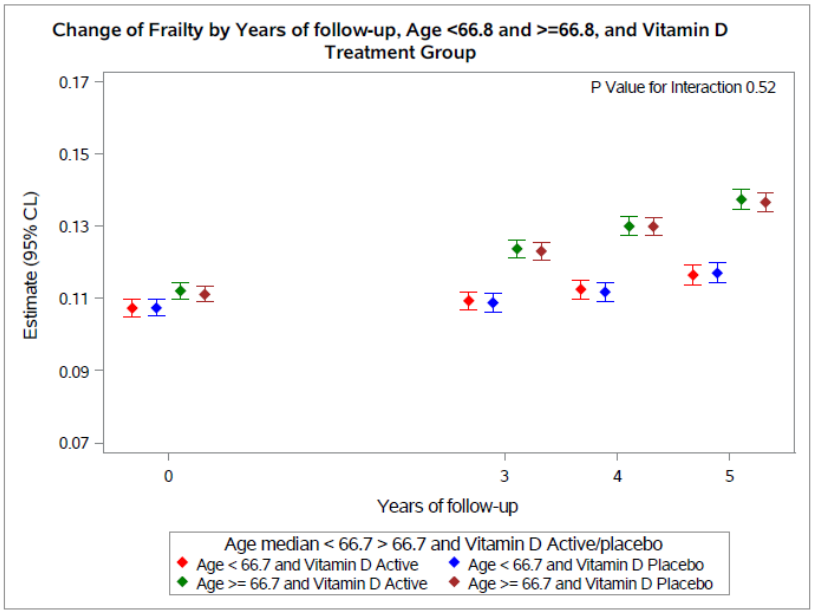

*p-interaction is between vitamin D and median age

b. Vitamin D_3 supplementation and frailty, stratified by sex

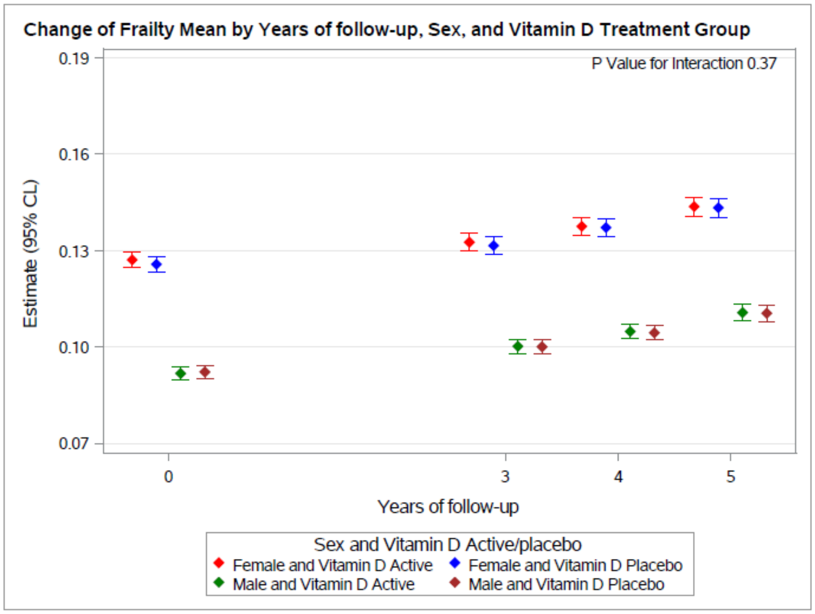

© 2022 Orkaby AR et al. *JAMA Network Open.*

c. Vitamin D3 supplementation and frailty, stratified by race/ethnicity

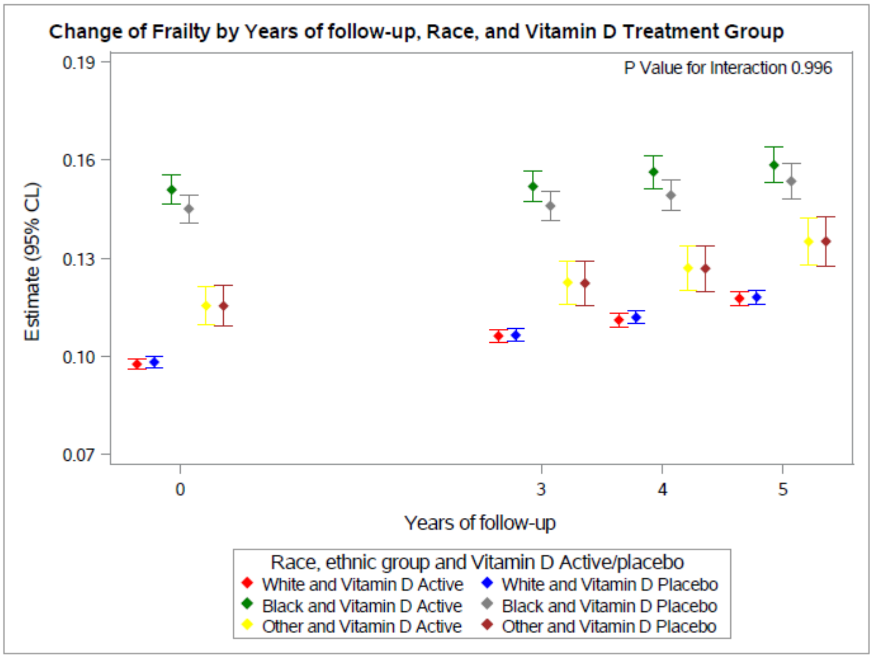

d. Vitamin D3 supplementation and frailty, stratified by baseline 25(OH)D

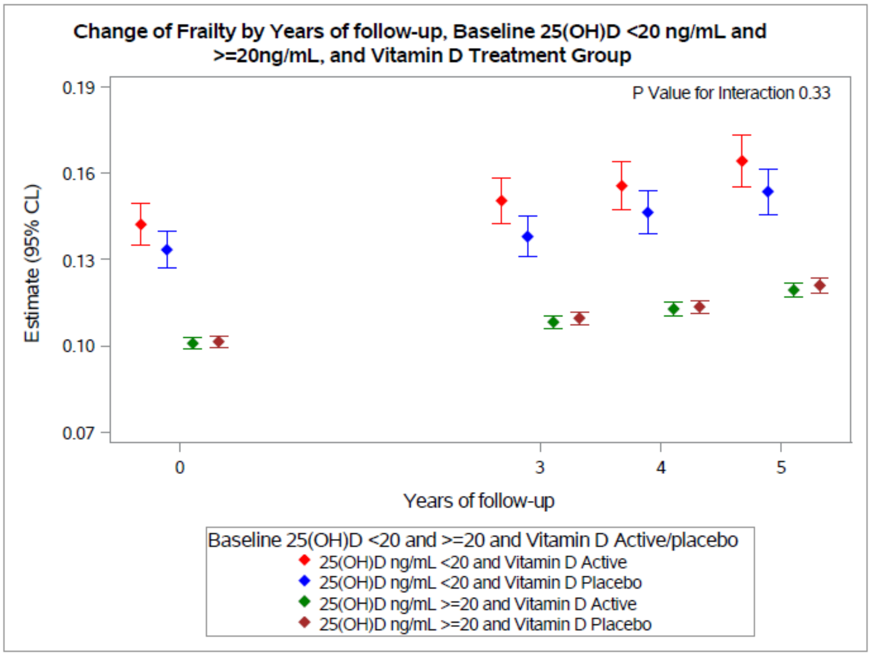

© 2022 Orkaby AR et al. *JAMA Network Open.*

e. Vitamin D_3 supplementation and frailty, stratified by BMI

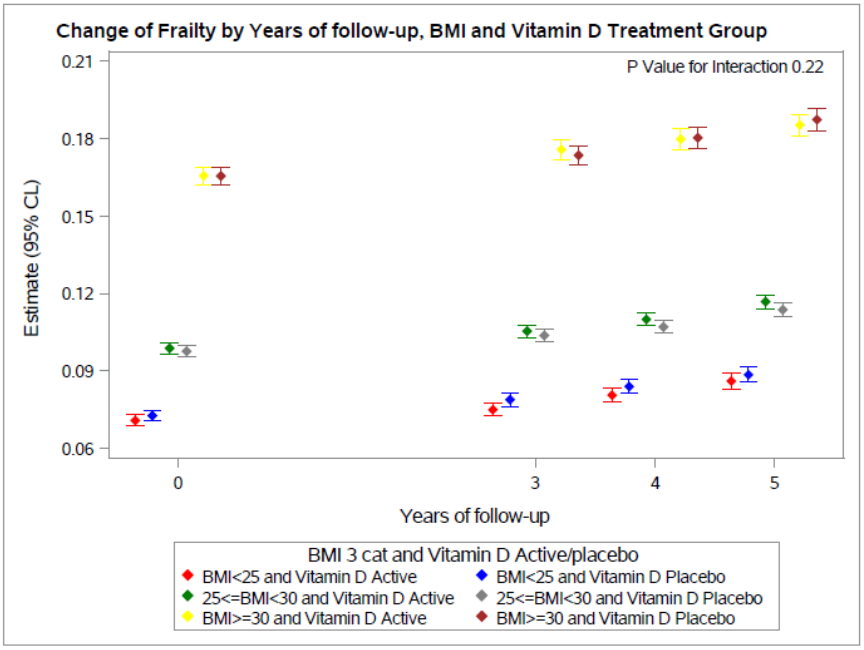

© 2022 Orkaby AR et al. *JAMA Network Open.*

eFigure 6. Omega-3 Supplementation and frailty subgroups

a. Omega 3 supplementation and frailty, stratified by age

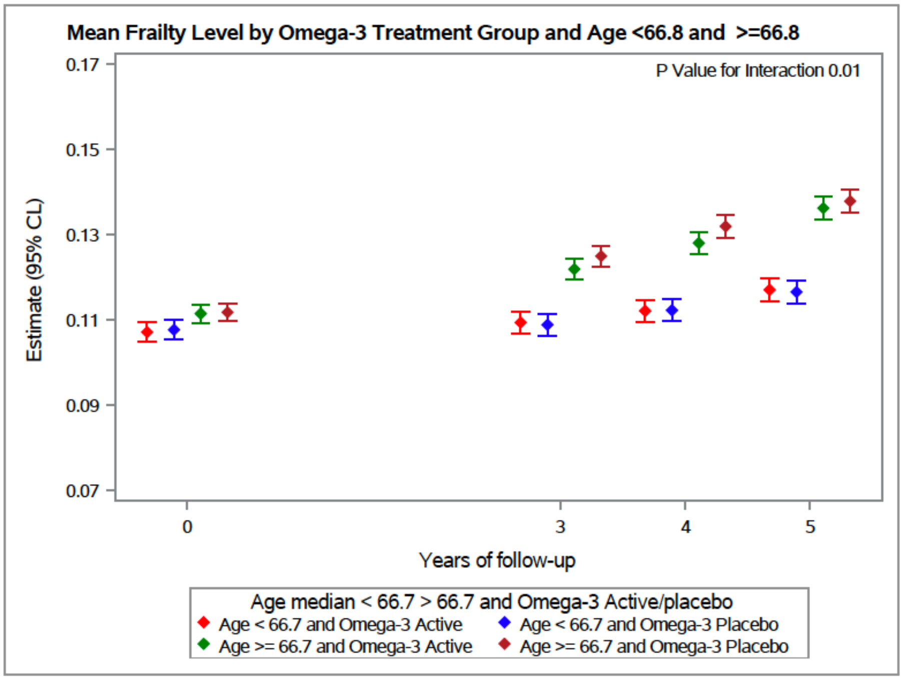

b. Omega 3 supplementation and frailty, stratified by sex

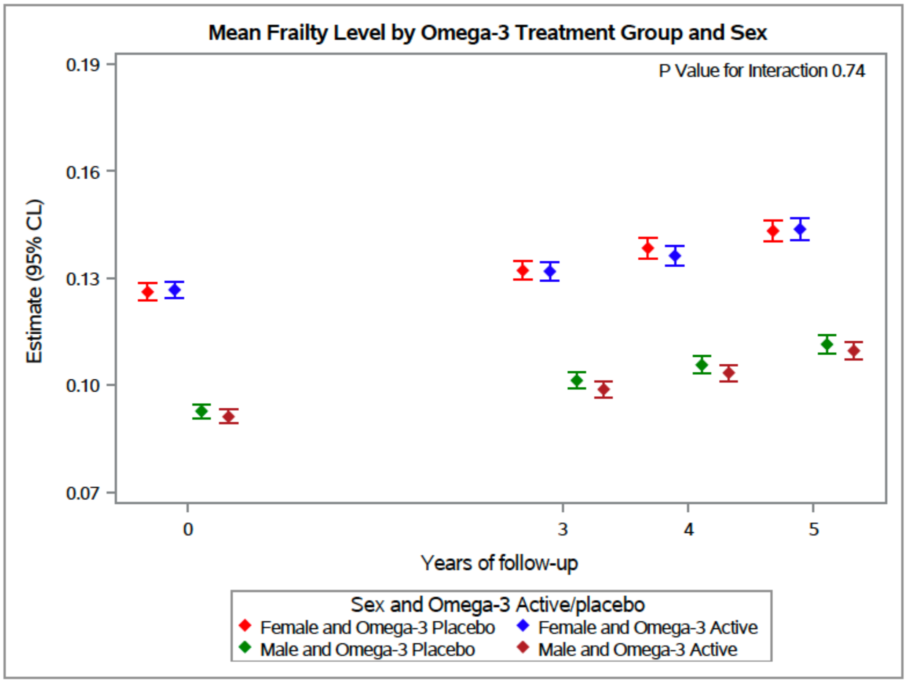

© 2022 Orkaby AR et al. *JAMA Network Open.*

c. Omega 3 supplementation and frailty, stratified by race/ethnicity

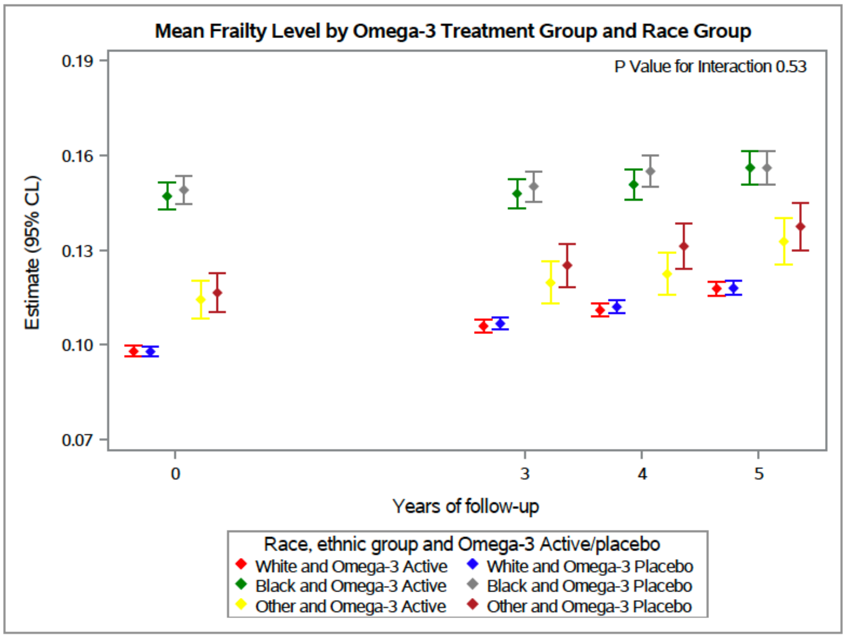

d. Omega 3 supplementation and frailty, stratified by baseline fish intake

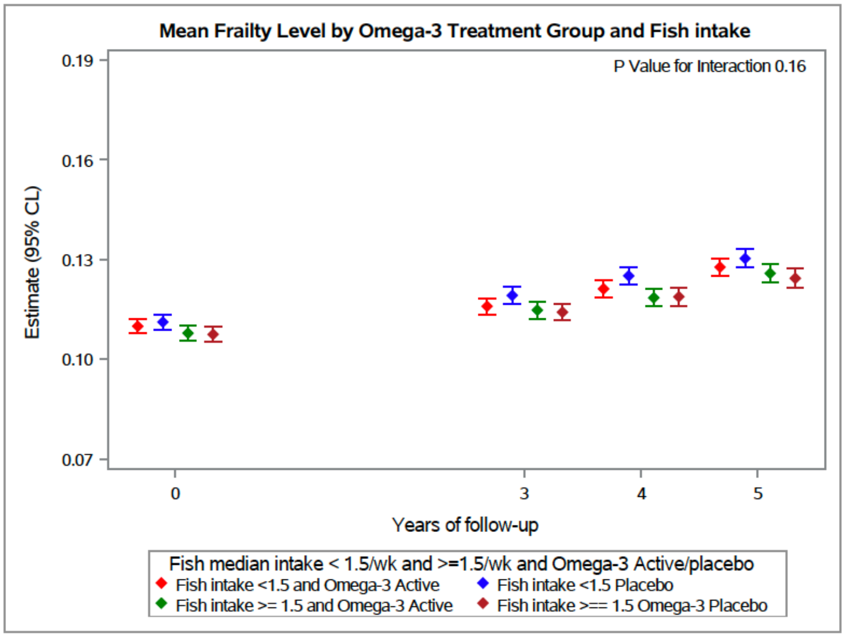

© 2022 Orkaby AR et al. *JAMA Network Open.*

eFigure 7. Effect of Vitamin D_3 on incident Fried frailty over time

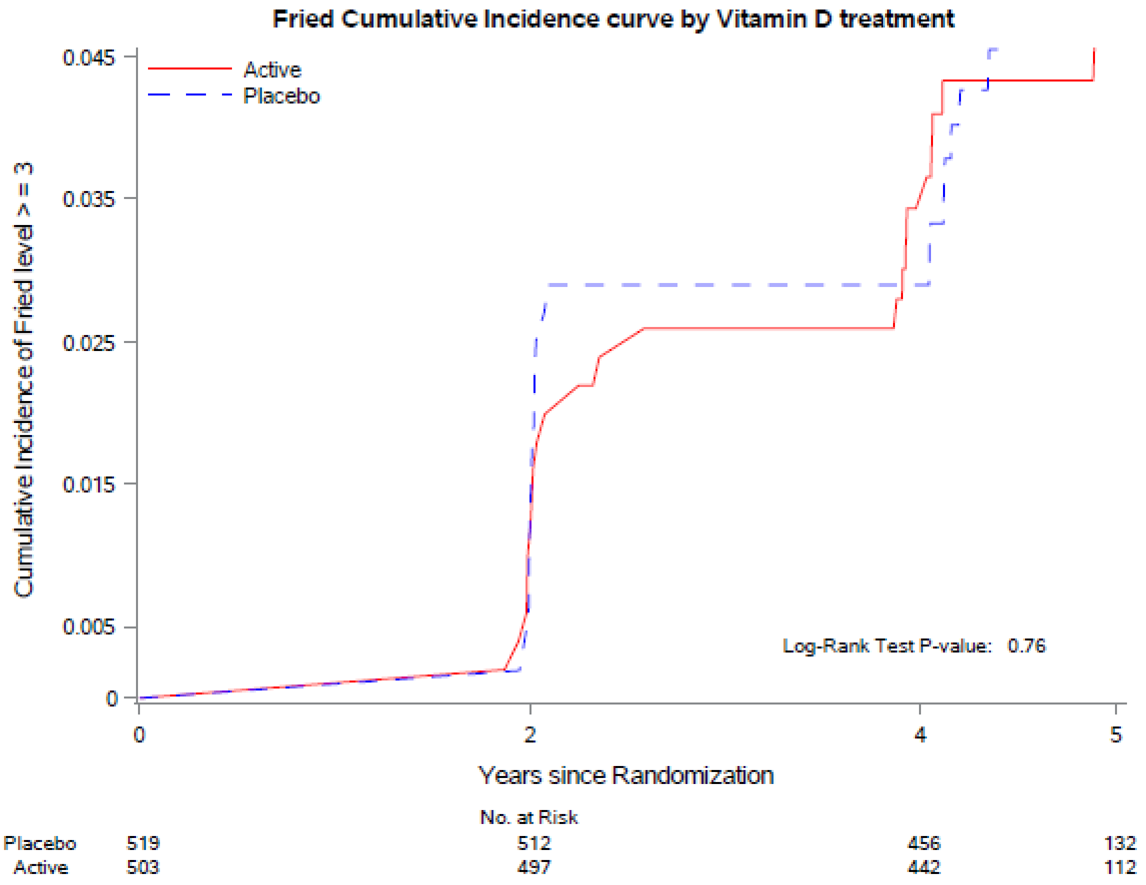

© 2022 Orkaby AR et al. *JAMA Network Open.*

eFigure 8. Effect of omega-3 on incident Fried frailty over time

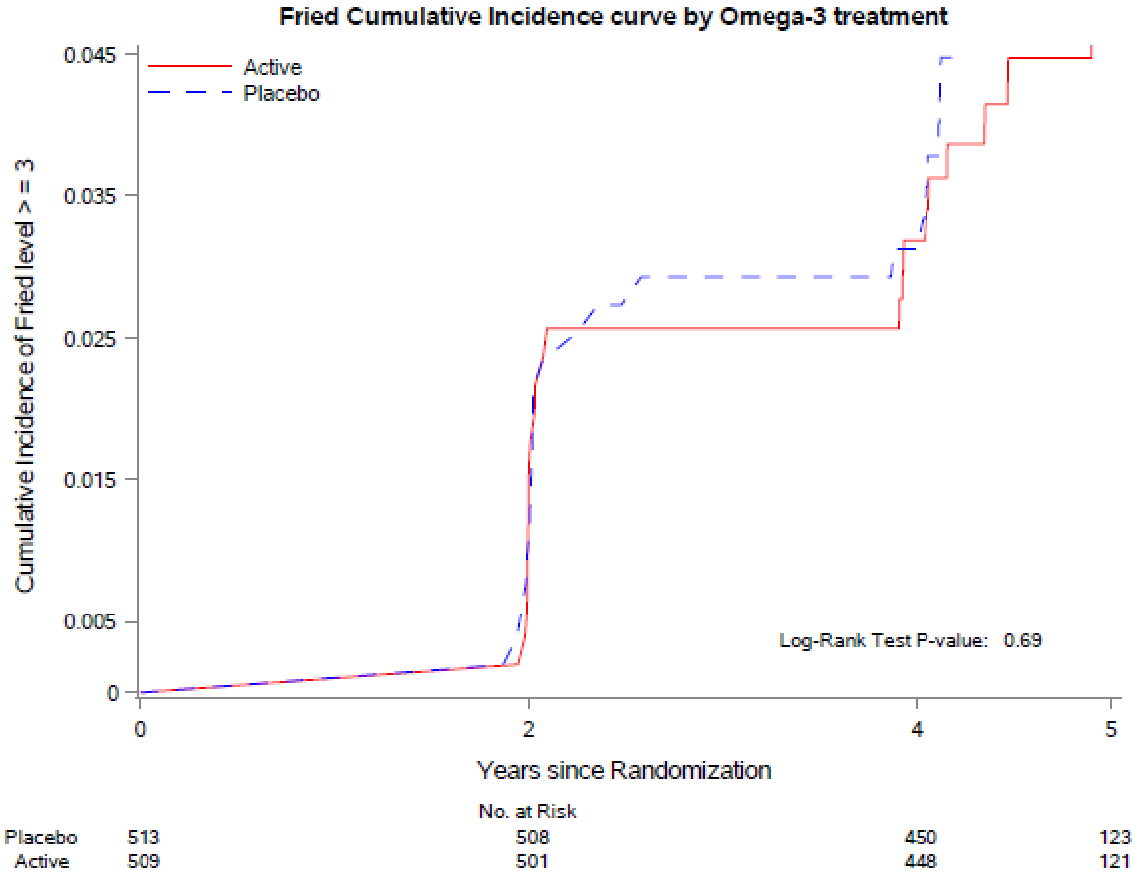

© 2022 Orkaby AR et al. *JAMA Network Open.*

eReferences

1. Searle SD, Mitnitski A, Gahbauer EA, Gill TM, Rockwood K. A standard procedure for creating a frailty index. *BMC geriatrics* 2008; **8**: 24.
2. Rockwood K, Mitnitski A. Frailty in relation to the accumulation of deficits. *The journals of gerontology Series A, Biological sciences and medical sciences* 2007; **62**(7): 722-7.

© 2022 Orkaby AR et al. *JAMA Network Open.*
