# Supplementary material for: Effect of Vitamin D3 and Omega-3 Fatty Acid Supplementation on Risk of Frailty: An Ancillary Study of a Randomized Clinical Trial
Source: JAMA Netw Open. 2022 Sep 13;5(9):e2231206. doi: 10.1001/jamanetworkopen.2022.31206 (PMC9471979; doi:10.1001/jamanetworkopen.2022.31206)
Supplement: Supplementary file 4 [file jamanetwopen-e2231206-s004.pdf]

Data Sharing Statement

Orkaby. Effect of Vitamin D_3 and Omega-3 Fatty Acid Supplementation on Risk of Frailty. *JAMA Netw Open*. Published September 13, 2022. doi:10.1001/jamanetworkopen.2022.31206

Data
Data available: Yes
Data types: Deidentified participant data, Data dictionary
How to access data: The PI of this analysis (aorkaby@bwh.harvard.edu) can be contacted for de-identified data requests
When available: With publication

Supporting Documents
Document types: None

Additional Information
Who can access the data: Researchers where the proposed use of the data is approved as outlined in the data sharing statement
Types of analyses: Analyses will need to be for a specified purpose
Mechanisms of data availability: Analyses proposal requests will require review and approval by the VITAL Publications & Presentations Committee and appropriate IRB approval. Once approved and a data access agreement has been executed, deidentified data generated from this research will be made available to affiliated investigators through secure databases for the prespecified analysis.
